# Global meta-analysis shows action is needed to halt genetic diversity loss

**DOI:** 10.1038/s41586-024-08458-x

**Published:** 2025-01-29

**Authors:** Robyn E. Shaw, Katherine A. Farquharson, Michael W. Bruford, David J. Coates, Carole P. Elliott, Joachim Mergeay, Kym M. Ottewell, Gernot Segelbacher, Sean Hoban, Christina Hvilsom, Sílvia Pérez-Espona, Dainis Ruņģis, Filippos Aravanopoulos, Laura D. Bertola, Helena Cotrim, Karen Cox, Vlatka Cubric-Curik, Robert Ekblom, José A. Godoy, Maciej K. Konopiński, Linda Laikre, Isa-Rita M. Russo, Nevena Veličković, Philippine Vergeer, Carles Vilà, Vladimir Brajkovic, David L. Field, William P. Goodall-Copestake, Frank Hailer, Tara Hopley, Frank E. Zachos, Paulo C. Alves, Aleksandra Biedrzycka, Rachel M. Binks, Joukje Buiteveld, Elena Buzan, Margaret Byrne, Barton Huntley, Laura Iacolina, Naomi L. P. Keehnen, Peter Klinga, Alexander Kopatz, Sara Kurland, Jennifer A. Leonard, Chiara Manfrin, Alexis Marchesini, Melissa A. Millar, Pablo Orozco-terWengel, Jente Ottenburghs, Diana Posledovich, Peter B. Spencer, Nikolaos Tourvas, Tina Unuk Nahberger, Pim van Hooft, Rita Verbylaite, Cristiano Vernesi, Catherine E. Grueber

**Affiliations:** 1 International Union for the Conservation of Nature (IUCN) Conservation Genetics Specialist Group (CGSG), https://www.cgsg.uni-freiburg.de/; 2 Biodiversity and Conservation Science, Department of Biodiversity, Conservation and Attractions, Kensington, Western Australia Australia; 3 School of Environmental and Conservation Sciences, Murdoch University, Perth, Western Australia Australia; 4 Division of Ecology and Evolution, Research School of Biology, The Australian National University, Canberra, Australian Capital Territory Australia; 5 Centre for Conservation Ecology and Genomics, University of Canberra, Canberra, Australian Capital Territory Australia; 6 School of Life and Environmental Sciences, Faculty of Science, The University of Sydney, Camperdown, New South Wales Australia; 7 Australian Research Council Centre of Excellence for Innovations in Peptide and Protein Science, The University of Sydney, Camperdown, New South Wales Australia; 8 School of Biosciences, Museum Avenue, Cardiff University, Cardiff, UK; 9 European Cooperation in Science and Technology (COST), COST Action CA 18134 ‘Genomic Biodiversity Knowledge for Resilient Ecosystems (G-BiKE)’, https://www.cost.eu/actions/ca18134/; 10 School of Biological Sciences, The University of Western Australia, Crawley, Western Australia Australia; 11 Research Institute for Nature and Forest, Geraardsbergen, Belgium; 12 Ecology, Evolution and Biodiversity Conservation, KU Leuven, Leuven, Belgium; 13 Wildlife Ecology and Management, University Freiburg, Freiburg, Germany; 14 The Center for Tree Science, The Morton Arboretum, Lisle, IL USA; 15 Copenhagen Zoo, Frederiksberg, Denmark; 16 The Royal (Dick) School of Veterinary Studies and The Roslin Institute, The University of Edinburgh, Easter Bush Campus, Midlothian, UK; 17 Genetic Resource Centre, Latvian State Forest Research Institute “Silava”, Salaspils, Latvia; 18 Laboratory of Forest Genetics and Tree Breeding, Faculty of Agriculture, Forestry and Natural Environment, Aristotle University of Thessaloniki, Thessaloniki, Greece; 19 Department of Biology, University of Copenhagen, Copenhagen, Denmark; 20 cE3c—Center for Ecology, Evolution and Environmental Change and CHANGE—Global Change and Sustainability Institute, Faculdade de Ciências, Universidade de Lisboa, Lisboa, Portugal; 21 Department of Animal Science, University of Zagreb Faculty of Agriculture, Zagreb, Croatia; 22 Wildlife Analysis Unit, Swedish Environmental Protection Agency, Stockholm, Sweden; 23 Estación Biológica de Doñana (EBD-CSIC), Seville, Spain; 24 Institute of Nature Conservation, Polish Academy of Sciences, Kraków, Poland; 25 Department of Zoology, Division of Population Genetics, Stockholm University, Stockholm, Sweden; 26 Department of Biology and Ecology, Faculty of Sciences, University of Novi Sad, Novi Sad, Serbia; 27 Plant Ecology and Nature Conservation Group, Wageningen University, Wageningen, The Netherlands; 28 Applied BioSciences, Macquarie University, Sydney, New South Wales Australia; 29 School of Science, Edith Cowan University, Joondalup, Western Australia Australia; 30 Royal Botanic Garden Edinburgh, Edinburgh, UK; 31 Institute of Zoology Joint Laboratory for Biocomplexity Research (CIBR), Chinese Academy of Sciences, Beijing, China; 32 Royal Botanic Gardens Victoria, Melbourne, Victoria Australia; 33 Natural History Museum Vienna, Vienna, Austria; 34 Department of Evolutionary Biology, University of Vienna, Vienna, Austria; 35 Department of Genetics, University of the Free State, Bloemfontein, South Africa; 36 Research Institute for the Environment and Livelihoods, Charles Darwin University, Casuarina, Northern Territory Australia; 37 CIBIO, Centro de Investigação em Biodiversidade e Recursos Genéticos, InBIO/ BIOPOLIS Program in Genomics, Biodiversity and Land Planning, University of Porto, Porto, Portugal; 38 Department of Biology, Faculty of Sciences, University of Porto, Porto, Portugal; 39 EBM, Biological Station of Mértola, Mértola, Portugal; 40 Centre for Genetic Resources, The Netherlands, Wageningen University, Wageningen, The Netherlands; 41 Faculty of Mathematics, Natural Sciences and Information Technologies, University of Primorska, Koper, Slovenia; 42 Faculty of Environmental Protection, Velenje, Slovenia; 43 Department of Veterinary Medicine, University of Sassari, Sassari, Italy; 44 Department of Chemistry and Bioscience, Aalborg University, Aalborg, Denmark; 45 Department of Ecology, Swedish University of Agricultural Sciences, Uppsala, Sweden; 46 Department of Phytology, Faculty of Forestry, Technical University in Zvolen, Zvolen, Slovakia; 47 Department of Forest Ecology, Faculty of Forestry and Wood Sciences, Czech University of Life Sciences Prague, Prague, Czech Republic; 48 Norwegian Institute for Nature Research (NINA), Trondheim, Norway; 49 Department of Earth Sciences, Natural Resources and Sustainable Development, Uppsala University, Uppsala, Sweden; 50 Department of Life Sciences, University of Trieste, Trieste, Italy; 51 Research Institute on Terrestrial Ecosystems (IRET), The National Research Council of Italy (CNR), Porano, Italy; 52 National Biodiversity Future Center, Palermo, Italy; 53 Wildlife Ecology and Conservation Group, Wageningen University, Wageningen, The Netherlands; 54 Forest Ecology and Forest Management, Wageningen University, Wageningen, The Netherlands; 55 Slovenian Forestry Institute, Ljubljana, Slovenia; 56 Department of Forest Genetics and Tree Breeding, Institute of Forestry, Lithuanian Research Centre for Agriculture and Forestry, Kėdainiai, Lithuania; 57 Forest Ecology Unit, Research and Innovation Centre, Fondazione Edmund Mach, San Michele all’Adige, Italy

**Keywords:** Genetic variation, Conservation biology

## Abstract

Mitigating loss of genetic diversity is a major global biodiversity challenge^1–4^. To meet recent international commitments to maintain genetic diversity within species^5,6^, we need to understand relationships between threats, conservation management and genetic diversity change. Here we conduct a global analysis of genetic diversity change via meta-analysis of all available temporal measures of genetic diversity from more than three decades of research. We show that within-population genetic diversity is being lost over timescales likely to have been impacted by human activities, and that some conservation actions may mitigate this loss. Our dataset includes 628 species (animals, plants, fungi and chromists) across all terrestrial and most marine realms on Earth. Threats impacted two-thirds of the populations that we analysed, and less than half of the populations analysed received conservation management. Genetic diversity loss occurs globally and is a realistic prediction for many species, especially birds and mammals, in the face of threats such as land use change, disease, abiotic natural phenomena and harvesting or harassment. Conservation strategies designed to improve environmental conditions, increase population growth rates and introduce new individuals (for example, restoring connectivity or performing translocations) may maintain or even increase genetic diversity. Our findings underscore the urgent need for active, genetically informed conservation interventions to halt genetic diversity loss.

## Abstract (web summary)

A comprehensive meta-analysis of global terrestrial and marine genetic diversity covering more than three decades of research demonstrates rapid loss of genetic diversity and identifies conservation interventions that could mitigate this process.

## Main

Biodiversity continues to be lost worldwide at unprecedented rates^7^. International agreements recognize biodiversity at three fundamental levels: ecosystem diversity, species diversity and within-species (intraspecific) genetic diversity (https://www.cbd.int). Intraspecific genetic diversity is critical to individual and population fitness, and thus the long-term survival of populations and species, which ensures ecosystem resilience^8,9^. Maintaining genetic diversity protects biodiversity against future environmental changes^1,10^ and supports nature’s contributions to society^11^. In recognition of its importance, the Convention on Biological Diversity’s Kunming–Montreal Global Biodiversity Framework^12^ now includes targets for safeguarding of genetic diversity of all species^5,6^.

Quantification and prediction of genetic diversity change over time are essential to biodiversity policy prioritization, risk assessment and landscape management^4,12^. Population decline and fragmentation due to anthropogenic factors, such as habitat degradation, unsustainable harvest, invasive species and extreme climatic events^13–16^, lead to genetic erosion^17^ (loss of genome-wide genetic diversity and adaptive potential). Observed genetic diversity loss is therefore both a signal of population decline, and a conservation concern in its own right^4^. Such losses have now been reported across several taxonomic groups^18,19^, and are not exclusive to rare and threatened species^13^. For example, a recent study showed around 6% loss of genetic diversity across populations of 91 animal species over the past century^13^. Theoretical predictions based on the relationship between habitat area and genetic diversity suggest that at least 10% of genetic diversity may have already disappeared in many plant and animal species^20^. Furthermore, even greater losses are predicted on the basis of population genetic theory and the Living Planet Index, unless interventions are taken to halt and reverse species’ population declines^21^.

Although previous research indicates a loss of genetic diversity in specific taxonomic groups and regions^3,22^, there is limited data on the extent and patterns of genetic diversity decline. Furthermore, although there is substantial evidence that individual conservation actions can have important benefits for biodiversity^23,24^, there has been no temporally, spatially and taxonomically comprehensive census of genetic diversity change, alongside information about threats and management action. Although existing molecular genetic datasets can be co-analysed for this purpose (applying macrogenetics^22,25^), this can be challenging^26^, prompting recent calls for greater standardization in genetic diversity reporting^27,28^. Alternatively, a comprehensive and robust assessment of the primary literature, targeting patterns and processes rather than absolute measures of population genetic diversity per se, can be conducted through statistical meta-analysis^29^. By formally combining published genetic diversity measures alongside metadata on threats and conservation actions, we can synthesize knowledge on the variables associated with population genetic diversity change.

**Extended Data Fig. 1:**
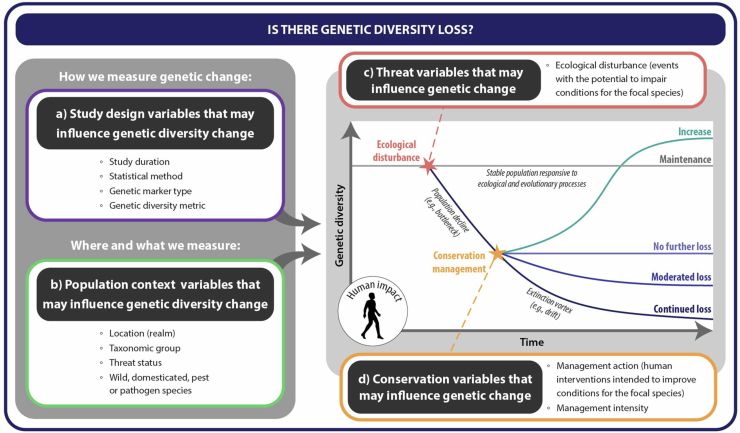
Conceptual diagram describing aims and predictions under different scenarios. Our aim is to determine whether there is overall genetic diversity change, and how this pattern is influenced by: a) study design, b) population context, c) threatening processes, and d) conservation management. When large, stable populations have not encountered or are resilient to disturbance (and thus do not require conservation management), we predict a scenario of ‘*maintenance*’, where no overall genetic diversity change is detected over a timescale relevant to human impact. Alternatively, disturbance may be a negative moderator of genetic change if it results in a loss of genetic diversity (e.g., through a decrease in population size [“bottleneck”]). Following conservation management, we predict three possible outcomes, 1) ‘*increase*’: conservation management reverses loss and genetic diversity returns to initial level (no change detected) or increases beyond initial level (gain detected; management is a positive moderator of genetic diversity change); 2) ‘*no further loss*’ or ‘*moderated loss*’: conservation management halts or mediates loss resulting in a net decline in genetic diversity, but to a lesser degree than if management were absent (i.e., management is a positive moderator of genetic change); or 3) ‘*continued loss*’: conservation management is ineffective or absent, resulting in continued decline (loss detected, e.g., due to genetic drift in small populations). If this trend continues, extinction is inevitable, although the time frame for a given species is hard to predict. Note that the particular timing of an ecological disturbance event or conservation management action in relation to the population’s trajectory is not recorded in our dataset, as we focus only on presence or absence of these drivers. In addition to the processes shown, disturbance can also occasionally increase genetic diversity; such data are included in our analysis, but not illustrated here.

Here we present a global meta-analysis of three decades of published data on genetic diversity change across the eukaryotic tree of life. Using meta-regressions, we quantify associations between ecological disturbance, conservation actions and genetic diversity change. We explore: (1) general patterns of genetic diversity change across varying study designs and population contexts; (2) whether greater losses are found when threats (ecological disturbance) are reported; and (3) whether there is evidence that conservation interventions can moderate (slow, halt or reverse) genetic diversity loss (aims and predictions are presented in Extended Data Fig. 1).

## A global census of genetic diversity change

**Extended Data Fig. 2:**
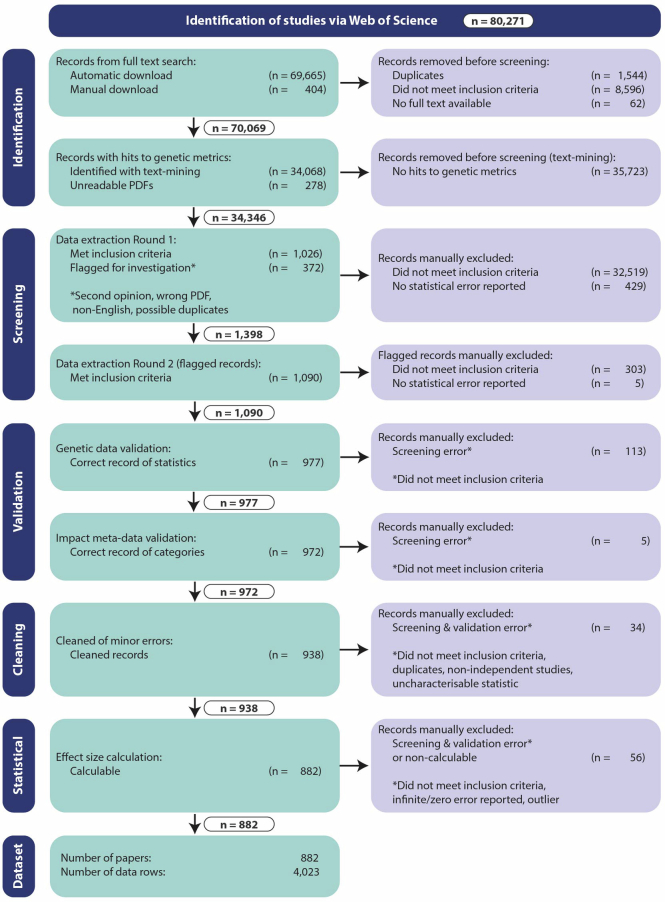
Preferred reporting items for systematic reviews and meta-analyses (PRISMA). Flow diagram detailing the five steps (identification, screening, validation, cleaning and statistical) taken to generate the systematic review dataset, with sample sizes (n) representing the number of papers (note that a further 40 rows of data from 11 papers, representing extreme values, were removed prior to conducting meta-analysis, see Supporting Information 1.4-1.5).

**Extended Data Fig. 3:**
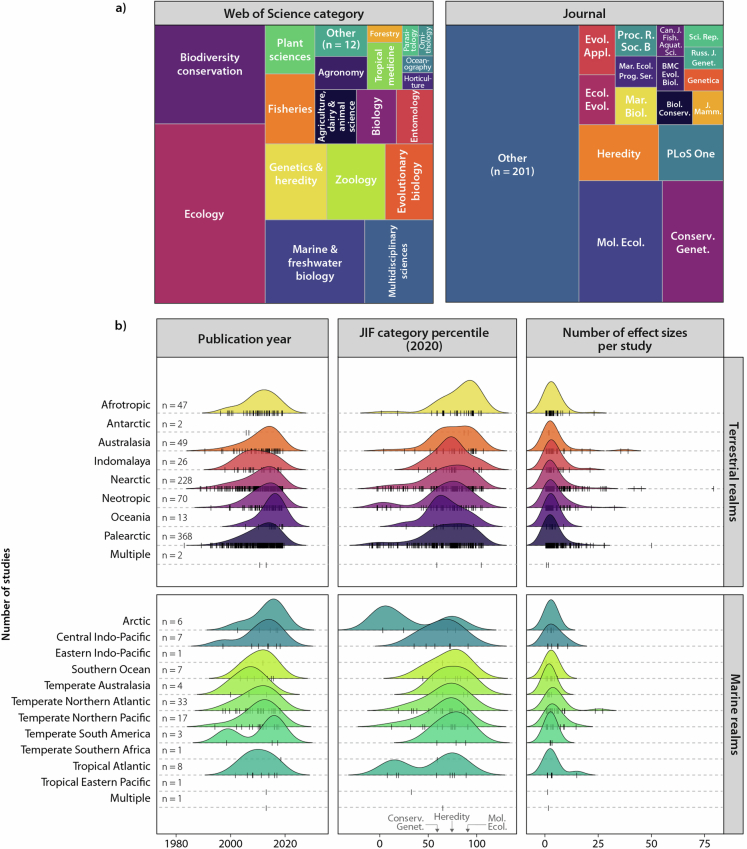
Summary of the systematic review dataset: research effort as reflected by publication trends. a) Tree maps illustrating the number of papers in each Web of Science category and scientific journal (total = 217 journals). The Web of Science category labelled as ‘Other’ provided fewer than 5 journals (left panel). Journals labelled as ‘Other’ provided fewer than 10 papers (right panel). b) Density plots and raw data (vertical ticks along x-axis) represent publication year of each paper, the journal impact factor (JIF) category percentile (three well-known biodiversity genetics journals [*Conservation Genetics*, *Heredity* and *Molecular Ecology*] are presented for context on the x-axis, bottom panel), and the number of effect sizes collected per paper, across terrestrial and marine realms (n = total number of papers). Papers reporting on multiple unique populations in different realms are counted multiple times, whereas the category called ‘multiple’ represents single populations where the distribution spans multiple realms.

Our systematic literature search identified 80,271 records, of which 882 (1.1%) met our inclusion criteria for measuring temporal genetic diversity change (that is, empirical studies of multicellular organisms that report temporal data on genetic diversity over timescales likely to have been impacted by human activities), providing 4,023 measurements for analysis (Extended Data Fig. 2, Supplementary Information 1.1 and Supplementary Data 1–3). Genetic diversity change was measured across a range of geographic regions, time frames and genetic marker types, and encompassed the eukaryotic tree of life. Publication dates spanned 34 years and 217 journals across the expected general fields of ecology, evolution, conservation and genetics, as well as narrow-focus, subject specific fields (Extended Data Fig. 3a,b, Supplementary Information 1.2 and Supplementary Data 1 and 3).

**Fig. 1:**
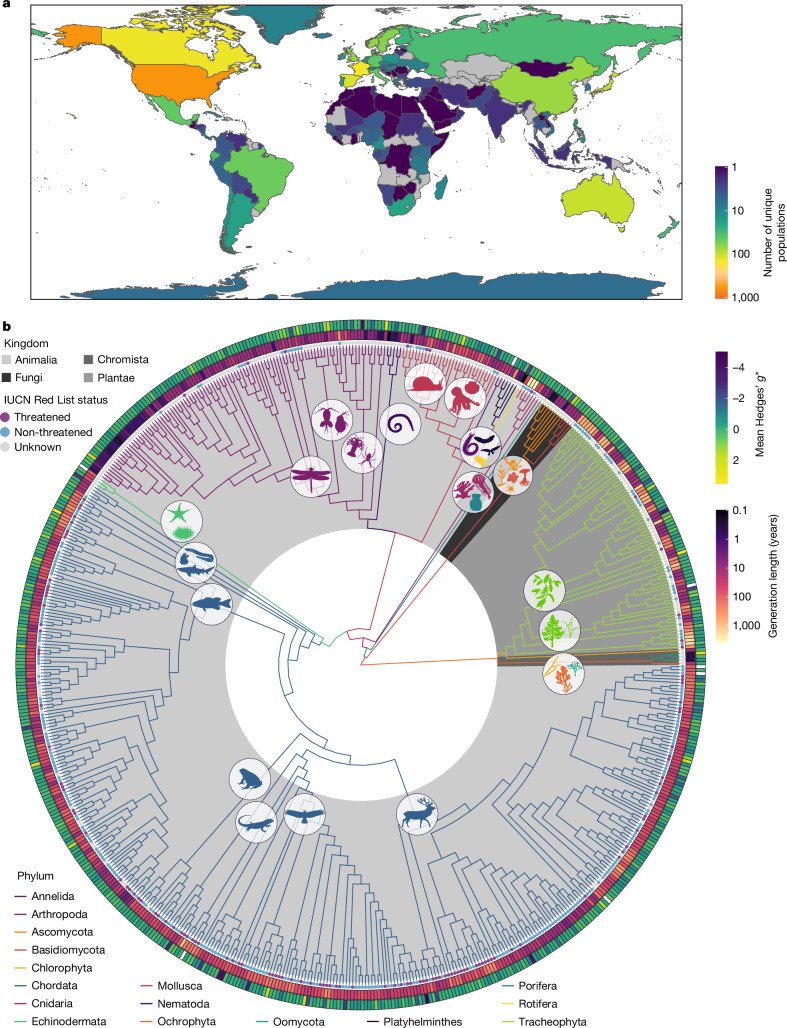
Summary of the systematic review dataset. **a**, World map with colour representing the number of unique populations included (unique species are presented in Extended Data Fig. 4). Grey represents zero counts. Note that both terrestrial and marine realms are represented within the relevant country boundaries, excluding one marine population that could not be reliably linked to a country. Studies spanning country borders are represented multiple times in this figure. World map modified from ref. ^36^. **b**, Visual representation of phylogenetic relationships among taxa, with IUCN Red List threat status, mean effect size (outermost ring; Hedges’ g*; missing data (white) represent extreme values; see Supplementary Information 1.1 and 1.4) and generation length (second outermost ring). In the tree, branch colours represent phyla, and unique classes are represented by silhouettes (coloured by phylum). Silhouettes obtained from PhyloPic (https://www.phylopic.org); image credits in Supplementary Table 15.

**Extended Data Fig. 4:**
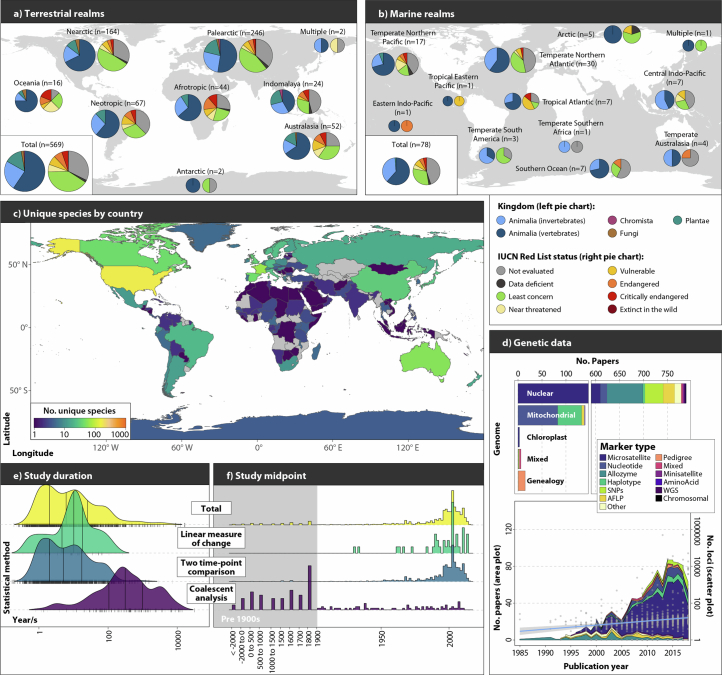
Summary of the systematic review dataset: research effort as reflected by study characteristics. a) The number of unique species (within phyla and IUCN Red List threat status categories) reported from each terrestrial and; b) marine realm (noting that some species occur in both a terrestrial and a marine realm, while those that occur across more than one terrestrial or marine realm are listed as ‘multiple’); c) world map where color represents the number of unique species (whereas unique populations are presented in Fig. 1). Gray represents zero counts. Note that both terrestrial and marine realms are represented within the relevant country boundaries, excluding one marine population that could not be reliably linked to a country. Studies spanning country borders are represented multiple times in this figure. World map modified from ref. ^36^; d) the number of papers reporting each genetic marker type for each genome (top panel, full definitions in Supporting Information 2.6), and an area plot and scatter plot with a regression line (error band is 95% confidence interval) showing the number of papers reporting different genetic marker types (left axis) and the number of loci reported in papers (right axis) across publication years (bottom panel; noting that one paper published in January 2019 was grouped with the 2018 publications); e) temporal characteristics of study duration (in years) and; f) year midpoint (for the total dataset, and across the three main statistical methods identified in our dataset).

Systematic review across 141 countries representing all terrestrial and most marine realms, including 628 species from 37 classes across 16 phyla, provided a field-wide view of how genetic diversity change is measured (Fig. 1a,b, Extended Data Figs. 3b and 4a–f and Supplementary Information 1.2 and 1.3). The vast majority of species studied were animals (84.7%; comprising 59.2% vertebrates and 25.5% invertebrates), followed by plants (12.7%), fungi (1.9%) and chromists (0.6%). Most species were categorized by the International Union for the Conservation of Nature (IUCN) Red List of Threatened Species^30^ as non-threatened (Least Concern, 39.3%; Near Threatened, 6.1%) or having unknown threat status (Data Deficient, 1.8%; Not Evaluated, 33.8%). One-fifth of the species were threatened (Vulnerable, 7.3%; Endangered, 6.7%; Critically Endangered, 4.9%; Extinct, 0.2%) (Fig. 1b, Extended Data Fig. 4a,b and Supplementary Information 1.3). Temporal genetic diversity change was mainly measured across nuclear or mitochondrial genomes (89.5% and 15.9% of studies, respectively) with microsatellite markers being the most common tool (Extended Data Fig. 4d and Supplementary Information 1.3), and estimated over periods of less than 1 year to 12,500 years (mean 111 years, median 6 years), for a median study midpoint of the year 2000 ce (Extended Data Fig. 4e,f and Supplementary Information 1.3).

## Genetic diversity is being lost globally

We investigated patterns of mean genetic diversity change across our dataset via Bayesian hierarchical meta-analysis, in which negative parameter estimates in our study are interpreted as a loss of genetic diversity over time, positive estimates are interpreted as a gain, and estimates close to zero suggest that genetic diversity was constant (maintained) over time. Genetic diversity change was interpreted as statistically significant when 95% highest posterior density (HPD) credible intervals did not overlap zero. For each meta-regression, parameter estimates were also compared to the model intercept (chosen as a biologically or methodologically meaningful reference category).

**Fig. 2:**
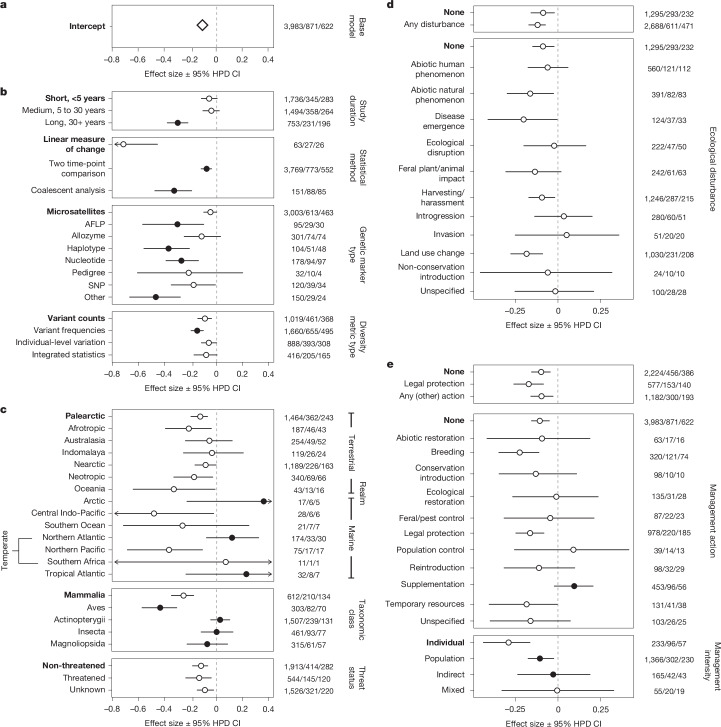
Genetic diversity change across temporal, methodological, geographical, taxonomic, threat and conservation contexts. **a**–**e**, Meta-regression (using the reduced meta-analysis dataset) of predicted genetic diversity change across our entire dataset (base model; **a**) and data subsets investigating associations between genetic diversity change and variables describing study design (**b**), population context (**c**), threats (**d**) and conservation management (**e**). Effect sizes (diamond for ‘overall’ in **a**, circles elsewhere) were measured as Hedges’ g* posterior mean and error bars represent the 95% HPD credible interval (CI). A negative effect size estimate represents a loss of genetic diversity that is statistically significant if the HPD credible intervals do not overlap zero (dashed line); a positive effect size estimate represents a gain in genetic diversity that is statistically significant if the HPD credible intervals do not overlap zero. Arrows denote 95% HPD credible intervals that extend beyond axis limits. Filled circles represent predictors that are significantly different from the intercept at *α* = 0.05, with the intercept for each meta-regression indicated in bold text. Numbers on the right represent sample sizes, presented as number of effect sizes/papers/species. Estimates for generation and study midpoint (included as fixed effects in all models) are provided in Supplementary Information 1.5–1.9.

After sensitivity testing (Methods and Supplementary Information 1.4 and 1.5), our reduced meta-analysis dataset comprised 871 published records, providing 3,983 Hedges’ g* effect sizes for modelling, encompassing 622 species from 36 classes across 16 phyla. Meta-analysis over this entire dataset revealed a small, but statistically significant loss of genetic diversity over time (Hedges’ g* posterior mean = −0.11; 95% HPD credible interval −0.15, −0.07) (Fig. 2a and Supplementary Information 1.4 and 1.5). No publication bias was detected (Supplementary Information 1.5). In a few cases, extreme genetic diversity change was observed, which had detectable influence on the results; therefore, such cases were removed so that our model outputs represented the general trends present across 99% of our dataset (extreme genetic diversity changes are narrated at Supplementary Information 1.4).

Using meta-regressions, we consistently found a mean loss of genetic diversity regardless of study duration, statistical method, genetic marker type or genetic diversity metric used (Fig. 2b and Supplementary Information 1.6). The magnitude of loss varied, with greater losses detected: (1) when temporal comparisons were conducted over a long time frame (30 or more years; despite controlling for the focal species’ generation length); (2) when measures were derived from linear statistical measurements (such as regression) versus comparisons of two time points or coalescent analyses; (3) when using AFLP (amplified fragment length polymorphism), haplotype, nucleotide and other data types versus microsatellite markers; and (4) when using population-level genetic diversity metrics that incorporate variant frequencies (for example, expected heterozygosity or nucleotide diversity) versus other genetic diversity metrics (Fig. 2b and Supplementary Information 1.6). Where studies reported multiple genetic diversity metrics, effect sizes were weakly or moderately correlated (*r* = 0.25–0.55) (Supplementary Information 1.6), suggesting that the four diversity metric types we used (variant counts, variant frequencies, individual-level diversity and effective population size; Methods) capture somewhat independent information about genetic diversity change.

From a biogeographical perspective, meta-regression showed that genetic diversity loss was observed across most terrestrial realms, which comprised a vast majority of the data (90.2%), whereas results across marine realms were more variable, albeit estimated from a small number of studies (Fig. 2c and Supplementary Information 1.7). Relative to the Palaearctic, the Arctic, Temperate Northern Atlantic and Tropical Atlantic marine realms showed significantly less genetic diversity loss, with positive parameter estimates (Fig. 2c and Supplementary Information 1.7).

From a broad evolutionary perspective, common ancestry (phylogeny) explained only a small percentage of variance in effect sizes across the dataset (3.79%) (Supplementary Information 1.5). Although patterns of genetic diversity change were not well correlated with ancestry relationships, variation was seen at the class taxonomic rank: of the five classes with the most data, the greatest loss of genetic diversity was observed in Aves (birds; predicted Hedges’ g* posterior mean = −0.43; 95% HPD credible interval −0.57, −0.30), followed by Mammalia (mammals; Hedges’ g* posterior mean = −0.25; 95% HPD credible interval −0.35, −0.17) (Fig. 2c). However, relative to Mammalia, three taxonomic classes—Magnoliopsida (dicotyledonous plants), Insecta (insects) and Actinopterygii (ray-finned fishes)—showed significantly less loss and no significant mean genetic diversity change, suggesting that, on average, genetic diversity was maintained over time in these three taxonomic classes (Fig. 2c and Supplementary Information 1.7).

**Extended Data Fig. 5:**
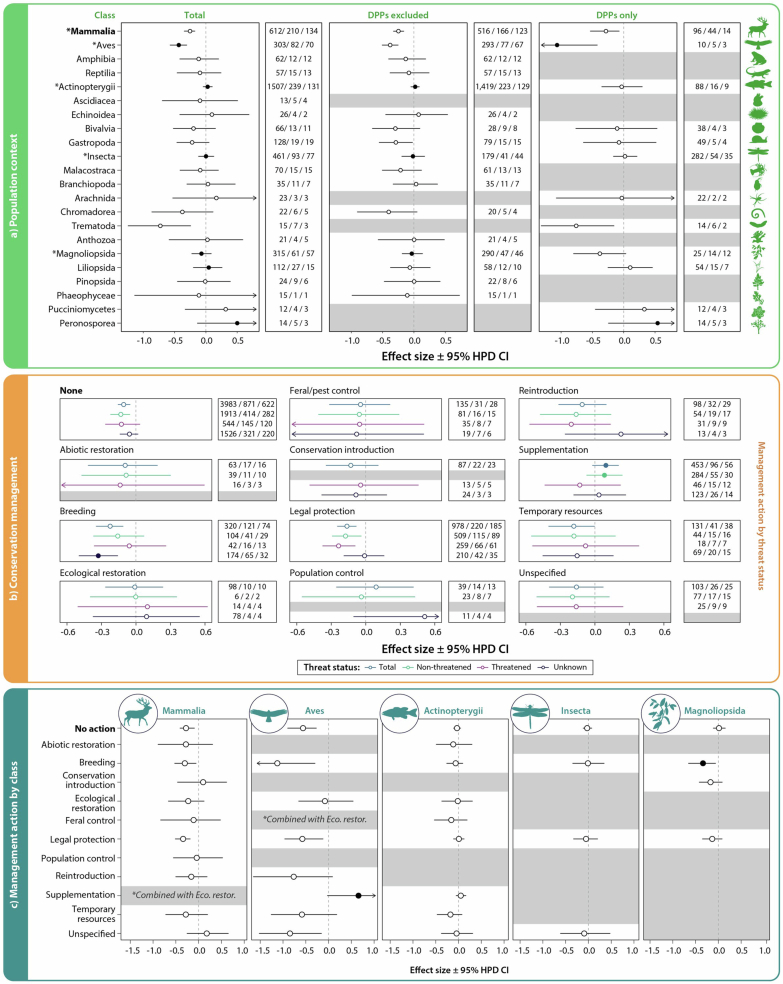
Genetic diversity change across population and conservation contexts. Meta-regressions (using the reduced meta-analysis dataset) of predicted change in genetic diversity for: a) species in each taxonomic class, for the total dataset (left), excluding domestic, pest or pathogen populations (“DPPs”; middle), and for domestic, pest or pathogen populations only (right), presented in order of phylogeny and including the five most data-rich classes (asterisks) that are also presented in Fig. 2; b) conservation management actions for the total dataset (blue, also presented in Fig. 2); non-threatened species (green); threatened species (light purple); and unknown threat status species (dark purple); c) conservation management action across the five most data-rich taxonomic classes, where asterisks indicate correlated actions that were combined for certain taxa. For all meta-regressions, effect sizes (circles) were measured as mean Hedges’ g* and error bars are the 95% highest posterior density credible intervals (HPD CIs). A negative effect size estimate represents a loss of genetic diversity and a positive effect size estimate represents a gain in genetic diversity, statistically significant if the HPD CIs do not overlap zero (dashed line). Arrows denote 95% HPD CIs that extend beyond axis limits. Filled circles represent predictors that are statistically significantly different from the intercept at *α* = 0.05, with the intercept indicated in bold text. Boxes to the right of forest plots provide sample sizes (presented as number of effect sizes / papers / species; see Supporting Information 1.9 for panel c sample sizes). Gray panels indicate variables that were excluded due to insufficient data for modelling (< 10 effect sizes). Estimates for generation and study midpoint (also included as fixed effects) can be found in Supporting Information 1.7 and 1.9. Organism silhouettes obtained from PhyloPic (www.phylopic.org), see Supporting Information 2.6 for image credits.

Demographic history prior to a temporal genetic study may plausibly affect our ability to detect further genetic diversity change. For those populations that were likely to have faced species-level threats and/or declines, as identified by IUCN Red List threat status^30^, meta-regression showed that genetic diversity loss occurred regardless of whether a focal species was threatened, non-threatened, or had unknown threat status (Fig. 2c and Supplementary Information 1.7). We further re-examined our main findings with and without a subset of studies focused on populations identified as ‘domestic, pest or pathogen’ (Extended Data Fig. 5a and Supplementary Information 1.7). Greater genetic diversity losses were detected in Aves and Magnoliopsida populations in our domestic, pest or pathogen data subset, with Aves representing significantly greater genetic diversity loss relative to the model reference category, Mammalia (Extended Data Fig. 5a).

## Disturbance is more common than management

**Extended Data Fig. 6:**
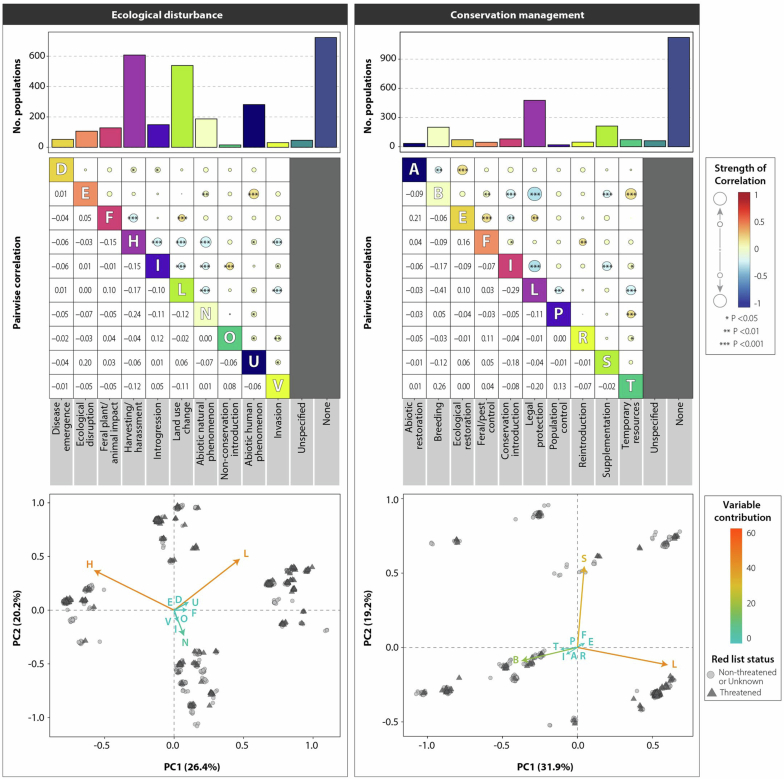
Summary of the systematic review dataset; summary statistics for impact meta-data. Ecological disturbance (left) and conservation management action (right) reported for unique populations of species within each study, including top panel: bar charts of total counts (i.e. sample size) per disturbance or action type; middle panel: correlations between disturbance or action types (lower half = correlation coefficient, upper half = strength of correlation represented by color and size, with asterisks showing statistical significance [two-sided test of H_0_ = 0, no correction made for multiple testing]); bottom panel: PCA biplots, where letters represent ecological disturbance or conservation management action (as defined in correlation plot above), point colour and shape represent IUCN threat status (light gray circles = non-threatened or unknown, dark gray triangles = threatened), and arrow and letter colour represents the variable contribution to the PCA.

We developed and applied a protocol to categorize threats to populations (ecological disturbance, including intentional or unintentional anthropogenic events and extreme natural events; described in Extended Data Table 1), as well as conservation management actions (described in Extended Data Table 2), to quantify their effects on genetic diversity change. For those variables with sufficient data for meta-regression (Supplementary Information 1.8 and 1.9), ten types of ecological disturbance showed negligible correlations (*r* ≤ |0.24|), as did ten types of conservation action (*r* ≤ |0.25|), with the exception of a weak negative correlation between legal protection and breeding management (*r* = −0.41) (Extended Data Fig. 6 and Supplementary Information 1.10 and 1.11), suggesting that overall our categorizations provide largely independent information about threats and management.

Within the temporal time frame of studies, at least one type of ecological disturbance or conservation management action was reported for 65.11% or 45.75% of the unique populations, respectively, in our systematic review dataset, with 35.35% reporting both. For ecological disturbances, harvesting or harassment (harvesting/harassment) of the focal species was the most commonly reported disturbance (29.34%), followed by land use change (26.01%) and abiotic human phenomenon (13.56%) (Extended Data Fig. 6 and Supplementary Information 1.10). For conservation management action, legal protection (23.02%) was the most commonly reported action, followed by supplementation (adding individuals to an existing population) (10.28%) and breeding management (9.70%) (Extended Data Fig. 6 and Supplementary Information 1.11). Ecological disturbances and conservation actions occurred more commonly for threatened species (82.33% and 66.78%, respectively) compared with the non-threatened, and Data Deficient and Not Evaluated species (62.38% and 42.38%, respectively).

**Fig. 3:**
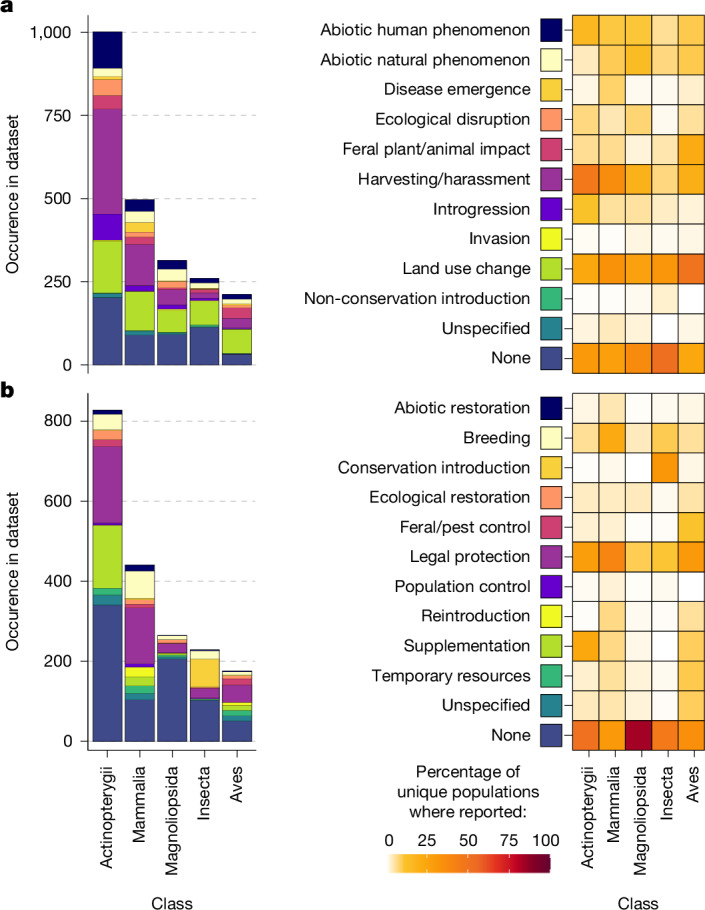
Summary of threats and management. **a**,**b**, Stacked bar charts showing total counts (sample size) and heat maps showing the percentage of unique populations of species for which the different types of ecological disturbance (**a**) and conservation management actions (**b**) were reported (definitions in Extended Data Tables 1 and 2 and Supplementary Information 2.5), for the five most data-rich taxonomic classes (the remaining classes are presented in Extended Data Fig. 6). Coloured squares to the right of the disturbance type and action labels indicate the colour used to represent the disturbances and actions in the bar charts.

**Extended Data Fig. 7:**
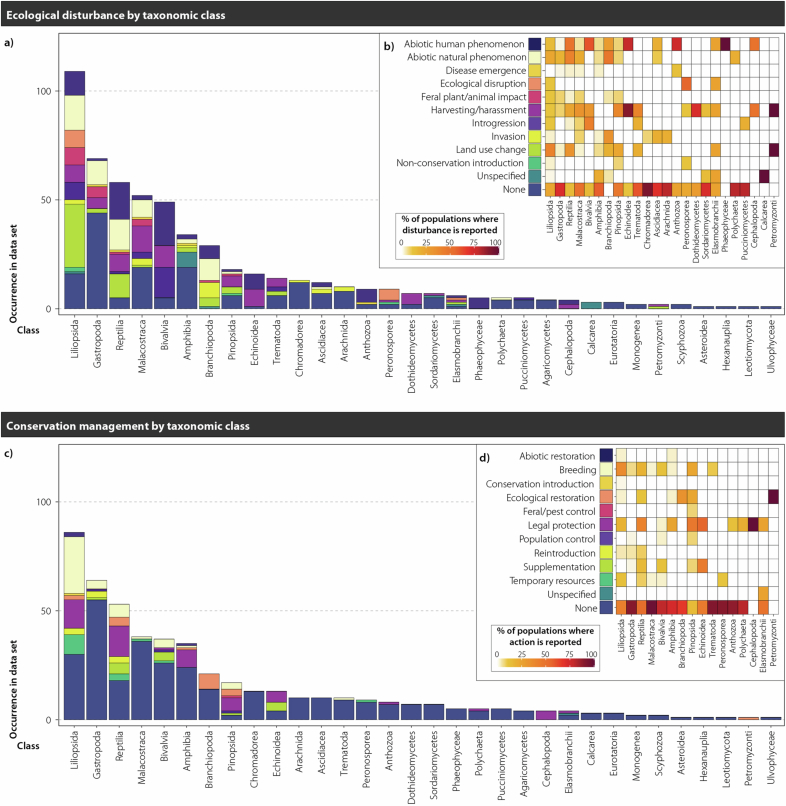
Summary of the systematic review dataset; impact meta-data by selected taxonomic classes. Ecological disturbance (a-b) and conservation management action (c-d) reported for unique populations of species within each study, including stacked bar charts of total counts (i.e., sample size) of the occurrence of each disturbance/action within each taxonomic class (classes are sorted by the amount of data), and heat maps showing the percentage of total unique populations within each taxonomic class for which the different disturbances/actions were reported. The top five most data-rich taxonomic classes (Mammalia, Aves, Actinopterygii, Insecta, Magnoliopsida) are instead presented in Fig. 3, while classes where no action was recorded across all populations are excluded from visualization in heat maps. Colored squares to the right of the disturbance types (b) and conservation actions (d) indicate the color used to represent the disturbances/actions in the bar charts (a, c).

When comparing threatened versus non-threatened species, there were no clear trends in the types of ecological disturbance or conservation management action reported (Extended Data Fig. 6 and Supplementary Information 1.10 and 1.11). However, among taxonomic classes, ecological disturbance and conservation actions varied (Fig. 3a,b, Extended Data Fig. 7 and Supplementary Information 1.10 and 1.11). For example, harvesting/harassment, followed by land use change, were the most reported disturbances for Mammalia and Actinopterygii, with land use change ranked as the most common for Aves, Insecta and Magnoliopsida (Fig. 3a). For conservation action, other than legal protection (the most common action reported), the next most common action for Actinopterygii was supplementation. For Mammalia, the second most common action was breeding. For Insecta, breeding and ecological restoration were equal second most common actions. For Magnoliopsida, the second most common action was conservation introduction, and for Aves it was control of feral and pest species (Fig. 3b and Supplementary Information 1.11).

## Actions to maintain genetic diversity

Using meta-regression, we explored genetic diversity change in studies that reported ecological disturbance compared with studies in which no disturbance was reported during the time period examined. A statistically significant mean loss of genetic diversity occurred even when no disturbance was reported, suggesting a background level of genetic diversity loss across species (Fig. 2d and Supplementary Information 1.8). When disturbance types were considered individually, statistically significant genetic diversity loss was detected alongside abiotic natural disturbances (for example, wildfire), disease emergence, harvesting/harassment and land use change, although these estimates were not statistically different from background loss (that is, they did not differ from the model intercept, which represents no reported disturbance) (Fig. 2d and Supplementary Information 1.8).

We also explored genetic diversity change in studies that reported conservation management actions during the course of the study compared with the absence of such action. Consistent with the background loss identified in our analysis of ecological disturbance, statistically significant mean loss of genetic diversity occurred in the absence of conservation actions (Fig. 2e and Supplementary Information 1.9). As legal protection alone does not involve active ecological intervention (although it may mandate it, in which case we recorded those actions if reported), we considered the effects of this action compared to all others, and found statistically significant mean loss of genetic diversity, similar to background loss (Fig. 2e, Extended Data Fig. 5b and Supplementary Information 1.9). When conservation management actions were considered individually, statistically significant genetic diversity loss was detected alongside reports of breeding, legal protection and/or temporary resources (for example, supplementary feeding), across all species and regardless of threat status (Fig. 2e, Extended Data Fig. 5b and Supplementary Information 1.9). This is not surprising, given that conservation management actions primarily target at-risk populations that may already be in decline, and such decline can result in loss of genetic diversity^31^. Even if conservation management actions succeed in slowing, halting or reversing genetic diversity decline, a net genetic diversity loss may still be recorded (Extended Data Fig. 1). By contrast, mean estimates for genetic diversity change were close to zero or positive when reported alongside ecological restoration, feral and pest control, population control and supplementation, suggesting that, on average, genetic diversity was maintained or increased across temporal comparisons. Supplementation was a statistically significant moderator of genetic diversity loss, and was the only conservation action associated with a significant increase in genetic diversity compared with cases in which no action was reported, especially in birds (Fig. 2e and Extended Data Fig. 5c). The positive effect of supplementation was observed in non-threatened species, but not in species that were threatened or had unknown threat status (Extended Data Fig. 5b and Supplementary Information 1.9). Considering conservation actions for the five most data-rich classes, loss of genetic diversity was observed in the absence of conservation action for Mammalia and Aves, but not for Actinopterygii, Insecta or Magnoliopsida (Extended Data Fig. 5c and Supplementary Information 1.9).

We classified conservation interventions into three levels of management intensity—namely actions that target individuals, populations or landscapes. The greatest loss of genetic diversity was associated with reports of the highest management intensity (that is, management at the individual level) (Fig. 2e and Supplementary Information 1.9). Compared with individual-level conservation management, significantly less loss was observed alongside studies reporting population-level management (for example, habitat restoration), but there was still an overall net loss of genetic diversity. Contexts associated with indirect management (for example, management targeting other species in the same habitat) showed no mean genetic diversity change, but this estimate had low precision (Fig. 2e and Supplementary Information 1.9).

## Discussion

Here we report an overall global decline in intraspecific genetic diversity. Our study provides the most comprehensive investigation of within-population genetic diversity change to date, transcending taxonomic and geographic boundaries, and the a priori objectives, predictions and methods of individual biological research reports. In birds and mammals in particular, the evidence for genetic diversity decline is clear. In other taxa, for which we had sufficient data (dicotyledonous plants, insects and ray-finned fishes), genetic diversity was maintained over time. However, these taxonomic groups may still be at risk, as genetic diversity losses are not always easily detected (Extended Data Fig. 1) or may lag behind demographic changes^32^. Declines in census sizes of species with massive populations or very long-lived species might not lead to measurable losses of genetic diversity over the timescales studied. Our finding of significant losses of genetic diversity across short study periods (on average) for several taxonomic classes, representing 207 species (with even more trending negative, although non-significant), indicates that the population size declines underlying these genetic diversity losses are likely to be considerable. This pattern carries two key implications: (1) further genetic diversity loss in the near term is likely if human societies do not take action urgently; and (2) we currently have sensitive methods and datasets for detection of genetic diversity change, which enable us to target biodiversity conservation actions effectively.

Most of the unique populations in our dataset were reportedly affected by disturbances within the time frame of the study, suggesting that anthropogenic activities are direct and widespread hazards, affecting not only diversity among species^3,33^, but also genetic diversity within species. For these reasons, even the subtle negative trends of genetic diversity change that we report here should raise concern over the resilience of populations and the capacity for natural ecosystems to sustain vital ecosystem services^11^, and should therefore trigger intensified conservation management actions to halt genetic erosion before further losses occur. Genetic diversity accumulates over evolutionary timescales through mutation and once lost, is difficult to restore^34^. Supplementation (that is, the addition of individuals to a population, including genetic or demographic rescue through restoring connectivity or performing translocations) was the only conservation management action associated with a statistically significant mean increase in genetic diversity over time relative to cases where no actions were reported. In addition, we found that other conservation actions designed to improve environmental conditions and increase population growth rates may halt or reduce further genetic diversity loss and therefore safeguard it. We have four recommendations to track within-population genetic diversity and determine when and where conservation actions may be needed:

1. *Conduct temporal genetic monitoring*. Genetic diversity metrics are sensitive to change, particularly over long-term studies. Monitoring genetic diversity alongside threats and conservation action can inform strategic management.
2. *Where temporal genetic data do not exist, start collecting now*. Although multiple-timepoint sampling informs change, single snapshots of genetic diversity are invaluable for tailoring management decisions and provide a point of comparison for future sampling.
3. *Where genetic data collection is difficult, utilize existing data*. Here we have identified hundreds of datasets as a starting point for informing genetic management to expand upon in the future.
4. *Where genetic data are absent, use proxies*. Genetic considerations should inform any biodiversity risk assessment, even if based solely on other data types, such as field observations of population size^35^.

Our analysis demonstrates that genetic diversity loss is a realistic general expectation for many species around the world. However, we also show that we have the theoretical and technical means, as well as the on-ground conservation management approaches, to prevent further loss if we act now.

## Methods

### Compilation of genetic diversity change measures

We conducted a literature search of peer-reviewed publications using the Web of Science (WOS) advanced search functions to find published works that contained temporal measures of genetic diversity. Our search was intentionally broad and followed established preferred reporting items for systematic reviews and meta-analysis (PRISMA) protocols as closely as possible^37,38^. The online search was conducted on 18 January 2019 using keywords targeting population genetic measurements, regardless of the direction of change (for example, ‘increase’ and ‘decrease’ were both included as search terms) (Supplementary Information 2.1 and Supplementary Data 4). A total of 80,271 records were retrieved, 78,727 after duplicate removal. We obtained full texts for 70,069 of these records. The remaining records were screened manually via their titles, abstracts and keywords, of which 8,596 were excluded against our inclusion criteria; 62 full texts could not be obtained (Extended Data Fig. 2 and Supplementary Information 2.1). We then performed full text mining in R v.3.5.2^39^, using the packages pdfsearch v.0.2.3^40^, dplyr v.0.8.0^41^, and stringi v.1.3.1^42^ to remove records that did not contain population genetic keywords (Supplementary Information 2.2). This resulted in 34,346 putatively includable studies of genetic diversity change, which were classified into thematic clusters using the package revtools v.0.4.0^43^ (Supplementary Information 2.2).

We performed initial screening and data extraction for all 34,346 works, followed by a series of re-extraction and data validation steps. Manual screening of studies against inclusion criteria, and extraction of genetic diversity measurements and metadata took place simultaneously by members of the authorship team, via multiple workshops using shared written guidelines (Extended Data Tables 1 and 2 and Supplementary Information 2.3–2.6). Studies were suitable for inclusion in our analysis only if they satisfied all of the following criteria:

- The research must report primary, quantitative, empirical data from a multicellular nonhuman organism.
- Laboratory and experimentally manipulated populations were excluded, where these experimental manipulations were for the purpose of testing a hypothesis related to population demography or genetics (note that populations established in controlled conditions for supportive breeding or propagation were potentially includable, such as ‘captive’ or ‘agricultural’ populations),
- The time frame of the study plausibly took place over timescales likely to have been impacted by human activities, regardless of whether the study organism was actually impacted by human activities (in general, we targeted genetic diversity changes in the last few hundred years and excluded studies on ancient admixture or expansion in response to events on ‘geological’ timescales; further detail in Supplementary Information 2.4),
- The study design enabled a temporal comparison of population genetic measurements (for example, samples collected over multiple years) or inference (for example, coalescent genetic studies),
- The study reports a quantitative measurement of within-population ‘genetic diversity’ (broadly defined), and an associated measurement error and sample size. Genetic diversity statistics were obtained from main texts (including tables and figures) and supplementary materials, but no re-analysis of published datasets was conducted. Summary statistics (mean and s.d.) were calculated from tabulated data where available.

In addition to recording bibliographic data for each record, we extracted data that would enable us to calculate our effect sizes (see below and Supplementary Information 2.4), as well as corresponding metadata for meta-regression (Extended Data Tables 1 and 2 and Supplementary Information 2.5 and 2.6). Our dataset included many studies for which the main goal was not an assessment of genetic diversity or change in genetic diversity per se, but which nevertheless reported temporal measures of genetic diversity that otherwise met the inclusion criteria for our meta-analysis.

We captured genetic diversity statistics aligned with three possible study designs (see Supplementary Information 2.4): (1) linear measure of change (for example, regression of two or more time points), yielding primarily statistical measurements, such as regression coefficients or *t* statistics; (2) two timepoint comparison (for example, comparison of two means), yielding primarily pairs of mean diversity estimates; or (3) coalescent analysis, yielding either statistical or genetic measurements, obtained by probabilistic modelling of past effective population sizes using a single sample in time^44,45^. The latter were uniquely identified in our analysis due to important differences in the underpinning theoretical framework for coalescent analyses. That is, ‘early’ measures of genetic diversity are not taken from real biological samples, but instead inferred from recent data and principles of genetic inheritance. Further, early time points in coalescent analyses may be identified by authors a priori based on environmental or other non-genetic hypotheses, or post hoc on the basis of substantive patterns in the data.

Genetic diversity change data were recorded alongside corresponding error estimates and sample sizes; all were extracted using the same level of precision as reported in the paper. We also recorded the time frame of the study (early and recent years, used to calculate study duration and study midpoint), plus amount and type of genetic data used (for example, number of loci, genetic marker type, genome). We classified genetic diversity change data into four metric types, aligned with ‘essential biodiversity variables’ for genetic composition^28^: (1) variant counts (for example, allelic richness); (2) evenness of variant frequencies (for example, expected heterozygosity and nucleotide diversity); (3) population means of individual-level variation (for example, observed heterozygosity and pedigree inbreeding); and (4) integrated statistics (for example, effective population size; see below).

We were particularly interested in associations between genetic diversity change and ecological disturbance or conservation management action, so these ‘impact metadata’ were collected where threats and/or conservation management actions were reported in a paper as plausibly impacting the study population between the sampling time points of the study. In principle, we categorized ecological disturbances as events with potential to impair conditions for the focal species or its habitat, and conservation management actions as human activities intended to improve conditions for the focal species or its habitat. For the latter, we also considered the intensity of conservation management actions (that is, the magnitude of conservation intervention as probably experienced by the focal species). We also collected additional metadata, as the objectives of ecological disturbances are likely to vary across species. For example, disturbance is often intentional for pests and pathogens (for example, population reduction), whereas disturbance of threatened species can include indirect or unintentional consequences of human activity (for example, habitat loss and fragmentation). Brief definitions of the categories that we used for each of these variables are in Extended Data Table 1 and 2 and full definitions are provided in Supplementary Information 2.5.

Additional moderators that were collected included (Supplementary Information 2.6): taxonomic identity of study species (nomenclature standardized by literature review to align with Open Tree of Life^46^), country and terrestrial and/or marine realm of the locality where samples were collected, following refs. ^36,47,48^, along with unique site identifiers in the case of multiple populations reported in a publication. We also collected the threat status of the study species^30^; generation length of the study species (Supplementary Data 5); and classification of domesticated species or populations considered as pathogens or pests (based on description in the source publication, relevant databases or other published sources).

Many studies reported multiple measurements of genetic diversity that were suitable for inclusion in our analysis. We extracted independent measures of genetic diversity change per publication taking into consideration the sampling scheme of the reported study and analysis of data subsets (Supplementary Information 2.4). Procedures for controlling non-independent data, missing data, infinite confidence intervals (in estimates of effective population size), zero variances, and unconventional study designs are described at Supplementary Information 2.7–2.9. After initial extraction, all included studies were re-processed by at least two additional members of a small validation group from the authorship team, to ensure consistency in the collection of genetic and metadata (Supplementary Information 2.10).

### Systematic review

Full bibliographic details for each included study were automatically downloaded from the WOS during the original search (see also Supplementary Data 1–3). Additional bibliographic metadata were also collected from the WOS, including journal title abbreviations, WOS subject categories for which each journal ranked highest, and the impact factor percentile ranking for each journal within its WOS category for 2020. Publication trends and the characteristics of studies included in our final dataset were summarized visually using the R packages ggplot2 v.3.4.3^49^, treemapify v.2.5.5^50^ and ggridges v.0.5.4^51^. We also explored patterns of co-occurrence and characterized the variation of ecological disturbance and conservation management actions reported across our full dataset, and data subsets of the five most data-rich taxonomic classes. We calculated pairwise Spearman’s rank correlation coefficients (*r*) among ecological disturbance categories, and conservation management actions, and visualized results in R using corrplot v.0.92^52^. We also examined relationships among these variables using principal component analysis, visualized with the package factoextra v.1.0.7^53^.

### Phylogeny

To visualize the taxonomic diversity of species in our dataset and their evolutionary relationships, we generated a phylogenetic tree using the R package rotl v.3.0.12^54^ using Open Tree of Life IDs as described above. *Saccharomyces cf. cerevisiae* (ott id 7511391) was used as the outgroup. Six species could not be placed in the phylogeny due to unresolved taxonomy: the Japanese mud snail (*Batillaria attramentaria*), white seabream (*Diplodus sargus*), a fruit fly (*Drosophila pseudoobscura*), a sea snail (*Euparthenia bulinea*), fourfinger threadfin (*Eleutheronema tetradactylum*) and the bicolour damselfish (*Stegastes partitus*). The phylogeny was visualized using the R packages ggtree v.3.8.2^55^, ggtreeExtra v.1.10.0^56^, ggimage v.0.3.3^57^ and rphylopic v.1.2.1^58^. Silhouettes of representative organisms for each taxonomic class were downloaded from PhyloPic (https://www.phylopic.org; see Supplementary Information 2.6 for credits). Owing to the taxonomic diversity of species in our study, obtaining a dated tree across all species was not possible and so the topology of the tree was used in modelling.

### Effect size extraction and calculation

For each comparison that satisfied our inclusion criteria, we calculated Hedges’ g* (sometimes referred to as Hedges’ *d*^59^ with sample size correction *J*) as our measure of effect size. Hedges’ g* was selected as the effect size measure as it is based on the standardized mean difference between two values, in our case the ‘early’ and ‘recent’ time points, minimizes over-inflation of effect size estimation in studies with sample sizes <20, and outperforms other common effect size measures such as Cohen’s *d* and Glass’ *Δ* when the assumption of homogeneity of variance is violated^60^. All formulae used to evaluate Hedges’ g* and its error are reported in Supplementary Information 2.11.

Calculation of Hedges’ g* requires the sample size and error associated with the measure of genetic diversity change. Depending on the way in which genetic diversity metrics or their summary statistics are calculated, the associated sample size for effect size calculation may be for example, the number of loci, the number of samples, the number of populations or a rarefied sample size. For comparisons based on linear measures of genetic diversity change, which varied in the methods used to determine genetic change, each paper was manually checked to retrieve the appropriate sample size and error. For two timepoint comparisons and comparisons based on coalescent analyses, we followed a hierarchical procedure to establish the sample size to use for each effect size (Supplementary Information 2.11). Multiple error types were reported (for example, s.d. or confidence intervals), and so Hedges’ g* was calculated using published formulae for interconversion of these data types (Supplementary Information 2.11).

For comparisons where an effect size was calculated, the direction of the effect was determined. We ensured consistent directionality among the following measures of genetic diversity (note that many metrics were recorded in our dataset, and so the abbreviations reported below are summaries only):

1. Variant counts were all positively associated with genetic diversity: mean of alleles across loci (*A*), standardized by sample size (*A*_R_), sum of alleles across loci (*T*_A_), total number of private alleles (pA) and number of polymorphic loci (NPL).
2. Variant frequencies:
  - Positive: expected heterozygosity (*H*_E_), nucleotide diversity (*π*), haplotype diversity (*h*), Shannon diversity index (*H*), polymorphic information content (PIC), number of effective alleles (NEA), frequency of an allele of interest (Freq) and mean individual nucleotide p-distance (NPD, occasionally seen in major histocompatibility complex and similar studies).
  - Negative: population-level inbreeding coefficient or selfing/outcrossing rate (*F*_IS_), mean relatedness or kinship among individuals (*R*), band sharing score (BS) and among-population *F*_ST_ (apF_ST_).
3. Individual-level diversity measures:
  - Positive: observed heterozygosity (*H*_O_), standardized observed heterozygosity (SH) and mean number of alleles per individual (*A*_i_).
  - Negative: individual-level inbreeding coefficient or coancestry (*F*).
4. Integrated statistics were all positively correlated with genetic diversity: effective population size (*N*_e_), effective number of breeders (*N*_b_), female effective population size (*N*_f_), effective population size estimated from demographic data (*N*_d_), effective population size estimated from a population census, and calculated based on an assumption about the ratio between effective and census population sizes (*N*_c_).

For comparisons where genetic diversity metric type was recorded as ‘other’, each paper was manually checked to determine the correct direction of the effect given the context of the metric within the publication and the authors’ interpretation of genetic diversity change as a loss or gain. For negatively correlated metrics, we multiplied the Hedges’ g* effect size by −1 to reverse the direction of the effect—that is, across our dataset a positive Hedges’ g* represents an increase in genetic diversity and a negative Hedges’ g* represents a loss of genetic diversity.

All calculated effect sizes >|4| were manually examined as potential outliers by a single member of the research team, to confirm absence of data entry errors. Considering our wide diversity of statistics, these data were also checked for possible calculation errors, misinterpretation of statistical error (for example, standard error versus s.d.), or other discrepancies. Results of screening of extreme values can be found in Supplementary Information 1.4.

### Meta-analysis

We fitted multi-level Bayesian hierarchical models in the R package MCMCglmm v.2.34^61^, with paper ID as a random effect for all models to account for non-independence introduced by studies that report multiple, includable effect sizes. Genetic diversity change was modelled per generation by including the *z*-standardized number of generations for that species (that is, number of years passed between the early and recent time points, divided by generation length) as a fixed effect in all meta-regressions. Unless otherwise stated in Supplementary Information 2.12, all meta-regressions also included a fixed effect of the *z*-standardized study midpoint (year).

Each model was run for 6,000,000 iterations, with a burn-in of 200,000 and a thinning interval of 5,000, using the weakly informative inverse-gamma prior. We report the posterior mean and the 95% HPD credible intervals for each model set. Estimates with a 95% HPD credible interval excluding zero were considered statistically significant at *α* = 0.05. Model diagnostics were visually checked for no pattern in the trace plots; effective size >1,000 and autocorrelation <0.1 between lag points were both checked using coda v.0.19.4^62^. Chain convergence was confirmed by passing the Heidelberger and Welch’s half-width and stationarity tests in coda. Additionally, each model was independently run three times to calculate a Gelman-Rubin convergence diagnostic of <1.1 using the potential scale reduction factor. The deviance information criterion (DIC) was obtained for each of the three models, and the model with the lowest DIC was selected for interpretation.

Our base model included fixed and random effects described above (that is, fixed effects = *z*-standardized midpoint, *z*-standardized number of generations; random effect = paper ID), although variations of this model underwent sensitivity testing to determine the influence of including phylogeny as an additional random effect, and including extreme values in the dataset (described in Supplementary Information 2.11). The extended heterogeneity statistic^63^ was calculated for both the base model and the sensitivity testing model that included phylogeny. Extended heterogeneity statistics partition total heterogeneity (*I*^2^_total_) into phylogenetic variance (in the phylogenetic model, *I*^2^_phylogeny_), study ID variance (*I*^2^_study_) and residual variance^63,64^ (*I*^2^_residual_). For the phylogenetic model, we also obtained lambda (phylogenetic signal (*H*^2^)) as the variance of the random effect of phylogeny divided by the total variance of all random effects (phylogeny, study ID and residual variance). Total heterogeneity was high, but phylogenetic signal only explained 5.48% of overall variance, so was excluded from further modelling (see also Supplementary Information 1.5); this also allowed for simplification of the model structure. Based on the results of the sensitivity testing (Supplementary Information 1.5), we excluded phylogeny and extreme values from subsequent meta-analytic modelling.

We assessed publication bias in our meta-analysis using two methods. First, we investigated time-lag bias, where different patterns in genetic diversity change may be reported over the years of publication. Such bias may plausibly occur given that methods for measuring genetic diversity have advanced substantially in recent decades. Therefore, we fitted the final base model with the addition of a *z*-standardized year of publication fixed effect. Evidence of time-lag bias is inferred if the 95% HPD credible interval of the slope estimate excludes zero. Second, we plotted Hedges’ g* precision against Hedges’ g* in a funnel plot to visualize possible publication bias that can occur if, for example, smaller studies without statistically significant results are not published. We did not observe time-lag bias nor funnel plot asymmetry (Supplementary Information 1.5). Given the high heterogeneity and lack of detectable publication bias in our dataset, we proceeded with meta-regression modelling.

Meta-regressions were conducted to assess the impact of different moderator variables on genetic diversity change. These were broadly categorized into moderators related to: (1) how genetic diversity change is measured (that is, study design); (2) where genetic diversity change is measured and in what species (that is, population context); (3) ecological disturbances (that is, threats); and (4) conservation interventions (that is, conservation management). In meta-regression, the coefficients estimate how each category differs from the nominated reference group, represented by the intercept^65^. As all moderator variables were categorical, we performed separate meta-regressions for each moderator to avoid the confounding effects of correlations and allow for biologically meaningful interpretation of categorical variables relative to the intercept. For all models, moderator variables were only included if there were 10 or more effect sizes contributing to a category^65^. All models were run with the weakly informative inverse-gamma prior, the paper ID random effect and the standardized year midpoint and the number of generations over which the study took place (as a measure of study length) as fixed effects (unless otherwise specified), and additional fixed effects described in Supplementary Information 2.12.

### Inclusion and ethics statement

No ethical approval or guidance was required as data were collected only from previous studies.

### Reporting summary

Further information on research design is available in the Nature Portfolio Reporting Summary linked to this article.

## Online content

Any methods, additional references, Nature Portfolio reporting summaries, source data, extended data, supplementary information, acknowledgements, peer review information; details of author contributions and competing interests; and statements of data and code availability are available at 10.1038/s41586-024-08458-x.

## Supplementary information

Supplementary Information

Reporting Summary

Peer Review File

Supplementary Data 1 Bibliography for systematic review dataset.

Supplementary Data 2 Source data for preferred reporting items for systematic reviews and meta-analysis (PRISMA) steps.

Supplementary Data 3 Source data for systematic review and meta-analysis.

Supplementary Data 4 Web of Science advanced search string.

Supplementary Data 5 Generation length information for unique species in our dataset.

## Figures and Tables

**Extended Data Table 1:**
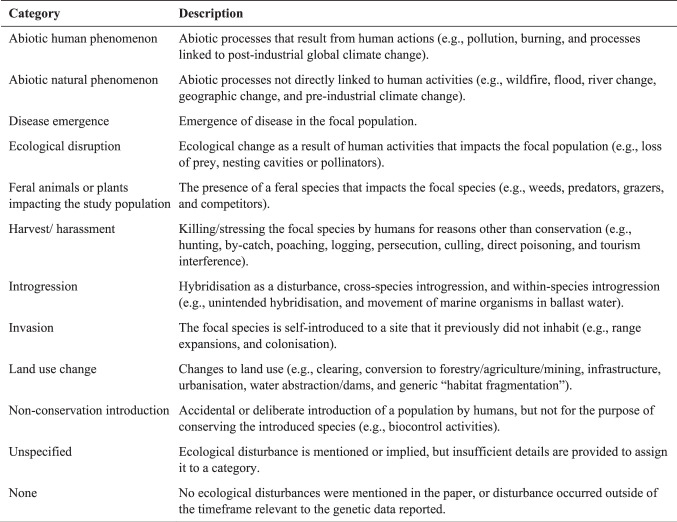
Impact meta-data detailing threatening processes, including ecological disturbance category and brief descriptions (full descriptions provided in Supporting Information 2.5)

**Extended Data Table 2:**
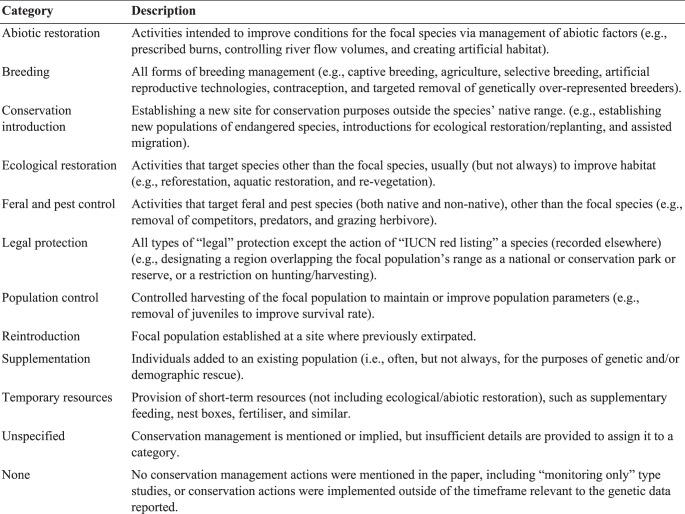
Impact meta-data detailing conservation management, including management action category and brief descriptions (full descriptions provided in Supporting Information 2.5)

## Data Availability

All datasets associated with this paper are available on Zenodo: 10.5281/zenodo.13903787 (ref. ^66^). The full bibliography of 882 included papers (including their DOIs) is provided in Supplementary Data 1. We used publicly available databases to obtain species characteristics for the 628 species included in our study. Full methods are in Supplementary Information 2.6. Generation lengths (Supplementary Data 5) were obtained from scientific literature and databases including Search FishBase (https://www.fishbase.se/search.php), AmphibiaWeb (https://www.amphibiaweb.org) and CABI Compendium (https://www.cabidigitallibrary.org/journal/cabicompendium). Threat status was sourced from the IUCN Red List of Threatened Species^30^ during June to August 2021. Invasive species status was sourced from the IUCN 100 of the World’s Worst Invasive Alien Species list (https://www.iucngisd.org/gisd/100_worst.php). Pathogen and pest statuses were sourced from the scientific literature and databases including the European and Mediterranean Plant Protection Organization Global Database (https://gd.eppo.int/), The Global Pest and Disease Database (www.gpdd.info), and CABI Compendium (https://www.cabi.org/isc).

## Code availability

Custom text mining code is available on Zenodo: 10.5281/zenodo.13903787 (ref. ^66^).

## References

[1] Bálint, M. et al. Cryptic biodiversity loss linked to global climate change. *Nat. Clim. Change* **1**, 313–318 (2011).

[2] Cardinale, B. J. et al. Biodiversity loss and its impact on humanity. *Nature* **486**, 59–67 (2012). 22678280 10.1038/nature11148

[3] Jaureguiberry, P. et al. The direct drivers of recent global anthropogenic biodiversity loss. *Sci. Adv.* **8**, eabm9982 (2022). 36351024 10.1126/sciadv.abm9982 PMC9645725

[4] Kardos, M. et al. The crucial role of genome-wide genetic variation in conservation. *Proc. Natl Acad. Sci. USA* **118**, e2104642118 (2021). 34772759 10.1073/pnas.2104642118 PMC8640931

[5] Convention on Biological Diversity. *Decision adopted by the Conference of the Parties to the Convention on Biological Diversity: 15/5 Monitoring framework for the Kunming–Montreal Global Biodiversity Framework* (United Nations Environment Programme, 2022); https://www.cbd.int/doc/decisions/cop-15/cop-15-dec-05-en.pdf.

[6] Convention on Biological Diversity. *Decision adopted by the Conference of the Parties to the Convention on Biological Diversity: 15/4 Kunming–Montreal Global Biodiversity Framework* (United Nations Environment Programme, 2022); https://www.cbd.int/doc/decisions/cop-15/cop-15-dec-04-en.pdf.

[7] Cowie, R. H., Bouchet, P. & Fontaine, B. The Sixth Mass Extinction: fact, fiction or speculation? *Biol. Rev.* **97**, 640–663 (2022). 35014169 10.1111/brv.12816 PMC9786292

[8] Reusch, T. B., Ehlers, A., Hämmerli, A. & Worm, B. Ecosystem recovery after climatic extremes enhanced by genotypic diversity. *Proc. Natl Acad. Sci. USA* **102**, 2826–2831 (2005). 15710890 10.1073/pnas.0500008102 PMC549506

[9] Hughes, A. R. et al. Ecological consequences of genetic diversity. *Ecol. Lett.* **11**, 609–623 (2008). 18400018 10.1111/j.1461-0248.2008.01179.x

[10] Pauls, S. U., Nowak, C., Bálint, M. & Pfenninger, M. The impact of global climate change on genetic diversity within populations and species. *Mol. Ecol.* **22**, 925–946 (2013). 23279006 10.1111/mec.12152

[11] Des Roches, S., Pendleton, L. H., Shapiro, B. & Palkovacs, E. P. Conserving intraspecific variation for nature’s contributions to people. *Nat. Ecol. Evol.* **5**, 574–582 (2021). 33649544 10.1038/s41559-021-01403-5

[12] Hoban, S. et al. Genetic diversity goals and targets have improved, but remain insufficient for clear implementation of the post-2020 global biodiversity framework. *Conserv. Genet.* **24**, 181–191 (2023). 36683963 10.1007/s10592-022-01492-0 PMC9841145

[13] Leigh, D. M., Hendry, A. P., Vázquez-Domínguez, E. & Friesen, V. L. Estimated six per cent loss of genetic variation in wild populations since the industrial revolution. *Evol. App.* **12**, 1505–1512 (2019). 10.1111/eva.12810 PMC6708419 31462910

[14] DiBattista, J. D. Patterns of genetic variation in anthropogenically impacted populations. *Conserv. Genet.* **9**, 141–156 (2008).

[15] Pinsky, M. L. & Palumbi, S. R. Meta-analysis reveals lower genetic diversity in overfished populations. *Mol. Ecol.* **23**, 29–39 (2014). 24372754 10.1111/mec.12509

[16] Miraldo, A. et al. An Anthropocene map of genetic diversity. *Science* **353**, 1532–1535 (2016). 27708102 10.1126/science.aaf4381

[17] Leroy, G. et al. Next-generation metrics for monitoring genetic erosion within populations of conservation concern. *Evol. App.* **11**, 1066–1083 (2018). 10.1111/eva.12564 PMC6050182 30026798

[18] Schmidt, C. et al. Continent-wide effects of urbanization on bird and mammal genetic diversity. *Proc. Royal Soc. B* **287**, 20192497 (2020). 10.1098/rspb.2019.2497 PMC7031673 32019443

[19] González, A. V., Gómez-Silva, V., Ramírez, M. J. & Fontúrbel, F. E. Meta-analysis of the differential effects of habitat fragmentation and degradation on plant genetic diversity. *Conserv. Biol.* **34**, 711–720 (2020). 31605401 10.1111/cobi.13422

[20] Exposito-Alonso, M. et al. Genetic diversity loss in the Anthropocene. *Science* **377**, 1431–1435 (2022). 36137047 10.1126/science.abn5642

[21] Hoban, S. et al. Global commitments to conserving and monitoring genetic diversity are now necessary and feasible. *BioScience* **71**, 964–976 (2021). 34475806 10.1093/biosci/biab054 PMC8407967

[22] Crandall, E. D. et al. Importance of timely metadata curation to the global surveillance of genetic diversity. *Conserv. Biol.* **37**, e14061 (2023). 36704891 10.1111/cobi.14061 PMC10751740

[23] Rey Benayas, J. M., Newton, A. C., Diaz, A. & Bullock, J. M. Enhancement of biodiversity and ecosystem services by ecological restoration: a meta-analysis. *Science* **325**, 1121–1124 (2009). 19644076 10.1126/science.1172460

[24] Langhammer, P. F. et al. The positive impact of conservation action. *Science* **384**, 453–458 (2024). 38662833 10.1126/science.adj6598

[25] Leigh, D. M. et al. Opportunities and challenges of macrogenetic studies. *Nat. Rev. Genet.* **22**, 791–807 (2021). 34408318 10.1038/s41576-021-00394-0

[26] Paz-Vinas, I. et al. Macrogenetic studies must not ignore limitations of genetic markers and scale. *Ecol. Lett.* **24**, 1282–1284 (2021). 33749962 10.1111/ele.13732

[27] Waldvogel, A.-M., Schreiber, D., Pfenninger, M. & Feldmeyer, B. Climate change genomics calls for standardized data reporting. *Front. Ecol. Evol.*10.3389/fevo.2020.00242 (2020).

[28] Hoban, S. et al. Global genetic diversity status and trends: towards a suite of Essential Biodiversity Variables (EBVs) for genetic composition. *Biol. Rev.* **97**, 1511–1538 (2022). 35415952 10.1111/brv.12852 PMC9545166

[29] Gurevitch, J., Koricheva, J., Nakagawa, S. & Stewart, G. Meta-analysis and the science of research synthesis. *Nature* **555**, 175–182 (2018). 29517004 10.1038/nature25753

[30] IUCN. *The IUCN Red List of Threatened Species* http://www.iucnredlist.org (accessed 3 June 2024).

[31] Wright, S. Evolution in Mendelian populations. *Genetics* **16**, 97–159 (1931). 17246615 10.1093/genetics/16.2.97 PMC1201091

[32] Pinto, A. V. et al. The impact of habitat loss and population fragmentation on genomic erosion. *Conserv. Genet.* **25**, 49–57 (2024).

[33] Boivin, N. L. et al. Ecological consequences of human niche construction: examining long-term anthropogenic shaping of global species distributions. *Proc. Natl Acad. Sci. USA* **113**, 6388–6396 (2016). 27274046 10.1073/pnas.1525200113 PMC4988612

[34] Frankham, R. et al. *Genetic Management of Fragmented Animal and Plant Populations* (Oxford Univ. Press, 2017).

[35] Mastretta-Yanes, A. et al. Multinational evaluation of genetic diversity indicators for the Kunming–Montreal Global Biodiversity Framework. *Ecol. Lett.* **27**, e14461 (2024). 38953253 10.1111/ele.14461

[36] ESRI, World Countries - Generalized [Esri Data & Maps]. Scale 1:5,000,000. https://hub.arcgis.com/datasets/esri::world-countries-generalized/about (2022).

[37] Page, M. J. et al. The PRISMA 2020 statement: an updated guideline for reporting systematic reviews. *Br. Med. J.* **372**, n71 (2021). 33782057 10.1136/bmj.n71 PMC8005924

[38] O’Dea, R. E. et al. Preferred reporting items for systematic reviews and meta-analyses in ecology and evolutionary biology: a PRISMA extension. *Biol. Rev.* **96**, 1695–1722 (2021). 33960637 10.1111/brv.12721 PMC8518748

[39] R Core Team. *R: A Language and Environment for Statistical Computing.* http://www.R-project.org/ (R Foundation for Statistical Computing, 2023).

[40] LeBeau, B. pdfsearch: search tools for PDF files. *J. Open Source Softw.* **3**, 668 (2018).

[41] Wickham, H., François, R., Henry, L. & Müller, K. dplyr: A grammar of data manipulation. Version 0.8.0 https://CRAN.R-project.org/package=dplyr (2019).

[42] Gagolewski, M. stringi: fast and portable character string processing in R. *J. Stat. Softw.* **103**, 1–59 (2022).

[43] Westgate, M. J. revtools: an R package to support article screening for evidence synthesis. *Res. Synth. Methods* **10**, 606–614 (2019). 31355546 10.1002/jrsm.1374

[44] Kingman, J. F. C. The coalescent. *Stoch. Process. Appl.* **13**, 235–248 (1982).

[45] Beaumont, M. A. Detecting population expansion and decline using microsatellites. *Genetics* **153**, 2013–2029 (1999). 10581303 10.1093/genetics/153.4.2013 PMC1460853

[46] Rees, J. A. & Cranston, K. Automated assembly of a reference taxonomy for phylogenetic data synthesis. *Biodivers Data J.* **5**, e12581 (2017). 10.3897/BDJ.5.e12581 PMC5515096 28765728

[47] Dinerstein, E. et al. An ecoregion-based approach to protecting half the terrestrial realm. *BioScience* **67**, 534–545 (2017). 28608869 10.1093/biosci/bix014 PMC5451287

[48] Spalding, M. D. et al. Marine ecoregions of the world: a bioregionalization of coastal and shelf areas. *BioScience* **57**, 573–583 (2007).

[49] Wickham, H. *ggplot2: Elegant Graphics for Data Analysis* (Springer, 2016).

[50] Wilkins, D. treemapify: Draw treemaps in ‘ggplot2’. Version 2.5.5 https://CRAN.R-project.org/package=treemapify (2021).

[51] Wilke, C. ggridges: Ridgeline plots in ‘ggplot2’. Version 0.5.4 https://CRAN.R-project.org/package=ggridges (2022).

[52] Wei, T. & Simko, V. R package corrplot: visualization of a correlation matrix. Version 0.92. *GitHub* https://github.com/taiyun/corrplot (2021).

[53] Kassambara, A. & Mundt, F. factoextra: Extract and visualize the results of multivariate data analyses. Version 1.0.7 https://CRAN.R-project.org/package=factoextra (2020).

[54] Michonneau, F., Brown, J. W. & Winter, D. J. rotl: an R package to interact with the Open Tree of Life data. *Methods Ecol. Evol.* **7**, 1476–1481 (2016).

[55] Yu, G. et al. ggtree: an R package for visualization and annotation of phylogenetic trees with their covariates and other associated data. *Methods Ecol. Evol.* **8**, 28–36 (2017).

[56] Xu, S. et al. ggtreeExtra: compact visualization of richly annotated phylogenetic data. *Mol. Biol. Evol.* **38**, 4039–4042 (2021). 34097064 10.1093/molbev/msab166 PMC8382893

[57] Yu, G. ggimage: Use image in ‘ggplot2’. Version 0.3.3 https://CRAN.R-project.org/package=ggimage (2023).

[58] Gearty, W. & Jones, L. A. rphylopic: an R package for fetching, transforming, and visualising PhyloPic silhouettes. *Methods Ecol. Evol.* **14**, 2700–2708 (2023).

[59] Koricheva, J., Gurevitch, J. & Mengersen, K. Handbook of Meta-Analysis in ecology and evolution (Princeton Univ. Press, 2013).

[60] Marfo, P. & Okyere, G. A. The accuracy of effect-size estimates under normals and contaminated normals in meta-analysis. *Heliyon* **5**, e01838 (2019). 31211256 10.1016/j.heliyon.2019.e01838 PMC6562325

[61] Hadfield, J. D. MCMC methods for multi-response generalized linear mixed models: the MCMCglmm R package. *J. Stat. Softw.* **33**, 1–22 (2010).20808728

[62] Plummer, M., Best, N., Cowles, K. & Vines, K. CODA: convergence diagnosis and output analysis for MCMC. *R News* **6**, 7–11 (2006).

[63] Nakagawa, S. & Santos, E. S. A. Methodological issues and advances in biological meta-analysis. *Evol. Ecol.* **26**, 1253–1274 (2012).

[64] Nakagawa, S. et al. Quantitative evidence synthesis: a practical guide on meta-analysis, meta-regression, and publication bias tests for environmental sciences. *Environ. Evid.* **12**, 8 (2023). 39294795 10.1186/s13750-023-00301-6 PMC11378872

[65] Deeks, J. J., Higgins, J. P. T. & Altman, D. G. in *Cochrane Handbook for Systematic Reviews of Interventions Version 6.4 (Updated August 2023)* (eds Higgins, J. P. T. et al.) Ch. 10 (Wiley-Blackwell, 2023).

[66] Shaw, R. E. Supporting data and code for: Global meta-analysis of genetic diversity (v1.0). *Zenodo*10.5281/zenodo.13903787 (2024).

