# Supplementary material for: Global meta-analysis shows action is needed to halt genetic diversity loss
Source: Nature. 2025 Jan 29;638(8051):704–10. doi: 10.1038/s41586-024-08458-x (PMC11839457; doi:10.1038/s41586-024-08458-x)
Supplement: Supplementary file 1 — Supplementary Information [file 41586_2024_8458_MOESM1_ESM.pdf]

nature portfolio

https://doi.org/10.1038/s41586-024-08458-x

Supplementary information

Global meta-analysis shows action is needed to halt genetic diversity loss

In the format provided by the authors and unedited

SUPPORTING INFORMATION

Supplementary results and methods for manuscript, entitled:

Global meta-analysis shows action is needed to halt genetic diversity loss.

Authors: Robyn E. Shaw* & Katherine A. Farquharson*; Michael W. Bruford†; David J Coates; Carole P. Elliott; Joachim Mergeay; Kym M. Ottewell; Gernot Segelbacher; Sean Hoban; Christina Hvilsom; Sílvia Pérez-Espona; Dainis Ruņģis; Filippos Aravanopoulos; Laura Bertola; Helena Cotrim; Karen Cox; Vlatka Cubric-Curik; Robert Ekblom; José A. Godoy; Maciej K. Konopiński; Linda Laikre; Isa-Rita M. Russo; Nevena Veličković; Philippine Vergeer; Carles Vilà; Vladimir Brajkovic; David L. Field; William P. Goodall-Copestake; Frank Hailer; Tara Hopley; Frank E. Zachos; Paulo C. Alves; Aleksandra Biedrzycka; Rachel M. Binks; Joukje Buiteveld; Elena Buzan; Margaret Byrne; Barton Huntley; Laura Iacolina; Naomi L. P. Keehnen; Peter Klinga; Alexander Kopatz; Sara Kurland; Jennifer A. Leonard; Chiara Manfrin; Alexis Marchesini; Melissa A. Millar; Pablo Orozco-terWengel; Jente Ottenburghs; Diana Posledovich; Peter B. Spencer§; Nikolaos Tourvas; Tina Unuk Nahberger; Pim van Hooft; Rita Verbylaite; Cristiano Vernesi; Catherine E. Grueber‡

* Joint first author, † deceased 13 April 2023, § deceased 11 March 2024, ‡ author for correspondence

Table of Contents

SUPPORTING INFORMATION 1. SUPPLEMENTARY RESULTS 4
Supporting Information 1.1. PRISMA and missing data 4
Overview 4
Statistical error not reported 4
Infinite confidence intervals – 4
Supporting Information 1.2. Systematic review dataset summary: bibliographic characteristics 6
Supporting Information 1.3. Systematic review dataset summary: study characteristics 7
Study design 7
Population context 7
Supporting Information 1.4. Extreme values and variances 9
Extreme values of Hedge's g* 9
Validating Hedge's g* as our effect size estimator 10
Supporting Information 1.5. Base model, sensitivity testing and publication bias 12
Overall genetic diversity change 12
Sensitivity testing 12
Publication bias 14
Supporting Information 1.6. Meta-regressions: study design 16
Study duration 16
Statistical method 16
Genetic marker type 16
Genetic diversity metric type 17
Supporting Information 1.7. Meta-regressions: population context 19
Realm 19
Taxonomic class 19
Threat status 20
Supporting Information 1.8. Meta-regressions: threats 23
Ecological disturbance 23
Supporting Information 1.9. Meta-regressions: conservation management 25
Conservation management action 25
Conservation management action overall and by IUCN Red List threat status 25
Conservation management intensity 26
Conservation management action by taxonomic class 26
Supporting Information 1.10. Systematic review dataset summary: threats 31
Overview of ecological disturbance across entire dataset 31
Ecological disturbance across taxonomic classes 31
Supporting Information 1.11. Systematic review dataset summary: conservation management 34
Overview of conservation management across entire dataset 34
Conservation management across taxonomic classes 34
SUPPORTING INFORMATION 2. SUPPLEMENTARY METHODS 37
Supporting Information 2.1. Literature search 37
Supporting Information 2.2. Text mining 39
Page number identification 39
Keywords 39
Thematic clusters 39
Supporting Information 2.3. Data screening and extraction protocol development 44
Supporting Information 2.4. Recording genetic diversity change 45
Measurements of genetic diversity change 45
Time frame of the measurement 45

Supporting Information 2.5. Impact meta-data ... 47
Ecological disturbance ... 47
Conservation management ... 47
Management intensity ... 47
Supporting Information 2.6. Additional variables ... 51
Taxonomic information ... 51
Genetic marker type ... 51
Generation length ... 51
Domestic species ... 51
Threat status ... 52
Invasive species ... 52
Pathogens and pests ... 52
Supporting Information 2.7. Identification and removal of non-independent data ... 55
Duplicate publication of genetic data ... 55
Stochastic dependency ... 55
Supporting Information 2.8. Missing data ... 56
Statistical error not reported ... 56
Infinite confidence intervals ... 56
Zero variance ... 56
Supporting Information 2.9. Unconventional study designs ... 57
Adult/juvenile comparisons of plants ... 57
Before/after comparisons over very short time frames ... 57
Within-study variation in number of samples or loci ... 57
Uncommon genetic diversity statistics ... 57
Uncommon genetic marker types ... 58
Data subset analyses ... 58
Excluded from effect size calculation ... 58
Supporting Information 2.10. Repeat extractions and validation ... 59
Overview ... 59
Validation subgroup 1 ... 59
Validation subgroup 2 ... 59
Supporting Information 2.11. Additional meta-analytic procedures ... 60
Sample sizes ... 60
Hedge's g and its error* ... 60
Meta-analysis base model ... 61
Phylogenetic modelling ... 62
Sensitivity testing of our base model ... 62
Supporting Information 2.12. Meta-regression ... 63
Study design ... 63
Population context ... 63
Threats ... 63
Conservation management ... 63
Conservation management actions by taxonomic class ... 64
Management intensity ... 64
References ... 66

SUPPORTING INFORMATION 1. SUPPLEMENTARY RESULTS

Supporting Information 1.1. PRISMA and missing data

Overview **–** Our search of the literature (18 January 2019) identified 80,271 records of published temporal measures of genetic diversity (Extended Figure 2; Supporting Data 2). Potentially relevant records were identified with text mining, then screened, validated and cleaned by 57 researchers using a streamlined data management approach that generated a dataset of 882 records (1.1%) for meta-analysis, providing 4,023 rows of data (i.e., measures of genetic diversity change) (Extended Figure 2; Supporting Data 1 and 3) that met our inclusion criteria and were successfully converted to effect sizes (Hedges g*; later refined to 4,021 rows after two errors were excluded) (Supporting Information 1.4). Most records were excluded during text mining and screening, due to the absence of genetic metrics (44.5%) or because they failed to meet the inclusion criteria (51.76%). This was followed by exclusion due to the presence of duplicate studies in the dataset (1.93%), or because statistical error for the measurement was not reported (0.54%; including significance reported as ‘p > 0.05’, more details below). Records were also excluded if full texts could not be sourced (0.08%).

During data extraction, additional data were excluded if the reported measurements could not be used to calculate genetic diversity change (0.04%; e.g., directionless metrics such as temporal F_{ST}, where F_{ST} was calculated between time-points rather than among populations at two separate time-points) (Supporting Information 2.9), or the data were not independent (0.02%; i.e., used common data across multiple studies) (Supporting Information 2.7). The remaining excluded records (0.03%) reported data where effect sizes were not calculable (e.g., estimates with infinite or zero variances, see below, or where the necessary information for effect size calculation was missing), or were identified as outliers (including biologically implausible estimates or data that did not fit into our analytical framework).

Statistical error not reported **–** In total, 437 publications were excluded because they did not report an appropriate measure of statistical error (including significance reported as ‘p >0.05’) that could be used to estimate the effect size precision. Within these, there were no obvious patterns in geographic or taxonomic characteristics of the publications, which overlapped with our main analysis. These 437 publications corresponded to data from 10 biogeographical terrestrial and marine realms, or in some cases multiple realms, and their distribution across realms was comparable to the main dataset (with most from the Palearctic, Nearctic, Afrotropic, Neotropic, or Australasian realms, see Supporting Information 1.3). The focal species within these studies were mainly animals (vertebrates = 68.9%, invertebrates = 31.1%), and their distribution across taxonomic kingdoms and phyla was also similar to the main dataset (see Supporting Information 1.3). Taken together, these summary statistics suggest that the excluded data likely represent a random sample of temporal population genetic research, and that their exclusion is unlikely to bias our conclusions.

Infinite confidence intervals **–** We identified 96 measurements from 45 publications that contained infinity. Note that the final number of publications excluded in this category was 14 as recorded in our PRISMA flowchart (Extended Figure 2). This is because summary of infinite values occurred in parallel to other data cleaning procedures and some publications were excluded against other inclusion criteria. Across the 45 publications that contained infinity, publication dates ranged from 1999-2018; all but one were post 2007, with the median year of publication being 2014. We note that popular population genetic analysis software that may generate estimates with infinite confidence intervals were published in or after 2004: NeEstimator 1.3[67], LDNE[68], NeEstimator 2.01[69].

Taxonomically, the 96 measurements were dominated by data from ray-finned fishes, Actinopterygii (n = 71, 74.0%), with the remaining records including amphibians, birds, bivalves, gastropods, insects, mammals, reptiles and just one plant, an eelgrass. Most of these included results from analysis of nuclear microsatellites (n = 89, 92.7%) with just a few records from SNPs and allozymes. There were, notably, no measurements of genetic diversity change generated from mitochondrial markers in this data subset. The median sample numbers used to derive the metrics were 45 and 50, for early and recent samples, respectively, with corresponding inter-quartile range c. 30-90. These generalisations highlight the context for the infinite confidence values: such populations have presumably high genetic diversity. For example, fish often have reasonably large population sizes, and microsatellites are generally highly polymorphic markers and used with sample sizes generally >30, implying that all alleles at frequencies above 5% would be captured to maximise the amount of genetic variation analysed. Some additional 'rare' alleles would also be expected to be captured but these appear to have been filtered prior to analysis in most of the studies examined here.

All measurements that gave infinite confidence intervals were either N_e (effective population size, n = 85) or N_b (effective number of breeders, n = 11). A recurring theme in these works was to interpret N_e as N_b when the metric was estimated from organisms with overlapping generations. Most of these measurements (n = 87, 90.6%) were estimated using a linkage disequilibrium-based method, typically with the software packages listed above. The remaining 9 measurements were estimated using a moment-based temporal approach, usually with the same software packages. This similarity among the records is consistent with claims that the LD method can generate infinite estimates, such as when sampling error is larger than any signal of LD and drift[70]. To facilitate results interpretation despite infinite upper CI estimates, authors sometimes discuss only the lower 95% jackknife CI within the context of minimum possible population sizes[70,71]. Because these data have infinite error or include infinite values, they were excluded from our study.

Supporting Information 1.2. Systematic review dataset summary: bibliographic characteristics

Our final dataset included studies on a wide range of topics published across a total of 217 journals (Extended Figure 3a). Journal topics spanned expected general fields of ecology, conservation, and genetics, as well as more specialist fields of forestry, agronomy, horticulture, agriculture, oceanography, parasitology, fisheries and tropical medicine. The five topic categories that included the greatest number of records were ecology (25.9%), biodiversity conservation (14.2%), marine and freshwater biology (10.7%), multidisciplinary sciences (7.4%), and genetics and heredity (6%). Almost half of our records came from 201 journals that were represented in the dataset fewer than 10 times ('Other', 47.8%). In general, these were region, taxon, or discipline-specific. After these, the journals with the greatest number of records were in the field of genetics, including Molecular Ecology (13.3%), Conservation Genetics (9.6%), and Heredity (5.9%), or in multidisciplinary journals such as PLoS One (4.5%).

Publication years ranged from 1985 to 2019 (Extended Figure 3b), with the earliest and most recent studies both from the Palearctic realm, reporting genetic diversity measured with allozyme or SNP markers, respectively. Our dataset included studies published in journals across a wide range of impact factors, suggesting our search has captured a substantial cross-section of the literature (Extended Figure 3b). There was no clear trend in impact factor rankings across data generated across various terrestrial or marine realms (noting that the realm refers to the location where data were collected, not author affiliations) (Extended Figure 3b). An average of five effect sizes were obtained per study, with a maximum of 79 collected for a single study. There was no statistical difference between the average number of effect sizes collected for terrestrial compared to marine realms (range = 1 to 11; $t(16.54) = 0.24$, $p = 0.82$).

Supporting Information 1.3. Systematic review dataset summary: study characteristics

Study design – The study duration and time period varied considerably across studies, with an average study duration of 111 years (median = 6, range = <1 – 12,500 years, Extended Figure 4e) and a median midpoint of the year 2000 (range = 10486 BCE – 2018 CE, Extended Figure 4f). Studies using coalescent methods tended to investigate genetic diversity over longer durations (mean = 1152 years, median = 306) and at earlier time periods (median = 1858 CE) compared to those using linear measures of change (e.g., regression; mean duration = 21.6 years, median duration = 10 years, median midpoint = 2001 CE) or those that reported genetic diversity at two individual time-points (mean duration = 28.5 years, median duration = 5 years, median midpoint = 2001 CE) (Extended Figure 4e-f).

Studies employed a diverse range of genetic marker types to measure genetic diversity across nuclear (89.5%), mitochondrial (15.9%), and/or chloroplast genomes (0.3%), or used genealogies (1.7%) or a mixed approach (0.7%; noting that some studies report multiple molecular data types) (Extended Figure 4d, upper). Microsatellites were the most popular marker type in our dataset (69.5% of studies), followed by nucleotide sequences (11.1%), allozymes (8.5%), haplotypes (6.1%), single nucleotide polymorphisms (SNPs; 4.4%) and a variety of others that were less commonly reported (total 5%). Microsatellites increased in use from 1996 and remained the most common marker type in our dataset from 1999 onwards, when they overtook allozymes (Extended Figure 4d, lower). The number of studies reporting data from allozyme, amplified fragment length polymorphisms (AFLPs), minisatellites and haplotype markers remained low but consistent over all publication years. In contrast, the use of SNPs and whole genome sequencing (WGS) increased by roughly five times from 2009 to 2018 (noting that one study from January 2019 was grouped with those published in 2018), corresponding with greater numbers of loci reported (Extended Figure 4d, lower). Nucleotide sequence data first appeared in our dataset in the year 2000, and the number of studies using this marker type varied considerably from year to year.

Population context – The 882 records in our dataset were global in geographic coverage, collected from 141 countries including all terrestrial realms and almost all marine realms, except for the Western Indo-Pacific (Figure 1a; Extended Figure 4a-b). Most studies were performed with data collected from terrestrial realms (90.4% of our dataset, Extended Figure 4a), followed by 9.4% from marine realms (Extended Figure 4b), and the remaining 0.2% included data from both. The top five realms that provided the most data were all terrestrial, and included the Palearctic (40.9%), followed by the Nearctic (25.4%), Neotropic (7.8%), Australasia (5.6%), and Afrotropic (5.3%).

The vast majority of species were animals (84.7%; vertebrates = 59.2%, invertebrates = 25.5%), followed by plants (12.7%), fungi (1.9%) and chromists (0.6%) (Figure 1b). The 628 unique species were distributed across 37 taxonomic classes, and we were able to obtain phylogenetic information for 624 taxa (excluding *Euparthenia bulinea*, *Drosophila pseudoobscura*, *Batillaria attramentaria* and *Diplodus sargus*) (Figure 1b). For meta-analytic sensitivity testing purposes (Supporting Information 1.5), a further two species were excluded (*Stegastes partitus* and *Eleutheronema tetradactylum*), as they could not be placed monophyletically on the Open Tree of Life dataset used for modelling. Based on IUCN Red List threat status classification, most species were non-threatened (Least Concern = 39.3%, Near Threatened = 6.1%) or the threat status was unknown (Data Deficient = 1.8%, Not Evaluated = 33.8%). Approximately one fifth of species were listed as threatened (Vulnerable = 7.3%, Endangered = 6.7%, Critically Endangered = 4.9%, Extinct = 0.2%) (Figure 1b; Extended Figure 4a-b). We noted a strong phylogenetic bias in whether a taxon had been assessed for IUCN Red List status, with 92% (341/371) of species within phylum Chordata having been assessed and only 30% (77/253) of taxa across the remaining 15 phyla in our dataset with an assessment (Figure 1b). Unsurprisingly, generation length (Supporting Data 5) showed a strong phylogenetic signal with taxa within Arthropoda, Oomycota and Ascomycota having the

shortest generation lengths, and corals (Cnidaria) and tree species (Tracheophyta) the longest (Figure 1b). We observed weak correlation in study effect sizes (Hedge's g*) across the phylogeny (Figure 1b). Visually, extreme effect size values (Supporting Information 1.4) were dispersed across the phylogeny, and we did not observe a trend in phylogenetic clustering of positive or negative effect sizes (Figure 1b).

Supporting Information 1.4. Extreme values and variances

Extreme values of Hedge's g* – We observed several Hedge's g* values that were substantially larger (in absolute magnitude) than the bulk of the dataset. Sensitivity testing revealed that these data had detectable influence on our results (see Supporting Information 1.5), and so we removed the most extreme 1% of the dataset (0.5% highest and 0.5% lowest values) from the meta-analysis to avoid their influence on the main conclusions. We chose to remove a small percentage to avoid substantially reducing our dataset (equivalent to 40 rows of data) and removed this data symmetrically from the upper and lower ranges to avoid biasing our parameter estimates. The 40 rows of removed data were further examined manually.

We found that many of the extreme values reflected population size changes (and associated genetic diversity metrics) across several orders of magnitude, often representing a rebound from the brink of extinction or extreme losses of genetic diversity as a result of domestication and selective breeding. Extreme negative Hedge's g* values ranged from -24.27 to -6.30, while extreme positive values ranged from 3.86 to 20.92. These 40 rows of data were obtained from 33 publications, representing studies of 31 species. These species overlapped with the broad distribution of species seen in our main analysis (5 threatened species, 12 non-threatened species, and 14 species of unknown threat status). The range of marker types used was similar to our main dataset, and included microsatellite, pedigree, AFLP, allozyme, mixed, haplotype, nucleotide, amino acid, whole genome sequencing and 'other'.

Two of the extreme values were found to be invalid data entries and were removed altogether. The remaining 38 rows of data represented genuine change across all groups of genetic diversity metrics (allelic richness, gene diversity, individual heterozygosity, inbreeding). Since the absolute value of the Hedge's g* is determined by precision (errors on the estimate), even changes with a small biological effect may yield large absolute values if the precision is very high. In some cases, the error was calculated across replicated sites ("subpopulations") that underwent the same external influence. However, since these subpopulations may not be independent of each other due to gene flow, this may have inflated precision, thereby yielding large Hedge's g* values despite representing small to marginal genetic diversity changes, that may not represent biologically meaningful differences (e.g.,[54,72,73]) (see also Supporting Information 2.7 and 2.9, which details methods for ensuring independence among effect sizes).

Effective population size estimates can plausibly change by several orders of magnitude over short time frames in nature, and thereby provide extreme Hedge's g* values, positive or negative. This was the case for several populations that underwent severe human-induced population declines, with or without subsequent recovery. Noticeably, we find this repeatedly in large-sized marine and terrestrial animals (e.g.,[74–78]), but we also find this as an effect of crop domestication[79] or of natural catastrophes[80]. A few cases represent extreme reductions in genetic diversity (e.g., H_E, π) that resulted from strong and sustained human-induced population declines (e.g.,[81–83]), or from an extreme natural disaster[84]. Genetic rescue and admixture following restocking from genetically dissimilar source populations is also a repeated cause of genuinely large changes in genetic diversity metrics, yielding extreme Hedge's g* values (especially in Salmonid fishes, e.g.,[85,86]), in one case this was a natural process during the invasion of a non-native species[87]. Across domesticated animals we regularly found genuine changes in inbreeding or effective size that were measured very precisely due to the nature of the data (detailed pedigrees estimating inbreeding nearly exactly), thereby yielding large absolute Hedge's g* values (e.g.,[57,88–92]).

We report our main analysis excluding these extreme values because they were not biased towards certain data types (e.g., metrics used for measuring genetic diversity), and so that our findings

represent the bulk (99%) of literature available for measuring genetic diversity change. Our meta-analysis dataset therefore included 3,983 effect sizes across 871 publications and 622 species.

Validating Hedge's g* as our effect size estimator **–** To further interrogate the suitability of Hedge's g* for our dataset, we investigated the ratio of the difference in errors on the early and recent diversity estimates, which are together used to calculate each effect size for two time-point and coalescent data (in our study, linear measures of change are intrinsic estimates of effect). We found, that for 95.9% (N = 3759 / 3920) of two time-point and coalescent data in our meta-analysis, the difference between the standard deviation of the early and recent estimates was within (1:10, 10:1). Marfo & Okyere[93] found that Hedge's g was the most appropriate common effect size under deviations of this magnitude, which we take as justification for our choice that Hedge's g is a reliable estimator for our dataset.

For the two time-point data, there was a linear relationship between the early and recent errors (Supporting Figure 1). For the small portion of our dataset where the error deviation was greater (N = 161 effect sizes; 4.1% of the combined two time-point and coalescent dataset, 4.0% of the total meta-analytic dataset), two-thirds of these (66.4%) were from coalescent studies, while the remainder were from two time-point studies. Given that two time-point studies are the vast majority of our total meta-analytic dataset (3769 / 3983 = 94.6%), this pattern indicates a high rate of error deviation in the coalescent data (Supporting Figure 2), compared to the rest of the dataset.

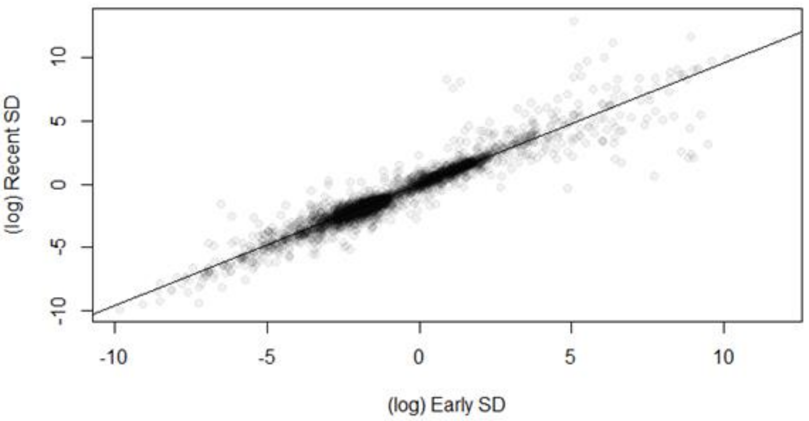

Supporting Figure 1. Two time-point comparison studies (N = 3,769) show a linear relationship between early and recent errors (SD = standard deviation)

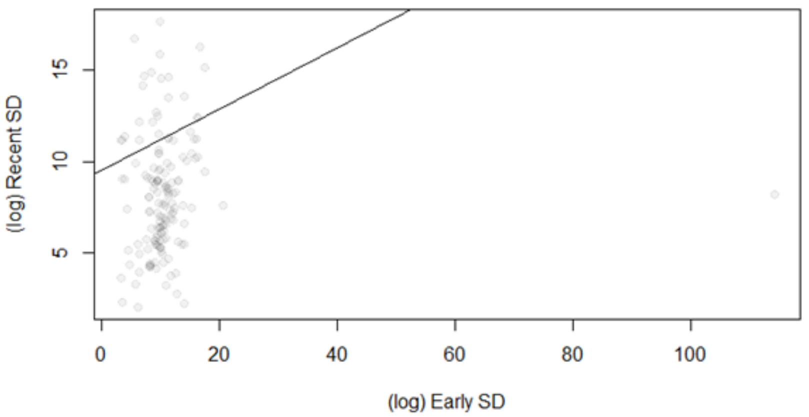

Supporting Figure 2. Coalescent analysis data (N = 151) contain a large number of comparisons where the errors for early and recent measurements of diversity were more different than (1:10, 10:1) (SD = standard deviation).

An error deviation greater than (1:10, 10:1) does not necessarily indicate that our data are biased, and may even be expected under very large changes in mean genetic diversity over time, in line with Taylor's law[94] and other large-effect analyses[95]. As these values are outside the ranges tested by statisticians in sensitivity analyses (e.g.,[37,93]), it is not known whether they present a problem for our analysis. Due to multiple rounds of dataset validation (e.g. Supporting Information 2.10), we do believe that the coalescent data in our analysis represent genuine biological measurements. In our own sensitivity testing, we identified separately the data generated by each statistical method (Figure 2b, Supporting Information 1.6) and saw no indication that our model parameter estimates, nor their variances, were substantially biased for data from coalescent studies. All models reported in this study met standard model validation criteria (corresponding methods reported under "Methods: Meta-analysis"). In the absence of evidence for statistical bias (which we acknowledge is not evidence of "no" bias), we therefore retained these data in our overall analysis, to avoid bias that may result from the systematic removal of real data.

Supporting Information 1.5. Base model, sensitivity testing and publication bias

Overall genetic diversity change – Our primary base model excluded extreme values and we considered genetic diversity change per generation and through time by including the number of focal species' generations over which the study took place (effect size found to be close to zero) and the midpoint of the study interval (effect size found to be slightly positive: more genetic diversity change was observed with a higher, more-recent year midpoint) (Supporting Table 1 and Supporting Figure 3A). We recorded a small but statistically significant negative overall genetic diversity change (Hedge's g* posterior mean = -0.11; 95% HPD = -0.15, -0.07), corresponding to a small mean loss of genetic diversity across all studies recorded in our dataset (Figure 2a). Heterogeneity of our base model was very high (I^2_{total} = 99.22%), indicating a high degree of variation in genetic diversity change across the studies in our analysis. Variation at the paper ID level accounted for approximately half of the heterogeneity observed (I^2_{paper} = 56.24%; $I^2_{residual}$ = 43.00%). These high levels of heterogeneity are consistent with other observations in ecological studies[96], and likely arise from methodological and/or biological variation among studies, which are therefore investigated in subsequent meta-regressions.

Sensitivity testing – We tested whether phylogenetic relationships among species could account for variation in effect sizes, for example evolutionary signal in the rate of genetic diversity change (e.g., if closely related species share characteristics that result in similar degrees of genetic diversity change). Expanding our base model to include phylogeny as a random effect gave a posterior mean contribution of the random effect of phylogeny to overall variance of 3.79% (95% HPD: 0.84%, 8.38%; phylogenetic heritability: 5.48%). This result suggests a very weak effect of phylogeny on Hedge's g* (also seen in Supporting Information 1.3), and that most variation in genetic diversity change results from study-level processes. Adding the phylogeny to the model decreased the precision of our main effect estimate (intercept; the confidence intervals included the value of the estimate calculated when phylogeny was excluded), bringing the overall estimate of genetic diversity change close to zero, with a wide 95% HPD CI (Supporting Table 1; Supporting Figure 3A). Due to the weak contribution of phylogeny to overall variance, we excluded phylogeny from our subsequent meta-analytic modelling.

Further testing to determine whether the exclusion of extreme values (1% of our dataset) (Supporting Information 1.1) had a substantive effect on our meta-analytic estimates of genetic diversity change revealed subtle changes in our base models when these values were included, with a slightly decreased intercept (overall effect) and slightly increased effect of year midpoint (Supporting Table 1; Supporting Figure 3A). Considering these visible changes were generated by adding only 1% of the data volume, inclusion of these data presented interpretation concerns for downstream moderator analyses, especially analysis of subsets, as including these values may lead to ambiguous inferences. We therefore opted to maintain the exclusion of our extreme values from subsequent modelling, to ensure that findings were representative of the remaining 99% of data. As indicated above, our symmetrical removal of the top 0.5% and bottom 0.5% of effect sizes was intended to avoid biasing our meta-analytic mean estimates of genetic diversity change.

In our finally sensitivity test, we investigated the implications of using generations to scale time in our analysis by comparing the base model to an alternative 'study interval model', where the length of the study in years is used to control for study length, rather than the number of generations of the focal species (Supporting Table 1; Supporting Figure 3B). Controlling for study length (i.e., time) in years rather than generations did not influence the overall estimate of genetic diversity change, suggesting that our results are robust to minor discrepancies in generation length estimates (provided in Supporting Data 5).

Supporting Table 1. Model summaries of overall genetic diversity change, sensitivity testing and publication bias, describing fixed effects, sample sizes (displayed as number of effect sizes / number of papers / number of species), and effect sizes (modelled and predicted genetic diversity change, i.e., mean Hedge's g* with 95% highest posterior density credible intervals). Effect sizes in bold are significantly different to the intercept (here, all are nonsignificant).

**Model**	**Fixed effect**	**Sample size**	**Modelled effect sizes (95% HPD Cis)**	**Predicted effect sizes (95% HPD Cis)**
Overall genetic diversity change				
Base model	Intercept	3983 / 871 / 622	-0.11 (-0.15, -0.07)	-0.11 (-0.15, -0.07)
	Generations	3983 / 871 / 622	0.01 (-0.04, 0.06)	-0.10 (-0.16, -0.03)
	Year midpoint	3983 / 871 / 622	0.03 (-0.03, 0.09)	-0.08 (-0.15, 4.45×10^{-3})
Sensitivity testing				
Base model with extreme values	Intercept	4021 / 881 / 628	-0.12 (-0.17, -0.07)	-0.12 (-0.17, -0.07)
	Generations	4021 / 881 / 628	0.03 (-0.03, 0.11)	-0.08 (-0.16, -6.68×10^{-4})
	Year midpoint	4021 / 881 / 628	0.09 (0.02, 0.15)	-0.03 (-0.12, 0.06)
Base model (i.e., without extreme values) with phylogeny	Intercept	3936 / 864 / 616	-0.05 (-0.28, 0.12)	-0.05 (-0.28, 0.12)
	Generations	3936 / 864 / 616	-7.00×10^{-4} (-0.05, 0.05)	-0.05 (-0.26, 0.14)
	Year midpoint	3936 / 864 / 616	0.01 (-0.05, 0.07)	-0.04 (-0.26, 0.15)
Base model with extreme values and phylogeny	Intercept	3971 / 874 / 622	-0.05 (-0.26, 0.15)	-0.05 (-0.26, 0.15)
	Generations	3971 / 874 / 622	0.02 (-0.04, 0.09)	-0.03 (-0.26, 0.18)
	Year midpoint	3971 / 874 / 622	0.07 (-5.68×10^{-4}, 0.14)	0.01 (-0.23, 0.21)
Study interval model	Intercept	3983 / 871 / 622	-0.11 (-0.15, -0.07)	-0.11 (-0.15, -0.07)
	Time (years)	3983 / 871 / 622	-0.03 (-0.08, 0.01)	-0.14 (-0.2, -0.08)
Publication bias				
Time lag	Intercept	3983 / 871 / 622	-0.11 (-0.15, -0.07)	-0.11 (-0.15, -0.07)
	Generations	3983 / 871 / 622	0.01 (-0.05, 0.06)	-0.10 (-0.17, -0.03)
	Year midpoint	3983 / 871 / 622	0.03 (-0.03, 0.09)	-0.08 (-0.17, -0.01)
	Publication year	3983 / 871 / 622	0.02 (-0.07, 0.10)	-0.09 (-0.17, 0.01)

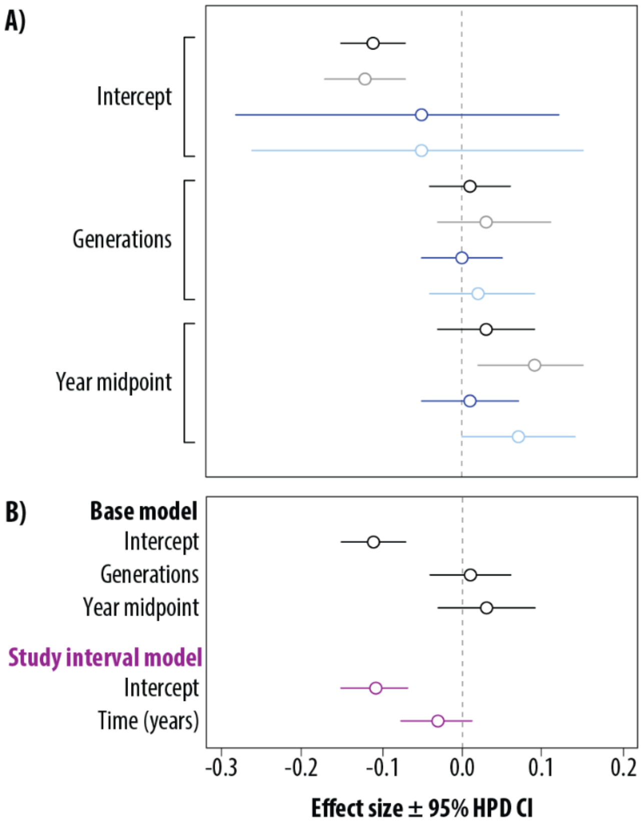

Supporting Figure 3. Sensitivity testing for meta-regression of genetic diversity change across our entire dataset (A). The forest plot shows the model outcomes (parameter estimates) for the following analyses: black = base model, grey = the base model with the inclusion of extreme values, dark blue = the base model with the inclusion of phylogeny as a random factor, light blue = base model with the inclusion of extreme values plus phylogeny as a random factor. Panel B shows additional sensitivity testing with regards to controlling for the length of the study, comparing generation length and the study midpoint (black = base model) to the time interval of the study in years (purple). For all models, paper ID was fitted as a random factor. Effect sizes (circles) were measured as mean Hedge's g* and error bars are the 95% highest posterior density credible intervals. Filled circles represent effect sizes that are significantly different to the intercept (here, all are nonsignificant). All sample sizes are provided in Supporting Table 1 (n = the number of effect sizes / number of papers / number of species).

Publication bias – Two statistical methods (and a variety of study design considerations, see below) were used to determine whether it was plausible that publication bias influenced our results, using our main model (i.e., base model without extreme values or phylogeny). Firstly, the possibility of time-lag bias (i.e., where results become more or less significant over time) was investigated by adding a fixed effect of study publication year to the model. The effect of publication year was not statistically significant (Supporting Table 1). Second, a funnel plot was used to visualise possible publication bias by plotting the precision of the effect size, calculated as:

$$\sqrt{\frac{1}{v_i}}$$

where v_i is the error on Hedges g* (calculated according to Supporting Information 2.11) against the effect size (Hedges g*). No systematic asymmetry was observed in our dataset (Supporting Figure 4). We further note that additional potential sources of publication bias were deliberately minimised by our Methods:

- Hypothesis-driven and search bias: we undertook several strategies to avoid selecting studies biased by their findings, as most study designs that reported measures of genetic diversity over time were suitable for inclusion in our analysis, regardless of whether recording change was an objective of the study. For example, our initial search terms were symmetrical in relation to

"gain" and "loss", our initial text mining used terms related to methods, rather than the degree or direction of any changes recorded, and our manual screening and extraction protocols were explicitly agnostic to the directionality of genetic diversity change, if any (see Supporting Information 2.1-2.4).

- Citation bias (occurs when strong effects may be more likely to be highly cited or published in higher impact journals): we did not restrict our search to journals/papers based on their citation scores. As seen in Supporting Information 1.2, almost half of our data came from 201 journals that were represented <10 times in our dataset, indicating reports from narrow-focus subject-matter journals, and much of our data was obtained from journals with low Web of Science journal impact factor category percentile (2020) (Supporting Information 1.2). Together these findings confirm that citation bias was unlikely to affect our results.
- Multiple-publication bias (occurs when results of "successful" studies are more likely to be reported on multiple times): we conducted both ad hoc and systematic searches across our dataset for duplicate reports, in doing so a total of 164 studies were flagged as potentially non-independent studies (i.e., duplicate datasets), which were resolved according to our inclusion criteria (targeting our priority genetic diversity metrics, and/or the largest dataset, and/or the most recent study, according to our screening and extraction protocol, see Supporting Information 2.7), resulting in the exclusion of 16 studies.
- Language bias (occurs when reports in languages other than English may be less likely to be detected): we did not restrict our search by language, and our team members translated non-English abstracts to verify inclusion criteria (69 non-English language abstracts were flagged and screened).

Taken together, we believe there is strong evidence that our conclusions are not likely to be influenced by publication bias.

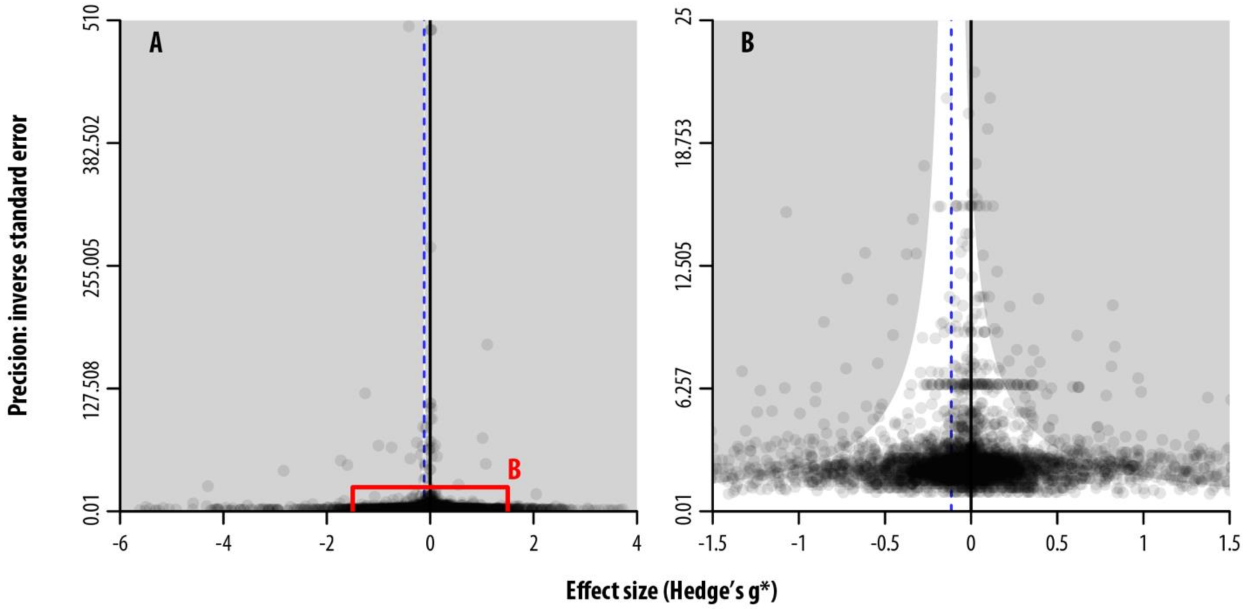

Supporting Figure 4. Funnel plot of precision against the effect size (Hedge's g*; n = 3983). Full dataset (A), with the red inset box indicating the data portion shown in B. Data shown within the axis limits of B represent 92.0% of our dataset. In both panels, a blue dashed line is plotted at our meta-analytic mean (-0.11) (Supporting Table 1; Supporting Figure 3), and the solid vertical line is plotted at the origin. The shaded portion of each panel indicates the threshold for statistical significance at α = 0.05. Points are shaded by their density.

Supporting Information 1.6. Meta-regressions: study design

Study duration – Considering the duration of temporal studies (i.e., the length of time in years between the early and recent sampling periods, classified as short: <5 years; medium: 5-30 years; and long: 30+ years), a statistically significant loss of genetic diversity was recorded in studies of the longest duration (predicted Hedge's g* posterior mean = -0.30; HPD CI = -0.38, -0.22) (Supporting Table 2; Figure 2b). This result is unsurprising, as measurable genetic diversity change is predicted to lag behind demographic change (e.g., population decline), suggesting longer-term studies are more likely to identify genetic diversity losses. It is also plausible that longer-term studies are conducted for populations where greater losses are anticipated, e.g., for populations that are threatened. The meta-analytic mean genetic diversity change in medium (predicted Hedge's g* posterior mean = -0.04; HPD = -0.11, 0.02) and short (Hedge's g* posterior mean = -0.06; HPD CI = -0.12, 4.26×10^{-3}) duration studies were also negative, although both 95% HPD CIs overlapped zero.

Statistical method – Statistical methods that recorded temporal genetic diversity change were classified into three alternative study designs commonly used by researchers: 1) linear measures of change over time, 2) two time-point comparisons and 3), coalescent analysis of change (often inferred from genetic data generated at a single sampling time-point). The large sample size excess of the two time-point study effect sizes (see Supporting Table 2), relative to the other two study designs is due in part to our screening and extraction protocol, which facilitated easier extraction of these data formats in cases where a dataset was reported using multiple forms of analysis. A statistically significant loss of genetic diversity was detected regardless of statistical method (HPD CIs did not overlap zero) (Supporting Table 2; Figure 2b). Genetic diversity loss was greatest in studies reporting a linear measure of change (Hedge's g* posterior mean = -0.72; HPD CI = -0.97, -0.46) than in two time-point comparison studies (predicted Hedge's g* posterior mean = -0.08; HPD CI = -0.12, -0.04) or coalescent analysis studies (predicted Hedge's g* posterior mean = -0.33; HPD CI = -0.48, -0.19).

Genetic marker type – The vast majority of data underlying the temporal estimates of genetic diversity were generated using common laboratory-based molecular genotyping techniques (95.4%), with the remainder based on more bespoke methods (such as karyotyping) (3.8%) or genealogy (0.8%) (see Supporting Information 1.3). Due to the large portion of our dataset being generated by microsatellites (n = 3,003 effect sizes from 613 studies in 463 species), we chose this marker type as our reference category (intercept, point of comparison) for meta-regression to compare the predicted mean genetic diversity change recorded with various genetic marker types. Our meta-regression showed a predicted loss of genetic diversity when recorded using microsatellites, although the 95% HPD CIs bounded zero (Hedge's g* posterior mean = -0.05; HPD CI: -0.10, 0.00) (Supporting Table 2; Figure 2b).

Compared to microsatellite data, effect sizes calculated on the basis of other types of molecular data showed significantly greater predicted loss, namely AFLPs (Hedge's g* posterior mean = -0.3; HPD CI = -0.57, -0.01), haplotypes (Hedge's g* posterior mean = -0.37; HPD CI = -0.56, -0.21), nucleotide sequences (Hedge's g* posterior mean = -0.27; HPD CI = -0.39, -0.14), and other data types (Hedge's g* posterior mean = -0.47; HPD CI = -0.67, -0.28). A statistically significant predicted loss of genetic diversity was also seen with SNP data (Hedge's g* posterior mean = -0.18; HPD CI = -0.35, -0.01); the predicted mean for this genetic marker type was not significantly different from the loss observed for our reference category, microsatellites. For the remaining two data types, allozymes and pedigrees, meta-regression predicted a mean loss of genetic diversity, but these were estimated with wide confidence intervals, especially for pedigrees, that overlapped zero (Supporting Table 2).

Genetic diversity metric type – Genetic data were used to calculated genetic diversity metrics which we categorised into four types: 1) population genetic diversity based on counts of unique variants (e.g., allelic richness; referred to as variant counts); 2) population-level genetic diversity based on variant frequencies (e.g., expected heterozygosity; referred to as variant frequencies); 3) population means of individual-level genetic diversity (e.g., observed heterozygosity; referred to as individual-level variation); and 4) integrated statistics (measures of effective population size). The majority of our data was generated using microsatellites (see above and Supporting Information 1.3) for which all four metric types are calculable; the majority of other genetic marker types are also suitable for calculating measures of genetic diversity across most of our four types of genetic diversity metrics.

Meta-regression of these genetic diversity metric types for our full dataset revealed that variant counts, variant frequencies and individual-level variation, on average, report statistically significant loss of genetic diversity (variant counts Hedge's g* posterior mean = -0.09; HPD CI = -0.15, -0.04; variant frequencies Hedge's g* posterior mean = -0.15; HPD CI = -0.20, -0.10; individual-level variation Hedge's g* posterior mean = -0.06; HPD CI = -0.12, -1.79×10^{-5}), with variant frequencies associated with a mean loss of genetic diversity that was of statistically significant greater magnitude than variant counts. For integrated statistics, mean predicted losses were substantively negative, but the 95% HPD intervals bounded zero (integrated statistics Hedge's g* posterior mean = -0.08; HPD CI = -0.17, 2.76×10^{-3}) (Supporting Table 2; Figure 2b).

We calculated a maximum of one effect size per genetic diversity metric type per dataset, although many datasets recorded genetic diversity data across multiple metric types (e.g., reporting both expected and observed heterozygosity). In total, there were 475 temporal studies that reported more than one metric type, with an average of 2.69 metric types reported for each dataset. For those populations where temporal measurements were made with multiple metrics, we examined correlations among diversity metric types. We found that most correlations were weak to moderate (Supporting Table 3). The highest pairwise correlation (0.55) was observed between variant counts and variant frequencies, while the lowest correlation was observed between individual-level variation and integrated statistics (0.25). Correlations among these metrics are not unexpected, as the biological and demographic processes that affect population genetic diversity are likely to covary among study populations and contexts; however, no strong correlations were observed, suggesting the four genetic diversity metric types do capture largely independent aspects of genetic diversity change.

Supporting Table 2. Model summaries of study design variables, including study duration, statistical method, genetic marker type and genetic diversity metric type, describing fixed effects, sample sizes (displayed as number of effect sizes / number of papers / number of species), and effect sizes (modelled and predicted genetic diversity change, i.e., mean Hedge's g* with 95% highest posterior density credible intervals). Effect sizes in bold are significantly different to the intercept.

Fixed effect	Sample size	Modelled effect sizes (95% HPD Cis)	Predicted effect sizes (95% HPD Cis)
Study duration			
Intercept (short, <5 years)	1736 / 345 / 283	-0.06 (-0.12, 4.26 x 10^{-3})	-0.06 (-0.12, 4.26 x 10^{-3})
Generations	3983 / 871 / 622	0.02 (-0.03, 0.06)	-0.04 (-0.12, 0.04)
Medium, 5-30 years	1494 / 358 / 264	0.02 (-0.06, 0.11)	-0.04 (-0.11, 0.02)
Long, 30+ years	753 / 231 / 196	**-0.24 (-0.34, -0.15)**	**-0.30 (-0.38, -0.22)**
Statistical method			
Intercept (linear measure of change)	63 / 27 / 26	-0.72 (-0.97, -0.46)	-0.72 (-0.97, -0.46)
Generations	3983 / 871 / 622	0.01 (-0.04, 0.07)	-0.70 (-0.99, -0.46)
Year midpoint	3983 / 871 / 622	-2.81 x 10^{-3} (-0.07, 0.06)	-0.72 (-0.98, -0.46)
Two time-point comparison	3769 / 773 / 552	**0.64 (0.39, 0.90)**	**-0.08 (-0.12, -0.04)**
Coalescent analysis	151 / 88 / 85	**0.39 (0.11, 0.68)**	**-0.33 (-0.48, -0.19)**
Genetic marker type			
Intercept (microsatellites)	3003 / 613 / 463	-0.05 (-0.10, -4.15 x 10^{-3})	-0.05 (-0.10, -4.15 x 10^{-3})
Generations	3983 / 871 / 622	0.01 (-0.04, 0.07)	-0.03 (-0.1, 0.04)
Year midpoint	3983 / 871 / 622	0.01 (-0.05, 0.07)	-0.04 (-0.12, 0.04)
AFLP	95 / 29 / 30	**-0.25 (-0.49, -0.02)**	**-0.30 (-0.57, -0.10)**
Allozyme	301 / 74 / 74	-0.07 (-0.21, 0.07)	-0.12 (-0.25, 0.03)
Haplotype	104 / 51 / 48	**-0.32 (-0.49, -0.14)**	**-0.37 (-0.56, -0.21)**
Nucleotide	178 / 94 / 97	**-0.22 (-0.36, -0.10)**	**-0.27 (-0.39, -0.14)**
Pedigree	32 / 10 / 4	-0.17 (-0.57, 0.24)	-0.22 (-0.61, 0.20)
SNP	120 / 39 / 34	-0.13 (-0.31, 0.05)	-0.18 (-0.35, -0.01)
Other	150 / 29 / 24	**-0.42 (-0.65, -0.24)**	**-0.47 (-0.67, -0.28)**
Diversity metric type			
Intercept (variant counts)	1019 / 461 / 368	-0.09 (-0.15, -0.04)	-0.09 (-0.15, -0.04)
Generations	3983 / 871 / 622	0.01 (-0.04, 0.07)	-0.08 (-0.15, -0.01)
Year midpoint	3983 / 871 / 622	0.03 (-0.03, 0.09)	-0.06 (-0.14, 0.02)
Variant frequencies	1660 / 655 / 495	**-0.06 (-0.12, -0.01)**	**-0.15 (-0.20, -0.10)**
Individual-level variation	888 / 393 / 308	0.03 (-0.02, 0.09)	-0.06 (-0.12, -1.79 x 10^{-5})
Integrated statistics	416 / 205 / 165	0.01 (-0.09, 0.10)	-0.08 (-0.17, 2.76 x 10^{-3})

Supporting Table 3. Spearman's pairwise correlations (r) between genetic diversity type metrics in publications that reported at least two metrics for the same population (p = significance, n = sample size). Genetic diversity metrics were categorised into four types: 1) population-level genetic diversity based on counting unique variants; 2) population-level genetic diversity metrics that incorporate variant frequencies; 3) population means of individual-level metrics; and 4) integrated statistics. See main methods for a full description of the genetic diversity metric types.

Diversity metric type	r	p	n
1 vs. 2	0.55	1.04 x 10^{-68}	899
1 vs. 3	0.28	2.43 x 10^{-13}	1045
1 vs. 4	0.34	2.40 x 10^{-3}	828
2 vs. 3	0.49	9.94 x 10^{-50}	867
2 vs. 4	0.42	2.09 x 10^{-6}	677
3 vs. 4	0.25	2.58 x 10^{-2}	800

Supporting Information 1.7. Meta-regressions: population context

Realm – Our dataset included nearly all terrestrial and marine biogeographical realms, although the vast majority were from terrestrial realms (90.2%; see Supporting Information 1.2-1.3). We therefore used meta-regression to test whether the mean predicted genetic diversity change varied across these realms. For this analysis, the largest portion of effect sizes were based on data generated for populations in the Palearctic (40.7%), so this realm was taken as our reference category. Effect sizes from the Palearctic followed the overall trend for our full dataset with a statistically significant mean loss of genetic diversity (Hedge's g* posterior mean = -0.13; HPD CI = -0.20, -0.07). Statistically significant losses of genetic diversity were also seen in the Afrotropic (Hedge's g* posterior mean = -0.21; HPD CI = -0.39, -0.04), Nearctic (Hedge's g* posterior mean = -0.09; HPD CI = -0.17, -3.93×10^{-3}), Neotropic (Hedge's g* posterior mean = -0.17; HPD CI = -0.33, -0.03), Oceania (Hedge's g* posterior mean = -0.33; HPD CI = -0.64, -0.01), Central Indo-Pacific (Hedge's g* posterior mean = -0.48; HPD CI = -0.96, -0.02), and Temperate Northern Pacific realms (Hedge's g* posterior mean = -0.37; HPD CI = -0.69, -0.11), although the latter three had poor precision, likely due to a small sample sizes (Supporting Table 4; Figure 2c). There was no evidence that the aforementioned losses were statistically different to those seen in the Palearctic, with the exception of the positive effect sizes seen in the Arctic (Hedge's g* posterior mean = 0.36; HPD CI = -0.14, 0.85), Temperate Northern Atlantic (Hedge's g* posterior mean = 0.12; HPD CI = -0.08, 0.32), and Tropical Atlantic (Hedge's g* posterior mean = 0.23; HPD CI = -0.16, 0.58), which showed a significant mean gain in genetic diversity compared to the Palearctic. In Australasia and Indomalaya, mean predicted effect sizes were close to zero (Australasia Hedge's g* posterior mean = -0.06; HPD CI = -0.24, 0.12; Indomalaya Hedge's g* posterior mean = -0.04; HPD CI = -0.26, 0.20). Despite large sample sizes, these estimates (and the estimate for the Afrotropic realm) lacked precision, suggesting there was substantial variation in measures of genetic diversity change across these three realms. Finally, estimates for the Southern Ocean (Hedge's g* posterior mean = -0.26; HPD CI = -0.72, 0.25) and Temperate Southern Africa marine realms (Hedge's g* posterior mean = 0.07; HPD CI = -0.87, 1.15) also lacked precision, due to small sample sizes.

Taxonomic class – Phylogeny explained a small proportion of the variance in effect sizes across our dataset (see Supporting Information 1.3 and 1.5). Patterns of genetic diversity change were, thus, not correlated with phylogenetic relationships across the tree of life. Next, we tested whether patterns varied across taxonomic classes, as this level of taxonomy has pragmatic use (e.g., for designing species management strategies, clustering research interests, etc.). For the 22 taxonomic classes for which more than 10 effect sizes were obtained, we used Mammalia as our reference category, in which a statistically significant mean loss of genetic diversity was observed (Hedge's g* posterior mean = -0.25; HPD CI = -0.35, -0.17), in line with our overall analysis. The most precise estimates were observed for Mammalia, Aves, Actinopterygii, Insecta and Magnoliopsida, which were the most data- and species-rich taxonomic classes (Figure 2c), while datasets for other classes were smaller and provided estimates that had poor precision (Extended Figure 5a; Supporting Table 4). In addition to Mammalia, a statistically significant mean loss of genetic diversity was observed in two further taxonomic classes: Aves (Hedge's g* posterior mean = -0.43; HPD CI = -0.57, -0.30), and Trematoda (Hedge's g* posterior mean = -0.73; HPD CI = -1.24, -0.24). For Aves, this loss was significantly greater than that seen for Mammalia.

Relative to Mammalia, five taxonomic classes showed significantly less loss of genetic diversity, with mean estimates close to zero or even trending positive (i.e., a mean gain in diversity): Actinopterygii (Hedge's g* posterior mean = 0.03; HPD CI = -0.04, 0.10), Insecta (Hedge's g* posterior mean = 1.87×10^{-3}; HPD CI = -0.12, 0.13), Magnoliopsida (Hedge's g* posterior mean = -0.07; HPD CI = -0.23, 0.09), Liliopsida (Hedge's g* posterior mean = 0.05; HPD CI = -0.20, 0.26), and Peronsporea (Hedge's g* posterior mean = 0.50; HPD CI = -0.14, 1.12) (Supporting

Table 4). For the remaining 14 classes examined, the predicted genetic diversity change was not significantly different from Mammalia (Supporting Table 4).

Next, we performed the taxonomic class meta-regression excluding populations identified as domestic, pests or pathogens, as these contexts differ from other wild species. Where there was sufficient data for modelling, results were similar to the full taxonomic class model, although estimates for Aves and Liliopsida were no longer statistically significantly different from the model intercept, Mammalia (Extended Figure 5a; Supporting Table 4).

Subsetting our dataset to effect sizes calculated only for populations identified as domestic, pests or pathogens encompassed 12 taxonomic classes with 10 effect sizes or more. Each class encompassed either domestic or pest populations (e.g., Mammalia), pest populations (e.g., Gastropoda), or pathogens (e.g., Pucciniomycetes). Interestingly, patterns observed in this dataset were broadly similar to those seen in the analysis of all data subset by taxonomic class (Extended Figure 5a; Supporting Table 4), with a statistically significant mean loss of genetic diversity seen in Mammalia (reference category, Hedge's g* posterior mean = -0.28; HPD CI = -0.53, -0.07), Aves (Hedge's g* posterior mean = -1.06; HPD CI = -1.72, -0.42) and Trematoda (Hedge's g* posterior mean = -0.76; HPD CI = -1.32, -0.16), where the effect in Aves was statistically significant greater loss than seen in Mammalia. All other classes showed mean genetic diversity change that was close to zero, and not statistically different from the reference mean trend seen for Mammalia, except for Peronsporea, a class of fungoid organisms that are plant pathogens, which showed significantly less loss of genetic diversity (Hedge's g* posterior mean = 0.54; HPD CI = -0.25, 1.32; Supporting Table 4). Although the effect size estimate for Peronosporea was positive, the small sample size (14 measurements from 3 species, all identified as pathogens) resulted in large confidence intervals that overlapped zero.

Threat status – We used a coarse approach to recording the threat status of populations examined in our study, assigning each the IUCN Red List category at the species level. We acknowledge that some locally threatened (or locally abundant) populations may be misclassified by this approach, but chose the broader approach as local status of species was not always reported in publications, nor obtainable from publicly available data for the jurisdiction and year that a study was conducted. By this approach, the largest portion of our dataset was from species designated as non-threatened, and this group was therefore our reference category, showing a statistically significant mean predicted loss of genetic diversity (Hedge's g* posterior mean = -0.12; HPD CI = -0.18, -0.07) in line with our overall observations (Figure 2c; Supporting Table 4). Data from species designated as threatened, or of unknown threat status, showed similar losses of genetic diversity (threatened Hedge's g* posterior mean = -0.13; HPD CI = -0.24, -0.04; unknown Hedge's g* posterior mean = -0.09; HPD CI = -0.15, -0.02).

Supporting Table 4. Model summaries of population context variables, including terrestrial realm, taxonomic class (total dataset and subset for domestic, pest or pathogen species only), and threat status (based on IUCN Red List category at the species level), describing fixed effects, sample sizes (displayed as number of effect sizes / number of papers / number of species), and effect sizes (modelled and predicted genetic diversity change, i.e., mean Hedge's g* with 95% highest posterior density credible intervals). Effect sizes in bold are significantly different to the intercept.

Fixed effect	Sample size	Modelled effect sizes (95% HPD CIs)	Predicted effect sizes (95% HPD CIs)
Realm			
Intercept (Terrestrial: Palearctic)	1464 / 362 / 243	-0.13 (-0.20, -0.07)	-0.13 (-0.20, -0.07)
Generations	3954 / 858 / 612	0.01 (-0.04, 0.07)	-0.11 (-0.19, -0.02)
Year midpoint	3954 / 858 / 612	0.02 (-0.04, 0.08)	-0.10 (-0.20, -0.02)
Terrestrial: Afrotropic	187 / 46 / 43	-0.09 (-0.29, 0.08)	-0.21 (-0.39, -0.04)
Terrestrial: Australasia	254 / 49 / 52	0.07 (-0.14, 0.25)	-0.06 (-0.24, 0.12)
Terrestrial: Indomalaya	119 / 26 / 24	0.09 (-0.17, 0.31)	-0.04 (-0.26, 0.2)
Terrestrial: Nearctic	1189 / 226 / 163	0.04 (-0.06, 0.14)	-0.09 (-0.17, -3.93 x 10^{-3})
Terrestrial: Neotropic	340 / 69 / 66	-0.05 (-0.21, 0.12)	-0.17 (-0.33, -0.03)
Terrestrial: Oceania	43 / 13 / 16	-0.20 (-0.51, 0.12)	-0.33 (-0.64, -0.01)
Marine: Arctic	17 / 6 / 5	**0.49 (2.37 x 10^{-3}, 0.99)**	**0.36 (-0.14, 0.85)**
Marine: Central Indo-Pacific	28 / 6 / 6	-0.36 (-0.79, 0.14)	-0.48 (-0.96, -0.02)
Marine: Southern Ocean	21 / 7 / 7	-0.14 (-0.62, 0.36)	-0.26 (-0.72, 0.25)
Marine: Temperate Northern Atlantic	174 / 33 / 30	**0.24 (0.03, 0.46)**	**0.12 (-0.08, 0.32)**
Marine: Temperate Northern Pacific	75 / 17 / 17	-0.24 (-0.53, 0.05)	-0.37 (-0.69, -0.11)
Marine: Temperate Southern Africa	11 / 1 / 1	0.20 (-0.75, 1.29)	0.07 (-0.87, 1.15)
Marine: Tropical Atlantic	32 / 8 / 7	**0.35 (0.01, 0.76)**	**0.23 (-0.16, 0.58)**
Taxonomic class: total			
Intercept (Chordata: Mammalia)	612 / 210 / 134	-0.25 (-0.35, -0.17)	-0.25 (-0.35, -0.17)
Generations	3913 / 844 / 596	1.30 x 10^{-4} (-0.05, 0.05)	-0.25 (-0.37, -0.16)
Year midpoint	3913 / 844 / 596	0.01 (-0.05, 0.07)	-0.24 (-0.35, -0.14)
Chordata: Aves	303 / 82 / 70	**-0.18 (-0.33, -0.03)**	**-0.43 (-0.57, -0.30)**
Chordata: Amphibia	62 / 12 / 12	0.14 (-0.18, 0.46)	-0.11 (-0.42, 0.20)
Chordata: Reptilia	57 / 15 / 13	0.16 (-0.22, 0.49)	-0.09 (-0.46, 0.24)
Chordata: Actinopterygii	1507 / 239 / 131	**0.28 (0.17, 0.39)**	**0.03 (-0.04, 0.10)**
Chordata: Ascidiacea	13 / 5 / 4	0.16 (-0.45, 0.76)	-0.09 (-0.70, 0.51)
Echinodermata: Echinoidea	26 / 4 / 2	0.35 (-0.20, 0.92)	0.10 (-0.42, 0.69)
Mollusca: Bivalvia	66 / 13 / 11	0.06 (-0.28, 0.41)	-0.20 (-0.52, 0.15)
Mollusca: Gastropoda	128 / 19 / 19	0.03 (-0.24, 0.31)	-0.22 (-0.46, 0.05)
Arthropoda: Insecta	461 / 93 / 77	**0.25 (0.11, 0.41)**	**1.86 x 10^{-3} (-0.12, 0.13)**
Arthropoda: Malacostraca	70 / 15 / 15	0.16 (-0.12, 0.54)	-0.09 (-0.41, 0.20)
Arthropoda: Branchiopoda	35 / 11 / 7	0.29 (-0.08, 0.69)	0.03 (-0.30, 0.47)
Arthropoda: Arachnida	23 / 3 / 3	0.42 (-0.30, 1.13)	0.17 (-0.53, 0.89)
Nematoda: Chromadorea	22 / 6 / 5	-0.12 (-0.61, 0.38)	-0.38 (-0.86, 0.12)
Platyhelminthes: Trematoda	15 / 7 / 3	-0.47 (-0.97, 0.05)	-0.73 (-1.24, -0.24)
Cnidaria: Anthozoa	21 / 4 / 5	0.28 (-0.34, 0.83)	0.02 (-0.59, 0.59)
Tracheophyta: Magnoliopsida	315 / 61 / 57	**0.18 (0.01, 0.37)**	**-0.07 (-0.23, 0.09)**
Tracheophyta: Liliopsida	112 / 27 / 15	**0.30 (0.06, 0.54)**	**0.05 (-0.20, 0.26)**
Tracheophyta: Pinopsida	24 / 9 / 6	0.24 (-0.18, 0.68)	-0.01 (-0.46, 0.39)
Gyrista: Phaeophyceae	15 / 1 / 1	0.14 (-0.87, 1.14)	-0.11 (-1.14, 0.85)
Basidiomycota: Pucciniomycetes	12 / 4 / 3	0.57 (-0.05, 1.27)	0.32 (-0.33, 0.96)
Oomycota: Peronosporea	14 / 5 / 3	**0.75 (0.12, 1.37)**	**0.50 (-0.14, 1.12)**

Continued on next page

Fixed effect	Sample size	Modelled effect sizes (95% HPD CIs)	Predicted effect sizes (95% HPD CIs)
Taxonomic class: domestic, pest or pathogen ***excluded***			
Intercept (Chordata: Mammalia)	516 / 166 / 123	-0.25 (-0.33, -0.16)	-0.25 (-0.33, -0.16)
Generations	3181 / 663 / 505	-0.39 (-0.82, 0.07)	-0.64 (-1.10, -0.17)
Year midpoint	3181 / 663 / 505	0.01 (-0.07, 0.10)	-0.24 (-0.36, -0.11)
Chordata: Aves	293 / 77 / 67	-0.14 (-0.29, 0.02)	-0.39 (-0.51, -0.25)
Chordata: Amphibia	62 / 12 / 12	0.12 (-0.17, 0.43)	-0.13 (-0.40, 0.19)
Chordata: Reptilia	57 / 15 / 13	0.17 (-0.13, 0.51)	-0.08 (-0.38, 0.24)
Chordata: Actinopterygii	1419 / 223 / 129	**0.27 (0.16, 0.38)**	**0.02 (-0.05, 0.09)**
Echinodermata: Echinoidea	26 / 4 / 2	0.32 (-0.15, 0.86)	0.08 (-0.45, 0.54)
Mollusca: Bivalvia	28 / 9 / 8	-0.05 (-0.41, 0.36)	-0.30 (-0.66, 0.10)
Mollusca: Gastropoda	79 / 15 / 15	-0.04 (-0.34, 0.22)	-0.29 (-0.55, -0.02)
Arthropoda: Insecta	179 / 41 / 44	**0.23 (0.03, 0.43)**	**-0.02 (-0.19, 0.17)**
Arthropoda: Malacostraca	61 / 13 / 13	0.03 (-0.27, 0.36)	-0.21 (-0.50, 0.12)
Arthropoda: Branchiopoda	35 / 11 / 7	0.28 (-0.08, 0.65)	0.03 (-0.33, 0.38)
Nematoda: Chromadorea	20 / 5 / 4	-0.15 (-0.63, 0.35)	-0.40 (-0.90, 0.05)
Cnidaria: Anthozoa	21 / 4 / 5	0.25 (-0.30, 0.77)	3.38×10^{-3} (-0.57, 0.49)
Tracheophyta: Magnoliopsida	290 / 47 / 46	**0.21 (0.04, 0.42)**	**-0.03 (-0.19, 0.14)**
Tracheophyta: Liliopsida	58 / 12 / 10	0.18 (-0.15, 0.51)	-0.06 (-0.37, 0.26)
Tracheophyta: Pinopsida	22 / 8 / 6	0.25 (-0.24, 0.67)	3.85×10^{-3} (-0.47, 0.42)
Gyrista: Phaeophyceae	15 / 1 / 1	0.14 (-0.72, 1.01)	-0.11 (-0.99, 0.73)
Taxonomic class: domestic, pest or pathogen ***only***			
Intercept (Chordata: Mammalia)	96 / 44 / 14	-0.28 (-0.53, -0.07)	-0.28 (-0.53, -0.07)
Generations	704 / 173 / 97	-0.02 (-0.09, 0.05)	-0.31 (-0.56, -0.05)
Year midpoint	704 / 173 / 97	-0.08 (-0.19, 0.04)	-0.36 (-0.64, -0.07)
Chordata: Aves	10 / 5 / 3	**-0.78 (-1.43, -0.07)**	**-1.06 (-1.72, -0.42)**
Chordata: Actinopterygii	88 / 16 / 9	0.25 (-0.12, 0.64)	-0.03 (-0.35, 0.30)
Mollusca: Bivalvia	38 / 4 / 3	0.18 (-0.52, 0.84)	-0.11 (-0.77, 0.53)
Mollusca: Gastropoda	49 / 5 / 4	0.21 (-0.43, 0.84)	-0.08 (-0.64, 0.52)
Arthropoda: Insecta	282 / 54 / 35	0.30 (-4.11×10^{-3}, 0.61)	0.02 (-0.16, 0.21)
Arthropoda: Arachnida	22 / 2 / 2	0.25 (-0.85, 1.25)	-0.03 (-1.08, 0.96)
Platyhelminthes: Trematoda	14 / 6 / 2	-0.47 (-1.13, 0.16)	-0.76 (-1.32, -0.16)
Tracheophyta: Magnoliopsida	25 / 14 / 12	-0.10 (-0.61, 0.33)	-0.38 (-0.80, 0.03)
Tracheophyta: Liliopsida	54 / 15 / 7	0.39 (-0.05, 0.81)	0.10 (-0.25, 0.46)
Basidiomycota: Pucciniomycetes	12 / 4 / 3	0.61 (-0.24, 1.30)	0.33 (-0.45, 1.03)
Oomycota: Peronosporea	14 / 5 / 3	**0.83 (0.01, 1.66)**	**0.54 (-0.25, 1.32)**
Threat status			
Intercept (non-threatened)	1913 / 414 / 282	-0.12 (-0.18, -0.07)	-0.12 (-0.18, -0.07)
Generations	3983 / 871 / 622	0.01 (-0.04, 0.07)	-0.11 (-0.19, -0.03)
Year midpoint	3983 / 871 / 622	0.03 (-0.04, 0.09)	-0.09 (-0.18, -0.01)
Threatened	544 / 145 / 120	-0.01 (-0.13, 0.10)	-0.13 (-0.24, -0.04)
Unknown	1526 / 321 / 220	0.03 (-0.06, 0.12)	-0.09 (-0.15, -0.02)

Supporting Information 1.8. Meta-regressions: threats

Ecological disturbance – The vast majority of effect sizes recorded in our data were associated with reports of ecological disturbance impacting the study population within the time frame of the temporal analysis (see Supporting Information 1.10, below). To investigate the association between ecological disturbance and genetic diversity change, two meta-regressions of ecological disturbance were fitted: the first to examine the effect of any ecological disturbance (in comparison to studies where no disturbance was recorded), and the second to examine the impact of different types of ecological disturbances. As in our main analysis, a statistically significant estimated mean loss of genetic diversity was recorded regardless of whether a disturbance was reported (no disturbance Hedge's g* posterior mean = -0.09; HPD CI = -0.16, -0.02; any disturbance predicted Hedge's g* posterior mean = -0.12; HPD CI = -0.17, -0.07) (Figure 2d; Supporting Table 5).

To examine the effect of each type of ecological disturbance, and because study populations may be exposed to multiple disturbances (or none), we fitted a meta-regression in which each disturbance is tested in a presence/absence framework. Here, our model intercept (point of comparison) is the absence of any disturbance (i.e., all disturbance variables set to zero, including studies that record no disturbance at all). We found that the absence of disturbance was again associated with a statistically significant loss of genetic diversity (predicted Hedge's g* posterior mean = -0.09; HPD CI = -0.15, -0.02), consistent with our main analysis and with our 'none vs. any' ecological disturbance model (Supporting Table 5). A statistically significant loss of genetic diversity was observed with multiple disturbances: including measurements recorded in populations that were reported to be affected by abiotic natural phenomenon (predicted Hedge's g* posterior mean = -0.16; HPD CI = -0.30, -0.03); disease emergence (predicted Hedge's g* posterior mean = -0.20; HPD CI = -0.41, -4.47×10^{-3}); harvesting/harassment (predicted Hedge's g* posterior mean = -0.09; HPD CI = -0.17, -0.02); and land use change (predicted Hedge's g* posterior mean = -0.18; HPD CI = -0.28, -0.09). No mean change in genetic diversity was observed for measurements reported to be affected by abiotic human phenomenon, ecological disruption, feral plant or animal impact, introgression, invasion, non-conservation introduction or unspecified disturbance, although we further note that none of the recorded disturbance types showed statistically significant differences to the intercept, for which statistically significant loss of genetic diversity was predicted (Figure 2d; Supporting Table 5). Note that ecological disturbance types are defined in Supporting Information 2.5.

Supporting Table 5. Summary of 'threat' meta-regressions, including comparisons between studies where no ecological disturbance was recorded compared to any disturbance, and comparisons across different types of ecological disturbance, describing fixed effects, sample sizes (displayed as number of effect sizes / number of papers / number of species), and effect sizes (modelled and predicted genetic diversity change, i.e., mean Hedge's g* with 95% highest posterior density credible intervals). Effect sizes in bold are significantly different to the intercept (here, all are nonsignificant).

**Fixed effect**	**Sample size**	**Modelled effect sizes (95% HPD CIs)**	**Predicted effect sizes (95% HPD CIs)**
Ecological disturbance: none vs. any			
Intercept (none)	1295 / 293 / 232	-0.09 (-0.16, -0.02)	-0.09 (-0.16, -0.02)
Generations	3983 / 871 / 622	0.01 (-0.04, 0.06)	-0.08 (-0.16, 0.01)
Year midpoint	3983 / 871 / 622	0.03 (-0.03, 0.08)	-0.06 (-0.14, 0.03)
Any disturbance	2688 / 611 / 471	-0.03 (-0.11, 0.05)	-0.12 (-0.17, -0.07)
Ecological disturbances			
Intercept (none)	1295 / 293 / 232	-0.09 (-0.15, -0.02)	-0.09 (-0.15, -0.02)
Generations	3983 / 871 / 622	0.01 (-0.04, 0.06)	-0.08 (-0.16, -3.18×10^{-3})
Year midpoint	3983 / 871 / 622	0.02 (-0.03, 0.09)	-0.06 (-0.15, 0.03)
Abiotic human phenomenon	560 / 121 / 112	0.03 (-0.08, 0.14)	-0.06 (-0.17, 0.06)
Abiotic natural phenomenon	391 / 82 / 83	-0.07 (-0.21, 0.06)	-0.16 (-0.30, -0.03)
Disease emergence	124 / 37 / 33	-0.11 (-0.30, 0.11)	-0.20 (-0.41, -4.47×10^{-3})
Ecological disruption	222 / 47 / 50	0.07 (-0.11, 0.23)	-0.02 (-0.20, 0.16)
Feral plant/animal impact	242 / 61 / 63	-0.05 (-0.19, 0.11)	-0.13 (-0.30, 0.02)
Harvesting/harassment	1246 / 287 / 215	-0.01 (-0.09, 0.08)	-0.09 (-0.17, -0.02)
Introgression	280 / 60 / 51	0.12 (-0.06, 0.27)	0.03 (-0.14, 0.20)
Invasion	51 / 20 / 20	0.14 (-0.14, 0.45)	0.05 (-0.25, 0.36)
Land use change	1030 / 231 / 208	-0.10 (-0.17, 1.22×10^{-3})	-0.18 (-0.28, -0.09)
Non-conservation introduction	24 / 10 / 10	0.03 (-0.34, 0.42)	-0.06 (-0.45, 0.32)
Unspecified	100 / 28 / 28	0.07 (-0.20, 0.29)	-0.01 (-0.25, 0.21)

Supporting Information 1.9. Meta-regressions: conservation management

Conservation management action – The vast majority of effect sizes recorded in our data were associated with reports of conservation management actions impacting the study population within the time frame of the temporal analysis (see Supporting Information 1.11, below). To investigate the association between conservation management action and genetic diversity change, two meta-regressions were fitted. The first analysis compared measurements with any type of conservation management action reported to those with none; there was a statistically significant estimated loss of genetic diversity regardless (no action Hedge's g* posterior mean = -0.10; HPD CI = -0.15, -0.04; any action Hedge's g* posterior mean = -0.12; HPD CI = -0.18, -0.07), with no statistically significant difference between the two groups (Figure 2e; Supporting Table 6). The second conservation management action meta-regression compared measurements with only legal protection recorded as the action type to measurements with any other action, or none. Again, a statistically significant loss of genetic diversity was observed across all three groups: no action (Hedge's g* posterior mean = -0.10; HPD CI = -0.15, -0.04); legal protection only (Hedge's g* posterior mean = -0.17; HPD CI = -0.26, -0.08); and any other action (Hedge's g* posterior mean = -0.10; HPD CI = -0.16, -0.03), and there were no significant differences between groups (Figure 2e; Supporting Table 6). Note that conservation management actions are defined in Supporting Information 2.5.

Conservation management action overall and by IUCN Red List threat status – Next, we fitted a meta-regression to examine all action types reported in the overall dataset, and further on data subsets according to the three threat categories: non-threatened; threatened; and unknown threat status. Conservation management actions that were associated with fewer than 10 effect sizes for a given subset were excluded from the corresponding subset analysis. These models were fitted with each conservation management action tested in a presence/absence framework, because study populations may be exposed to multiple actions (or none). The model intercept (point of comparison) is the absence of any action (i.e., all conservation management action variables set to zero, including studies that record no action at all). In this model, as in the previous two, the absence of conservation management action was associated with a statistically significant mean predicted loss of genetic diversity (Hedge's g* posterior mean = -0.11; HPD CI = -0.15, -0.05) (Figure 2e; Supporting Table 6).

For our overall dataset (i.e., all species, regardless of threat status), a statistically significant mean loss of genetic diversity was predicted for measurements where breeding, legal protection, and/or temporary resources occurred (Extended Figure 5b; Supporting Table 6). Mean estimates of genetic diversity change were also negative (although not statistically significant) for abiotic restoration, conservation introduction, reintroduction, and unspecified conservation actions. No statistically significant mean genetic diversity change was predicted for measurements that occurred alongside ecological restoration, feral and pest control, and/or population control (i.e., estimates were close to zero and not statistically significant), while supplementation was the only conservation management action associated with a statistically significant improvement in genetic diversity (i.e., less loss) relative to no action (Hedge's g* posterior mean = 0.10; HPD CI = -0.02, 0.21).

For non-threatened species, mean predicted effect size estimates for the conservation management actions were generally similar to the overall dataset (Extended Figure 5b; Supporting Table 6); this pattern also held for threatened species, although mean effects for the latter were estimated with lower precision (due to the smaller sample size of 544 effect sizes, versus 3,983 for the full dataset). For threatened species, only legal protection was associated with a statistically significant mean loss of genetic diversity (Hedge's g* posterior mean = -0.24; HPD CI = -0.37; -0.09), and no actions were significantly different to the intercept. A positive effect size estimate for supplementation

observed in the other models was not seen for threatened species, which were the only group to show a negative estimate (predicted Hedge's g* posterior mean = -0.13; HPD CI = -0.44, 0.22) (Extended Figure 5b; Supporting Table 6).

For species with unknown threat status (including unlisted species), breeding was associated with a statistically significant mean predicted genetic diversity change (Hedge's g* posterior mean = -0.33; HPD CI = -0.49, -0.16). Mean effect size estimates for other conservation management actions were in some cases quite different to the overall, threatened and non-threatened species models. For example, effect sizes recorded alongside legal protection showed a significant loss of genetic diversity in all other models, but showed no genetic diversity change in the unknown threat status species (Hedge's g* posterior mean = -0.01; HPD CI = -0.19, 0.16). Unlike other models, the reintroduction effect size for this group was positive, though it was estimated with poor precision (predicted Hedge's g* posterior mean = 0.23; HPD CI = -0.26, 0.69) and was not significantly different to the intercept (Extended Figure 5b; Supporting Table 6).

Conservation management intensity – For those contexts where conservation management actions were reported, we classified the level of intensity of these actions, based on author descriptions. Our three levels were: 1) individual (most intensive), 2) population, 3) indirect (least intensive) and 4) mixed (detailed at Supporting Information 2.5). Where multiple conservation management actions were reported for a given measurement, the highest management intensity level that applied was recorded. Effect sizes associated with no action (or unknown) were excluded from this analysis.

We observed that mean predicted measures of genetic diversity change varied according to the level of conservation management intensity during the study period, with higher intensity management coinciding with populations reporting the greatest loss of genetic diversity. Populations reportedly managed at the individual and population level showed statistically significant negative mean predicted genetic diversity (individual Hedge's g* posterior mean = -0.29; HPD CI = -0.44, -0.16; population Hedge's g* posterior mean = -0.11; HPD CI = -0.17; -0.02), and significantly less loss was seen in populations receiving population-level management than individual-level management (Figure 2e; Supporting Table 6). For populations where indirect or mixed management was reported, the mean predicted genetic diversity change was close to zero, with a wide confidence interval (indirect Hedge's g* posterior mean = -0.03; HPD CI = -0.24; 0.19; mixed Hedge's g* posterior mean = -4.67×10^{-3}; HPD CI = -0.33; 0.33), with the former significantly different from individual management where greatest loss was seen (Figure 2e; Supporting Table 6).

Conservation management action by taxonomic class – We further investigated associations between conservation management actions and genetic diversity change in the five most data and species rich taxonomic classes in our dataset: Actinopterygii, Mammalia, Insecta, Magnoliopsida, and Aves. Unlike our overall conservation management actions model, where the absence of actions was associated with a statistically significant loss of genetic diversity, we saw mixed predictions for our five most data-rich taxonomic classes. For Mammalia and Aves, a statistically significant mean loss of genetic diversity was predicted in the absence of conservation action, similar to our main analysis (Mammalia Hedge's g* posterior mean = -0.28; HPD CI = -0.41; -0.09; Aves Hedge's g* posterior mean = -0.56; HPD CI = -0.90, -0.27) (Extended Figure 5c; Supporting Table 6). However, for Actinopterygii, Insecta and Magnoliopsida, no mean genetic diversity change was predicted in the absence of conservation management action (Actinopterygii Hedge's g* posterior mean = -0.03; HPD CI = -0.11, 0.05; Insecta Hedge's g* posterior mean = -0.03; HPD CI = -0.12, 0.07; Magnoliopsida Hedge's g* posterior mean = 0.01; HPD CI = -0.11, 0.15) (Extended Figure 5c; Supporting Table 6).

For Mammalia, similar to the overall conservation management model, a statistically significant loss of genetic diversity was observed for measurements associated with breeding and legal

protection (Extended Figure 5c; Supporting Table 6). No statistically significant genetic diversity change was observed where abiotic restoration, conservation introduction, ecological restoration/supplementation (combined variable), feral and pest control, population control, reintroduction, temporary resources or when unspecified actions were reported, noting that most estimated effect sizes were negative, although feral and pest control and population control were close to zero, while estimates for conservation introduction and unspecified actions were positive. No conservation management actions were significantly different to the intercept.

For Aves, a statistically significant mean loss of genetic diversity was predicted for measurements where breeding, legal protection and unspecified actions were reported (Extended Figure 5c; Supporting Table 6). No statistically significant genetic diversity change was observed across measures where ecological restoration/feral and pest control (combined variable), reintroduction, or temporary resources were reported. Mean predicted genetic diversity change with reintroduction and temporary resources was strongly negative, though estimated with poor precision, while for ecological restoration/feral and pest control the mean predicted genetic diversity change was close to zero. Significantly more genetic diversity was predicted where supplementation occurred, relative to no action, and while the mean predicted genetic diversity change with supplementation was positive, the 95% CI bounded zero (predicted Hedge's g* posterior mean = 0.66; HPD CI = -0.03, 1.40).

For Actinopterygii, meta-regression predicted no statistically significant mean genetic diversity change in association with any of the reported conservation management actions: abiotic restoration, breeding, ecological restoration, feral and pest control, legal protection, supplementation, temporary resources nor when unspecified actions were reported (Extended Figure 5c; Supporting Table 6). However, in this dataset year midpoint was a significant positive moderator of genetic diversity change relative to the intercept, and a statistically significant increase in genetic diversity was observed for this variable (predicted Hedge's g* posterior mean = 0.92; HPD CI = 1.44×10^{-3}, 1.97). This result suggests that, as studies become more recent, they are more likely to report an increase in genetic diversity, although this effect was estimated with poor precision.

For Insecta, few conservation management actions were reported (Extended Figure 5c; Supporting Table 6), such that sufficient data for meta-regression were only available for breeding (17 measurements across 5 species), legal protection (35 measurements across 11 species), and where unspecified conservation management actions were reported (10 measurements across 3 species). All three actions showed no statistically significant change in genetic diversity, with negative effect sizes that were close to zero.

Similar to Insecta, only three conservation management actions had sufficient data for modelling in Magnoliopsida (Extended Figure 5c; Supporting Table 6): breeding (23 measurements across 13 species), conservation introduction (72 measurements across 2 species) and legal protection (44 measurements across 11 species). While conservation introduction and legal protection showed no statistically significant change in genetic diversity, with negative effect sizes that were close to zero, a significant loss of genetic diversity was predicted for breeding, which was also significantly lower (i.e., greater loss) than with no management (Hedge's g* posterior mean = -0.34; HPD CI = -0.66, -0.06).

Supporting Table 6. Summary of conservation management meta-regressions, including comparisons between studies where no conservation management action was recorded compared to any action, no management action compared legal protection and any other action, comparisons across different types of management actions (for the total dataset, for subsets of species based on threat status, and across the five most data-rich classes [Mammalia, Aves, Actinopterygii, Insecta and Magnoliopsida]), and management intensity, describing fixed effects, sample sizes (displayed as number of effect sizes / number of papers / number of species), and effect sizes (modelled and predicted genetic diversity change, i.e., mean Hedge's g* with 95% highest posterior density credible intervals). Effect sizes in bold are significantly different to the intercept. Correlated actions were combined for taxon-specific meta-regressions (see Supporting Information 2.12).

**Fixed effect**	**Sample size**	**Modelled effect sizes (95% HPD CIs)**	**Predicted effect sizes (95% HPD CIs)**
Management action: none vs. any			
Intercept (none)	2224 / 456 / 386	-0.10 (-0.15, -0.04)	-0.10 (-0.15, -0.04)
Generations	3983 / 871 / 622	0.01 (-0.04, 0.07)	-0.09 (-0.16, -4.27 x 10^{-3})
Year midpoint	3983 / 871 / 622	0.03 (-0.03, 0.09)	-0.07 (-0.15, 0.01)
Any action	1759 / 438 / 302	-0.02 (-0.10, 0.06)	-0.12 (-0.18, -0.07)
Management action: none vs. legal protection vs. any (other)			
Intercept (none)	2224 / 456 / 386	-0.10 (-0.15, -0.04)	-0.10 (-0.15, -0.04)
Generations	3983 / 871 / 622	0.01 (-0.04, 0.07)	-0.09 (-0.16, -4.37 x 10^{-3})
Year midpoint	3983 / 871 / 622	0.03 (-0.04, 0.09)	-0.07 (-0.14, 0.01)
Legal protection	577 / 153 / 140	-0.07 (-0.17, 0.04)	-0.17 (-0.26, -0.08)
Any (other) action	1182 / 300 / 193	-0.10 (-0.15, -0.04)	-0.10 (-0.16, -0.03)
Management action by threat status: total			
Intercept (none)	3983 / 871 / 622	-0.11 (-0.15, -0.05)	-0.11 (-0.15, -0.05)
Generations	3983 / 871 / 622	0.02 (-0.04, 0.07)	-0.09 (-0.17, -0.02)
Year midpoint	3983 / 871 / 622	0.02 (-0.04, 0.08)	-0.09 (-0.17, -0.01)
Abiotic restoration	63 / 17 / 16	0.01 (-0.31, 0.29)	-0.09 (-0.41, 0.19)
Breeding	320 / 121 / 74	-0.12 (-0.24, 0.01)	-0.22 (-0.35, -0.11)
Conservation introduction	98 / 10 / 10	-0.02 (-0.26, 0.20)	-0.13 (-0.34, 0.11)
Ecological restoration	135 / 31 / 28	0.10 (-0.14, 0.35)	-0.01 (-0.26, 0.24)
Feral and pest control	87 / 22 / 23	0.06 (-0.22, 0.31)	-0.04 (-0.31, 0.21)
Legal protection	978 / 220 / 185	-0.06 (-0.14, 0.05)	-0.16 (-0.25, -0.08)
Population control	39 / 14 / 13	0.20 (-0.12, 0.54)	0.09 (-0.25, 0.42)
Reintroduction	98 / 32 / 29	-4.69 x 10^{-3} (-0.23, 0.19)	-0.11 (-0.31, 0.10)
Supplementation	453 / 96 / 56	**0.20 (0.09, 0.32)**	**0.10 (-0.02, 0.21)**
Temporary resources	131 / 41 / 38	-0.08 (-0.28, 0.12)	-0.18 (-0.40, -3.41 x 10^{-3})
Unspecified	103 / 26 / 25	-0.05 (-0.28, 0.20)	-0.16 (-0.40, 0.07)
Management action by threat status: non-threatened			
Intercept (none)	1913 / 414 / 282	-0.13 (-0.22, -0.05)	-0.13 (-0.22, -0.05)
Generations	1913 / 414 / 282	0.14 (-0.04, 0.31)	4.84 x 10^{-3} (-0.20, 0.19)
Year midpoint	1913 / 414 / 282	0.23 (-0.03, 0.46)	0.10 (-0.16, 0.35)
Abiotic restoration	39 / 11 / 10	0.05 (-0.35, 0.41)	-0.08 (-0.47, 0.30)
Breeding	104 / 41 / 29	-0.03 (-0.24, 0.19)	-0.16 (-0.37, 0.07)
Ecological restoration	81 / 16 / 15	0.13 (-0.23, 0.50)	-1.54 x 10^{-3} (-0.40, 0.36)
Feral and pest control	50 / 14 / 15	0.08 (-0.29, 0.41)	-0.06 (-0.41, 0.29)
Legal protection	509 / 115 / 89	-0.04 (-0.19, 0.08)	-0.18 (-0.29, -0.04)
Population control	23 / 8 / 7	0.09 (-0.43, 0.55)	-0.04 (-0.55, 0.43)
Reintroduction	54 / 19 / 17	-0.03 (-0.33, 0.28)	-0.16 (-0.48, 0.14)
Supplementation	284 / 55 / 30	**0.21 (0.06, 0.36)**	**0.08 (-0.07, 0.24)**
Temporary resources	44 / 15 / 16	-0.05 (-0.41, 0.32)	-0.18 (-0.55, 0.18)
Unspecified	77 / 17 / 15	-0.06 (-0.42, 0.24)	-0.20 (-0.51, 0.13)

Fixed effect	Sample size	Modelled effect sizes (95% HPD CIs)	Predicted effect sizes (95% HPD CIs)
Continued on next page			
Management action by threat status: threatened			
Intercept (none)	544 / 145 / 120	-0.13 (-0.26, 0.03)	-0.13 (-0.26, 0.03)
Generations	544 / 145 / 120	-0.22 (-1.08, 0.69)	-0.35 (-1.19, 0.63)
Year midpoint	544 / 145 / 120	0.02 (-0.11, 0.14)	-0.11 (-0.30, 0.10)
Abiotic restoration	16 / 3 / 3	-0.01 (-0.84, 0.67)	-0.14 (-0.89, 0.60)
Breeding	42 / 16 / 13	0.07 (-0.25, 0.40)	-0.06 (-0.36, 0.26)
Conservation introduction	14 / 4 / 4	0.08 (-0.39, 0.52)	-0.04 (-0.49, 0.46)
Ecological restoration	35 / 8 / 7	0.23 (-0.28, 0.77)	0.10 (-0.50, 0.62)
Feral and pest control	13 / 5 / 5	0.07 (-0.52, 0.64)	-0.05 (-0.68, 0.51)
Legal protection	259 / 66 / 61	-0.11 (-0.29, 0.09)	-0.24 (-0.37, -0.09)
Reintroduction	31 / 9 / 9	-0.08 (-0.42, 0.25)	-0.21 (-0.57, 0.14)
Supplementation	46 / 15 / 12	-0.01 (-0.31, 0.30)	-0.13 (-0.44, 0.22)
Temporary resources	18 / 7 / 7	0.04 (-0.42, 0.48)	-0.08 (-0.54, 0.38)
Unspecified	25 / 9 / 9	-0.04 (-0.42, 0.4)	-0.16 (-0.50, 0.24)
Management action by threat status: unknown			
Intercept (none)	1526 / 321 / 220	-0.06 (-0.14, 0.02)	-0.06 (-0.14, 0.02)
Generations	1526 / 321 / 220	0.01 (-0.06, 0.07)	-0.05 (-0.14, 0.06)
Year midpoint	1526 / 321 / 220	-0.05 (-0.14, 0.04)	-0.10 (-0.22, 0.02)
Breeding	174 / 65 / 32	**-0.27 (-0.44, -0.09)**	**-0.33 (-0.49, -0.16)**
Conservation introduction	78 / 4 / 4	-0.03 (-0.31, 0.27)	-0.08 (-0.38, 0.18)
Ecological restoration	19 / 7 / 6	0.15 (-0.30, 0.64)	0.09 (-0.37, 0.55)
Feral and pest control	24 / 3 / 3	-0.02 (-0.7, 0.58)	-0.08 (-0.74, 0.50)
Legal protection	210 / 42 / 35	0.05 (-0.14, 0.23)	-0.01 (-0.19, 0.16)
Population control	11 / 4 / 4	0.57 (-0.04, 1.17)	0.51 (-0.10, 1.14)
Reintroduction	13 / 4 / 3	0.28 (-0.15, 0.79)	0.23 (-0.26, 0.69)
Supplementation	123 / 26 / 14	0.09 (-0.11, 0.33)	0.04 (-0.18, 0.27)
Temporary resources	69 / 20 / 15	-0.10 (-0.38, 0.22)	-0.15 (-0.45, 0.16)
Management intensity			
Intercept (individual)	233 / 96 / 57	-0.29 (-0.44, -0.16)	-0.29 (-0.44, -0.16)
Generations	1819 / 447 / 309	0.01 (-0.05, 0.07)	-0.28 (-0.44, -0.14)
Year midpoint	1819 / 447 / 309	0.02 (-0.06, 0.09)	-0.27 (-0.42, -0.11)
Population	1366 / 302 / 230	**0.18 (0.04, 0.34)**	**-0.11 (-0.17, -0.02)**
Indirect	165 / 42 / 43	**0.26 (0.02, 0.52)**	**-0.03 (-0.24, 0.19)**
Mixed	55 / 20 / 19	0.28 (-0.11, 0.60)	-4.67×10^{-3} (-0.33, 0.33)
Management action by class: Mammalia			
Intercept (no action)	612 / 210 / 134	-0.28 (-0.41, -0.09)	-0.28 (-0.41, -0.09)
Generations	612 / 210 / 134	-0.19 (-0.87, 0.49)	-0.47 (-1.14, 0.29)
Year midpoint	612 / 210 / 134	-0.06 (-0.21, 0.07)	-0.33 (-0.58, -0.13)
Abiotic restoration	26 / 4 / 4	-2.89×10^{-3} (-0.65, 0.55)	-0.28 (-0.89, 0.31)
Breeding	94 / 40 / 17	-0.03 (-0.30, 0.21)	-0.30 (-0.53, -0.06)
Conservation introduction	13 / 4 / 4	0.37 (-0.14, 0.90)	0.10 (-0.46, 0.62)
Ecological restoration & Supplementation	40 / 12 / 10	0.05 (-0.30, 0.43)	-0.23 (-0.66, 0.12)
Feral and pest control	17 / 5 / 5	0.17 (-0.46, 0.82)	-0.11 (-0.84, 0.48)
Legal protection	270 / 82 / 66	-0.06 (-0.26, 0.15)	-0.34 (-0.51, -0.18)
Population control	20 / 7 / 5	0.23 (-0.31, 0.80)	-0.04 (-0.56, 0.53)
Reintroduction	53 / 16 / 14	0.12 (-0.20, 0.43)	-0.16 (-0.50, 0.19)
Temporary resources	36 / 12 / 10	-4.97×10^{-3} (-0.47, 0.41)	-0.28 (-0.72, 0.20)
Unspecified	28 / 10 / 9	0.45 (-0.02, 0.94)	0.18 (-0.26, 0.65)

**Fixed effect**	**Sample size**	**Modelled effect sizes (95% HPD CIs)**	**Predicted effect sizes (95% HPD CIs)**
Continued on next page			
Management action by class: Aves			
Intercept (no action)	303 / 82 / 70	-0.56 (-0.90, -0.27)	-0.56 (-0.90, -0.27)
Generations	303 / 82 / 70	-0.39 (-4.93, 4.16)	-0.95 (-5.49, 3.82)
Year midpoint	303 / 82 / 70	0.05 (-0.69, 0.82)	-0.51 (-1.42, 0.31)
Breeding	20 / 7 / 5	-0.56 (-1.37, 0.30)	-1.12 (-1.96, -0.30)
Ecological restoration & Feral and pest control	38 / 12 / 13	0.48 (-0.13, 1.04)	-0.08 (-0.66, 0.54)
Legal protection	117 / 28 / 26	-0.01 (-0.53, 0.41)	-0.58 (-0.96, -0.12)
Reintroduction	23 / 6 / 6	-0.20 (-1.08, 0.71)	-0.77 (-1.65, 0.09)
Supplementation	27 / 10 / 7	**1.22 (0.60, 1.93)**	**0.66 (-0.03, 1.40)**
Temporary resources	15 / 7 / 8	-0.02 (-0.66, 0.79)	-0.58 (-1.26, 0.18)
Unspecified	28 / 7 / 7	-0.29 (-1.05, 0.43)	-0.85 (-1.52, -0.16)
Management action by class: Actinopterygii			
Intercept (no action)	1507 / 239 / 131	-0.03 (-0.11, 0.05)	-0.03 (-0.11, 0.05)
Generations	1507 / 239 / 131	-2.73 x 10^{-3} (-2.33, 2.35)	-0.04 (-2.37, 2.35)
Year midpoint	1507 / 239 / 131	**0.95 (0.08, 1.97)**	**0.92 (1.44 x 10^{-3}, 1.99)**
Abiotic restoration	22 / 7 / 6	-0.08 (-0.47, 0.31)	-0.11 (-0.49, 0.30)
Breeding	70 / 26 / 14	-0.03 (-0.18, 0.16)	-0.06 (-0.26, 0.10)
Ecological restoration	52 / 7 / 7	0.02 (-0.31, 0.34)	-0.02 (-0.37, 0.31)
Feral and pest control	31 / 4 / 4	-0.13 (-0.47, 0.26)	-0.16 (-0.53, 0.19)
Legal protection	425 / 61 / 48	0.04 (-0.08, 0.16)	0.01 (-0.11, 0.12)
Supplementation	345 / 65 / 30	0.08 (-0.01, 0.18)	0.05 (-0.05, 0.16)
Temporary resources	37 / 9 / 7	-0.14 (-0.42, 0.11)	-0.18 (-0.47, 0.08)
Unspecified	37 / 9 / 7	-0.01 (-0.35, 0.35)	-0.04 (-0.38, 0.31)
Management action by class: Insecta			
Intercept (no action)	461 / 93 / 77	-0.03 (-0.12, 0.07)	-0.03 (-0.12, 0.07)
Generations	461 / 93 / 77	0.17 (-0.10, 0.49)	0.15 (-0.10, 0.41)
Year midpoint	461 / 93 / 77	0.54 (-0.45, 1.44)	0.51 (-0.41, 1.41)
Breeding	17 / 7 / 5	0.02 (-0.37, 0.32)	-0.01 (-0.35, 0.35)
Legal protection	35 / 13 / 11	-0.02 (-0.26, 0.29)	-0.05 (-0.33, 0.20)
Unspecified	10 / 3 / 3	-0.06 (-0.62, 0.48)	-0.09 (-0.61, 0.48)
Management action by class: Magnoliopsida			
Intercept (no action)	315 / 61 / 57	0.01 (-0.11, 0.15)	0.01 (-0.11, 0.15)
Generations	315 / 61 / 57	-0.04 (-0.14, 0.06)	-0.03 (-0.20, 0.17)
Year midpoint	315 / 61 / 57	-0.09 (-0.23, 0.05)	-0.08 (-0.33, 0.12)
Breeding	23 / 14 / 13	**-0.35 (-0.69, -0.05)**	**-0.34 (-0.66, -0.06)**
Conservation introduction	72 / 2 / 2	-0.19 (-0.48, 0.09)	-0.17 (-0.42, 0.09)
Legal protection	44 / 11 / 11	-0.15 (-0.38, 0.13)	-0.13 (-0.35, 0.08)

Supporting Information 1.10. Systematic review dataset summary: threats

Overview of ecological disturbance across entire dataset – At least one type of ecological disturbance was reported for 65.11% of unique populations in our systematic review data, within the temporal time frame of the reported study, with harvesting/harassment being the most commonly reported disturbance (29.34%), followed by land use change (26.01%), and abiotic human phenomenon (13.56%) (Extended Figure 6). Our protocol for classifying ecological disturbance, generated via iterative consultation and validation amongst our research team, identified 10 largely independent categories of ecological disturbance (plus a category for "unspecified"): although some classifications showed statistically significant correlations, all correlations were negligible ($r \leq |0.24|$) (Extended Figure 6). It is not unexpected that our dataset reports co-occurring ecological disturbances within studies, as many species, populations and habitats face multiple, complex disturbances. Ordination plots were used to characterise variation in ecological disturbance across our dataset as a whole. Harvesting/harassment, land use change and, to a lesser extent, abiotic natural phenomenon described the greatest portion of variation in the data, mostly consistent with the high occurrence of these disturbances in our dataset (Supporting Table 7). Finally, there were no clear patterns in the types of ecological disturbance reported for threatened versus non-threatened species (here non-threatened includes unlisted and Data Deficient species) (Extended Figure 6). The occurrence of any ecological disturbance was more common for threatened species (82.33% of unique populations) than for non-threatened species (62.38% of unique populations).

Supporting Table 7. Loadings matrix for principal component axes one to ten, and the standard deviation and variance explained across the ten axes, based on ordination of the variation in ecological disturbance across our dataset as a whole.

Ecological disturbance	PC1	PC2	PC3	PC4	PC5	PC6	PC7	PC8	PC9	PC10
Abiotic human phenomenon	0.14	0.11	0.94	0.2	-0.09	0.07	-0.19	-0.03	-0.02	-0.01
Abiotic natural phenomenon	0.09	-0.36	-0.15	0.72	-0.2	0.48	0.2	-0.1	-0.04	0
Disease emergence	0.02	-0.01	-0.02	-0.01	0.02	-0.19	-0.01	-0.98	-0.05	-0.01
Ecological disruption	0.03	0.02	0.22	-0.04	0.19	-0.16	0.94	0.03	-0.03	-0.01
Feral plant/animal impact	0.12	0.01	0.03	-0.13	0.81	0.54	-0.09	-0.08	-0.04	0.02
Harvesting/harassment	-0.76	0.56	0	0.14	-0.06	0.28	0.07	-0.07	-0.06	0
Introgression	0.04	-0.16	0.09	-0.63	-0.48	0.56	0.15	-0.11	0	0.04
Invasion	0	-0.06	-0.01	-0.04	-0.02	-0.06	-0.04	0.07	-0.99	0.09
Land use change	0.62	0.72	-0.21	0.08	-0.16	0.13	0.07	-0.02	-0.05	0
Non-conservation introduction	0	-0.01	-0.01	-0.03	-0.01	0.03	0	0.01	-0.09	-0.99
**Standard deviation**	0.54	0.48	0.42	0.35	0.3	0.28	0.26	0.19	0.15	0.11
**Proportion of Variance**	0.26	0.2	0.15	0.11	0.08	0.07	0.06	0.03	0.02	0.01
**Cumulative Proportion**	0.26	0.47	0.62	0.73	0.81	0.88	0.94	0.97	0.99	1

Ecological disturbance across taxonomic classes – Ecological disturbance was reported across 29 taxonomic classes of organisms included in our dataset (Extended Figure 7). We note that although we have referred to these data as "ecological disturbance", we took a broad definition including phenomena that represent intentional or unintentional actions by humans, or extreme natural events (beyond natural fluctuations), that are expected to worsen conditions for a focal population (see Methods). The five taxonomic classes with the most data across the different types of ecological disturbances were: Mammalia, Aves, Actinopterygii, Insecta, and Magnoliopsida. As in our main dataset, abiotic human phenomenon and harvesting/harassment were the most commonly reported ecological disturbances across the majority of classes (Figure 3). For Mammalia and Actinopterygii, harvesting/harassment, followed by land use change, were the most reported disturbances.

Similarly, for Aves, Insecta, and Magnoliopsida, land use change ranked as the most common ecological disturbance.

Breaking down our dataset by taxonomic class revealed some correlations among ecological disturbances (Supporting Figure 5), although these relationships varied among classes. For example, feral plant or animal impact and harvesting/harassment were positively correlated in Insecta ($r = 0.51$, $p < 0.001$) and Magnoliopsida ($r = 0.36$, $p < 0.001$), but negatively correlated in Actinopterygii ($r = -0.46$, $p < 0.001$). For Magnoliopsida, feral plant or animal impact also showed a weak positive correlation with introgression ($r = 0.34$, $p < 0.001$) and abiotic natural phenomena ($r = 0.31$, $p < 0.01$), while non-conservation introductions were weakly correlated with harvesting/harassment ($r = 0.37$, $p < 0.001$). Ecological disruption showed a weak positive correlation with disease emergence in Aves ($r = 0.32$, $p < 0.001$), and a strong positive correlation with abiotic human phenomenon in Insecta ($r = 0.79$, $p < 0.001$). Weak negative correlations were found between land use change and abiotic natural phenomena in Magnoliopsida ($r = -0.45$, $p < 0.001$) and Aves ($r = -0.32$, $p < 0.001$), and between land use change and harvesting/harassment in Insecta ($r = -0.30$, $p < 0.05$). Introgression was correlated with a number of disturbances, including invasion for Mammalia ($r = 0.33$, $p < 0.001$), land use change for Insecta ($r = -0.54$, $p < 0.001$), and non-conservation introductions for Magnoliopsida ($r = 0.57$, $p < 0.001$).

For this analysis (and ordination, below), data generated from populations identified as domestic, and pathogens or pests were analysed separately, as intentional disturbances may differ in their goals for these contexts, as compared to wild populations in general. For example, “harvesting/harassment” of pests and pathogens is likely to have an intentional population suppression or eradication goal, as compared to sustainable harvest of wildlife. For those contexts identified as domestic populations, introgression was negatively correlated with harvesting/harassment ($r = -0.37$, $p = 0.01$) and ecological disruption ($r = -0.32$, $p < 0.01$). For pest or pathogen species, introgression also showed a weak negative correlation with harvesting/harassment ($r = -0.36$, $p < 0.001$), while abiotic human phenomenon was positively correlated with introgression ($r = 0.37$, $p < 0.001$) and ecological disruption ($r = 0.38$, $p < 0.001$).

Ordination of the variation in ecological disturbance showed that land use change explained the most variation in these variables across our five major classes (Supporting Figure 5), alongside harvesting/harassment (Mammalia), feral plant or animal impact and harvesting/harassment (Aves), harvesting/harassment and abiotic human phenomenon (Actinopterygii), abiotic human phenomenon (Insecta), and abiotic natural phenomenon (Magnoliopsida). Within the top five taxonomic classes in our study, there were no clear patterns in the ecological disturbances reported for threatened versus non-threatened species (here non-threatened includes unlisted and Data Deficient species). For contexts identified as domestic populations, introgression and harvesting/harassment explained most of the variability in the ecological disturbance data, compared to harvesting/harassment and abiotic natural phenomenon for those identified as pests and pathogens (Supporting Figure 5).

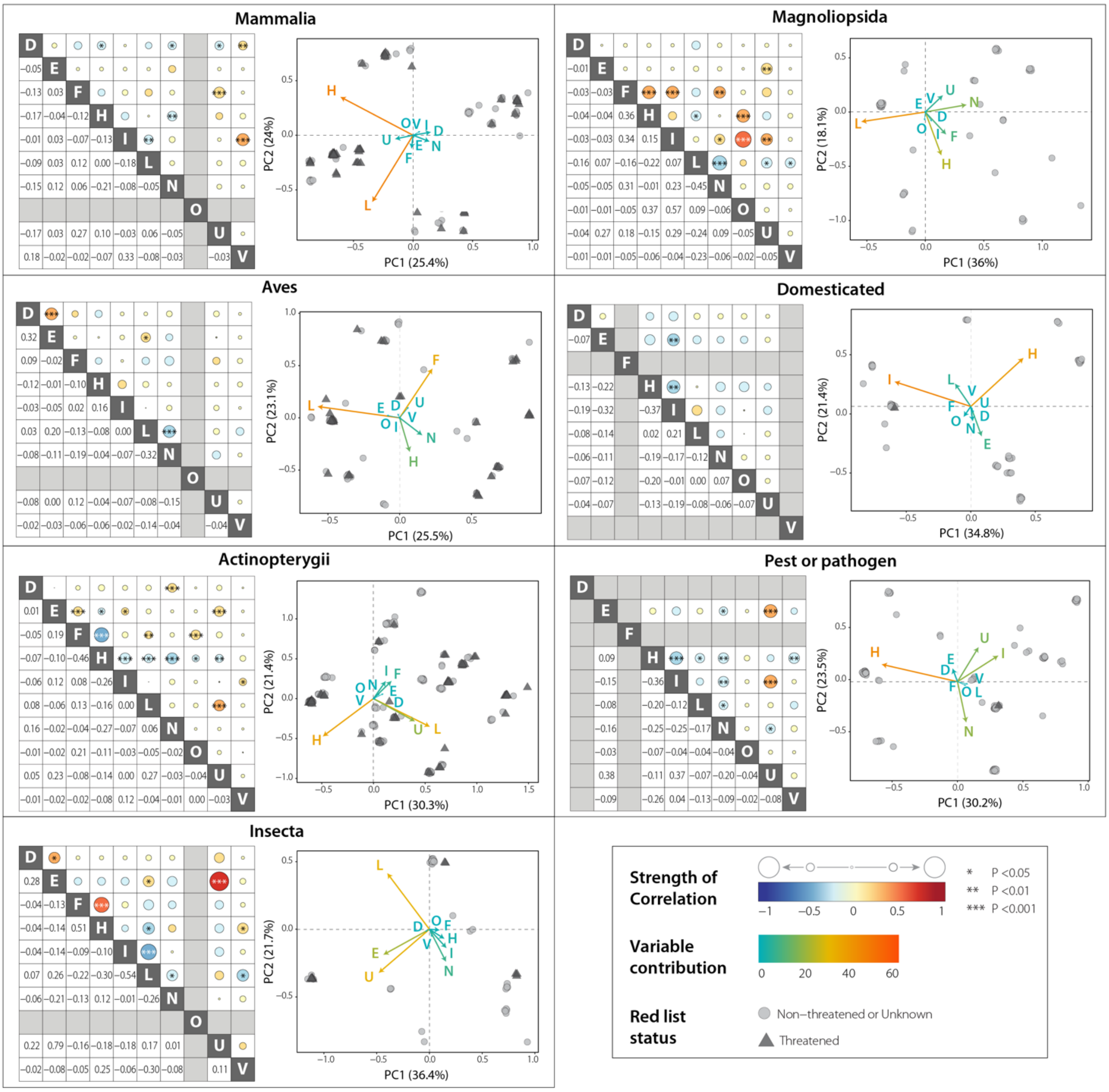

Supporting Figure 5. Ecological disturbances reported for unique populations of species per unique study, across the five most data and species rich taxonomic classes (Mammalia, Aves, Actinopterygii, Insecta, and Magnoliopsida), domestic species and pest or pathogen species, including correlations between disturbance types and PCA biplots. The lower half of the correlation plot shows the correlation coefficient, the upper half shows the strength of correlation represented by color and size, the asterisks show significance, and greyed out actions were not recorded in the data subset. In the PCA biplots, point color and shape represent IUCN Red List threat status, and arrow and letter color represent the variable contribution to the PCA. Letters in plots represent disturbance types (D = disease emergence; E = ecological disruption; F = feral plant/animal impact; H = harvesting/harassment; I = introgression; L = land use change; N = abiotic natural phenomenon; O = non-conservation introduction; U = abiotic human phenomenon; V = invasion).

Supporting Information 1.11. Systematic review dataset summary: conservation management

Overview of conservation management across entire dataset – Conservation management actions were reported within the temporal time frame of a lower proportion of studies in our dataset compared to ecological disturbance, with at least one type of management action reported for 45.75% of unique populations in our systematic review dataset (Extended Figure 6). The most commonly reported action was legal protection (23.02%), followed by supplementation (10.28%) and breeding (9.70%), and ordination methods revealed that these actions explained the most variation in conservation action across our dataset (Extended Figure 6; Supporting Table 8). Our classification of conservation management actions, generated via expert discussion as for our ecological disturbance categories, identified 10 largely independent categories of conservation management action (plus a category for unspecified). Although some classifications showed statistically significant correlations, most correlations were negligible ($r \leq |0.25|$) (Extended Figure 6). One exception was a statistically significant negative correlation ($r = -0.41$, $p < 0.001$) between breeding and legal protection, suggesting that breeding is reported less often when species or populations are legally protected. However, this correlation was weak and both variables were retained in further analyses. As for ecological disturbance, correlations among conservation management actions are not unexpected, as many threatened species and habitats face complex threats that are mitigated by multidisciplinary approaches (such as ecological restoration alongside translocations). Finally, there were no clear patterns in the types of conservation management action reported for threatened versus non-threatened species (here non-threatened includes unlisted and Data Deficient species) (Extended Figure 6). The occurrence of any conservation management action was more common for threatened species (66.78% of unique populations) than for non-threatened species (42.38% of unique populations).

Supporting Table 8. Loadings matrix for principal component axes one to ten, and the standard deviation and variance explained across the ten axes, based on ordination of the variation in conservation management across our dataset as a whole.

**Management actions**	**PC1**	**PC2**	**PC3**	**PC4**	**PC5**	**PC6**	**PC7**	**PC8**	**PC9**	**PC10**
Reintroduction	0.01	0	-0.02	0	-0.15	-0.69	0.41	0.57	-0.13	0
Supplementation	0.05	0.96	0.15	0.12	0	0.11	0.14	0.02	-0.08	0.03
Conservation introduction	-0.1	-0.09	-0.55	0.1	-0.01	0.44	0.63	0	-0.25	0.11
Population control	-0.03	0	0	0	0.09	-0.04	-0.08	0.06	0.15	0.98
Breeding	-0.53	-0.17	0.69	0.14	-0.24	0.18	0.27	-0.05	-0.17	0.06
Feral and pest control	0.03	0.04	0	-0.31	0.02	-0.44	0.32	-0.77	-0.09	0.06
Ecological restoration	0.07	0.05	0.07	-0.84	-0.33	0.27	0.11	0.2	0.23	0
Abiotic restoration	0.01	0.01	-0.03	-0.26	-0.04	0.01	-0.36	0.05	-0.89	0.11
Temporary resources	-0.16	-0.02	0.18	-0.29	0.89	0.04	0.16	0.17	-0.03	-0.08
Legal protection	0.82	-0.19	0.4	0.12	0.05	0.17	0.25	0.01	-0.14	0.07
**Standard deviation**	0.56	0.44	0.38	0.28	0.26	0.24	0.22	0.2	0.17	0.14
**Proportion of variance**	0.32	0.19	0.15	0.08	0.07	0.06	0.05	0.04	0.03	0.02
**Cumulative proportion**	0.32	0.51	0.66	0.74	0.81	0.86	0.91	0.95	0.98	1

Conservation management across taxonomic classes – Conservation management action was reported across 21 taxonomic classes of organisms included in our dataset (Extended Figure 7). We note that although we have referred to these moderators as conservation management actions, it is plausible that such action might not necessarily have a conservation motivation, although the action is expected to improve conditions for a focal population (see Methods). The five taxonomic classes with the most data for the different types of conservation management action were the same as those that had the most reports of ecological disturbance (i.e., Mammalia, Aves, Actinopterygii, Insecta, and Magnoliopsida). As in our main dataset, legal protection was the most common

conservation action reported across most taxonomic classes, and it was often the only type of action reported (Figure 3). This was true across the five most data-rich taxonomic classes, except for Magnoliopsida where legal protection ranked second after conservation introductions. For the remaining four most data-rich classes, the second most common actions were supplementation (Actinopterygii), breeding (Mammalia and Insecta), ecological restoration (equal ranked with breeding for Insecta), and feral and pest control (Aves).

Breaking down our dataset by taxonomic class revealed some correlations among conservation management actions that tended to co-occur (Supporting Figure 6). Note that these correlations differ from those presented in Supporting Table 16, as they include the full systematic review dataset (rather than the meta-analytical dataset that was further reduced). Breeding and temporary resources showed a weak to moderate positive correlation for Mammalia ($r = 0.36$, $p < 0.001$), Actinopterygii ($r = 0.37$, $p < 0.001$), and Insecta ($r = 0.68$, $p < 0.001$), and breeding was also positively correlated with supplementation for Insecta ($r = 0.68$, $p < 0.001$) and Aves ($r = 0.36$, $p < 0.001$). Ecological restoration was often reported alongside other actions, which varied by class, but included abiotic restoration (Actinopterygii: $r = 0.36$, $p < 0.001$), supplementation (Mammalia: $r = 0.71$, $p < 0.001$; Insecta: $r = 0.4$, $p < 0.05$), temporary resources (Insecta: $r = 0.4$, $p < 0.05$), and feral and pest control (Aves: $r = 0.39$, $p < 0.001$). Feral and pest control also showed a weak positive correlation with reintroduction in Mammalia ($r = 0.35$, $p < 0.001$) and abiotic restoration in Aves ($r = 0.32$, $p < 0.01$). Population control was strongly correlated with breeding for Magnoliopsida ($r = 0.81$, $p < 0.001$), and supplementation and temporary resources always co-occurred for Insecta ($r = 1.00$, $p < 0.001$); however, the latter result was driven by several populations from within a single study. As for ecological disturbance, we have identified and analysed separately those data generated in contexts where species are identified as domestic, pathogens or pests (although the latter is not presented due to the lack of conservation management action reported in this group). For those contexts identified as domestic populations, population control was weakly correlated with supplementation ($r = 0.39$, $p < 0.001$).

A few conservation management actions were rarely reported together, producing negative correlations that varied across the taxonomic classes, but which usually included legal protection (Supporting Figure 6). For example, legal protection tended not to occur with supplementation (Actinopterygii: $r = -0.40$, $p < 0.001$), breeding (Mammalia: $r = -0.36$, $p < 0.001$; domestic populations: $r = -0.40$, $p < 0.001$), population control (Mammalia: $r = -0.32$, $p < 0.001$), reintroductions (Insecta: $r = -0.48$, $p < 0.05$), conservation introductions (Magnoliopsida: $r = -0.83$, $p < 0.001$), or temporary resources (Aves: $r = -0.45$, $p < 0.001$). We also observed that breeding and supplementation tended not to co-occur for domestic contexts ($r = -0.67$, $p < 0.001$).

Ordination of the variation in conservation management action showed that legal protection explained the most variation in these variables across our five major taxonomic classes (Supporting Figure 6), alongside supplementation (Mammalia and Actinopterygii), feral and pest control and temporary resources (Aves), ecological restoration (Insecta), and conservation introduction (Magnoliopsida). Within our top five taxonomic classes there were no clear patterns in the conservation management actions reported for threatened versus non-threatened species (here non-threatened includes unlisted and Data Deficient species). This was also the case for those contexts identified as domestic populations, which were analysed separately. For domestic contexts, breeding and supplementation explained most of the variability in the conservation management action data, rather than legal protection.

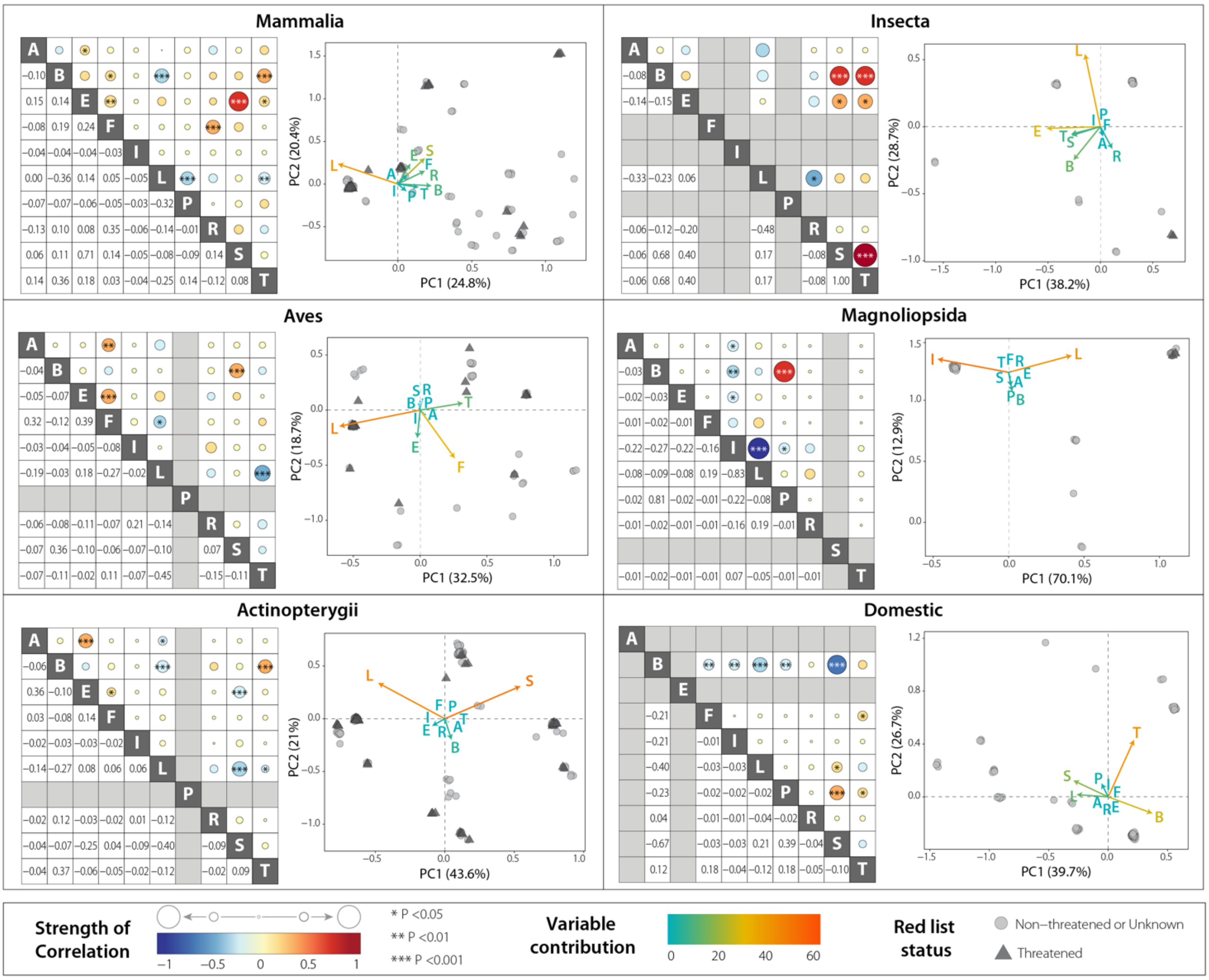

Supporting Figure 6. Conservation management actions reported for unique populations of species per unique study, across the five most data-rich taxonomic classes (Mammalia, Aves, Actinopterygii, Insecta, and Magnoliopsida) and domestic species, including correlations between action types and PCA biplots. The lower half of the correlation plot shows the correlation coefficient, the upper half shows the strength of correlation represented by color and size, the asterisks show significance, and greyed out actions were not recorded in the data subset. In the PCA biplots, point color and shape represent IUCN Red List threat status, and arrow and letter color represent the variable contribution to the PCA. Letters in plots represent conservation management actions (A = abiotic restoration; B = breeding; E = ecological restoration; F = feral and pest control; I = conservation introduction; L = legal protection; P = population control; R = reintroduction; S = supplementation; T = temporary resources).

SUPPORTING INFORMATION 2. SUPPLEMENTARY METHODS

Supporting Information 2.1. Literature search

Our Web of Science (WOS) advanced search did not include terms associated with locations, taxa or institutions, so as to obtain studies that measure genetic diversity across a wide range of regions and organisms (Supporting Table 9, Supporting Data 4). Search results were downloaded as BibTeX citations and duplicate records were removed in R[97] using the package *revtools* v 0.4.0[97]. We retrieved full texts using the automatic download options in Zotero v 5.0.60[99] and Endnote v X9[100]. Where full texts could not be retrieved automatically, the titles, keywords and abstracts were manually screened using an exclusion flowchart to support processing of the large number of publications captured by our database searches (Supporting Figure 7a). Papers published in languages other than English were captured in our search, and eligible for inclusion in our dataset, provided the study met our inclusion criteria (Supporting Figure 7b). When identified in our search, these were flagged and then screened by a member of the authorship team who was fluent in the relevant language.

Supporting Table 9. Summary of the Web of Science advanced search option strings used in the literature search.

Term	Advanced search options
TOPIC	(genetic* OR “effective population size” OR genom*)<br>*AND*: (population*)<br>*AND*: (NE OR temporal OR monitor* OR historic* OR museum OR archeolog* OR archaeolog* OR change* OR declin* OR grow* OR increas* OR decreas* OR bottleneck* OR expan* OR erosion)<br>*NOT*: (patient OR cancer OR clinic* OR hospital OR hypertension OR microbiom*)<br>*NOT*: (physics)
DOCUMENT TYPES	*NOT:* (Meeting Abstract OR Correction OR Review OR Database Review OR News Item OR Retracted Publication OR Biographical Item OR Editorial Material OR Book Review OR Software Review OR Book Chapter)
WEB OF SCIENCE CATEGORY	*NOT:* 55 WOS categories, including any category related to human medicine (e.g., Critical Care Medicine).
PUBLICATION NAME	*NOT*: 6 human specific journals (e.g., American Journal of Human Genetics).
CONFERENCE	*NOT*: 1510 conference titles. Despite excluding the “Conference Proceedings Citation Index” from our search, we still got 3000+ hits associated with conferences (which had to be individually excluded by conference title).
TIMESPAN	All years
INDEXES	SCI-EXPANDED

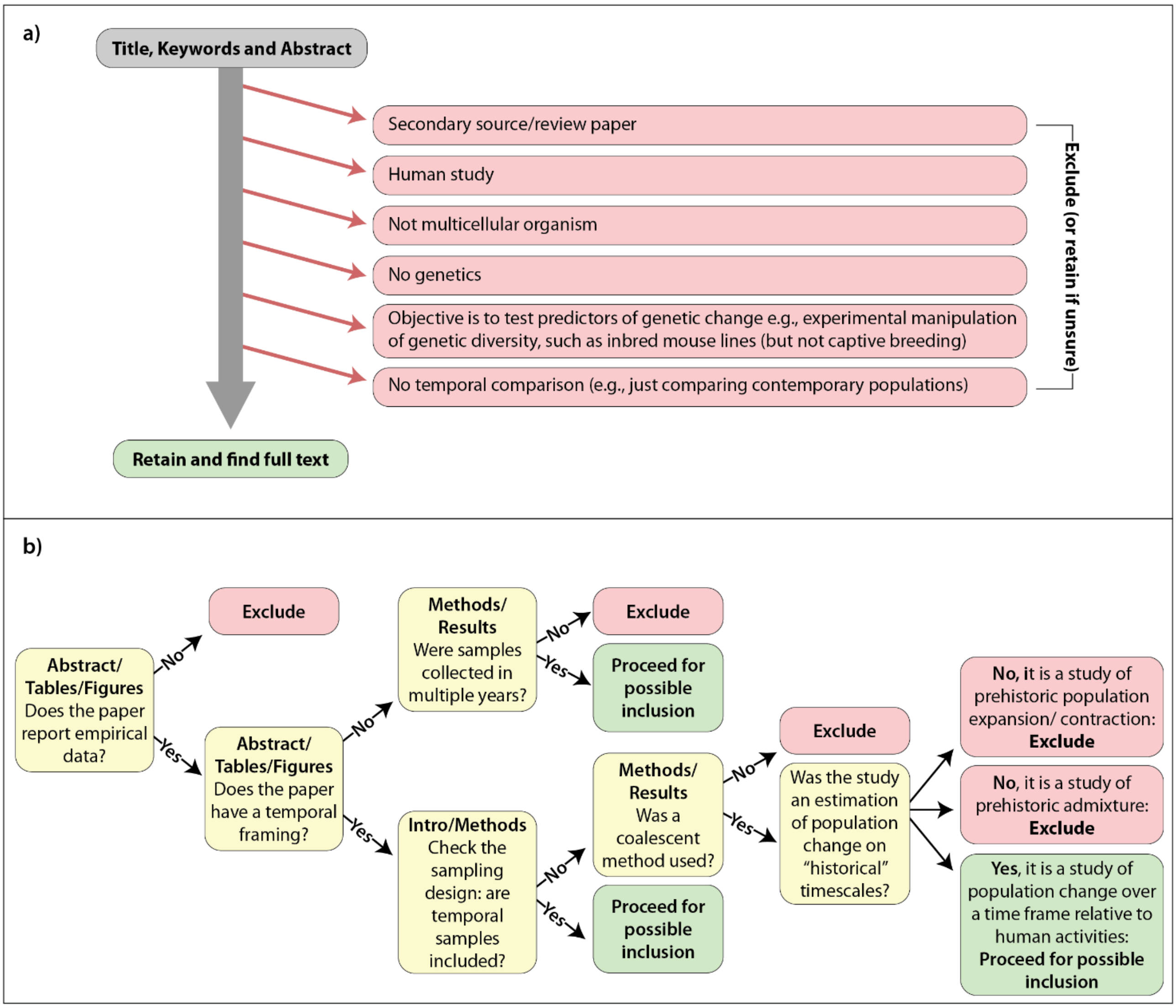

Supporting Figure 7. a) Simplified exclusion flowchart to support manual screening of studies for which full texts were unavailable for automatic download, based on titles, keywords and abstracts of BibTeX citations, and b) Detailed screening flowchart to determine possible inclusion of study where full text was available (see Supporting Information 2.2-2.3).

Supporting Information 2.2. Text mining

Custom text mining code is available on Zenodo DOI: 10.5281/zenodo.13903787. We also briefly describe our approach below.

Page number identification – We used text mining to further refine our search results, using specific terms representing analyses and metrics commonly employed in genetic studies. We performed text mining on the full texts to identify studies where these terms were found in the Methods and Results sections, since these were more likely to report empirical genetic data. Our custom text mining R scripts first searched for “Methods” and “Discussion” headings, to restrict the text mining to these sections using relevant page numbers. If no “Methods” heading was found, we started from page 1. If no “Discussion” heading was found, we used the “Acknowledgements” heading (or page 50 if this section was also not detected).

Keywords – Next, each full text PDF was searched for combinations of keywords, based on metrics likely to be in papers that measured genetic diversity over multiple time-points (Supporting Table 10). These included “alleles” (mean number, total and private, richness), “diversity” (nucleotide, haplotype, Shannon), “loci” (number polymorphic and polymorphic information content), “heterozygosity” (expected [H_E] and observed [H_O]), “F/G statistics” and “effective population size”. We also included several keywords related to methods often used in temporal genetic studies, including “bottleneck” (alongside the relevant software citation to avoid biasing search results with studies that report a bottleneck), “Markov-switching vector autoregression” and “approximate Bayesian computation”. Text mining refined the dataset to 34,346 records.

Thematic clusters – The refined list of 34,346 records was manually screened to determine whether each contained data suitable for inclusion in our study (e.g., following Supporting Figure 7b). This involved a streamlined approach to data management that initially divided the papers into smaller batches based on similar topics. These thematic clusters were generated solely for the purpose of allowing team members to self-assign to portions of the dataset for screening (and possible data extraction) that aligned most closely to their own knowledge and expertise. This strategy ensured that subject-matter expertise was available for the interpretation of particular population genetic data, and that team members extracted data from papers that include methods that they are familiar with. Thematic clustering of papers for screening was achieved by dividing papers into five broad thematic groups based on text mining keywords (Supporting Table 11). Within these groups, the *revtools* v 0.4.0[98] package was used to visualise the content of papers using topic models, clustering similar papers based on highly weighted topics in study abstracts (Supporting Figure 8). These topics were used to describe each screening sub-folder (Supporting Table 11), within which, papers were divided into batches of approximately 100. A small number of PDFs were not machine readable (278 records) and were grouped into a separate folder.

Supporting Table 10. Text mining rules used to identify sixteen keywords. Unless otherwise stated, searches were restricted to the Methods and Results sections (or within the full text if these sections were unidentifiable).

**Keywords**	**Text mining rule**
Approximate Bayesian Computation (ABC)	"Bayesian" must appear within one line of "Approximate" within the full paper AND "ABC" OR "ONESAMP" appear within the methods/results sections
Markov-switching vector autoregression (MSVAR)	"MSVAR" appears within text
Diversity (nucleotide)	"Diversity" must appear within one line of "nucleotide"
Diversity (haplotype)	"Diversity" must appear within one line of "haplotype"
Diversity (Shannon)	"Diversity" must appear within one line of "Shannon"
Heterozygosity (H_E)	"Heterozygosity" AND " He " OR " He= " OR " He," appear in text (note spaces so hits within words aren't included)
Heterozygosity (H_O)	"Heterozygosity" AND " Ho " OR " Ho= " OR " Ho," appear in text (note spaces so hits within words aren't included)
F/G statistics	"F-statistics" OR "G-statistics" OR "F statistics" OR "G statistics" OR "FST" OR "F'ST" OR "GST" OR "G'ST" appear in text
Alleles (mean)	"Alleles" must appear within one line of "mean" AND "number"
Alleles (total)	"Alleles" must appear within one line of "total" AND "number"
Alleles (private)	"Alleles" must appear within one line of "private"
Alleles (richness)	"Allelic" must appear within one line of "richness"
Loci (number polymorphic)	"Polymorphic" must appear within one line of "number" AND "loci"
Loci (Polymorphic Information Criteria)	"Polymorphic" must appear within one line of "information" AND "criteria"
Effective population size (Ne)	"Population" must appear within one line of "effective" AND "size" within the full paper AND " Ne " OR " Ne=" OR " Ne," appear within the methods/results sections (note spaces so hits within words aren't included)
Bottleneck	"Bottleneck" must appear within one line of "Piry" (note that this is to identify studies using the program bottleneck, rather than biasing our search to studies that detect a bottleneck)

Supporting Table 11. Thematic groups and screening topics for data management, used to divide papers into smaller batches so that team members could screen topics that aligned most closely to their own knowledge and expertise.

Broad thematic group	Topics (screening sub-folders)
ABC/MSVAR	• Breeding; Extinction; Fish<br>• Island; Invasive; Native; Introduction; Europe<br>• Isolation; Habitat; Gene<br>• Miscellaneous<br>• Simulations; Model; Inference<br>• Species; Divergence; Pleistocene; Climate
Diversity	• Evolution; Drivers; Adaptation<br>• Expansion; Pleistocene; Island; Refugia<br>• Haplotypes; Florida; Sequences<br>• Isolates; Primers; PCR; Cluster<br>• Methods; Tools; Genome; Validation<br>• Miscellaneous<br>• mtDNA; Origin; Europe; Asia<br>• Phylogeny; Morphology; mtDNA; nuclear<br>• Plant; Abundance; Community<br>• Pop; Spatial; Differentiation<br>• Populations; Altitude<br>• Rate; Hybridisation; Microsatellite<br>• River; Invasive; Fish; Conservation<br>• Selection; Expression; MHC<br>• Virus; Infection; Host; Strain<br>• Wild; Breeding; Cultivated; SNPs
F and G statistics/ heterozygosity	• Adaptation; Environment; Plasticity<br>• Australia; Island; Populations<br>• Biological; Processes<br>• Bottleneck; Founder<br>• Breeding; Cattle; Domestication; Wild<br>• Clusters<br>• Differentiation; Mantel<br>• Distribution; Expansion; Pleistocene; Refugia<br>• Divergence; Morphological; Lineages<br>• Diversity; Richness; Populations<br>• Gene; Functional; Protein; Expression<br>• Hybrid; Introgression; Interspecific<br>• Individual; Samples; Contaminated<br>• Landscape; Urban; Spatial; Dispersal<br>• Management; Restoration; Conservation<br>• Markers; AFLP; RAPD<br>• Microsatellite<br>• Miscellaneous<br>• Model; Methods; Simulations<br>• mtDNA; Haplotypes; Clades<br>• Mutation; Balancing; Selection; Evolution<br>• Origin; Native; Invasive; African; European<br>• Pathogen; Resistance; Strains; Host<br>• Populations; Partitioning<br>• QTL; Cultivars; Wheat<br>• Reproduction; Inbreeding<br>• River; Marine; Fish; Atlantic<br>• SNPs; Scan; Genome<br>• Time; Cohorts; Years; Spring<br>• Traits; Heritability; Growth; Body<br>• Tree; Clonal; Pollination

Continued over page.

Broad thematic group	Screening sub-folders (topics)
Allele/loci	• Breeding; Pedigree; Heritability<br>• Dispersal; Conservation; Tree; River<br>• Enzyme; Population<br>• Miscellaneous<br>• QTL; Chromosome; Wheat<br>• Reproductive; Mating; Sex; Offspring<br>• Resistance; Disease; Host; Gene<br>• Selection; Evolution; Drift; Mutation<br>• Species; Population; Divergence; Hybrid<br>• SSR; Microsatellite; Germplasm
Ne/Bottleneck	• Adapt; Evolution; Infection; Environmental<br>• Captive; Conservation; Management<br>• Census; Estimates; Magnitude<br>• Divergence; Speciation; Introgression<br>• Diversity; Levels; Population<br>• Genome; Selection; QTL<br>• Habitat; Fragment; Disturbance; Dispersal<br>• Introduction; Invasive; Australia<br>• Japan; Island; Mainland; Structure<br>• Mediterranean; Range; Distribution<br>• Methods; Model; Literature<br>• Miscellaneous<br>• mtDNA; Pleistocene; Expansion; Phylogeographic<br>• Origin; Domestication; Africa; Europe; Asia<br>• Pedigree; Sheep; Breeding<br>• Plant; Mating; Sexual; Selfing<br>• River; Fish; Hatchery<br>• Selection; Recombination; Mutations; Evolution<br>• SSR; Equilibrium; Variation
Unreadable PDFs	• N/A

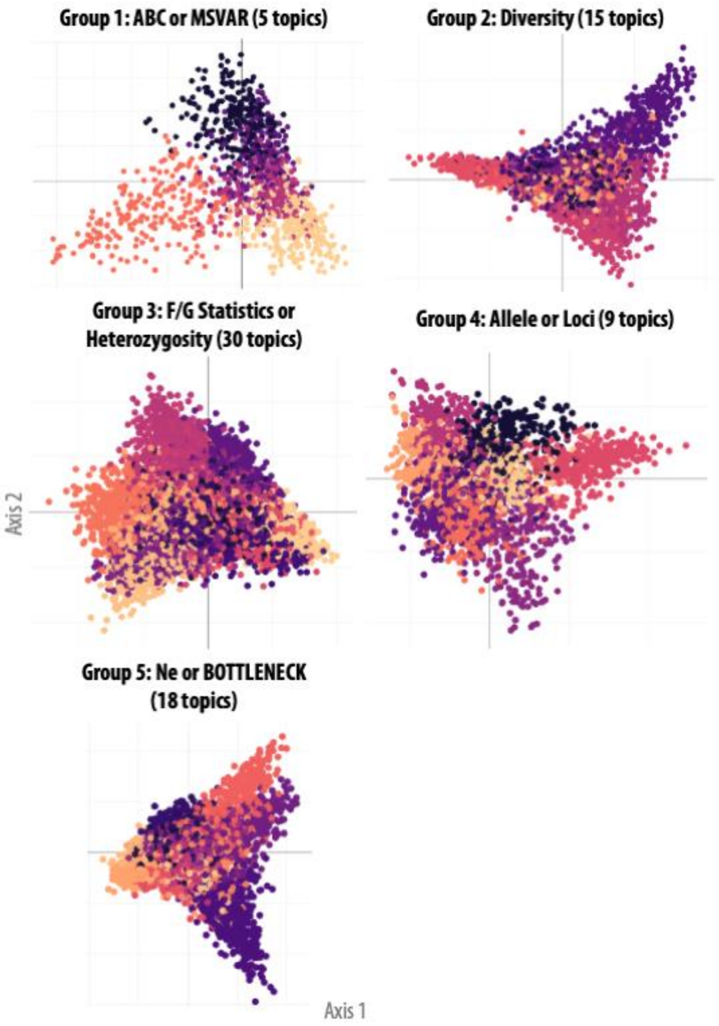

Supporting Figure 8. Ordination plots across the five broad themes used to group papers for manual screening. Each point within the five major groups represents a paper, where similar papers (represented by proximity and color) are clustered together based on highly weighted topics in study abstracts (described in Supporting Table 11). The number of topics ranged from 5 to 30 (noting that each group also included an additional "miscellaneous" topic), and unreadable PDFs were grouped separately.

Supporting Information 2.3. Data screening and extraction protocol development

Due to the size and complexity of the dataset generated, preliminary examination of several hundred papers by a core group of co-authors was used to create an initial screening and data extraction protocol, which was then tested by other members of the authorship team, and refined iteratively, prior to our main analysis. All data extracted during pilot stages were re-examined during our main data extraction.

To initiate our major data screening and extraction of the main dataset, we held a workshop in March 2020, in Tovetorp (Sweden) with 20 attendees (16 in-person, 4 virtual). Via discussion and consensus, we made minor clarifications and expansions to our extraction protocol upon identifying ambiguous papers that could not be easily classified according to our original definitions. This workshop enabled us to screen approximately 11,000 records (around 300 of which were deemed includable, and subsequently extracted). Following this in-person workshop, we held two further online workshops to recruit additional members to our research team and conduct the remaining extraction. To support team members in the correct application of our extraction protocol, and to ensure ambiguous cases were recorded consistently, we held weekly catchup meetings online where these were discussed. Multiple members of the core team were present at each catchup, to ensure continuity of consensus decisions over the course of the project.

Following the main data collection exercise, we formed subgroups to undertake collection of additional meta-data for our study species (Supporting Information 2.6) and to validate the dataset (Supporting Information 2.10). These activities were facilitated through shared in-progress documentation and regular online meetings to identify and manage ambiguous cases.

Supporting Information 2.4. Recording genetic diversity change

Measurements of genetic diversity change **–** We developed a data coding spreadsheet to record measures of genetic diversity change and associated meta-data from the included studies. All parts of a paper were eligible for screening and extraction (including text, tables, figures, and supplementary material). Where data were only presented graphically, values were extracted using GetData Graph Digitizer v 2.26 (getdata-graph-digitizer.com/download.php). Study population characteristics were extracted using the authors' description in their paper, and (to a point, limited by time investment) we attempted to follow-up works cited within the focal paper where appropriate (e.g., to obtain sampling years of previously published datasets, cited within the target paper). To maximise the use of published data, whilst minimising the potential for non-independent measurements of genetic diversity change entering our dataset, we conducted our initial data extraction using the following philosophy (summary only, see below, especially Supporting Information 2.9, for more detail):

1. Measurements should be independent temporal records of genetic diversity that reflect different evolutionary properties of a population or system.
2. We collected as many independent measures as possible from includable papers. For example, if a manuscript reported analysis of a population from multiple perspectives, and these perspectives capture separate evolutionary processes, all such results were potentially includable.
3. Where multiple time-points were reported, we targeted a "first to last" comparison in order to capture the most data.
4. We collected only "complete" data, i.e., measurements that were accompanied by all required statistics such as sample sizes, year of collection, error measurements, etc. Where errors were not reported, but data were presented in a format such that means and standard deviations were manually calculable over loci, or for biologically sensible groupings of populations or sampling time periods, we calculated summary statistics for these papers. However, we did not conduct any further analysis of published datasets (such as genotypic datasets uploaded to online repositories, such as Data Dryad, datadryad.org).
5. If multiple measurements of genetic diversity were reported that were not independent, we minimised bias by using the following hierarchy to determine which data to extract:

   First priority: measurements that had "complete data" for extraction (especially a clear measure of diversity, with associated error and sample size).

   Second priority: comparison that best represented an "overall" assessment of the study system.

   Third priority: the statistics that were reported first in the paper.
6. Where data were analysed with multiple statistical methods, we generally favoured recording statistics reported as a quantitative change (e.g., regression, see main Methods) provided the results were clearly described in a manner that could be aligned with our study design. If ambiguous, and a two time-point comparison was also available, the latter was extracted.

Time frame of the measurement **–** Our goal was to interpret genetic diversity change relative to human activities. We excluded studies reflecting prehistoric (phylogeographic) changes in the Pleistocene caused by natural long-term processes. We did not define a specific "time limit" for inclusion of coalescent studies: the relevant time frame was determined case-by-case using the human context as described by the authors (or in the absence of such, 500 years). In general, this means we targeted genetic diversity changes in the last few hundred years and excluded studies on ancient admixture/expansion e.g., in response to prehistoric climate events or events on "geological" timescales. Colonization of landmasses by humans was considered includable, even if thousands of years ago, if relevant to the population under study. Where samples were collected over multiple "early" and "recent" years for each time-point (e.g., museum specimens collected over several years, pooled as "historical" samples), we used a weighted average based on number of

samples per year as the “early” year, or the midpoint of the range of years if a weighted average could not be calculated.

Supporting Information 2.5. Impact meta-data

The influence of human activities on populations was captured by recording threatening processes and/or conservation management actions that were reported by authors, and which plausibly influenced the population between the sampling time-points of the study (regardless of whether the study population was actually affected). We focused on presence/absence of ecological disturbance events and/or conservation management actions in our analysis, acknowledging that this meant we were unable to attribute genetic diversity change to the timing of the disturbance event/management action. Multiple categories could be applied to individual cases in our dataset. We used three categorical variables to record the information:

***Ecological disturbance* –** Here we define ecological or other disturbances as events with potential to impair conditions for the focal species or its habitat (Supporting Table 12); we also included "unusual" disturbances that were not a direct result of human activities (such as natural disasters). We took a broad interpretation of "disturbance" as an unusual ecological change (either temporary or permanent). We acknowledge that such events do not necessarily lead to a loss of genetic diversity (e.g., many may have no effect, while others such as hybridisation may cause an increase in genetic diversity) and may have a wide range of biological implications beyond the focal species. We recorded any disturbance that applied during the study time frame, including those that started during the study period, and any that occurred prior to the study period, provided it could be reasonably presumed from the authors' description in the paper that the disturbance continued to impact the focal species during the study period.

***Conservation management* –** Here we include intentional human actions that are intended to improve conditions for the focal species or its habitat (Supporting Table 13), that plausibly have benefits for the demography and/or genetic diversity of the study population. We consider any conservation management action that may apply during the sampling period of the study, including actions that were initiated during the study period and any action that was initiated prior to the study period, provided it could be reasonably presumed from the authors' description in the paper that the action continued to provide the intended benefit during the study period. For example, predator control that started prior to "early" and continued throughout the study period applies; predator control that took place prior to "early", but was discontinued prior to "early", did not apply. Similarly, habitat restoration that was conducted prior to the "early" sampling point probably still benefits the species during the study period, unless it was explicitly indicated that the restoration failed or was reverted prior to "early".

***Management intensity* –** For all cases where at least one type of conservation management action was recorded, we also recorded general information to broadly quantify the magnitude of human intervention in the study population (Supporting Table 14). The categories we chose are not necessarily discrete, and we acknowledge that there is regional/taxonomic/contextual variation in the interpretation of management intensity. In our preliminary protocol development (see Supporting Information 2.3) we found that conventional discrimination between ex situ and in situ (e.g., "captive" and "wild" populations, respectively) was challenging to characterise reliably amongst the diversity of studies included in our analysis. Therefore, we instead chose levels that focus on the entity being targeted: individuals, populations, the broader environment, or none. We acknowledge that reserves, national parks, game parks, conservation islands, etc. do not have standardised definitions that align with our categorisations, and, therefore, that the categories we chose here may be different to how such sites are labelled in publications or elsewhere.

Supporting Table 12. Impact meta-data detailing threatening processes, including ecological disturbance category, abbreviation codes used for data collection, and descriptions.

**Category**	**Code**	**Description**
Abiotic human phenomenon	U	Abiotic processes that result from human actions including pollution, burning, processes linked by the authors to post-industrial global climate change, including acidification, glacier retreat, extreme weather, economic factors (domesticated species, i.e., maintaining a breed is no longer commercially viable), etc.
Abiotic natural phenomenon	N	Abiotic natural phenomena include wildfire, flood, river change, geographic change, pre-industrial climate change, or other abiotic processes not directly linked to human activities or post-industrial global climate change.
Disease emergence	D	Emergence of disease in the study population.
Ecological disruption	E	Ecological change as a result of human activities, such as loss of prey, loss of nesting cavities, loss of pollinators, etc.
Feral animals or plants impacting the study population	F	The presence of a feral species that impacts the species for which genetic data were collected. Includes weeds, predators, grazers, competitors, etc.
Harvest and harassment	H	Killing/stressing the focal species by humans for reasons other than conservation; including hunting, by-catch, poaching, logging, persecution, culling, direct poisoning, and tourism interference. Domesticated species are not automatically included, as the study must specify harvest.
Introgression	I	Including hybridisation, as a disturbance (e.g., animal or plant populations mixing in situ due to land use change), cross-species introgression, and within-species introgression. Examples may include unintended hybridisation during captive breeding, movement of marine organisms in ballast water, etc. (Note, introgression as a conservation action is classified as “supplementation”).
Invasion	V	The focal species is self-introduced to a site that it previously did not inhabit (includes range expansions, and colonisation). Note that human-mediated introductions of species for reasons other than the conservation of that species is included under “Non-conservation introductions by humans”.
Land use change	L	Including clearing, conversion to forestry/agriculture/mining, infrastructure, urbanisation (e.g., road installation/upgrade), water abstraction/dams, etc.; includes generic “habitat fragmentation”
Non-conservation introduction	O	Accidental or deliberate introduction of a population by humans, but not for the purpose of conserving the introduced species (e.g., biocontrol activities, introduction of invasive species outside their native range, etc).
Unspecified	S	Ecological disturbance is mentioned or implied, but insufficient details are provided to assign it to a category.
None	X	Any population for which no ecological disturbances were mentioned in the paper. Or, disturbance may be mentioned, but not across the timescale of the genetic data.

Supporting Table 13. Impact meta-data detailing conservation management, including management action (i.e., category), abbreviation codes used for data collection, and descriptions.

Category	Code	Description
Abiotic restoration	A	Activities that are intended to improve conditions for the target species via management of abiotic factors, such as prescribed burns, controlling river flow volumes, creating artificial habitat, etc.
Breeding	B	All forms of breeding management, including captive breeding, agriculture, selective breeding, artificial reproductive technologies, contraception, targeted removal of genetically over-represented breeders, etc.
Conservation introduction	I	Establishing a new site for conservation purposes, i.e., to increase the range or population size of the focal species, outside the species' native range. Includes establishing new populations of endangered species, introductions for ecological restoration (e.g., replanting), and assisted migration.
Ecological restoration	E	Activities that target species other than the species the genetic data were collected from, usually (but not always) to improve habitat, e.g., reforestation, aquatic restoration, re-vegetation, etc.
Feral and pest control	F	Activities that target feral and pest species other than the species the genetic data were collected from (often non-native, but native species may be included), e.g., removal of competitors, predators, grazing herbivores, etc.
Legal protection	L	All types of "legal" protection except the action of "IUCN red listing" a species, which is recorded elsewhere. Does not necessarily require the passing of legislation, but includes any mechanism that provides for the establishment of a set of protective rules that are recognised and/or enforced via social or governmental institutions. Examples include designating a region overlapping the study population's range as a national or conservation park or reserve, a restriction on hunting/harvesting. Includes protection that applies to a species wherever it is found (such as trade restrictions, private property, etc).
Population control	P	Controlled harvesting of the studied population to maintain or improve population parameters (e.g., removal of juveniles to improve survival rate).
Reintroduction	R	Population established at a site where previously extirpated.
Supplementation	S	Individuals added to existing population (i.e., often, but not always, for the purposes of genetic and/or demographic rescue).
Temporary resources	T	Provision of resources beyond the realm of ecological/abiotic restoration, such as supplementary feeding, nest boxes, fertiliser, and similar. Planting of food trees would typically be classified as "ecological restoration". Putting out temporary water supplies may be included under "temporary resources", but permanent modifications to water supply, such as redirecting streams, building dams, etc. may be included under "abiotic restoration", depending on context. Provision of resources to captive or semi-captive populations (even if ongoing or long-term) is included in "temporary resources". Our test for defining a resource as "temporary" was that, if human involvement were to cease, the resource would likely become unavailable or degraded by the next time it is needed (e.g., supplementary food provided during a storm that would need to be resupplied the next time there is a storm; nest boxes that have a short lifespan of only a few breeding seasons depending on the design and target species).
Unspecified	U	Conservation management is mentioned or implied, but insufficient details are provided to assign it to a category.
None	N	Any population for which no conservation benefits were mentioned in the paper, including "monitoring only" type studies. Or, conservation actions are mentioned, but do not apply over the time frame of the genetic data (e.g., actions that were initiated after the most recent genetic samples were collected).

Supporting Table 14. Impact meta-data detailing the magnitude of conservation intervention, including management intensity category and descriptions.

Category	Description
Individual	The focal species is subject to individual-level management, including zoos, botanic gardens and agriculture, etc. Any populations under intensive human management, e.g., individuals primarily obtain food/nutrients/water from human caregivers, they are housed in controlled pairs/groups for breeding or social purposes, and diseases are managed. All individuals are the direct intended beneficiaries of the management. Individuals are usually uniquely identifiable/tagged and demographics known (if taxonomically appropriate, e.g., aquatic species housed in a large school in an aquarium might not be individually identified, but because keepers provide the animals their food and determine the group composition [e.g., group size], they fit our "individual" definition as used here).
Population	The focal species is subject to targeted population-level or landscape-level management, including populations where some management is targeted at the study species, but it is not individually directed. This may include populations living in areas with intensive predator control (for the benefit of the focal species), partial supplementary provision of food/water/breeding sites/pollinators, etc. Individuals retain autonomy over their feeding/breeding/territories (e.g., individuals may or may not utilise supplementary feed or nest box). Part of the population may be individually tagged/monitored, and demographics estimated (e.g., by mark-recapture, sampling).
Indirect	The focal species is not the target of direct conservation action, but may benefit from indirect conservation actions, including populations living in areas where management occurs, but it is not targeted at the study species, such replanting of species that the focal species does not necessarily use for food/nesting (if it did, the "Population" designation may be more appropriate). A hunting ban for a different species, but living in the same area, would also be considered "indirect". Exclusion of human activities is included, provided such exclusion is not specifically targeted at the focal species. For example, a fishing ban for a harvested species is "Population" management, a general fishing ban at a site is "Indirect" management. "Indirect" may also apply to species sampled in national or marine parks, where management may occur for other species, but the species that is the focus of the genetic analysis in the paper is not specifically managed.
Mixed	Multiple of the above, but reported collectively, or multiple populations analysed collectively.
Unknown	Unable to determine management intensity from the information reported in the paper.
None	Any population for which no conservation benefits were mentioned in the paper.

Supporting Information 2.6. Additional variables

Taxonomic information – We used the R package *rotl* v 3.0.12[101] to resolve taxonomic names to internal identifiers (OTT IDs) by matching the binomen collected from the paper with records in the Open Tree Taxonomy[102]. These were used to generate a phylogenetic tree (described at Supporting Information 2.11) and to collect additional higher order taxonomic information (kingdom, phylum, class, order and family). Silhouettes of representative organisms for each taxonomic class were downloaded from PhyloPic (www.phylopic.org; Supporting Table 15).

Genetic marker type – Underlying data used to generate genetic diversity measurements (which we refer to as "markers"), classified as: allozyme (including isozyme), amino acid (including protein sequences), amplified fragment length polymorphism (AFLP), chromosome (karyotypes), haplotype (set of genetic variants that tend to be inherited together, and are analysed as a unit), microsatellite (also known as simple sequence repeats, SSRs), minisatellite (including DNA fingerprinting, band sharing), nucleotide (full sequences of genes or candidate loci), single nucleotide polymorphisms (SNPs, nucleotide filtered data targeting only polymorphic loci, including reduced-representation sequencing, SNPchips, etc.), whole-genome sequences, other, and mixed. We also recorded whether data were generated from the nuclear, mitochondrial, or chloroplast genomes (or mixed), and the total number of loci or base-pairs analysed (where relevant for marker type). Where data were separately presented using multiple marker types (e.g., a study reporting both microsatellite and mitochondrial analysis), both were includable in our analysis, provided one was not a subset of the other (e.g., a multigene analysis, plus a detailed analysis of a single gene – only the larger analysis would be includable).

Generation length – Evolutionary genetic diversity change in populations occurs generationally, but is typically observed/recorded in years for wild populations. For our main analysis, we considered genetic diversity change per generation by calculating the number of generations as the duration of the study divided by the generation length of the species. We collected the generation length of all species included in our dataset. Where available, we used the value for generation length reported in the paper. Where values were not reported, we obtained values from a range of published databases, papers or websites, or used data provided from these resources to calculate generation length (as the average age of adult individuals). For example, for long lived trees and shrubs we calculated generation length as age of first reproduction + z ∗ length of reproductive period[103], where z was estimated as 0.33 as calculated for the long-lived tree *Araucaria cunninghamii*[104]. Global databases included Search FishBase (www.fishbase.se/search.php), AmphibiaWeb (www.amphibiaweb.org), CABI Compendium (www.cabidigitallibrary.org/journal/cabicompendium), as well as those in published articles[105,106]. Due to the wide diversity of species in our dataset, generation length (in years) varied through several orders of magnitude across species. Generation length can also vary for a given species depending on the definition used to calculate it, and challenges associated with certain life histories (such as plant species with seeds that may germinate after many years of dormancy, or species that reproduce clonally or vegetatively). Our final list of generation lengths for all species included in our dataset, along with notes recording the source of information, is available at Supporting Data 5.

Domestic species – The population genetic dynamics of domestic species may differ substantially from wild populations, such as differences in effective population size (N_e), the N_e to census size ratio, controlled breeding and its effects on generation length, controlled culling, veterinary treatment, and other factors. As such, it was important to determine whether such species showed a different trend in genetic diversity change relative to other species in our dataset. In our dataset, taxonomy was recorded at the species level and cultivar or breed names were ignored. The domestication status of species was determined through web searches and expert knowledge of the authorship team. We only considered full domesticates (i.e., species undergoing a sustained multi-

generational relationship in which humans assume a significant degree of control over the reproduction and care of another group of organisms to secure a more predictable supply of resources from that group[107]). Some species may exhibit some aspects of domestication (partial), but we considered this information to have been captured by our impact meta-data variables. A common line of enquiry in domestication studies is comparison with wild relatives or unmanaged populations, so for those species identified as domesticates in our analysis we re-examined the source publications to ensure that the extracted genetic data were correctly labelled as domesticated populations where appropriate.

Threat status – Species-level threat status was used as a coarse metric of species history and demography, whereby species of higher threat level are likely to have smaller population sizes, greater fragmentation of populations, a history of decline, and are more vulnerable to ecological disturbances and more likely to experience conservation management actions, compared to less-threatened species. A species' threat status may be classified under a range of legislative instruments at regional, national or international scales. Our main data extraction included the "conservation action" variable "legal protection" (see Supporting Information 2.5), which we have defined broadly to encompass any of the diverse legal procedures by which a population may be protected. "Legal protection" was only recorded if mentioned by the authors of a paper, and therefore focussed only on the population/range for which the genetic data were generated. Due to the global perspective and wide use of the IUCN Red List threat status categorisations, we also recorded Red List status as a separate variable, based on the species identity (IUCN Red List version 2021-2, accessed between Jun-Aug 2021). If a publication mentioned listing of a species on the IUCN Red List, this was not recorded as "legal protection" under "conservation management actions" (unless additional legal actions were identified by the authors), to avoid confounding between these two variables in our analysis.

Invasive species – Populations of species identified as invasive ("invasive species") are likely to have different population genetic dynamics compared to non-invasive species, because many invasive populations grow rapidly following a population bottleneck, are exposed to novel selection pressures, and are subject to control measures to varying degrees[108]. As such, it was important to identify invasive species in our dataset, to determine whether genetic diversity change in this group differed from the overall trend. This was a challenge because invasive status can have multiple definitions, and is dependent on sampling location (native or invasive ranges). For example, the European rabbit (*Oryctolagus cuniculus*) has a threatened status in its native range, but is considered invasive outside of Europe. We therefore identified whether any of the taxa in our dataset were recorded as invasive species according to the IUCN's *100 of the World's Worst Invasive Alien Species* list (www.iucngisd.org/gisd/100_worst.php). We note that "Invasion" is also captured in our impact meta-data as a category under "ecological disturbance" for individual rows of data (Supporting Information 2.5).

Pathogens and pests – Pathogen and pest species may have different population dynamics to their host species, which may impact on the interpretation of genetic diversity change. In particular, pathogen and pest species may respond differently to disturbances, and are often the target of control activities (either directly, or via their host) that would constitute an "ecological disturbance" by our definitions. These data focussed on Least Concern, Data Deficient and unlisted species. Descriptions within the source publications, as well as information from the broader literature, supported by global databases including the European and Mediterranean Plant Protection Organization Global Database (gd.eppo.int/), The Global Pest and Disease Database (www.gpdd.info), and CABI Compendium (www.cabi.org/isc), were used to determine if a species was a pathogen or pest. We then re-examined these individual studies to ensure that the extracted genetic data were associated with pest or pathogenic populations, since pest/pathogenic status can

vary depending on geographic location or context and studies may provide comparison to non-pathogenic or non-pest populations.

Supporting Table 15. PhyloPic (www.phylopic.org) image credits and licenses for organism silhouettes representing taxonomic classes.

Class	Credit	License
Actinopterygii	Sherman Foote Denton (illustration) and Timothy J. Bartley (silhouette)	CC BY-SA 3.0
Agaricomycetes	Guillaume Dera	CC0 1.0
Amphibia	Steven Traver	CC0 1.0
Anthozoa	Victor Piñon-González	CC0 1.0
Arachnida	Birgit Lang	CC0 1.0
Ascidiacea	Melissa Frey (photograph) and Malio Kodis (silhouette)	CC BY-NC 3.0
Asteroidea	Birgit Lang	CC0 1.0
Aves	Andy Wilson	CC0 1.0
Bivalvia	Katie Collins	CC0 1.0
Branchiopoda	Mathilde Cordellier	CC BY-NC 3.0
Calcarea	Guillaume Dera	CC0 1.0
Cephalopoda	Margot Michaud	CC0 1.0
Chromadorea	Smithsonian Institution (image) and Malio Kodis (silhouette)	CC BY-NC 3.0
Dothideomycetes	Guillaume Dera	CC0 1.0
Echinoidea	Harold N Eyster	CC BY 3.0
Elasmobranchii	Zimices (Julián Bayona)	CC BY 3.0
Eurotatoria	Sofdrakou (photograph) and Julie Blommaert (silhouette)	CC BY-SA 3.0
Gastropoda	Martin R Smith	CC0 1.0
Hexanauplia	James Bernot	CC0 1.0
Insecta	Wouter Koch	CC0 1.0
Leotiomycota	Guillaume Dera	CC0 1.0
Liliopsida	Jonathan Wells	CC0 1.0
Magnoliopsida	T. Michael Keesey	CC0 1.0
Malacostraca	Joanna Wolfe	CC0 1.0
Mammalia	Ferran Sayol	CC0 1.0
Monogenea	Timothée Poisot	CC0 1.0
Peronosporea	Kelsey Wood	CC0 1.0
Petromyzonti	Christoph Schomburg	CC0 1.0
Phaeophyceae	Yan Wong	CC0 1.0
Pinopsida	Gabriele Midolo	CC0 1.0
Polychaeta	B. Duygu Özpolat	CC BY-NC-SA 3.0
Pucciniomycetes	Levi Simons	CC0 1.0
Reptilia	Jose Carlos Arenas-Monroy	CC0 1.0
Scyphozoa	Levi Simons	CC0 1.0
Sordariomycetes	Guillaume Dera	CC0 1.0
Trematoda	Maxime Dahirel	CC BY 4.0
Ulvophyceae	Sergio A. Muñoz-Gómez	CC BY-NC-SA 3.0

Supporting Information 2.7. Identification and removal of non-independent data

Duplicate publication of genetic data – In our study, non-independent data may arise when a genetic dataset is used as a common point of comparison for multiple works (e.g., an early measure of genetic diversity that is subsequently compared to more recent measures taken at different time-points), or when a dataset is expanded in a new publication (e.g., a study based on a small number of loci is later expanded to draw new inferences, i.e., more loci/samples/populations added). Duplication was suspected when a later publication cited an earlier work in reference to a similar or partially overlapping dataset. Such cases were flagged and then manually examined for duplication against our inclusion criteria as a separate exercise after the main data extraction.

To identify any possible non-independent data that were undetected using the above method we further tested our extracted dataset for putatively duplicate entries. This was achieved using a custom R script, which identified duplicated information across studies using binomen, realm, marker type, genome, neutrality, and genetic diversity metric type. In cases where this information matched, the time frames of these studies were compared to check if they overlapped. Overlapping studies were then manually examined to determine if they used the same dataset (or if a subset of the data was re-analysed), and where this was true, one of the pair was excluded following our screening and extraction protocol (i.e., targeting our priority genetic diversity metrics, and/or the largest dataset, and/or the most recent study).

Stochastic dependency – A special case of statistical non-independence can occur when multiple independent measurements are made from the same genetic dataset (e.g., estimating both individual-level and population-level evolutionary hypotheses), resulting in correlated estimates of genetic diversity change. It can also occur when multiple measures of contemporary genetic diversity are compared to the same historical baseline; however, our data extraction procedure only required a single time-point comparison be extracted per population. Where multiple estimates of genetic diversity change are generated for a single population, we controlled for this using the study ID random effect in our modelling.

Supporting Information 2.8. Missing data

Statistical error not reported **–** We did not perform multiple imputation of missing data due to the large sample size of complete cases in the dataset. Instead, we conducted a qualitative assessment of absent data, to investigate whether those studies that were excluded differed from the main analysis in any of the major analytical axes, e.g., species and realm (Supporting Information 1.1).

Infinite confidence intervals **–** Confidence intervals that included "infinity" (or "negative infinity"), rather than absolute values were collected during our main data extraction, as they were valid measurements of genetic diversity change and met our inclusion criteria. This was also true for studies that reported infinite values elsewhere (e.g., infinite mean value for N_e estimates from coalescent methods). However, we were unable to calculate effect sizes from this type of data. Therefore, we qualitatively summarised 96 records from 45 studies that fit this criterion to determine if these data differed from our main data set (Supporting Information 1.1). Note that the majority of these were subsequently excluded for other reasons, resulting in only 14 records being excluded for 'infinite confidence intervals' in our PRISMA.

Zero variance **–** Error estimates of zero (including variance, standard deviation, etc.) were collected during our main data extraction, as they were valid measurements of genetic diversity change and met our inclusion criteria. However, we were unable to calculate effect sizes for these measurements, as they have no error (infinite precision). We did not investigate whether these data differed from our main dataset, as only eight studies fell into this category.

Supporting Information 2.9. Unconventional study designs

Adult/juvenile comparisons of plants – As a general rule, we did not include comparisons between parents and offspring sampled at a single time-point. The exception was plants, if the following conditions were met: a quantitative measure of the "time frame" was present. For example, 40-year-old adults compared to 100-year-old adults have a time frame of 60 years between them; or seedlings versus adults, where the "mean generation time" is clearly specified (and which can be used to calculate the time frame of the study). In addition, such studies were only included if it was possible to quantitatively assign time frames to groups (e.g., mixtures of offspring produced over a range of unspecified dates were typically not includable). For research with this study design, we preferred data that compared adult cohorts to one another, and the longest comparison possible (in line with our extraction protocol) (Supporting Information 2.4). Comparing adults to juveniles was a lower priority statistic, and only extracted if no other data were available in the paper. We recorded a flag "Adults and juveniles sampled at a single time-point" to identify such studies in our dataset. We acknowledge that inference around these kinds of "temporal" comparisons is complicated, because of selective processes that may occur between adults and seedlings. We further acknowledge that permitting this type of comparison for only one taxon (plants) is not ideal, but many studies in very long-lived trees are conducted in this way, and so excluding such studies would inhibit inferences from a major taxonomic group.

Before/after comparisons over very short time frames – Rapid ecological disturbances or conservation management interventions may give rise to study designs in which before/after genetic diversity measurements are taken over a short time period (less than 1 year). Such studies were included in our dataset, with the same year recorded as the "early year" and "recent year", giving a time period of 0 years (interpreted as less than 1 year). Effect size calculation does not take into account the time period between the "early year" and "recent year" so was not affected by these cases. However, the number of generations of the focal species in these cases was set to zero, as across our dataset we recorded year as an integer, not portions of a year (e.g., in months). Note that some year entries in our dataset do include non-integer values, which have occurred as a result of averaging years across a reported sampling window, not as a result of recording portions of a year.

Within-study variation in number of samples or loci – In some genetic studies, researchers genotype or sequence multiple loci, but only report results for some of these (e.g., loci were dropped due to genotyping error, deviation from HWE, or other reasons). Similarly, the analysed sample size may be smaller than the number genotyped or sequenced (e.g., some samples excluded due to poor quality data). For data extraction, we targeted the sample size of loci/individuals corresponding to the statistics being extracted. Where the number of loci varied across comparisons, or the number of samples varied across loci, we used totals as reported in the paper.

Uncommon genetic diversity statistics – Where a clear measure of genetic diversity was recorded that was not captured by one of the main categories in our extraction protocol, it was recorded as "Other", and classed into one of the four genetic diversity metric types as defined in our main Methods, based on the statistical properties of the metric (e.g., whether it targets population or individual-level genetic diversity). We also examined the source paper, as well as related literature, to confirm whether the statistic had a "positive", "negative" or "undetermined" relationship with genetic diversity. For example, number of alleles and heterozygosity are both positively correlated with genetic diversity (larger values are associated with higher genetic diversity); inbreeding and relatedness are both negatively correlated (larger values are associated with lower genetic diversity). Where a directionless difference was measured, it was recorded as an "undetermined" relationship to genetic diversity and, therefore, excluded from meta-analysis.

Uncommon genetic marker types – Where a clear measure of genetic diversity was derived from a data type that was not captured by one of the main genetic marker type categories in our extraction protocol, it was recorded as "Other". Many of these forms of genetic diversity were based on non-laboratory methods, such as pedigree statistics and phenotypic traits. For each of the rows attributed an "Other" genetic marker type, the original paper was scrutinised to ensure the correct sample size was used for the effect size calculation, and that the genome, neutrality, number of loci or number of base pairs were correctly recorded (where appropriate).

Data subset analyses – It is not uncommon for empirical studies to report an "overall" analysis of the data, as well as meaningful subset analyses of the same dataset. For example, a paper may report "male", "female" and "overall" analyses in line with various hypotheses, where the "overall" is the pooled data of "male" and "female", and therefore duplicated information. To avoid non-independence in our data from this issue, we used the following hierarchical framework to identify suitable data for inclusion:

1. If data subsets were natural delineations of the system without overlap (such as comparisons in different countries or species) we extracted data from each subset and not the "overall".
2. If data subsets were breaks in the data that applied to a research question (e.g., males vs. females, healthy vs. diseased), but it is reported, or reasonable to assume, that the data subsets were otherwise generated from a single, interbreeding population, we used "overall" statistics and not subsets.
3. If the "overall" would be preferred based on criterion #2, but full statistics are not given for the overall (or are incomplete), and instead the subset findings are complete, we extracted data from the subset(s) and not the overall data. Depending on the study design, we either included all subsets, or the one with the largest sample size, by consensus discussion with other members of the authorship team to ensure extracted data were independent.
4. If it was ambiguous how data were divided, we extracted the subset that had the most complete statistics, the clearest definition, and/or the largest sample size, depending on which of these criteria was the least ambiguous.

Excluded from effect size calculation – Two among-population F_{ST} comparisons did not contain enough information in the paper to reliably estimate the sample size required for effect size calculation, so were considered non-calculable.

Supporting Information 2.10. Repeat extractions and validation

Overview **–** We sought to ensure the consistency of data extraction across our large extraction team, given the heterogeneity of our dataset, by conducting repeat extractions. Members of the 'core group' repeated the data extraction of a random selection of 150 "includable" papers, targeting rows of data that they themselves had not initially extracted. We found 15 papers that should have been excluded, and other minor errors, such as additional extractable data not included and minor data entry errors. As a result, two subgroups of our authorship team re-examined all included papers to reduce inconsistency in data extraction, data entry, or interpretation across our dataset (data validation subgroup members identified under "Author Contributions" in the main manuscript). Each subgroup contained multiple members of the 'core group', who had higher level oversight of the project to ensure consistency in approach and decision making.

Validation subgroup 1 **–** Inclusion criteria and genetic data / statistics. The first validation subgroup tested for correct inclusion of data rows, and specifically checked and validated the extracted genetic data variables. The subgroup comprised authors who had contributed to a major portion of the original extraction, and were therefore familiar with the types of studies that have entered our dataset and where ambiguities might occur. Each row was validated by a different researcher to its original extractor. To ensure consistency, in all cases where the validating researcher considered that a different inclusion/exclusion decision should be made, compared to the original extractor, a consensus discussion of at least two members of the validation subgroup was used to confirm the validation.

Validation subgroup 2 **–** Impact meta-data. Our initial repeat extraction process also revealed that the data recorded on conservation interventions and ecological disturbances had a high degree of discrepancy, primarily due to conservation and ecological contexts appearing outside our original definitions, and minor differences of opinion in interpretation. To resolve this, we held workshops amongst the authorship team to refine our category definitions within these two variables and formed a second validation subgroup to scrutinise our entire dataset against these variables. This subgroup comprised authors who had contributed to a major portion of the original extraction and were therefore familiar with the types of studies that have entered our dataset, and where ambiguities might occur. Meta-data validation took place after the genetic validation, to avoid validating rows erroneously included in our dataset, and to ensure that the meta-data for any rows added by the genetic validation team was further examined by at least one more researcher. We validated all data rows by re-examining the papers against our refined definitions and each row was validated by a different researcher to its original extractor. Because these variables are described qualitatively in publications, this subgroup met weekly to discuss ambiguous data rows, until all rows were validated. Meetings ensured that classifications were recorded by consensus discussion, and maximised consistency among team members through frequent discussion of our definitions. Importantly, conservation interventions and ecological disturbances were recorded solely based on the information reported by authors in the extracted paper.

Supporting Information 2.11. Additional meta-analytic procedures

Sample sizes **–** Variance in population genetic analyses may be associated with both the number of individuals and the number of genomic positions sampled (i.e., loci, especially when a small number of loci are used). This complicates the estimation of effect size in meta-analysis, as increasing the sample size of both individuals and/or loci reduces variance across separate biological axes, depending on the study design, evolutionary process being quantified, and at what level (e.g., individual vs. population genetic diversity). Furthermore, population-level statistics may be summarised across populations or years, to provide inter-population or inter-year variances, respectively, in which case the number of populations or years is the appropriate sample size for calculating effect sizes for meta-analysis. In our analysis, we used the following hierarchy to establish the most appropriate sample size to apply to our effect size calculations, in order of priority:

1. For comparisons where the Marker Type = "Other" and/or Diversity Metric Type = "Other", the paper was manually checked to ascertain the associated sample size.
2. For comparisons where the Diversity Metric Type = "apFST" (i.e., among-population F_{ST}), the paper was checked to ascertain the associated sample size; usually the F_{ST} matrix size, but occasionally a different value as per the study design described within the paper.
3. If the mean and/or standard deviation of the genetic diversity measure was calculated by the data extractor, the denominator of the calculation was used.
4. If the Diversity Metric Type was AR (allelic richness), the rarefied AR sample size was used as collected during data extraction.
5. If the number of loci was recorded and > 1, the number of loci was used.
6. For remaining comparisons, the number of samples in the early and recent comparison were used for the $N_{(early)}$ and $N_{(recent)}$, respectively, or if these were not recorded, the total N was used for both $N_{(early)}$ and $N_{(recent)}$. For coalescent comparisons, either recent N or total N were used as both $N_{(early)}$ and $N_{(recent)}$.

Hedge's g* and its error **–** Effect sizes were calculated using the following formulae:

$$Hedge's\ g^* = \frac{\bar{Y}_{recent} - \bar{Y}_{early}}{\sqrt{\frac{(n_{recent} - 1)s^2_{recent} + (n_{early} - 1)s^2_{early}}{n_{recent} + n_{early} - 2}}} \times J$$

where,

$$J = 1 - \frac{3}{4(n_{recent} + n_{early} - 2) - 1},$$

And $\bar{Y}_{recent}, s^2_{recent}$ and n_{recent} are the respective mean, standard deviation, and sample size associated with the recent timepoint, and $\bar{Y}_{early}, s^2_{early}$ and n_{early} are associated with the early timepoint.

For each effect size, the error associated with the measure of genetic diversity change was converted to standard deviation for the calculation of Hedge's g* (at both the early and recent time-point for two time-point comparison and coalescent study designs). Conversions to standard deviation (SD) were undertaken as follows:

1. Standard error (SE):

$$SD = SE \times \sqrt{N}$$

2. Variance:

$$SD = \sqrt{Var}$$

3. 95% CI or highest posterior density credible intervals (HPD CIs):

$$SD = \sqrt{N} \times \frac{(upper\ limit - lower\ limit)}{3.92}$$

4. 90% CI or HPD:

$$SD = \sqrt{N} \times \frac{(upper\ limit - lower\ limit)}{3.29}$$

5. 80% CI or HPD:

$$SD = \sqrt{N} \times \frac{(upper\ limit - lower\ limit)}{2.56}$$

6. Interquartile range (IQR; following Wan et al.[109]):

$$SD \approx \frac{q_3 - q_1}{\eta(n)}$$

where q_3 and q_1 are the upper and lower interquartile values, respectively, and $\eta(n)$ is obtained from Table 2 of Wan et al.[109] for values of $Q \leq 50$, where

$$n = 4Q + 1$$

where n is the total sample size.

7. P-value: following section 6.5.2.3 of Cochrane v 6.2[110] we first obtained SE from the absolute value of the Student *t*-distribution corresponding to the p-value reported and sample size, using the qt function in the *base stats* R package. SE was then converted to SD using equation 1 above.

Meta-analysis base model – The base model is described in detail in the main Article Methods. R code specifying the weakly informative inverse-gamma model prior and model structure was as follows:

prior.basemodel <- list(R=list(V=diag(1), nu=0.002), G=list(G1=list(V=1, nu=0.002)))

basemodel <- MCMCglmm(HedgesG ~ Z.Generations + Z.YearMidpoint, random = ~ PaperID, mev=HedgesG.err, data=data, verbose=T, nitt=6000000, thin=5000, burnin=200000, prior=prior.basemodel, pr=T)

Where Z.Generations refers to the Z-standardised number of generations (i.e., number of years passed between the early and recent time-points, divided by generation length), Z.YearMidpoint refers to the Z-standardised study midpoint (year), PaperID refers to the study, HedgesG is the

Hedge's g* effect size, and HedgesG.err is the error associated with the Hedge's g* effect size calculation.

The base model and all other meta-analytic models described herein were executed in triplicate on the University of Sydney's High Performance Computer Artemis.

Phylogenetic modelling – Closely related species are likely to share life history traits and ecology, and therefore may share similar responses to disturbance and/or patterns of genetic diversity change. To account for non-independence of effect sizes among species with shared evolutionary history, we calculated the phylogenetic correlation between species using the Open Tree Taxonomy (OTT IDs described in Supporting Information 2.6). *Saccharomyces cf. cerevisiae* (OTT ID 7511391) was used as the outgroup to calculate the full variance-covariance matrix of phylogenetic relationships. Due to the wide phylogenetic diversity of species included in our study, it was not possible to accurately estimate branch lengths, so we calculated branch lengths based on topology alone using the *ape* package v 5.6.1[111].

Sensitivity testing of our base model – We observed several Hedge's g* values that were substantially larger (in absolute magnitude) than the bulk of the dataset. We therefore performed sensitivity testing of the base model to investigate the impact of the most extreme 1% of values (highest 0.5% and lowest 0.5% of all values) to examine their leverage on main conclusions. The extreme values were equivalent to 38 rows of data and were removed symmetrically from the upper and lower ranges to avoid biasing our parameter estimates. The data contributing to extreme values were further examined manually. We also performed sensitivity testing of the base model to investigate the impact of phylogenetic co-variance on model results, either with or without the extreme values, by fitting phylogeny as a random effect. Extreme values were determined to have undue influence on the base model, so were removed from further modelling so that our findings represent the bulk (99%) of literature available for genetic diversity change (see Supporting Information 1.4 for further details).

Supporting Information 2.12. Meta-regression

Study design – Four meta-regressions were used to investigate the impact of genetic diversity change measurement methods on genetic diversity change estimates. Firstly, study duration was investigated, and categorised as short (< 5 years), medium (between 5 and 30 years inclusive) or long (> 30 years), with short being the reference category. This meta-regression excluded the fixed effect of Year (Midpoint), which was fit in all other models, due to the correlation between midpoint and duration (i.e., shorter studies tended to be more recent). Next, the statistical method (linear measure of change, two time-point comparison, and coalescent analysis) was investigated by meta-regression as a categorical predictor with linear measures of genetic diversity change being the reference category. The third study design meta-regression explored the genetic marker type used to measure genetic diversity change, with microsatellites set to the reference category. As there was an existing "Other" category, marker types with < 20 data points (amino acid, chromosomal, minisatellite, and mixed) were also collapsed into this category. Finally, a meta-regression of the genetic diversity metric type (variant counts, variant frequencies, individual-level variation, or integrated statistics) used to measure genetic diversity change was modelled. All sample sizes used for study design meta-regressions can be found in Supporting Information 1.6.

Population context – We fit three meta-regressions to investigate where genetic diversity change is measured, and in what species (including the threat status of study species). First, we fit a meta-regression with realm as the categorical moderator variable. Realms with less than 10 effect sizes were removed (the majority of these were marine), with Palearctic as the reference category. Next, we examined the threat status of the focal species as a moderator of genetic diversity change by meta-regression. We used the IUCN Red List to group species into Threatened (IUCN Red List categories: EX, EW, CR, EN, VU); Non-threatened (IUCN Red List categories: NT and LC); and Unknown (IUCN Red List category DD or Not Evaluated). We set non-threatened as the reference category. Finally, despite low phylogenetic signal, we investigated whether coarser taxonomic relationships impacted genetic diversity change estimates via a meta-regression of taxonomic class. Classes with < 10 effect sizes were removed from modelling, with Mammalia as the reference category. As we expected that studies of domesticated, pest, or pathogen species may show different trends to other species, we subset the taxonomic class dataset to species identified as domesticated, pest, or pathogen, retaining classes with 10 or more effect sizes. All sample sizes used for population context meta-regressions can be found in Supporting Information 1.7.

Threats – To investigate the impact of ecological disturbance on genetic diversity change, we first modelled a meta-regression comparing any reported disturbance to cases where no disturbance was reported (i.e., "any" or "none"), with "none" being the reference category. In a separate model, we then evaluated the impact of the different types of ecological disturbances on genetic diversity change. As studies may report multiple different disturbances, the model structure differed from the above meta-regressions. The various disturbances were coded as "1" if they were reported for a given effect size, or "0" if not reported. Each disturbance was included in the model as an independent, categorical, moderator variable, where the intercept of the model represents all disturbances held to "0". All sample sizes used for ecological disturbance meta-regressions can be found in Supporting Information 1.8.

Conservation management – Similar to the threat meta-regressions, we first modelled a meta-regression comparing any reported conservation management action to cases where no management was reported (i.e., "any" or "none"), with "none" being the reference category. We also performed a meta-regression comparing "legal protection", to any other reported conservation management action, and cases where no management was reported (i.e., "legal" or "any" or "none"), to capture the difference between passive and active management actions (reference category = "none"). In a separate model, we then investigated the impact of individual conservation management actions on

genetic diversity change estimates by coding each action as "1" if it was reported or "0" if not reported, and fitting each as an independent, categorical moderator where the intercept represents all interventions held to "0". We considered that the genetic diversity change estimates across conservation interventions may differ as a result of the IUCN Red List threat status of the focal species. Therefore, we also fit sub-models of the conservation management actions for each threat status (threatened; non-threatened; and unknown threat status). Only interventions with 10 or more effect sizes in the subgroups were modelled. All sample sizes used for conservation management meta-regressions can be found in Supporting Information 1.9.

Conservation management actions by taxonomic class – We also considered that the impact of conservation management on genetic diversity change estimates may differ between taxonomic classes. Therefore, we modelled conservation management actions for which there were 10 or more effect sizes for each of the five taxonomic classes with the most data (Actinopterygii, Mammalia, Insecta, Magnoliopsida, and Aves) (Supporting Information 1.7). Prior to fitting meta-regressions for individual actions, we tested whether there were any substantial correlations between actions in these taxa, across the data subsets used for modelling. We calculated pairwise Spearman's rank correlation coefficients (r_s) between conservation management actions in R[97], and combined variables for which Spearman's $r_s \geq 0.5$ to describe co-occurring actions. Within these subgroups, there were two instances where there was sufficient data for modelling (i.e., 10 or more effect sizes) and the conservation management actions were correlated and so could not be modelled as independent moderator variables. Ecological restoration and feral and pest control were correlated in Aves ($r = 0.54$), and ecological restoration and supplementation were correlated in Mammalia (0.58), suggesting that these actions were often reported together (Supporting Table 16). As such, the correlated management actions were combined and modelled as one moderator representing the joint action. For example, in Mammalia, a joint moderator representing the presence of either (or both) ecological restoration and supplementation was instead fit. We also found moderate and significant negative correlations (range = -0.53 to -0.68) between "none" (i.e., no conservation management) and three other variables ("legal protection", "supplementation", and "conservation introduction") across taxonomic classes (Supporting Table 16), reflecting conservation management actions that tended to occur in isolation. However, as described above, we did not include the "none" category per se, rather, our intercept represents all interventions held to "0" and so actions negatively correlated with "none" were still included in the modelling. The variables in the remaining pairwise comparisons were not included in subsequent modelling, as they were calculated for conservation management actions that impacted fewer than 10 effect sizes. All sample sizes used for conservation management meta-regressions for taxonomic classes can be found in Supporting Information 1.9.

Management intensity – We examined whether the intensity of management was a moderator of genetic diversity change via meta-regression. The data were subset to remove the "unknown" and "none" categories of conservation management action intensity, leaving the "individual", "indirect", "population" and "mixed" level management categories. Management intensity was modelled as a categorical moderator with "individual" as the reference. All sample sizes used for conservation management intensity meta-regression can be found in Supporting Information 1.9.

Supporting Table 16. Spearman's pairwise correlations between conservation management actions, where r ≥ |0.5|, for the meta-analysis dataset (i.e., excluding extreme values) across the five most data-rich taxonomic classes. Note that correlated variables for Insecta were not combined, as there was insufficient data for modelling (fewer than 10 effect sizes).

Class	Variable 1	Variable 2	r	p
Actinopterygii	Legal protection (L)	None (N)	-0.68	1.05×10^{-203}
	Supplementation (S)	None (N)	-0.59	7.20×10^{-142}
Aves	Legal protection (L)	None (N)	-0.53	1.57×10^{-23}
	Ecological restoration (E)	Feral and pest control (F)	0.54	7.70×10^{-24}
Insecta	Legal protection (L)	None (N)	-0.68	1.16×10^{-64}
	Population control (P)	Supplementation (S)	0.58	4.09×10^{-42}
	Temporary resources (T)	Supplementation (S)	0.61	3.89×10^{-48}
	Temporary resources (T)	Feral and pest control (F)	0.61	3.89×10^{-48}
Magnoliopsida	Conservation introduction (I)	None (N)	-0.59	2.54×10^{-31}
Mammalia	Legal protection (L)	None (N)	-0.57	2.68×10^{-53}
	Ecological restoration (E)	Supplementation (S)	0.58	5.61×10^{-56}

References

67. Peel, D., Ovenden, J. R. & Peel, S. L. NeEstimator: software for estimating effective population size [online]. Version 1.3. Queensland Government, Department of Primary Industries and Fisheries (2004).
68. Waples, R. S. & Do, C. LDNE: a program for estimating effective population size from data on linkage disequilibrium. *Molecular Ecology Resources* **8**, 753–756 (2008).
69. Do, C. *et al.* NeEstimator v2: re-implementation of software for the estimation of contemporary effective population size (N_e) from genetic data. *Molecular Ecology Resources* **14**, 209–214 (2014).
70. Waples, R. S. & Do, C. Linkage disequilibrium estimates of contemporary N_e using highly variable genetic markers: a largely untapped resource for applied conservation and evolution. *Evolutionary Applications* **3**, 244–262 (2010).
71. Jones, A. T., Ovenden, J. R. & Wang, Y. G. Improved confidence intervals for the linkage disequilibrium method for estimating effective population size. *Heredity* **117**, 217–223 (2016).
72. Baillie, S. M., Muir, A. M., Scribner, K., Bentzen, P. & Krueger, C. C. Loss of genetic diversity and reduction of genetic distance among lake trout *Salvelinus namaycush* ecomorphs, Lake Superior 1959 to 2013. *Journal of Great Lakes Research* **42**, 204–216 (2016).
73. Narum, S. R., Boe, S., Moran, P. & Powell, M. Small-scale genetic structure and variation in steelhead of the Grande Ronde River, Oregon, USA. *Trans Am Fish Soc* **135**, 979–986 (2006).
74. Hoffman, J. I., Grant, S. M., Forcada, J. & Phillips, C. D. Bayesian inference of a historical bottleneck in a heavily exploited marine mammal. *Molecular Ecology* **20**, 3989–4008 (2011).
75. Casas-Marce, M. *et al.* Spatiotemporal Dynamics of Genetic Variation in the Iberian Lynx along Its Path to Extinction Reconstructed with Ancient DNA. *Molecular Biology and Evolution* **34**, 2893–2907 (2017).
76. Ishibashi, Y. *et al.* Loss of allelic diversity in the MHC class II *DQB* gene in western populations of the Japanese black bear *Ursus thibetanus japonicus*. *Conserv Genet* **18**, 247–260 (2017).
77. Weber, D. S., Stewart, B. S. & Lehman, N. Genetic consequences of a severe population bottleneck in the Guadalupe fur seal (*Arctocephalus townsendi*). *Journal of Heredity* **95**, 144–153 (2004).
78. Molfetti, E. *et al.* Recent demographic history and present fine-scale structure in the northwest Atlantic leatherback (*Dermochelys coriacea*) turtle population. *PLoS ONE* **8**, e58061 (2013).
79. Nabholz, B. *et al.* Transcriptome population genomics reveals severe bottleneck and domestication cost in the African rice (*Oryza glaberrima*). *Molecular Ecology* **23**, 2210–2227 (2014).
80. Miura, O. *et al.* Ecological and genetic impact of the 2011 Tohoku Earthquake Tsunami on intertidal mud snails. *Sci Rep* **7**, 44375 (2017).
81. Janecka, J. E. *et al.* Loss of genetic diversity among ocelots in the United States during the 20th century linked to human induced population reductions. *PLoS ONE* **9**, e89384 (2014).
82. Pacioni, C. *et al.* Genetic diversity loss in a biodiversity hotspot: ancient DNA quantifies genetic decline and former connectivity in a critically endangered marsupial. *Mol Ecol* **24**, 5813–5828 (2015).
83. Johnson, B. M., Kemp, B. M. & Thorgaard, G. H. Increased mitochondrial DNA diversity in ancient Columbia River basin Chinook salmon *Oncorhynchus tshawytscha*. *PLoS ONE* **13**, e0190059 (2018).
84. Gallardo, M. H. & Köhler, N. Demographic changes and genetic losses in populations of a subterranean rodent (*Ctenomys maulinus brunneus*) affected by a natural catastrophe. *Zeitschrift für Säugetierkunde* **59**, 358–365 (1994).
85. Ciborowski, K. L. *et al.* Stocking may increase mitochondrial DNA diversity but fails to halt the decline of endangered Atlantic salmon populations. *Conserv Genet* **8**, 1355–1367 (2007).
86. Ciborowski, K., Jordan, W. C., Garcia de Leaniz, C. & Consuegra, S. Temporal and spatial instability in neutral and adaptive (MHC) genetic variation in marginal salmon populations. *Sci Rep* **7**, 42416 (2017).
87. Fennell, M., Gallagher, T., Vintro, L. L. & Osborne, B. Using soil seed banks to assess temporal patterns of genetic variation in invasive plant populations. *Ecol Evol* **4**, 1648–1658 (2014).
88. Negro, S. *et al.* Molecular diversity between two cohorts of six Spanish riding-horse breeds: impact of selection in crossbred vs purebred populations. *Livestock Science* **193**, 88–91 (2016).
89. Biermann, A. D. M., Pimentel, E. C. G., Tietze, M., Pinent, T. & König, S. Implementation of genetic evaluation and mating designs for the endangered local pig breed 'Bunte Bentheimer'. *J Animal Breeding Genetics* **131**, 36–45 (2014).
90. Rizzi, R., Tullo, E., Cito, A. M., Caroli, A. & Pieragostini, E. Monitoring of genetic diversity in the endangered Martina Franca donkey population. *Journal of Animal Science* **89**, 1304–1311 (2011).
91. Roos, L., Hinrichs, D., Nissen, T. & Krieter, J. Investigations into genetic variability in Holstein horse breed using pedigree data. *Livestock Science* **177**, 25–32 (2015).
92. Wellmann, R., Hartwig, S. & Bennewitz, J. Optimum contribution selection for conserved populations with historic migration. *Genet Sel Evol* **44**, 34 (2012).
93. Marfo, P. & Okyere, G. A. The accuracy of effect-size estimates under normals and contaminated normals in meta-analysis. *Heliyon* **5**, e01838 (2019).
94. Taylor, L. R. Aggregation, variance and the mean. *Nature* **189**, 732–735 (1961).

95. Grissom, R. J. Heterogeneity of variance in clinical data. *Journal of Consulting and Clinical Psychology* **68**, 155–165 (2000).
96. Senior, A. M. *et al.* Heterogeneity in ecological and evolutionary meta-analyses: its magnitude and implications. *Ecology* **97**, 3293–3299 (2016).
97. R Core Team. *R: A Language and Environment for Statistical Computing*. (R Foundation for Statistical Computing, Vienna, Austria, 2024).
98. Westgate, M. J. revtools: an R package to support article screening for evidence synthesis. *Research Synthesis Methods* **10**, 606–614 (2019).
99. Corporation for Digital Scholarship. Zotero [Software]. Version 5.0.60. (2023).
100. The EndNote Team. EndNote X9. Clarivate (2018).
101. Michonneau, F., Brown, J. W. & Winter, D. J. rotl: an R package to interact with the Open Tree of Life data. *Methods in Ecology and Evolution* **7**, 1476–1481 (2016).
102. Rees, J. & Cranston, K. Automated assembly of a reference taxonomy for phylogenetic data synthesis. *Biodiversity Data Journal* **5**, e12581 (2017).
103. IUCN. *Guidelines for Appropriate Uses of IUCN Red List Data. Incorporating, as Annexes, the 1) Guidelines for Reporting on Proportion Threatened (Ver. 1.1); 2) Guidelines on Scientific Collecting of Threatened Species (Ver. 1.0); and 3) Guidelines for the Appropriate Use of the IUCN Red List by Business (Ver. 1.0)*. (2016).
104. Fung, H. C. & Waples, R. S. Performance of IUCN proxies for generation length. *Conservation Biology* **31**, 883–893 (2017).
105. Pacifici, M. *et al.* Generation length for mammals. *Nature Conservation* **5**, 87–94 (2013).
106. Bird, J. P. *et al.* Generation lengths of the world's birds and their implications for extinction risk. *Conservation Biology* **34**, 1252–1261 (2020).
107. Zeder, M. A. Core questions in domestication research. *Proc Natl Acad Sci U S A* **112**, 3191–3198 (2015).
108. Prentis, P. J., Wilson, J. R. U., Dormontt, E. E., Richardson, D. M. & Lowe, A. J. Adaptive evolution in invasive species. *Trends in Plant Science* **13**, 288–294 (2008).
109. Wan, X., Wang, W., Liu, J. & Tong, T. Estimating the sample mean and standard deviation from the sample size, median, range and/or interquartile range. *BMC Medical Research Methodology* **14**, 135 (2014).
110. *Cochrane Handbook for Systematic Reviews of Interventions*. vol. Version 6.2 (updated February 2021) (Cochrane, 2011).
111. Paradis, E. & Schliep, K. ape 5.0: an environment for modern phylogenetics and evolutionary analyses in R. *Bioinformatics* **35**, 526–528 (2019).
