# Supplementary material for: Global meta-analysis shows action is needed to halt genetic diversity loss
Source: Nature. 2025 Jan 29;638(8051):704–10. doi: 10.1038/s41586-024-08458-x (PMC11839457; doi:10.1038/s41586-024-08458-x)
Supplement: Supplementary file 2 — Reporting Summary [file 41586_2024_8458_MOESM2_ESM.pdf]

Corresponding author(s): Catherine Grueber
Last updated by author(s): Oct 8, 2024

Reporting Summary

Nature Portfolio wishes to improve the reproducibility of the work that we publish. This form provides structure for consistency and transparency in reporting. For further information on Nature Portfolio policies, see our Editorial Policies and the Editorial Policy Checklist.

Statistics

For all statistical analyses, confirm that the following items are present in the figure legend, table legend, main text, or Methods section.

n/a	Confirmed	
☐	☒	The exact sample size (*n*) for each experimental group/condition, given as a discrete number and unit of measurement
☐	☒	A statement on whether measurements were taken from distinct samples or whether the same sample was measured repeatedly
☐	☒	The statistical test(s) used AND whether they are one- or two-sided<br>*Only common tests should be described solely by name; describe more complex techniques in the Methods section.*
☐	☒	A description of all covariates tested
☐	☒	A description of any assumptions or corrections, such as tests of normality and adjustment for multiple comparisons
☐	☒	A full description of the statistical parameters including central tendency (e.g. means) or other basic estimates (e.g. regression coefficient) AND variation (e.g. standard deviation) or associated estimates of uncertainty (e.g. confidence intervals)
☐	☒	For null hypothesis testing, the test statistic (e.g. *F*, *t*, *r*) with confidence intervals, effect sizes, degrees of freedom and *P* value noted<br>*Give P values as exact values whenever suitable.*
☐	☒	For Bayesian analysis, information on the choice of priors and Markov chain Monte Carlo settings
☐	☒	For hierarchical and complex designs, identification of the appropriate level for tests and full reporting of outcomes
☐	☒	Estimates of effect sizes (e.g. Cohen's *d*, Pearson's *r*), indicating how they were calculated

Our web collection on statistics for biologists contains articles on many of the points above.

Software and code

Policy information about availability of computer code

Data collection	All data used in this study were obtained from published and publicly available sources, as indicated at the “Data” section.
Data analysis	Custom text mining code is available on Zenodo DOI: 10.5281/zenodo.13903787 ref#66. Effect size calculation was conducted via established equations provided in Supporting Information 2.11. Meta-analysis was conducted with the MCMCglmm package v 2.34 in R v 3.5.2, with the model equation provided in Supporting Information 2.11. Phylogenetic modelling for sensitivity testing of the base model used phylogenetic relationships established via the Open Tree of Life and the ape package v 5.6.1 in R. We also used the following R-packages: pdfsearch v 0.2.3, dplyr v 0.8.0, stringi v 1.3.1, revtools v 0.4.0, ggplot2 v 3.4.3, treemapify v 2.5.5, ggridges v 0.5.4, corrplot v 0.92, factoextra v 1.0.7, rotl v 3.0.12, ggtree v 3.8.2, ggtreeExtra v 1.10.0, ggimage v 0.3.3, rphylopic v 1.2.1, coda v 0.19.4, and the following software: Zotero v 5.0.60, Endnote v X9 and GetData Graph Digitizer v 2.26.

For manuscripts utilizing custom algorithms or software that are central to the research but not yet described in published literature, software must be made available to editors and reviewers. We strongly encourage code deposition in a community repository (e.g. GitHub). See the Nature Portfolio guidelines for submitting code & software for further information.

Data

Policy information about availability of data

All manuscripts must include a data availability statement. This statement should provide the following information, where applicable:

- Accession codes, unique identifiers, or web links for publicly available datasets
- A description of any restrictions on data availability
- For clinical datasets or third party data, please ensure that the statement adheres to our policy

All datasets associated with this paper are available on Zenodo DOI: 10.5281/zenodo.13903787 [66]. The full bibliography of 882 included papers (including their DOIs) are listed in Supporting Data 1. We used publicly available databases to obtain species characteristics for the 628 species included in our study. Full methods are in Supporting Information 2.6. Generation lengths (see Supporting Data 5) were obtained from scientific literature and databases including Search FishBase (www.fishbase.se/search.php), AmphibiaWeb (www.amphibiaweb.org), CABI Compendium (www.cabidigitallibrary.org/journal/cabicompendium). Threat status was sourced from the IUCN Red List of Threatened Species [30] at June-August 2021. Invasive species status was sourced from the IUCN 100 of the World's Worst Invasive Alien Species list (www.iucngisd.org/gisd/100_worst.php). Pathogen and pest statuses were sourced from the scientific literature and databases including the European and Mediterranean Plant Protection Organization Global Database (gd.eppo.int/), The Global Pest and Disease Database (www.gpdd.info), and CABI Compendium (www.cabi.org/isc).

Research involving human participants, their data, or biological material

Policy information about studies with human participants or human data. See also policy information about sex, gender (identity/presentation), and sexual orientation and race, ethnicity and racism.

Reporting on sex and gender	N/A
Reporting on race, ethnicity, or other socially relevant groupings	N/A
Population characteristics	N/A
Recruitment	N/A
Ethics oversight	N/A

Note that full information on the approval of the study protocol must also be provided in the manuscript.

Field-specific reporting

Please select the one below that is the best fit for your research. If you are not sure, read the appropriate sections before making your selection.

☐ Life sciences ☐ Behavioural & social sciences ☒ Ecological, evolutionary & environmental sciences

For a reference copy of the document with all sections, see nature.com/documents/nr-reporting-summary-flat.pdf

Ecological, evolutionary & environmental sciences study design

All studies must disclose on these points even when the disclosure is negative.

Study description	A systematic review and meta-analysis of the literature on genetic change over recent (human-impacted) timescales. Measures of genetic change were extracted from the published literature and converted to Hedge's g* effect sizes for meta-analysis (n=4021 effect sizes). Hierarchical MCMCglmm models were fit to account for non-independence as a result of multiple datapoints per study (StudyID random factor), with sensitivity testing to examine the impact of non-independence as a result of phylogenetic relationships between species.
Research sample	This study uses data extracted from the published literature. Reporting of the systematic review and meta-analysis follows the global best-practice PRISMA guidelines. Briefly, publications were identified using search strings to query the Web of Science database. Text mining was conducted to refine search results, before manual examination of 34,346 publications. Relevant data were manually extracted as per our study protocol, and data extractions validated by independent authors. A total of 4021 datapoints from 882 publications and 628 species, covering 37 taxonomic classes, were obtained for meta-analysis. All details of the systematic review and meta-analysis are reported either in the Main Article or Supplementary Methods.
Sampling strategy	The search string was designed to avoid hypothesis-driven bias around genetic erosion. For example, we included symmetrical search terms such as "gain" and "loss", the text mining related to methods rather than the magnitude or direction of any genetic change, and our manual extraction protocol was agnostic to the directionality of any genetic change (i.e. we reported measures of genetic diversity over time regardless of whether change was observed or not). This resulted in a large dataset of 4021 datapoints.
Data collection	After identifying studies meeting our inclusion criteria (Supporting Information 2), we manually extracted data from published

Data collection	records into an Excel template as per the study protocol. A randomly selected subset of 150 papers was independently (and blindly) re-extracted to examine reproducibility. As a result, all data were checked and validated by two teams, one examining the inclusion criteria and genetic data, and the other examining the conservation and ecological disturbance metadata.
Timing and spatial scale	The systematic search of the literature was conducted on 18 January 2019. Text mining was conducted shortly thereafter. The manual screening of studies per the inclusion criteria and extraction of data from studies meeting the criteria began at a workshop in Tovetorp, Sweden, in March 2020. Data was collected from papers published between 1985 and 2019 (noting that there was no time constraint on year of publication in the search). The data in this study is not limited by geographic location, and represents the global literature on genetic diversity change. Measures of genetic change span from 10,486 BCE to 2018 CE.
Data exclusions	Data were excluded at various steps as reported in the PRISMA flowchart (Extended Figure 1 and Supporting Information 1.1). Data was excluded if it did not meet our pre-specified inclusion criteria, such as by not reporting genetic metrics, if it was duplicated in the dataset, or because statistical measures of error were not reported in the primary study. Additional data was excluded if the statistic was directionless for genetic change (e.g., FST), could not be converted to effect sizes (e.g., datapoints with infinite measures of variance), or as outliers. Sensitivity testing was performed to examine the impact of excluding extreme values. All details of data exclusions are reported in the Supporting Information.
Reproducibility	A subset of data extractions were independently and blindly re-extracted by other members of the team to the original extractors. As a result, all data were re-examined and validated by two teams as described in the Supporting Information 2.10 (Repeat extractions and Validation). All Bayesian MCMCglmm meta-analytic models were run in triplicate to calculate a Gelman-Rubin convergence diagnostic of <1.1, ensuring that model results were consistently reproducible and not subject to chain divergence.
Randomization	The systematic review dataset after text mining of 34,346 publications was grouped thematically into 16 groups (Supporting Table 2.2a) based on text mining of keywords. Within these groups, studies were randomly split into batches of 100 papers for manual screening, and authors randomly selected a batch within a theme of their knowledge to screen. No further randomization was applicable in this study.
Blinding	A subset of extracted data from 150 papers was re-examined by independent members of the team that were blind to the original extractions to examine reproducibility of the study extraction protocol. As a result, more targeted efforts were conducted to validate both the genetic and metadata fields of the entire dataset.

Did the study involve field work? ☐ Yes ☒ No

Reporting for specific materials, systems and methods

We require information from authors about some types of materials, experimental systems and methods used in many studies. Here, indicate whether each material, system or method listed is relevant to your study. If you are not sure if a list item applies to your research, read the appropriate section before selecting a response.

Materials & experimental systems

n/a	Involved in the study
☒	☐ Antibodies
☒	☐ Eukaryotic cell lines
☒	☐ Palaeontology and archaeology
☒	☐ Animals and other organisms
☒	☐ Clinical data
☒	☐ Dual use research of concern
☒	☐ Plants

Methods

n/a	Involved in the study
☒	☐ ChIP-seq
☒	☐ Flow cytometry
☒	☐ MRI-based neuroimaging

Plants

Seed stocks	*Report on the source of all seed stocks or other plant material used. If applicable, state the seed stock centre and catalogue number. If plant specimens were collected from the field, describe the collection location, date and sampling procedures.*
Novel plant genotypes	*Describe the methods by which all novel plant genotypes were produced. This includes those generated by transgenic approaches, gene editing, chemical/radiation-based mutagenesis and hybridization. For transgenic lines, describe the transformation method, the number of independent lines analyzed and the generation upon which experiments were performed. For gene-edited lines, describe the editor used, the endogenous sequence targeted for editing, the targeting guide RNA sequence (if applicable) and how the editor was applied.*
Authentication	*Describe any authentication procedures for each seed stock used or novel genotype generated. Describe any experiments used to assess the effect of a mutation and, where applicable, how potential secondary effects (e.g. second site T-DNA insertions, mosiacism, off-target gene editing) were examined.*
