# Supplementary material for: Global meta-analysis shows action is needed to halt genetic diversity loss
Source: Nature. 2025 Jan 29;638(8051):704–10. doi: 10.1038/s41586-024-08458-x (PMC11839457; doi:10.1038/s41586-024-08458-x)
Supplement: Supplementary file 4 — Bibliography for systematic review dataset. [file 41586_2024_8458_MOESM4_ESM.pdf]

SUPPORTING DATA 1: BIBLIOGRAPHY

1. Adcock, G. J., Shaw, P. W., Rodhouse, P. G., & Carvalho, G. R. (1999). Microsatellite analysis of genetic diversity in the squid Illex argentinus during a period of intensive fishing. Marine Ecology Progress Series, 187, 171–178. doi: 10.3354/meps187171

2. Abbott, J. K., Bensch, S., Gosden, T. P., & Svensson, E. I. (2008). Patterns of differentiation in a colour polymorphism and in neutral markers reveal rapid genetic changes in natural damselfly populations. Molecular Ecology, 17, 1597–1604. doi: 10.1111/j.1365-294X.2007.03641.x

3. Abdelkrim, J., Hunt, G. R., Gray, R. D., & Gemmell, N. J. (2012). Population genetic structure and colonisation history of the tool-using New Caledonian crow. PLoS ONE, 7, e36608. doi: 10.1371/journal.pone.0036608

4. Abdelkrim, J., Pascal, M., & Samadi, S. (2005). Island colonization and founder effects: the invasion of the Guadeloupe islands by ship rats (Rattus rattus). Molecular Ecology, 14, 2923–2931. doi: 10.1111/j.1365-294X.2005.02604.x

5. Abreu, A. G., & Solferini, V. N. (2008). Genetic population structure and hybridization in two sibling species, Tomoplagia reticulata and Tomoplagia pallens (Diptera: Tephritidae). Genetics and Molecular Research, 7, 1298–1311. doi: 10.4238/vol7-4gmr534

6. Adams, J. R., Vucetich, L. M., Hedrick, P. W., Peterson, R. O., & Vucetich, J. A. (2011). Genomic sweep and potential genetic rescue during limiting environmental conditions in an isolated wolf population. Proceedings of the Royal Society B, 278, 3336–3344. doi: 10.1098/rspb.2011.0261

7. Aglieri, G., Papetti, C., Zane, L., Milisenda, G., Boero, F., & Piraino, S. (2014). First evidence of inbreeding, relatedness and chaotic genetic patchiness in the holoplanktonic jellyfish Pelagia noctiluca (Scyphozoa, Cnidaria). PLoS ONE, 9, e99647. doi: 10.1371/journal.pone.0099647

8. Aguilar, A., Banks, J. D., Levine, K. F., & Wayne, R. K. (2005). Population genetics of northern pike (Esox lucius) introduced into Lake Davis, California. Canadian Journal of Fisheries and Aquatic Sciences, 62, 1589–1599. doi: 10.1139/f05-068

9. Akimoto, M., Shimamoto, Y., & Morishima, H. (1999). The extinction of genetic resources of Asian wild rice, Oryza rufipogon Griff.: A case study in Thailand. Genetic Resources and Crop Evolution, 46, 419–425. doi: 10.1023/A:1008622405001

10. Al-Sadi, A. M., Al-Ghaithi, A. G., Al-Balushi, Z. M., & Al-Jabri, A. H. (2012). Analysis of diversity in Pythium aphanidermatum populations from a single greenhouse reveals phenotypic and genotypic changes over 2006 to 2011. Plant Disease, 96, 852–858. doi: 10.1094/PDIS-07-11-0624

11. Albaladejo, R. G., González-MartÍnez, S. C., Heuertz, M., Vendramin, G. G., & Aparicio, A. (2009). Spatiotemporal mating pattern variation in a wind-pollinated Mediterranean shrub. Molecular Ecology, 18, 5195–5206. doi: 10.1111/j.1365-294X.2009.04415.x

12. Alfsnes, K., Hobæk, A., Weider, L. J., & Hessen, D. O. (2016). Birds, nutrients, and climate change: mtDNA haplotype diversity of Arctic Daphnia on Svalbard revisited. Polar Biology, 39, 1425–1437. doi: 10.1007/s00300-015-1868-8

13. Ali, S., Soubeyrand, S., Gladieux, P., Giraud, T., Leconte, M., Gautier, A., … Enjalbert, J. (2016). cloncase: Estimation of sex frequency and effective population size by clonemate resampling in partially clonal organisms. Molecular Ecology Resources, 16, 845–861. doi: 10.1111/1755-0998.12511

14. Allen, D. E., & Lynch, M. (2012). The effect of variable frequency of sexual reproduction on the genetic structure of natural populations of a cyclical parthenogen. Evolution, 66, 919–926. doi: 10.1111/j.1558-5646.2011.01488.x

15. Almathen, F., Charruau, P., Mohandesan, E., Mwacharo, J. M., Orozco-TerWengel, P., Pitt, D., … Burger, P. A. (2016). Ancient and modern DNA reveal dynamics of domestication and cross-continental dispersal of the dromedary. Proceedings of the National Academy of Sciences of the United States of America, 113, 6707–6712. doi: 10.1073/pnas.1519508113

16. Alström-Rapaport, C., Leskinen, E., & Pamilo, P. (2010). Seasonal variation in the mode of reproduction of Ulva intestinalis in a brackish water environment. Aquatic Botany, 93, 244–249. doi: 10.1016/j.aquabot.2010.08.003

17. Alter, S. E., Newsome, S. D., & Palumbi, S. R. (2012). Pre-whaling genetic diversity and population ecology in eastern pacific gray whales: Insights from ancient DNA and stable isotopes. PLoS ONE, 7, e35039. doi: 10.1371/journal.pone.0035039

18. Altukhov, Y. P., & Salmenkova, E. A. (1991). The genetic structure of salmon populations. Aquaculture, 98, 11–40. doi: 10.1016/0044-8486(91)90368-H

19. Alves, F. M., Sartori, Â. L. B., Zucchi, M. I., Azevedo-Tozzi, A. M. G., Tambarussi, E. V, & De Souza, A. P. (2018). A high level of outcrossing in the vulnerable species Prosopis rubriflora in a Chaco remnant. Australian Journal of Botany, 66, 360–368. doi: 10.1071/BT17195

20. Alves, M. L., Belo, M., Carbas, B., Brites, C., Paulo, M., Mendes-Moreira, P., … Vaz Patto, M. C. (2018). Long-term on-farm participatory maize breeding by stratified mass selection retains molecular diversity while improving agronomic performance. Evolutionary Applications, 11, 254–270. doi: 10.1111/eva.12549

21. Amavet, P. S., Rueda, E. C., Vilardi, J. C., Siroski, P., Larriera, A., & Saidman, B. O. (2017). The broad-snouted caiman population recovery in Argentina. A case of genetics conservation. Amphibia Reptilia, 38, 411–424. doi: 10.1163/15685381-00003123

22. Amouroux, P., Normand, F., Nibouche, S., & Delatte, H. (2013). Invasive mango blossom gall midge, Procontarinia mangiferae (Felt) (Diptera: Cecidomyiidae) in Reunion Island: ecological plasticity, permanent and structured populations. Biological Invasions, 15, 1677–1693. doi: 10.1007/s10530-012-0400-0

23. Andersen, L. W., Born, E. W., Dietz, R., Haug, T., Øien, N., & Bendixen, C. (2003). Genetic population structure of minke whales Balaenoptera acutorostrata from Greenland, the North East Atlantic and the North Sea probably reflects different ecological regions. *Marine Ecology Progress Series*, *247*, 263–280. doi: 10.3354/meps247263

24. Andersen, L. W., Jacobsen, M. W., Lydersen, C., Semenova, V., Boltunov, A., Born, E. W., … Kovacs, K. M. (2017). Walruses (Odobenus rosmarus rosmarus) in the Pechora Sea in the context of contemporary population structure of Northeast Atlantic walruses. Biological Journal of the Linnean Society, 122, 897–915. doi: 10.1093/BIOLINNEAN/BLX093

25. Anderson, A. P., Denson, M. R., & Darden, T. L. (2014). Genetic Structure of Striped Bass in the Southeastern United States and Effects from Stock Enhancement. North American Journal of Fisheries Management, 34, 653–667. doi: 10.1080/02755947.2014.902409

26. Andrivon, D. (1994). Races of Phytophthora infestans in France, 1991-1993. *Potato Research*, *37*, 279–286. doi: 10.7868/s0555109916010153

27. Angeletti, D., Cimmaruta, R., & Nascetti, G. (2010). Genetic diversity of the killifish Aphanius fasciatus paralleling the environmental changes of Tarquinia salterns habitat. Genetica, 138, 1011–1021. doi: 10.1007/s10709-010-9487-3

28. Angeletti, D., Cimmaruta, R., Sebbio, C., Bellisario, B., Carere, C., & Nascetti, G. (2017). Environmental heterogeneity promotes microgeographic genetic divergence in the Mediterranean killifish Aphanius fasciatus (Cyprinodontidae). Ethology Ecology and Evolution, 29, 367–386. doi: 10.1080/03949370.2016.1188159

29. Ardren, W. R., & Kapuscinski, A. R. (2003). Demographic and genetic estimates of effective population size (Ne) reveals genetic compensation in steelhead trout. Molecular Ecology, 12, 35–49. doi: 10.1046/j.1365-294X.2003.01705.x

30. Arnaud-Haond, S., Vonau, V., Bonhomme, F., Boudry, P., Blanc, F., Prou, J., … Goyard, E. (2004). Spatio-temporal variation in the genetic composition of wild populations of pearl oyster (Pinctada margaritifera cumingii) in French Polynesia following 10 years of juvenile translocation. Molecular Ecology, 13, 2001–2007. doi: 10.1111/j.1365-294X.2004.02188.x

31. Arrendal, J., Walker, C. W., Sundqvist, A. K., Hellborg, L., & Vilà, C. (2004). Genetic evaluation of an otter translocation program. Conservation Genetics, 5, 79–88. doi: 10.1023/B:COGE.0000014059.49606.dd

32. Arruda, C. C. B., Silva, M. B., Sebbenn, A. M., Kanashiro, M., Lemes, M. R., & Gribel, R. (2015). Mating system and genetic diversity of progenies before and after logging: a case study of Bagassa guianensis (Moraceae), a low-density dioecious tree of the Amazonian forest. Tree Genetics and Genomes, 11, 1–9. doi: 10.1007/s11295-015-0837-2

33. Aspi, J., Roininen, E., Ruokonen, M., Kojola, I., & Vilà, C. (2006). Genetic diversity, population structure, effective population size and demographic history of the Finnish wolf population. Molecular Ecology, 15, 1561–1576. doi: 10.1111/j.1365-294X.2006.02877.x

34. Athrey, G., Barr, K. R., Lance, R. F., & Leberg, P. L. (2012). Birds in space and time: Genetic changes accompanying anthropogenic habitat fragmentation in the endangered black-capped vireo (Vireo atricapilla). Evolutionary Applications, 5, 540–552. doi: 10.1111/j.1752-4571.2011.00233.x

35. Athrey, G., Hodges, T. K., Reddy, M. R., Overgaard, H. J., Matias, A., Ridl, F. C., … Slotman, M. A. (2012). The Effective Population Size of Malaria Mosquitoes: Large Impact of Vector Control. PLoS Genetics, 8, e1003097. doi: 10.1371/journal.pgen.1003097

36. Augusteyn, J., Hughes, J., Armstrong, G., Real, K., & Pacioni, C. (2018). Tracking and tracing central Queensland's Macroderma -determining the size of the Mount Etna ghost bat population and potential threats. Australian Mammalogy, 40, 243–253. doi: 10.1071/AM16010

37. Aunins, A. W., Epifanio, J. M., & Brown, B. L. (2014). Genetic Evaluation of Supplementation-Assisted American Shad Restoration in the James River, Virginia. Marine and Coastal Fisheries, 6, 127–141. doi: 10.1080/19425120.2014.893465

38. Austin, J. D., Gore, J. A., Greene, D. U., & Gotteland, C. (2015). Conspicuous genetic structure belies recent dispersal in an endangered beach mouse (Peromyscus polionotus trissyllepsis). Conservation Genetics, 16, 915–928. doi: 10.1007/s10592-015-0710-8

39. Ayllon, F., Davaine, P., Beall, E., & Garcia-Vazquez, E. (2006). Dispersal and rapid evolution in brown trout colonizing virgin Subantarctic ecosystems. Journal of Evolutionary Biology, 19, 1352–1358. doi: 10.1111/j.1420-9101.2005.01075.x

40. Ayllon, F., Davaine, P., Beall, E., Martinez, J. L., & Garcia-Vazquez, E. (2004). Bottlenecks and genetic changes in Atlantic salmon (Salmo salar L.) stocks introduced in the Subantarctic Kerguelen Islands. Aquaculture, 237, 103–116. doi: 10.1016/j.aquaculture.2004.04.014

41. Ayllon, F., Moran, P., & Garcia-Vazquez, E. (2006). Maintenance of a small anadromous subpopulation of brown trout (Salmo trutta L.) by straying. Freshwater Biology, 51, 351–358. doi: 10.1111/j.1365-2427.2005.01486.x

42. Ayre, D. J., Ottewell, K. M., Krauss, S. L., & Whelan, R. J. (2009). Genetic structure of seedling cohorts following repeated wildfires in the fire-sensitive shrub persoonia mollis ssp. nectens. Journal of Ecology, 97, 752–760. doi: 10.1111/j.1365-2745.2009.01516.x

43. Baalsrud, H. T., Sæther, B. E., Hagen, I. J., Myhre, A. M., Ringsby, T. H., Pärn, H., & Jensen, H. (2014). Effects of population characteristics and structure on estimates of effective population size in a house sparrow metapopulation. Molecular Ecology, 23, 2653–2668. doi: 10.1111/mec.12770

44. Baerwald, M., Bien, V., Feyrer, F., & May, B. (2007). Genetic analysis reveals two distinct Sacramento splittail (Pogonichthys macrolepidotus) populations. Conservation Genetics, 8, 159–167. doi: 10.1007/s10592-006-9157-2

45. Baillie, S. M., Muir, A. M., Scribner, K., Bentzen, P., & Krueger, C. C. (2016). Loss of genetic diversity and reduction of genetic distance among lake trout Salvelinus namaycush ecomorphs, Lake Superior 1959 to 2013. Journal of Great Lakes Research, 42, 204–216. doi: 10.1016/j.jglr.2016.02.001

46. Baker, R. J., Dickins, B., Wickliffe, J. K., Khan, F. A. A., Gaschak, S., Makova, K. D., & Phillips, C. D. (2017). Elevated mitochondrial genome variation after 50 generations of radiation exposure in a wild rodent. Evolutionary Applications, 10, 784–791. doi: 10.1111/eva.12475

47. Baker, S. J., Anthonysamy, W. J. B., Davis, M. A., Dreslik, M. J., Douglas, M. R., Douglas, M. E., & Phillips, C. A. (2018). Temporal Patterns of Genetic Diversity in an Imperiled Population of the Eastern Massasauga Rattlesnake (Sistrurus catenatus). Copeia, 106, 414–420. doi: 10.1643/CG-17-682

48. Baltazar-Soares, M., Bracamonte, S. E., Bayer, T., Chain, F. J. J., Hanel, R., Harrod, C., & Eizaguirre, C. (2016). Evaluating the adaptive potential of the European eel: Is the immunogenetic status recovering? PeerJ, 4, e1868. doi: 10.7717/peerj.1868

49. Banhos, A., Hrbek, T., Sanaiotti, T. M., & Farias, I. P. (2016). Reduction of genetic diversity of the Harpy Eagle in Brazilian tropical forests. PLoS ONE, 11, e0148902. doi: 10.1371/journal.pone.0148902

50. Banks, S. C., Dubach, J., Viggers, K. L., & Lindenmayer, D. B. (2010). Adult survival and microsatellite diversity in possums: Effects of major histocompatibility complex-linked microsatellite diversity but not multilocus inbreeding estimators. Oecologia, 162, 359–370. doi: 10.1007/s00442-009-1464-0

51. Banks, S. C., Lorin, T., Shaw, R. E., McBurney, L., Blair, D., Blyton, M. D. J., … Lindenmayer, D. B. (2015). Fine-scale refuges can buffer demographic and genetic processes against short-term climatic variation and disturbance: A 22-year case study of an arboreal marsupial. Molecular Ecology, 24, 3831–3845. doi: 10.1111/mec.13279

52. Barcia, A. R., López, G. E., Hernández, D., & García-Machado, E. (2005). Temporal variation of the population structure and genetic diversity of Farfantepenaeus notialis assessed by allozyme loci. Molecular Ecology, 14, 2933–2942. doi: 10.1111/j.1365-294X.2005.02613.x

53. Barker, J. S. F., Frydenberg, J., Sarup, P., & Loeschcke, V. (2011). Altitudinal and seasonal variation in microsatellite allele frequencies of Drosophila buzzatii. Journal of Evolutionary Biology, 24, 430–439. doi: 10.1111/j.1420-9101.2010.02180.x

54. Barrantes, D., Macaya, G., Guarino, L., Baudoin, J. P., & Rocha, O. J. (2008). The impact of local extinction on genetic structure of wild populations of lima beans (Phaseolus lunatus) in the central valley of Costa Rica: Consequences for the conservation of plant genetic resources. Revista de Biologia Tropical, 56, 1023–1041. doi: 10.15517/rbt.v56i3.5690

55. Barrett, L. G., He, T., Lamont, B. B., & Krauss, S. L. (2005). Temporal patterns of genetic variation across a 9-year-old aerial seed bank of the shrub Banksia hookeriana (Proteaceae). Molecular Ecology, 14, 4169–4179. doi: 10.1111/j.1365-294X.2005.02726.x

56. Barrière, A., & Félix, M. A. (2007). Temporal dynamics and linkage disequilibrium in natural Caenorhabditis elegans populations. Genetics, 176, 999–1011. doi: 10.1534/genetics.106.067223

57. Bartáková, V., Bryja, J., & Reichard, M. (2018). Fine-scale genetic structure of the European bitterling at the intersection of three major European watersheds. BMC Evolutionary Biology, 18, 1–15. doi: 10.1186/s12862-018-1219-9

58. Basarab, J. A., Crowley, J. J., Abo-Ismail, M. K., Manafiazar, G. M., Akanno, E. C., Baron, V. S., & Plastow, G. (2018). Genomic retained heterosis effects on fertility and lifetime productivity in beef heifers. Canadian Journal of Animal Science, 98, 642–655. doi: 10.1139/cjas-2017-0192

59. Bastos, H. B., Gonçalves, E. C., Ferrari, S. F., Silva, A., & Schneider, M. P. C. (2010). Genetic structure of red-handed howler monkey populations in the fragmented landscape of eastern Brazilian Amazonia. Genetics and Molecular Biology, 33, 774–780. doi: 10.1590/S1415-47572010000400027

60. Bateson, Z. W., Dunn, P. O., Hull, S. D., Henschen, A. E., Johnson, J. A., & Whittingham, L. A. (2014). Genetic restoration of a threatened population of greater prairie-chickens. *Biological Conservation*, *174*, 12–19. doi: 10.1016/j.biocon.2014.03.008

61. Bech, N., Beltran, S., Portela, J., Rognon, A., Allienne, J. F., Boissier, J., & Théron, A. (2010). Follow-up of the genetic diversity and snail infectivity of a Schistosoma mansoni strain from field to laboratory. Infection, Genetics and Evolution, 10, 1039–1045. doi: 10.1016/j.meegid.2010.06.012

62. Begg, G. A., Keenan, C. P., & Sellin, M. J. (1998). Genetic variation and stock structure of school mackerel and spotted mackerel in northern Australian waters. Journal of Fish Biology, 53, 543–559. doi: 10.1006/jfbi.1998.9969

63. Bellemain, E., Nawaz, M. A., Valentini, A., Swenson, J. E., & Taberlet, P. (2007). Genetic tracking of the brown bear in northern Pakistan and implications for conservation. Biological Conservation, 134, 537–547. doi: 10.1016/j.biocon.2006.09.004

64. Benavente, J. N., Seeb, L. W., Seeb, J. E., Arismendi, I., Hernández, C. E., Gajardo, G., … Gomez-Uchida, D. (2015). Temporal genetic variance and propagule-driven genetic structure characterize naturalized rainbow trout (Oncorhynchus mykiss) from a patagonian lake impacted by trout farming. PLoS ONE, 10, e0142040. doi: 10.1371/journal.pone.0142040

65. Benzie, J. A. H., & Wakeford, M. (1997). Genetic structure of crown-of-thorns starfish (Acanthaster planci) on the Great Barrier Reef, Australia: comparison of two sets of outbreak populations occurring ten years apart. Marine Biology, 129, 149–157. doi: 10.1007/s002270050155

66. Bergner, L. M., Dussex, N., Jamieson, I. G., & Robertson, B. C. (2016). European colonization, not polynesian arrival, impacted population size and genetic diversity in the critically endangered New Zealand Kakapo. Heredity, 107, 593–602. doi: 10.1093/jhered/esw065

67. Berlin, A., Samils, B., Djurle, A., Wirsén, H., Szabo, L., & Yuen, J. (2013). Disease development and genotypic diversity of Puccinia graminis f. sp. avenae in Swedish oat fields. Plant Pathology, 62, 32–40. doi: 10.1111/j.1365-3059.2012.02609.x

68. Bernard, A. M., Feldheim, K. A., Nemeth, R., Kadison, E., Blondeau, J., Semmens, B. X., & Shivji, M. S. (2016). The ups and downs of coral reef fishes: the genetic characteristics of a formerly severely overfished but currently recovering Nassau grouper fish spawning aggregation. Coral Reefs, 35, 273–284. doi: 10.1007/s00338-015-1370-3

69. Bernardes, P. A., Grossi, D. A., Savegnago, R. P., Buzanskas, M. E., Ramos, S. B., Romanzini, E. P., … Munari, D. P. (2016). Population structure of Tabapuã beef cattle using pedigree analysis. *Livestock Science*, *187*, 96–101. doi: 10.1016/j.livsci.2016.03.002

70. Bernos, T. A., & Fraser, D. J. (2016). Spatiotemporal relationship between adult census size and genetic population size across a wide population size gradient. Molecular Ecology, 25, 4472–4487. doi: 10.1111/mec.13790

71. Berthier, K., Charbonnel, N., Galan, M., Chaval, Y., & Cosson, J. F. (2006). Migration and recovery of the genetic diversity during the increasing density phase in cyclic vole populations. Molecular Ecology, 15, 2665–2676. doi: 10.1111/j.1365-294X.2006.02959.x

72. Besnard, G., Dupuy, J., Larter, M., Cuneo, P., Cooke, D., & Chikhi, L. (2014). History of the invasive African olive tree in Australia and Hawaii: Evidence for sequential bottlenecks and hybridization with the Mediterranean olive. Evolutionary Applications, 7, 195–211. doi: 10.1111/eva.12110

73. Biedrzycka, A., & Kloch, A. (2016). Development of novel associations between MHC alleles and susceptibility to parasitic infections in an isolated population of an endangered mammal. *Infection, Genetics and Evolution*, *44*, 210–217. doi: 10.1016/j.meegid.2016.07.014

74. Biermann, A. D. M., Pimentel, E. C. G., Tietze, M., Pinent, T., & König, S. (2014). Implementation of genetic evaluation and mating designs for the endangered local pig breed “Bunte Bentheimer.” Journal of Animal Breeding and Genetics, 131, 36–45. doi: 10.1111/jbg.12041

75. Bingham, D. M., Kennedy, B. M., Hanson, K. C., & Smith, C. T. (2014). Loss of Genetic Integrity in Hatchery Steelhead Produced by Juvenile-Based Broodstock and Wild Integration: Conflicts in Production and Conservation Goals. North American Journal of Fisheries Management, 34, 609–620. doi: 10.1080/02755947.2014.901257

76. Binks, R. M., Kennington, W. J., & Johnson, M. S. (2007). Rapid evolutionary responses in a translocated population of intertidal snail (Bembicium vittatum) utilise variation from different source populations. Conservation Genetics, 8, 1421–1429. doi: 10.1007/s10592-007-9293-3

77. Bitocchi, E., Bellucci, E., Rau, D., Albertini, E., Rodriguez, M., Veronesi, F., … Nanni, L. (2015). European flint landraces grown in situ reveal adaptive introgression from modern maize. PLoS ONE, 10, e0121381. doi: 10.1371/journal.pone.0121381

78. Björklund, M., & Arrendal, J. (2008). Demo-genetic analysis of a recovering population of otters in Central Sweden. Animal Conservation, 11, 529–534. doi: 10.1111/j.1469-1795.2008.00214.x

79. Björklund, M., Aho, T., & Behrmann-Godel, J. (2015). Isolation over 35 years in a heated biotest basin causes selection on MHC class IIß genes in the European perch (Perca fluviatilis L.). Ecology and Evolution, 5, 1440–1455. doi: 10.1002/ece3.1426

80. Bjorndal, K. A., & Bolten, A. B. (2008). Annual variation in source contributions to a mixed stock: Implications for quantifying connectivity. Molecular Ecology, 17, 2185–2193. doi: 10.1111/j.1365-294X.2008.03752.x

81. Blanchet, S., Rey, O., Berthier, P., Lek, S., & Loot, G. (2009). Evidence of parasite-mediated disruptive selection on genetic diversity in a wild fish population. Molecular Ecology, 18, 1112–1123. doi: 10.1111/j.1365-294X.2009.04099.x

82. Blanchong, J. A., Scribner, K. T., Epperson, B. K., & Winterstein, S. R. (2006). Changes in Artificial Feeding Regulations Impact White-Tailed Deer Fine-Scale Spatial Genetic Structure. Journal of Wildlife Management, 70, 1037–1043. doi: 10.2193/0022-541x(2006)70[1037:ciafri]2.0.co;2

83. Blott, S. C., Williams, J. L., & Haley, C. S. (1998). Genetic variation within the Hereford breed of cattle. Animal Genetics, 29, 202–211. doi: 10.1046/j.1365-2052.1998.00326.x

84. Bolton, P. E., Rollins, L. A., Brazill-Boast, J., Maute, K. L., Legge, S., Austin, J. J., & Griffith, S. C. (2018). Genetic diversity through time and space: diversity and demographic history from natural history specimens and serially sampled contemporary populations of the threatened Gouldian finch (Erythrura gouldiae). Conservation Genetics, 19, 737–754. doi: 10.1007/s10592-018-1051-1

85. Born, C., Kjellberg, F., Chevallier, M. H., Vignes, H., Dikangadissi, J. T., Sanguié, J., … Hossaert-Mckey, M. (2008). Colonization processes and the maintenance of genetic diversity: Insights from a pioneer rainforest tree, Aucoumea klaineana. Proceedings of the Royal Society B, 275, 2171–2179. doi: 10.1098/rspb.2008.0446

86. Borrell, Y. J., Bernardo, D., Blanco, G., Vázquez, E., & Sánchez, J. A. (2008). Spatial and temporal variation of genetic diversity and estimation of effective population sizes in Atlantic salmon (Salmo salar, L.) populations from Asturias (Northern Spain) using microsatellites. Conservation Genetics, 9, 807–819. doi: 10.1007/s10592-007-9400-5

87. Bourgeois, L., & Beaman, L. (2017). Tracking the genetic stability of a honey bee (Hymenoptera: Apidae) breeding program with genetic markers. Journal of Economic Entomology, 110, 1419–1423. doi: 10.1093/jee/tox175

88. Bourke, B. P., Frantz, A. C., Lavers, C. P., Davison, A., Dawson, D. A., & Burke, T. A. (2010). Genetic signatures of population change in the British golden eagle (Aquila chrysaetos). Conservation Genetics, 11, 1837–1846. doi: 10.1007/s10592-010-0076-x

89. Bousset, L., Henry, P. Y., Sourrouille, P., & Jarne, P. (2004). Population biology of the invasive freshwater snail Physa acuta approached through genetic markers, ecological characterization and demography. Molecular Ecology, 13, 2023–2036. doi: 10.1111/j.1365-294X.2004.02200.x

90. Bouzat, J. L., Johnson, J. A., Toepfer, J. E., Simpson, S. A., Esker, T. L., & Westemeier, R. L. (2009). Beyond the beneficial effects of translocations as an effective tool for the genetic restoration of isolated populations. Conservation Genetics, 10, 191–201. doi: 10.1007/s10592-008-9547-8

91. Bouzat, J. L., Lewin, H. A., & Paige, K. N. (1998). The ghost of genetic diversity past: historical DNA analysis of the greater prairie chicken. The American Naturalist, 152, 1–6. doi: 10.1086/286145

92. Breed, M. F., Ottewell, K. M., Gardner, M. G., Marklund, M. H. K., Dormontt, E. E., & Lowe, A. J. (2015). Mating patterns and pollinator mobility are critical traits in forest fragmentation genetics. Heredity, 115, 108–114. doi: 10.1038/hdy.2013.48

93. Bristol, R. M., Tucker, R., Dawson, D. A., Horsburgh, G., Prys-Jones, R. P., Frantz, A. C., … Groombridge, J. J. (2013). Comparison of historical bottleneck effects and genetic consequences of re-introduction in a critically endangered island passerine. Molecular Ecology, 22, 4644–4662. doi: 10.1111/mec.12429

94. Brito, L. F., McEwan, J. C., Miller, S. P., Pickering, N. K., Bain, W. E., Dodds, K. G., … Clarke, S. M. (2017). Genetic diversity of a New Zealand multi-breed sheep population and composite breeds' history revealed by a high-density SNP chip. BMC Genetics, 18, 1–11. doi: 10.1186/s12863-017-0492-8

95. Britten, H. B., Fleishman, E., Austin, G. T., & Murphy, D. D. (2003). Genetically effective and adult population sizes in the Apache silverspot butterfly, Speyeria nokomis apacheana (Lepidoptera: Nymphalidae). Western North American Naturalist, 63, 229–235.

96. Bronnenhuber, J. E., Dufour, B. A., Higgs, D. M., & Heath, D. D. (2011). Dispersal strategies, secondary range expansion and invasion genetics of the nonindigenous round goby, Neogobius melanostomus, in Great Lakes tributaries. Molecular Ecology, 20, 1845–1859. doi: 10.1111/j.1365-294X.2011.05030.x

97. Browett, S., McHugo, G., Richardson, I. W., Magee, D. A., Park, S. D. E., Fahey, A. G., … MacHugh, D. E. (2018). Genomic characterisation of the indigenous Irish Kerry cattle breed. Frontiers in Genetics, 9, 1–17. doi: 10.3389/fgene.2018.00051

98. Brown, J. E., Obas, V., Morley, V., & Powell, J. R. (2013). Phylogeography and spatio-temporal genetic variation of aedes aegypti (Diptera: Culicidae) populations in the Florida keys. Journal of Medical Entomology, 50, 294–299. doi: 10.1603/ME12173

99. Browne, L., Ottewell, K., & Karubian, J. (2015). Short-term genetic consequences of habitat loss and fragmentation for the neotropical palm Oenocarpus bataua. Heredity, 115, 389–395. doi: 10.1038/hdy.2015.35

100. Brunet, J., Zalapa, J., & Guries, R. (2016). Conservation of genetic diversity in slippery elm (Ulmus rubra) in Wisconsin despite the devastating impact of Dutch elm disease. Conservation Genetics, 17, 1001–1010. doi: 10.1007/s10592-016-0838-1

101. Bryant, J. V, Gottelli, D., Zeng, X., Hong, X., Chan, B. P. L., Fellowes, J. R., … Turvey, S. T. (2016). Assessing current genetic status of the Hainan gibbon using historical and demographic baselines: implications for conservation management of species of extreme rarity. Molecular Ecology, 25, 3540–3556. doi: 10.1111/mec.13716

102. Brykov, V. A., Polyakova, N., Skurikhina, L. A., Kukhlevsky, A. D., Kirillova, O. N., Churikov, D., … Gharrett, A. J. (1999). Analysis of mtDNA indicates weak temporal genetic heterogeneity in pink salmon spawning runs in two rivers on Sakhalin Island. Journal of Fish Biology, 55, 617–635. doi: 10.1006/jfbi.1999.1025

103. Bunje, P. M. E., Barluenga, M., & Meyer, A. (2007). Sampling genetic diversity in the sympatrically and allopatrically speciating Midas cichlid species complex over a 16 year time series. *BMC Evolutionary Biology*, *7*, 1–14. doi: 10.1186/1471-2148-7-25

104. Buonaccorsi, V. P., Kimbrell, C. A., Lynn, E. A., & Hyde, J. R. (2012). Comparative population genetic analysis of bocaccio rockfish sebastes paucispinis using anonymous and gene-associated simple sequence repeat loci. Heredity, 103, 391–399. doi: 10.1093/jhered/ess002

105. Burchhardt, K. M., Miller, M. E., Cline, W. O., & Cubeta, M. A. (2017). Fine-scale genetic structure and reproductive biology of the blueberry pathogen Monilinia vaccinii-corymbosi. Phytopathology, 107, 231–239. doi: 10.1094/PHYTO-02-16-0093-R

106. Busch, J. D., Waser, P. M., & Dewoody, J. A. (2009). The influence of density and sex on patterns of fine-scale genetic structure. Evolution, 63, 2302–2314. doi: 10.1111/j.1558-5646.2009.00721.x

107. Byrne, R. J., Bernardi, G., & Avise, J. C. (2013). Spatiotemporal genetic structure in a protected marine fish, the California Grunion (Leuresthes tenuis), and relatedness in the genus leuresthes. Heredity, 104, 521–531. doi: 10.1093/jhered/est024

108. Calderón, I., & Turon, X. (2010). Temporal genetic variability in the Mediterranean common sea urchin Paracentrotus lividus. *Marine Ecology Progress Series*, *408*, 149–159. doi: 10.3354/meps08576

109. Calderón, I., PalacÍn, C., & Turon, X. (2009). Microsatellite markers reveal shallow genetic differentiation between cohorts of the common sea urchin Paracentrotus lividus (Lamarck) in northwest Mediterranean. Molecular Ecology, 18, 3036–3049. doi: 10.1111/j.1365-294X.2009.04239.x

110. Camara, M. D. (2011). Changes in molecular genetic variation at AFLP loci associated with naturalization and domestication of the Pacific oyster (Crassostrea gigas). Aquatic Living Resources, 24, 35–43. doi: 10.1051/alr/2011107

111. Cammen, K. M., Schultz, T. F., Bowen, W. D., Hammill, M. O., Puryear, W. B., Runstadler, J., … Kinnison, M. (2018). Genomic signatures of population bottleneck and recovery in Northwest Atlantic pinnipeds. Ecology and Evolution, 8, 6599–6614. doi: 10.1002/ece3.4143

112. Cammen, K. M., Vincze, S., Heller, A. S., McLeod, B. A., Wood, S. A., Bowen, W. D., … Frasier, T. R. (2018). Genetic diversity from pre-bottleneck to recovery in two sympatric pinniped species in the Northwest Atlantic. Conservation Genetics, 19, 555–569. doi: 10.1007/s10592-017-1032-9

113. Campagna, L., Van Coeverden de Groot, P. J., Saunders, B. L., Atkinson, S. N., Weber, D. S., Dyck, M. G., … Lougheed, S. C. (2013). Extensive sampling of polar bears (Ursus maritimus) in the Northwest Passage (Canadian Arctic Archipelago) reveals population differentiation across multiple spatial and temporal scales. Ecology and Evolution, 3, 3152–3165. doi: 10.1002/ece3.662

114. Cañas-Álvarez, J. J., Gónzalez-Rodríguez, A., Martín-Collado, D., Avilés, C., Altarriba, J., Baro, J. A., … Piedrafita, J. (2014). Monitoring changes in the demographic and genealogical structure of the main Spanish local beef breeds. Journal of Animal Science, 92, 4364–4374. doi: 10.2527/jas.2013-7420

115. Caplins, S. A., Gilbert, K. J., Ciotir, C., Roland, J., Matter, S. F., & Keyghobadi, N. (2014). Landscape structure and the genetic effects of a population collapse. Proceedings of the Royal Society B, 281, 2–10. doi: 10.1098/rspb.2014.1798

116. Cardoso, M. J., Eldridge, M. D. B., Oakwood, M., Rankmore, B., Sherwin, W. B., & Firestone, K. B. (2009). Effects of founder events on the genetic variation of translocated island populations: implications for conservation management of the northern quoll. *Conservation Genetics*, Vol. 10, pp. 1719–1733. doi: 10.1007/s10592-008-9774-z

117. Cardoso, P. C. B., Veiga, M. M., de Menezes, I. P. P., Valdisser, P. A. M. R., Borba, T. C. O., Melo, L. C., … Vianello-Brondani, R. P. (2013). Molecular characterization of high performance inbred lines of Brazilian common beans. Genetics and Molecular Research, 12, 5467–5484. doi: 10.4238/2013.February.6.4

118. Carlsson, J., & Nilsson, J. (2000). Population genetic structure of brown trout (Salmo trutta L.) within a northern boreal forest stream. Hereditas, 132, 173–181. doi: 10.1111/j.1601-5223.2000.00173.x

119. Carriconde, F., Gryta, H., Jargeat, P., Mouhamadou, B., & Gardes, M. (2008). High sexual reproduction and limited contemporary dispersal in the ectomycorrhizal fungus Tricholoma scalpturatum: New insights from population genetics and spatial autocorrelation analysis. Molecular Ecology, 17, 4433–4445. doi: 10.1111/j.1365-294X.2008.03924.x

120. Cartaxo, M. F. S., Ayres, C. F. J., & Weetman, D. (2011). Loss of genetic diversity in Culex quinquefasciatus targeted by a lymphatic filariasis vector control program in Recife, Brazil. Transactions of the Royal Society of Tropical Medicine and Hygiene, 105, 491–499. doi: 10.1016/j.trstmh.2011.05.004

121. Casas-Marce, M., Marmesat, E., Soriano, L., Martínez-Cruz, B., Lucena-Perez, M., Nocete, F., … Godoy, J. A. (2017). Spatiotemporal dynamics of genetic variation in the iberian lynx along its path to extinction reconstructed with ancient DNA. Molecular Biology and Evolution, 34, 2893–2907. doi: 10.1093/molbev/msx222

122. Casas-Marce, M., Soriano, L., Lõpez-Bao, J. V, & Godoy, J. A. (2013). Genetics at the verge of extinction: Insights from the Iberian lynx. Molecular Ecology, 22, 5503–5515. doi: 10.1111/mec.12498

123. Casey, C. S., Orozco-terWengel, P., Yaya, K., Kadwell, M., Fernández, M., Marín, J. C., … Bruford, M. W. (2018). Comparing genetic diversity and demographic history in co-distributed wild South American camelids. Heredity, 121, 387–400. doi: 10.1038/s41437-018-0120-z

124. Cervantes, I., Goyache, F., Molina, A., Valera, M., & Gutiérrez, J. P. (2008). Application of individual increase in inbreeding to estimate realized effective sizes from real pedigrees. Journal of Animal Breeding and Genetics, 125, 301–310. doi: 10.1111/j.1439-0388.2008.00755.x

125. Chabarria, R., Furiness, S., Patterson, L., Hall, J., Chen, Y., Lynch, B., & Pezold, F. (2014). Genetic structure and demographic history of endemic micronesian blue riffle goby, stiphodon caeruleus (Gobiidae) inferred from mitochondrial DNA sequence analysis. Copeia, 1, 23–37. doi: 10.1643/CI-12-150

126. Chacón-Sánchez, M. I., & Martínez-Castillo, J. (2017). Testing domestication scenarios of Lima bean (Phaseolus lunatus L.) in mesoamerica: Insights from genome-wide genetic markers. Frontiers in Plant Science, 8, 1–20. doi: 10.3389/fpls.2017.01551

127. Chang, K., Han, Y., & Tzeng, W. (2007). Population genetic structure among intra-annual arrival waves of the Japanese Eel Anguilla japonica in Northern Taiwan. Zoological Studies, 46, 583–590.

128. Charbonnel, N., & Pemberton, J. (2005). A long-term genetic survey of an ungulate population reveals balancing selection acting on MHC through spatial and temporal fluctuations in selection. Heredity, 95, 377–388. doi: 10.1038/sj.hdy.6800735

129. Charbonnel, N., Quesnoit, M., Razatavonjizay, R., Brémond, P., & Jarne, P. (2002). A spatial and temporal approach to microevolutionary forces affecting population biology in the freshwater snail Biomphalaria pfeifferi. The American Naturalist, 160, 741–755. doi: 10.1086/343875

130. Charles, K., Roussel, J. M., Lebel, J. M., Baglinière, J. L., & Ombredane, D. (2006). Genetic differentiation between anadromous and freshwater resident brown trout (Salmo trutta L.): Insights obtained from stable isotope analysis. Ecology of Freshwater Fish, 15, 255–263. doi: 10.1111/j.1600-0633.2006.00149.x

131. Charlier, J., Laikre, L., & Ryman, N. (2012). Genetic monitoring reveals temporal stability over 30 years in a small, lake-resident brown trout population. Heredity, 109, 246–253. doi: 10.1038/hdy.2012.36

132. Chen, M., Fontaine, M. C., Ben Chehida, Y., Zheng, J., Labbé, F., Mei, Z., … Wang, D. (2017). Genetic footprint of population fragmentation and contemporary collapse in a freshwater cetacean. Scientific Reports, 7, 1–12. doi: 10.1038/s41598-017-14812-z

133. Chen, S. Y., Zhang, Y. J., Wang, X. L., Sun, J. Y., Xue, Y., Zhang, P., … Qu, L. H. (2012). Extremely low genetic diversity indicating the endangered status of Ranodon sibiricus (amphibia: Caudata) and implications for phylogeography. PLoS ONE, 7, e33378. doi: 10.1371/journal.pone.0033378

134. Chen, Y. H., Berlocher, S. H., Opp, S. B., & Roderick, G. K. (2010). Post-colonization temporal genetic variation of an introduced fly, Rhagoletis completa. Genetica, 138, 1059–1075. doi: 10.1007/s10709-010-9491-7

135. Cheng, X. Y., Cheng, F. X., Xu, R. M., & Xie, B. Y. (2008). Genetic variation in the invasive process of Bursaphelenchus xylophilus (Aphelenchida: Aphelenchoididae) and its possible spread routes in China. Heredity, 100, 356–365. doi: 10.1038/sj.hdy.6801082

136. Chevolot, M., Ellis, J. R., Rijnsdorp, A. D., Stam, W. T., & Olsen, J. L. (2008). Temporal changes in allele frequencies but stable genetic diversity over the past 40 years in the Irish Sea population of thornback ray, Raja clavata. Heredity, 101, 120–126. doi: 10.1038/hdy.2008.36

137. Chevolot, M., Wolfs, P. H. J., Pálsson, J., Rijnsdorp, A. D., Stam, W. T., & Olsen, J. L. (2007). Population structure and historical demography of the thorny skate (Amblyraja radiata, Rajidae) in the North Atlantic. Marine Biology, 151, 1275–1286. doi: 10.1007/s00227-006-0556-1

138. Chiappero, M. B., Sommaro, L. V, Priotto, J. W., Wiernes, M. P., Steinmann, A. R., & Gardenal, C. N. (2016). Spatio-temporal genetic structure of the rodent Calomys venustus in linear, fragmented habitats. Journal of Mammalogy, 97, 424–435. doi: 10.1093/jmammal/gyv186

139. Christensen, C., Jacobsen, M. W., Nygaard, R., & Hansen, M. M. (2018). Spatiotemporal genetic structure of anadromous Arctic char (Salvelinus alpinus) populations in a region experiencing pronounced climate change. Conservation Genetics, 19, 687–700. doi: 10.1007/s10592-018-1047-x

140. Christie, M. R., Marine, M. L., French, R. A., Waples, R. S., & Blouin, M. S. (2012). Effective size of a wild salmonid population is greatly reduced by hatchery supplementation. Heredity, 109, 254–260. doi: 10.1038/hdy.2012.39

141. Chung, M. G., Chung, M. Y., Oh, G. S., & Epperson, B. K. (2000). Spatial genetic structure in a Neolitsea sericea population (Lauraceae). Heredity, 85, 490–497. doi: 10.1046/j.1365-2540.2000.00781.x

142. Chung, M. Y., Epperson, B. K., & Chung, M. G. (2003). Genetic structure of age classes in Camellia japonica (Theaceae). Evolution, 57, 62–73. doi: 10.1111/j.0014-3820.2003.tb00216.x

143. Chung, P. P., Chu, I., & Ballard, J. W. O. (2014). Assessment of temporal genetic variability of two epibenthic amphipod species in an eastern Australian estuarine environment and their suitability as biological monitors. Australian Journal of Zoology, 62, 206–215. doi: 10.1071/ZO13104

144. Ciancaleoni, S., Raggi, L., & Negri, V. (2018). Assessment of spatial–temporal variation in natural populations of Brassica incana in south Italy: implications for conservation. Plant Systematics and Evolution, 304, 731–745. doi: 10.1007/s00606-018-1505-4

145. Ciborowski, K. L., Consuegra, S., García De Leániz, C., Wang, J., Beaumont, M. A., & Jordan, W. C. (2007). Stocking may increase mitochondrial DNA diversity but fails to halt the decline of endangered Atlantic salmon populations. Conservation Genetics, 8, 1355–1367. doi: 10.1007/s10592-007-9286-2

146. Ciborowski, K., Jordan, W. C., Garcia De Leaniz, C., & Consuegra, S. (2017). Temporal and spatial instability in neutral and adaptive (MHC) genetic variation in marginal salmon populations. Scientific Reports, 7, 1–11. doi: 10.1038/srep42416

147. Clifford, S. L., McGinnity, P., & Ferguson, A. (1998). Genetic changes in Atlantic salmon (Salmo salar) populations of Northwest Irish rivers resulting from escapes of adult farm salmon. Canadian Journal of Botany, 55, 358–363. doi: 10.1006/jfbi.1997.0566

148. Coates, D. J. (1992). Genetic consequences of a bottleneck and spatial genetic structure in the triggerplant stylidium coroniforme (Stylidiaceae). Heredity, 69, 512–520. doi: 10.1038/hdy.1992.166

149. Cobble, K. R., Califf, K. J., Stone, N. E., Shuey, M. M., Birdsell, D. N., Colman, R. E., … Busch, J. D. (2016). Genetic variation at the MHC DRB1 locus is similar across Gunnison's prairie dog (Cynomys gunnisoni) colonies regardless of plague history. Ecology and Evolution, 6, 2624–2651. doi: 10.1002/ece3.2077

150. Cole, T. L., Hammer, M. P., Unmack, P. J., Teske, P. R., Brauer, C. J., Adams, M., & Beheregaray, L. B. (2016). Range-wide fragmentation in a threatened fish associated with post-European settlement modification in the Murray–Darling Basin, Australia. Conservation Genetics, 17, 1377–1391. doi: 10.1007/s10592-016-0868-8

151. Collins, C. J., Chilvers, B. L., Taylor, M., & Robertson, B. C. (2016). Historical population size of the threatened New Zealand sea lion Phocarctos hookeri. Journal of Mammalogy, 97, 436–443. doi: 10.1093/jmammal/gyv187

152. Colominas, L., Edward, C. J., Beja-pereira, A., Vigne, J., Silva, R. M., Castanyer, P., … Bower, M. A. (2015). Detecting the T1 cattle haplogroup in the Iberian Peninsula from Neolithic to medieval times: new clues to continuous cattle migration through time. *Journal of Archaeological Science*, *59*, 110–117. doi: https://doi.org/10.1016/j.jas.2015.04.014

153. Consuegra, S., De Eyto, E., McGinnity, P., Stet, R. J. M., & Jordan, W. C. (2011). Contrasting responses to selection in class i and class IIα major histocompatibility-linked markers in salmon. Heredity, 107, 143–154. doi: 10.1038/hdy.2010.177

154. Cooper, A. M., Miller, L. M., & Kapuscinski, A. R. (2010). Conservation of population structure and genetic diversity under captive breeding of remnant coaster brook trout (Salvelinus fontinalis) populations. Conservation Genetics, 11, 1087–1093. doi: 10.1007/s10592-009-9841-0

155. Costa, M., Fernandes, C., Birks, J. D. S., Kitchener, A. C., Santos-Reis, M., & Bruford, M. W. (2013). The genetic legacy of the 19th-century decline of the British polecat: Evidence for extensive introgression from feral ferrets. Molecular Ecology, 22, 5130–5147. doi: 10.1111/mec.12456

156. Costedoat, C., Pech, N., Salducci, M. D., Chappaz, R., & Gilles, A. (2005). Evolution of mosaic hybrid zone between invasive and endemic species of Cyprinidae through space and time. Biological Journal of the Linnean Society, 85, 135–155. doi: 10.1111/j.1095-8312.2005.00478.x

157. Côté, C. L., Gagnaire, P. A., Bourret, V., Verreault, G., Castonguay, M., & Bernatchez, L. (2013). Population genetics of the American eel (Anguilla rostrata): FST = 0 and North Atlantic Oscillation effects on demographic fluctuations of a panmictic species. Molecular Ecology, 22, 1763–1776. doi: 10.1111/mec.12142

158. Cousseau, L., Husemann, M., Foppen, R., Vangestel, C., & Lens, L. (2016). A longitudinal genetic survey identifies temporal shifts in the population structure of Dutch house sparrows. Heredity, 117, 259–267. doi: 10.1038/hdy.2016.38

159. Couvray, S., Miard, T., Bunet, R., Martin, Y., Grillasca, J. P., Bonnefont, J. L., & Coupé, S. (2015). Experimental release of juvenile sea urchins (Paracentrotus lividus) in exploited sites along the French mediterranean coast. Journal of Shellfish Research, 34, 555–563. doi: 10.2983/035.034.0240

160. Cowled, B. D., Lapidge, S. J., Hampton, J. O., & Spencer, P. B. S. (2006). Measuring the Demographic and Genetic Effects of Pest Control in a Highly Persecuted Feral Pig Population. Journal of Wildlife Management, 70, 1690–1697. doi: 10.2193/0022-541x(2006)70[1690:mtdage]2.0.co;2

161. Cozzi, M. C., Colombo, E., Zaniboni, L., Madeddu, M., Mosca, F., Strillacci, M. G., … Cerolini, S. (2017). Phenotypic and genetic characterization of the Italian bantam chicken breed Mericanel della Brianza. Livestock Science, 205, 56–63. doi: 10.1016/j.livsci.2017.09.013

162. Craul, M., Chikhi, L., Sousa, V., Olivieri, G. L., Rabesandratana, A., Zimmermann, E., & Radespiel, U. (2009). Influence of forest fragmentation on an endangered large-bodied lemur in northwestern Madagascar. Biological Conservation, 142, 2862–2871. doi: 10.1016/j.biocon.2009.05.026

163. Crispo, E., & Chapman, L. J. (2010). Temporal variation in population genetic structure of a riverine African cichlid fish. Heredity, 101, 97–106. doi: 10.1093/jhered/esp078

164. Cross, M. A., Collins, C., Campbell, N., Watts, P. C., Chubb, J. C., Cunningham, C. O., … MacKenzie, K. (2007). Levels of intra-host and temporal sequence variation in a large CO1 sub-units from Anisakis simplex sensu stricto (Rudolphi 1809) (Nematoda: Anisakisdae): implications for fisheries management. Marine Biology, 151, 695–702. doi: 10.1007/s00227-006-0509-8

165. Crozier, W. W. (2000). Escaped farmed salmon, Salmo salar L., in the Glenarm River, Northern Ireland: genetic status of the wild population 7 years on. Fisheries Management and Ecology, 7, 437–446. doi: 10.1046/j.1365-2400.2000.00219.x

166. Cubry, P., Gallagher, E., O'Connor, E., & Kelleher, C. T. (2015). Phylogeography and population genetics of black alder (Alnus glutinosa (L.) Gaertn.) in Ireland: putting it in a European context. Tree Genetics and Genomes, 11, 1–15. doi: 10.1007/s11295-015-0924-4

167. Cui, D., Li, J., Tang, C., A, X., Yu, T., Ma, X., … Han, L. (2016). Diachronic analysis of genetic diversity in rice landraces under on-farm conservation in Yunnan, China. Theoretical and Applied Genetics, 129, 155–168. doi: 10.1007/s00122-015-2617-7

168. Cullingham, C. I., & Moehrenschlager, A. (2013). Temporal Analysis of Genetic Structure to Assess Population Dynamics of Reintroduced Swift Foxes. Conservation Biology, 27, 1389–1398. doi: 10.1111/cobi.12122

169. Cuveliers, E. L., Larmuseau, M. H. D., Hellemans, B., Verherstraeten, S. L. N. A., Volckaert, F. A. M., & Maes, G. E. (2012). Multi-marker estimate of genetic connectivity of sole (Solea solea) in the North-East Atlantic Ocean. Marine Biology, 159, 1239–1253. doi: 10.1007/s00227-012-1905-x

170. Cuveliers, E. L., Volckaert, F. A. M., Rijnsdorp, A. D., Larmuseau, M. H. D., & Maes, G. E. (2011). Temporal genetic stability and high effective population size despite fisheries-induced life-history trait evolution in the North Sea sole. Molecular Ecology, 20, 3555–3568. doi: 10.1111/j.1365-294X.2011.05196.x

171. D'Aloia, C. C., Azodi, C. B., Sheldon, S. P., Trombulak, S. C., & Ardren, W. R. (2015). Genetic models reveal historical patterns of sea lamprey population fluctuations within Lake Champlain. PeerJ, 3, e1369. doi: 10.7717/peerj.1369

172. Da Costa-Ribeiro, M. C. V, Lourenço-de-Oliveira, R., & Failloux, A. B. (2006). Geographic and temporal genetic patterns of Aedes aegypti populations in Rio de Janeiro, Brazil. Tropical Medicine and International Health, 11, 1276–1285. doi: 10.1111/j.1365-3156.2006.01667.x

173. da Silva, J. M., & Tolley, K. A. (2018). Conservation genetics of an endemic and threatened amphibian (Capensibufo rosei): a leap towards establishing a genetic monitoring framework. Conservation Genetics, 19, 349–363. doi: 10.1007/s10592-017-1008-9

174. Danchin-Burge, C., Leroy, G., Brochard, M., Moureaux, S., & Verrier, E. (2012). Evolution of the genetic variability of eight French dairy cattle breeds assessed by pedigree analysis. Journal of Animal Breeding and Genetics, 129, 206–217. doi: 10.1111/j.1439-0388.2011.00967.x

175. Darling, J. A., Tsai, Y. H. E., Blakeslee, A. M. H., & Roman, J. (2014). Are genes faster than crabs? Mitochondrial introgression exceeds larval dispersal during population expansion of the invasive crab Carcinus maenas. Royal Society Open Science, 1, 1–11. doi: 10.1098/rsos.140202

176. David, P., Perdieu, M.-A., Pernot, A.-F., & Jarne, P. (1997). Fine-grained spatial and temporal population genetic structure in the marine bivalve Spisula ovalis. Evolution, 51, 1318–1322. doi: 10.1111/j.1558-5646.1997.tb03979.x

177. Dawson, M. N., Barber, P. H., González-Guzmán, L. I., Toonen, R. J., Dugan, J. E., & Grosberg, R. K. (2011). Phylogeography of Emerita analoga (Crustacea, Decapoda, Hippidae), an eastern Pacific Ocean sand crab with long-lived pelagic larvae. Journal of Biogeography, 38, 1600–1612. doi: 10.1111/j.1365-2699.2011.02499.x

178. De Barba, M., Waits, L. P., Garton, E. O., Genovesi, P., Randi, E., Mustoni, A., & Groff, C. (2010). The power of genetic monitoring for studying demography, ecology and genetics of a reintroduced brown bear population. Molecular Ecology, 19, 3938–3951. doi: 10.1111/j.1365-294X.2010.04791.x

179. De Sousa, G. B., Jiménez, A., Blanco, A., & Gardenal, C. N. (1997). Temporal variation of allozyme frequencies in Aedes albifasciatus (Diptera: Culicidae) from Argentina. Biochemical Genetics, 35, 339–349. doi: 10.1023/A:1022225531822

180. De Wolf, H., Backeljau, T., & Verhagen, R. (1998). Spatio-temporal genetic structure and gene flow between two distinct shell morphs of the planktonic developing periwinkle Littorina striata (Mollusca: Prosobranchia). *Marine Ecology Progress Series, 163,* 155–163. doi: 10.3354/meps163155

181. Debinski, D. M. (1994). Genetic diversity assessment in a metapopulation of the butterfly Euphydryas gillettii. Biological Conservation, 70, 25–31. doi: 10.1016/0006-3207(94)90295-X

182. Dedryver, C. A., Le Gallic, J. F., Haack, L., Halkett, F., Outreman, Y., & Simon, J. C. (2008). Seasonal and annual genotypic variation and the effect of climate on population genetic structure of the cereal aphid Sitobion avenae in northern France. Bulletin of Entomological Research, 98, 159–168. doi: 10.1017/S0007485307005500

183. DeFaveri, J., & Merilä, J. (2015). Temporal stability of genetic variability and differentiation in the three-spined stickleback (Gasterosteus aculeatus). PLoS ONE, 10, e0123891. doi: 10.1371/journal.pone.0123891

184. Delaney, D. A., Meixner, M. D., Schiff, N. M., & Sheppard, W. S. (2009). Genetic characterization of commercial honey bee (Hymenoptera: Apidae) populations in the United States by using mitochondrial and microsatellite markers. Annals of the Entomological Society of America, 102, 666–673. doi: 10.1603/008.102.0411

185. Deu, M., Sagnard, F., Chantereau, J., Calatayud, C., Vigouroux, Y., Pham, J. L., … Bezançon, G. (2010). Spatio-temporal dynamics of genetic diversity in Sorghum bicolor in Niger. Theoretical and Applied Genetics, 120, 1301–1313. doi: 10.1007/s00122-009-1257-1

186. Diaz, M., Wethey, D., Bulak, J., & Ely, B. (2000). Effect of Harvest and Effective Population Size on Genetic Diversity in a Striped Bass Population. Transactions of the American Fisheries Society, 129, 1367–1372. doi: 10.1577/1548-8659(2000)129<1367:eohaep>2.0.co;2

187. Dibattista, J. D., Feldheim, K. A., Garant, D., Gruber, S. H., & Hendry, A. P. (2011). Anthropogenic disturbance and evolutionary parameters: A lemon shark population experiencing habitat loss. Evolutionary Applications, 4, 1–17. doi: 10.1111/j.1752-4571.2010.00125.x

188. Dionne, M., Miller, K. M., Dodson, J. J., & Bernatchez, L. (2009). MHC standing genetic variation and pathogen resistance in wild Atlantic salmon. Philosophical Transactions of the Royal Society B, 364, 1555–1565. doi: 10.1098/rstb.2009.0011

189. Dixon, T. J., Coman, G. J., Arnold, S. J., Sellars, M. J., Lyons, R. E., Dierens, L., … Li, Y. (2008). Shifts in genetic diversity during domestication of Black Tiger shrimp, Penaeus monodon, monitored using two multiplexed microsatellite systems. Aquaculture, 283, 1–6. doi: 10.1016/j.aquaculture.2008.07.009

190. Domingues, F. A., Silva-Brandão, K. L., Abreu, A. G., Perera, O. P., Blanco, C. A., Cônsoli, F. L., & Omoto, C. (2012). Genetic structure and gene flow among Brazilian populations of heliothis virescens (Lepidoptera: Noctuidae). Journal of Economic Entomology, 105, 2136–2146. doi: 10.1603/EC12123

191. Dornier, A., & Cheptou, P. O. (2012). Determinants of extinction in fragmented plant populations: Crepis sancta (asteraceae) in urban environments. Oecologia, 169, 703–712. doi: 10.1007/s00442-011-2229-0

192. Dowling, T. E., Marsh, P. C., Kelsen, A. T., & Tibbets, C. A. (2005). Genetic monitoring of wild and repatriated populations of endangered razorback sucker (Xyrauchen texanus, Catostomidae, Teleostei) in Lake Mohave, Arizona-Nevada. Molecular Ecology, 14, 123–135. doi: 10.1111/j.1365-294X.2004.02408.x

193. Dowling, T. E., Saltzgiver, M. J., Adams, D., & Marsh, P. C. (2012). Genetic variability in a recruiting population of endangered razorback suckers from Lake Mead, Arizona-Nevada. Transactions of the American Fisheries Society, 141, 990–999. doi: 10.1080/00028487.2012.679018

194. Dowling, T. E., Turner, T. F., Carson, E. W., Saltzgiver, M. J., Adams, D., Kesner, B., & Marsh, P. C. (2014). Time-series analysis reveals genetic responses to intensive management of razorback sucker (Xyrauchen texanus). Evolutionary Applications, 7, 339–354. doi: 10.1111/eva.12125

195. Draheim, H. M., Baird, P., & Haig, S. M. (2012). Temporal analysis of mtDNA variation reveals decreased genetic diversity in least terns. The Condor, 114, 145–154. doi: 10.1525/cond.2012.110007

196. Draheim, H. M., Moore, J. A., Fortin, M. J., & Scribner, K. T. (2018). Beyond the snapshot: Landscape genetic analysis of time series data reveal responses of American black bears to landscape change. Evolutionary Applications, 11, 1219–1230. doi: 10.1111/eva.12617

197. Drenth, A., Tas, I. C. Q., & Govers, F. (1994). DNA fingerprinting uncovers a new sexually reproducing population of Phytophthora infestans in the Netherlands. *European Journal of Plant Pathology, 100,* 97–107. doi: 10.1007/978-3-662-44056-8_9

198. Drobik, W., & Martyniuk, E. (2016). Inbreeding and its impact on the prolific Polish Olkuska sheep population. *Small Ruminant Research*, *137*, 28–33. doi: 10.1016/j.smallrumres.2016.02.009

199. Du, Y., Zou, X., Xu, Y., Guo, X., Li, S., Zhang, X., … Guo, S. (2016). Microsatellite loci analysis reveals post-bottleneck recovery of genetic diversity in the tibetan antelope. Scientific Reports, 6, 1–7. doi: 10.1038/srep35501

200. Dubois, A., Galan, M., Cosson, J. F., Gauffre, B., Henttonen, H., Niemimaa, J., … Charbonnel, N. (2017). Microevolution of bank voles (Myodes glareolus) at neutral and immune-related genes during multiannual dynamic cycles: Consequences for Puumala hantavirus epidemiology. *Infection, Genetics and Evolution*, *49*, 318–329. doi: 10.1016/j.meegid.2016.12.007

201. Duong, T. Y., Scribner, K. T., Forsythe, P. S., Crossman, J. A., & Baker, E. A. (2013). Interannual variation in effective number of breeders and estimation of effective population size in long-lived iteroparous lake sturgeon (Acipenser fulvescens). Molecular Ecology, 22, 1282–1294. doi: 10.1111/mec.12167

202. Dussex, N., Rawlence, N. J., & Robertson, B. C. (2015). Ancient and contemporary DNA reveal a pre-human decline but no population bottleneck associated with recent human persecution in the kea (Nestor notabilis). PLoS ONE, 10, e0118522. doi: 10.1371/journal.pone.0118522

203. Dussex, N., Taylor, H. R., Stovall, W. R., Rutherford, K., Dodds, K. G., Clarke, S. M., & Gemmell, N. J. (2018). Reduced representation sequencing detects only subtle regional structure in a heavily exploited and rapidly recolonizing marine mammal species. Ecology and Evolution, 8, 8736–8749. doi: 10.1002/ece3.4411

204. Dussex, N., von Seth, J., Robertson, B. C., & Dalén, L. (2018). Full mitogenomes in the critically endangered kākāpō reveal major post-glacial and anthropogenic effects on neutral genetic diversity. Genes, 9, 1–14. doi: 10.3390/genes9040220

205. E, G. X., Huang, Y. F., Zhao, Y. J., He, J. N., Liu, N., Zhong, T., … Chen, L. P. (2015). Dynamic comparison of genetic diversity in a Small Tail Han sheep population using meta-analysis. Genetics and Molecular Research, 14, 14607–14614. doi: 10.4238/2015.November.18.24

206. Echodu, R., Beadell, J. S., Okedi, L. M., Hyseni, C., Aksoy, S., & Caccone, A. (2011). Temporal stability of Glossina fuscipes fuscipes populations in Uganda. Parasites and Vectors, 4, 1–10. doi: 10.1186/1756-3305-4-19

207. Eda, M., Koike, H., Kuro-o, M., Mihara, S., Hasegawa, H., & Higuchi, H. (2012). Inferring the ancient population structure of the vulnerable albatross Phoebastria albatrus, combining ancient DNA, stable isotope, and morphometric analyses of archaeological samples. Conservation Genetics, 13, 143–151. doi: 10.1007/s10592-011-0270-5

208. Edillo, F., Kiszewski, A., Manjourides, J., Pagano, M., Hutchinson, M., Kyle, A., … Boisvert, M. (2009). Effects of latitude and longitude on the population structure of Culex pipiens s.l., vectors of West Nile virus in North America. American Journal of Tropical Medicine and Hygiene, 81, 842–848. doi: 10.4269/ajtmh.2009.08-0605

209. Eimes, J. A., Bollmer, J. L., Whittingham, L. A., Johnson, J. A., Van Oosterhout, C., & Dunn, P. O. (2011). Rapid loss of MHC class II variation in a bottlenecked population is explained by drift and loss of copy number variation. Journal of Evolutionary Biology, 24, 1847–1856. doi: 10.1111/j.1420-9101.2011.02311.x

210. Eldridge, M. D. B., Rummery, C., Bray, C., Zenger, K. R., Browning, T. L., & Close, R. L. (2004). Genetic analysis of a population crash in brush-tailed rock-wallabies (Petrogale penicillata) from Jenolan Caves , south-eastern Australia. *Wildlife Research*, *31*, 229–240. doi: 10.1071/WR03030

211. Eldridge, W. H., & Killebrew, K. (2008). Genetic diversity over multiple generations of supplementation: An example from Chinook salmon using microsatellite and demographic data. Conservation Genetics, 9, 13–28. doi: 10.1007/s10592-007-9298-y

212. Eldridge, W. H., Myers, J. M., & Naish, K. A. (2009). Long-term changes in the fine-scale population structure of coho salmon populations (Oncorhynchus kisutch) subject to extensive supportive breeding. Heredity, 103, 299–309. doi: 10.1038/hdy.2009.69

213. Ellis, J. S., Sumner, K. J., Griffiths, A. M., Bright, D. I., & Stevens, J. R. (2011). Population genetic structure of Atlantic salmon, Salmo salar L., in the River Tamar, southwest England. Fisheries Management and Ecology, 18, 233–245. doi: 10.1111/j.1365-2400.2010.00776.x

214. Epps, C. W., Crowhurst, R. S., & Nickerson, B. S. (2018). Assessing changes in functional connectivity in a desert bighorn sheep metapopulation after two generations. Molecular Ecology, 27, 2334–2346. doi: 10.1111/mec.14586

215. Evanno, G., Castella, E., Antoine, C., Paillat, G., & Goudet, J. (2009). Parallel changes in genetic diversity and species diversity following a natural disturbance. Molecular Ecology, 18, 1137–1144. doi: 10.1111/j.1365-294X.2009.04102.x

216. Fabbri, E., Caniglia, R., Galov, A., Arbanasić, H., Lapini, L., Bošković, I., … Randi, E. (2014). Genetic structure and expansion of golden jackals (Canis aureus) in the north-western distribution range (Croatia and eastern Italian Alps). Conservation Genetics, 15, 187–199. doi: 10.1007/s10592-013-0530-7

217. Fabiani, A., Gratton, P., Zappes, I. A., Seminara, M., D'Orsi, A., Sbordoni, V., & Allegrucci, G. (2018). Investigating the genetic structure of trout from the Garden of Ninfa (central Italy): Suggestions for conservation and management. Fisheries Management and Ecology, 25, 1–11. doi: 10.1111/fme.12259

218. Facon, B., Hufbauer, R. A., Tayeh, A., Loiseau, A., Lombaert, E., Vitalis, R., … Estoup, A. (2011). Inbreeding depression is purged in the invasive insect harmonia axyridis. Current Biology, 21, 424–427. doi: 10.1016/j.cub.2011.01.068

219. Farias, I. P., Santos, W. G., Gordo, M., & Hrbek, T. (2015). Effects of Forest Fragmentation on Genetic Diversity of the Critically Endangered Primate, the Pied Tamarin (Saguinus bicolor): Implications for Conservation. Heredity, 106, 512–521. doi: 10.1093/jhered/esv048

220. Farrington, H. L., & Petren, K. (2011). A century of genetic change and metapopulation dynamics in the galápagos warbler finches (certhidea). Evolution, 65, 3148–3161. doi: 10.1111/j.1558-5646.2011.01385.x

221. Farrington, L. W., Lintermans, M., & Ebner, B. C. (2014). Characterising genetic diversity and effective population size in one reservoir and two riverine populations of the threatened Macquarie perch. Conservation Genetics, 15, 707–716. doi: 10.1007/s10592-014-0572-5

222. Fauvelot, C., Cleary, D. F. R., & Menken, S. B. J. (2006). Short-term impact of 1997/1998 ENSO-induced disturbance on abundance and genetic variation in a tropical butterfly. Heredity, 97, 367–380. doi: 10.1093/jhered/esl010

223. Feng, X., Johnson, E. G., Williams, E. P., & Place, A. R. (2017). Successful Identification and Discrimination of Hatchery-Reared Blue Crabs (Callinectes sapidus) Released into the Chesapeake Bay Using a Genetic Tag. Journal of Shellfish Research, 36, 277–282. doi: 10.2983/035.036.0131

224. Fennell, M., Gallagher, T., Vintro, L. L., & Osborne, B. (2014). Using soil seed banks to assess temporal patterns of genetic variation in invasive plant populations. Ecology and Evolution, 4, 1648–1658. doi: 10.1002/ece3.1043

225. Ferchaud, A. L., Perrier, C., April, J., Hernandez, C., Dionne, M., & Bernatchez, L. (2016). Making sense of the relationships between Ne, Nb and Nc towards defining conservation thresholds in Atlantic salmon (Salmo salar). Heredity, 117, 268–278. doi: 10.1038/hdy.2016.62

226. Fernández-De-Mera, I. G., Vicente, J., Pérez De La Lastra, J. M., Mangold, A. J., Naranjo, V., Fierro, Y., … Gortázar, C. (2009). Reduced major histocompatibility complex class II polymorphism in a hunter-managed isolated Iberian red deer population. Journal of Zoology, 277, 157–170. doi: 10.1111/j.1469-7998.2008.00524.x

227. Fernández-Tajes, J., Arias-Pérez, A., Fernández-Moreno, M., & Méndez, J. (2012). Sharp decrease of genetic variation in two Spanish localities of razor clam Ensis siliqua: Natural fluctuation or Prestige oil spill effects? Ecotoxicology, 21, 225–233. doi: 10.1007/s10646-011-0781-3

228. Fernández, R., Schubert, M., Vargas-Velázquez, A. M., Brownlow, A., Víkingsson, G. A., Siebert, U., … Orlando, L. (2016). A genomewide catalogue of single nucleotide polymorphisms in white-beaked and Atlantic white-sided dolphins. Molecular Ecology Resources, 16, 266–276. doi: 10.1111/1755-0998.12427

229. Fields, P. D., McCauley, D. E., McAssey, E. V, & Taylor, D. R. (2014). Patterns of cyto-nuclear linkage disequilibrium in Silene latifolia: Genomic heterogeneity and temporal stability. Heredity, 112, 99–104. doi: 10.1038/hdy.2013.79

230. Fillatre, E. K., Etherton, P., & Heath, D. D. (2003). Bimodal run distribution in a northern population of sockeye salmon (Oncorhynchus nerka): Life history and genetic analysis on a temporal scale. Molecular Ecology, 12, 1793–1805. doi: 10.1046/j.1365-294X.2003.01869.x

231. Finger, A. J., Mahardja, B., Fisch, K. M., Benjamin, A., Lindberg, J., Ellison, L., … May, B. (2018). A conservation hatchery population of delta smelt shows evidence of genetic adaptation to captivity after 9 generations. Heredity, 109, 689–699. doi: 10.1093/jhered/esy035

232. Finger, A. J., Parmenter, S., & May, B. P. (2013). Conservation of the owens pupfish: Genetic effects of multiple translocations and extirpations. Transactions of the American Fisheries Society, 142, 1430–1443. doi: 10.1080/00028487.2013.811097

233. Fisch, K. M., Ivy, J. A., Burton, R. S., & May, B. (2013). Evaluating the performance of captive breeding techniques for conservation hatcheries: A case study of the delta smelt captive breeding program. Heredity, 104, 92–104. doi: 10.1093/jhered/ess084

234. Fitak, R. R., Rinkevich, S. E., & Culver, M. (2018). Genome-Wide Analysis of SNPs Is Consistent with No Domestic Dog Ancestry in the Endangered Mexican Wolf (Canis lupus baileyi). Heredity, 109, 372–383. doi: 10.1093/jhered/esy009

235. Flagstad, Ø., Hedmark, E., Landa, A., Brøseth, H., Persson, J., Andersen, R., … Ellegren, H. (2004). Colonization history and noninvasive monitoring of a reestablished wolverine population. Conservation Biology, 18, 676–688. doi: 10.1111/j.1523-1739.2004.00328.x-i1

236. Flagstad, Ø., Syvertsen, P. O., Stenseth, N. C., Stacy, J. E., Olsaker, I., RØed, K. H., & Jakobsen, K. S. (2000). Genetic variability in Swayne's Hartebeest, an endangered antelope of Ethiopia. Conservation Biology, 14, 254–264. doi: 10.1046/j.1523-1739.2000.98339.x

237. Flagstad, Ø., Walker, C. W., Vilà, C., Sundqvist, A. K., Fernholm, B., Hufthammer, A. K., … Ellegren, H. (2003). Two centuries of the Scandinavian wolf population: Patterns of genetic variability and migration during an era of dramatic decline. Molecular Ecology, 12, 869–880. doi: 10.1046/j.1365-294X.2003.01784.x

238. Fonseca, D. M., Widdel, A. K., Hutchinson, M., Spichiger, S. E., & Kramer, L. D. (2010). Fine-scale spatial and temporal population genetics of Aedes japonicus, a new US mosquito, reveal multiple introductions. Molecular Ecology, 19, 1559–1572. doi: 10.1111/j.1365-294X.2010.04576.x

239. Fontaine, M. C., Snirc, A., Frantzis, A., Koutrakis, E., Ožtürk, B., Öžťurk, A. A., & Austerlitz, F. (2012). History of expansion and anthropogenic collapse in a top marine predator of the Black Sea estimated from genetic data. Proceedings of the National Academy of Sciences of the United States of America, 109, E2569–E2576. doi: 10.1073/pnas.1201258109

240. Ford, M. J., Parsons, K. M., Ward, E. J., Hempelmann, J. A., Emmons, C. K., Bradley Hanson, M., … Park, L. K. (2018). Inbreeding in an endangered killer whale population. Animal Conservation, 21, 423–432. doi: 10.1111/acv.12413

241. Foré, S. A., & Guttman, S. I. (1996). Spatial and temporal genetic structure of Asclepias verticillata (whorled milkweed) among prairie patches in a forested landscape. Canadian Journal of Botany, 74, 1289–1297. doi: 10.1139/b96-156

242. Forsberg, N. E. G., Russell, J., Macaulay, M., Leino, M. W., & Hagenblad, J. (2015). Farmers without borders - genetic structuring in century old barley (Hordeum vulgare). Heredity, 114, 195–206. doi: 10.1038/hdy.2014.83

243. Franchini, P., Sola, L., Crosetti, D., Milana, V., & Rossi, A. R. (2012). Low levels of population genetic structure in the gilthead sea bream, Sparus aurata, along the coast of Italy. *ICES Journal of Marine Science*, *69*, 41–50. doi: 10.4135/9781412953924.n678

244. Franck, P., & Timm, A. E. (2010). Population genetic structure of Cydia pomonella: A review and case study comparing spatiotemporal variation. Journal of Applied Entomology, 134, 191–200. doi: 10.1111/j.1439-0418.2009.01426.x

245. Franck, P., Ricci, B., Klein, E. K., Olivares, J., Simon, S., Cornuet, J. M., & Lavigne, C. (2011). Genetic inferences about the population dynamics of codling moth females at a local scale. Genetica, 139, 949–960. doi: 10.1007/s10709-011-9598-5

246. Franklin, M. T., Myers, J. H., & Cory, J. S. (2014). Genetic similarity of island populations of tent caterpillars during successive outbreaks. PLoS ONE, 9, e96679. doi: 10.1371/journal.pone.0096679

247. Frantz, A. C., Hamann, J. L., & Klein, F. (2008). Fine-scale genetic structure of red deer (Cervus elaphus) in a French temperate forest. European Journal of Wildlife Research, 54, 44–52. doi: 10.1007/s10344-007-0107-1

248. Fraser, B. A., Ramnarine, I. W., & Neff, B. D. (2010). Temporal variation at the MHC class iib in wild populations of the guppy (Poecilia reticulata). Evolution, 64, 2086–2096. doi: 10.1111/j.1558-5646.2010.00965.x

249. Fraser, D. J., Calvert, A. M., Bernatchez, L., & Coon, A. (2013). Multidisciplinary population monitoring when demographic data are sparse: A case study of remote trout populations. Ecology and Evolution, 3, 4954–4969. doi: 10.1002/ece3.871

250. Fraser, D. J., Lippé, C., & Bernatchez, L. (2004). Consequences of unequal population size, asymmetric gene flow and sex-biased dispersal on population structure in brook charr (Salvelinus fontinalis). Molecular Ecology, 13, 67–80. doi: 10.1046/j.1365-294X.2003.02038.x

251. Freeland, J. R., Anderson, S., Allen, D., & Looney, D. (2007). Museum samples provide novel insights into the taxonomy and genetic diversity of Irish red grouse. Conservation Genetics, 8, 695–703. doi: 10.1007/s10592-006-9217-7

252. Frisch, D., Morton, P. K., Culver, B. W., Edlund, M. B., Jeyasingh, P. D., & Weider, L. J. (2017). Paleogenetic records of Daphnia pulicaria in two North American lakes reveal the impact of cultural eutrophication. Global Change Biology, 23, 708–718. doi: 10.1111/gcb.13445

253. Fu, J., Lü, W., Li, W., Shen, M., Luo, X., Ke, C., & You, W. (2017). Comparative assessment of the genetic variation in selectively bred generations from two geographic populations of ivory shell (Babylonia areolata). Aquaculture Research, 48, 4205–4218. doi: 10.1111/are.13241

254. Fuentes-Contreras, E., Basoalto, E., Franck, P., Lavandero, B., Knight, A. L., & Ramírez, C. C. (2014). Measuring local genetic variability in populations of codling moth (Lepidoptera: Tortricidae) across an unmanaged and commercial orchard interface. Environmental Entomology, 43, 520–527. doi: 10.1603/EN13131

255. Gallardo, M. H., & Köhler, N. (1994). Demographic changes and genetic losses in populations of a subterranean rodent (Ctenomys maulinus brunneus) affected by a natural catastrophe. Zeitschrift Fur Saugetierkunde, 59, 358–365.

256. Garant, D., Dodson, J. J., & Bernatchez, L. (2000). Ecological determinants and temporal stability of the within-river population structure in Atlantic salmon (Salmo salar L.). Molecular Ecology, 9, 615–628. doi: 10.1046/j.1365-294X.2000.00909.x

257. García-Navas, V., Bonnet, T., Waldvogel, D., Wandeler, P., Camenisch, G., & Postma, E. (2015). Gene flow counteracts the effect of drift in a Swiss population of snow voles fluctuating in size. *Biological Conservation*, *191*, 168–177. doi: 10.1016/j.biocon.2015.06.021

258. Gauffre, B., Berthier, K., Inchausti, P., Chaval, Y., Bretagnolle, V., & Cosson, J. F. (2014). Short-term variations in gene flow related to cyclic density fluctuations in the common vole. Molecular Ecology, 23, 3214–3225. doi: 10.1111/mec.12818

259. Ge, X. J., Hwang, C. C., Liu, Z. H., Huang, C. C., Huang, W. H., Hung, K. H., … Chiang, T. Y. (2011). Conservation genetics and phylogeography of endangered and endemic shrub Tetraena mongolica (Zygophyllaceae) in Inner Mongolia, China. *BMC Genetics*, *12*, 1–12. doi: 10.1186/1471-2156-12-1

260. Geary, B., Longest, S. M., Ottewell, K., Lantz, S. M., Walter, S. T., Karubian, J., & Leberg, P. L. (2017). Genetic structure of brown pelicans (Pelecanus occidentalis) in the northern Gulf of Mexico in the context of human management and disturbance. PLoS ONE, 12, e0185309. doi: 10.1371/journal.pone.0185309

261. Genner, M. J., Nichols, P., Shaw, P. W., Carvalho, G. R., Robinson, R. L., & Turner, G. F. (2010). Population structure on breeding grounds of Lake Malawi's "twilight zone" cichlid fishes. Journal of Biogeography, 37, 258–269. doi: 10.1111/j.1365-2699.2009.02196.x

262. George, S., Sharma, J., & Yadon, V. L. (2009). Genetic diversity of the endangered and narrow endemic Piperia Yadonii (Orchidaceae) assessed with ISSR polymorphisms. American Journal of Botany, 96, 2022–2030. doi: 10.3732/ajb.0800368

263. Głażewska, I., Gralak, B., Naczk, A. M., & Prusak, B. (2018). Genetic diversity and population structure of Polish Arabian horses assessed through breeding and microsatellite data. Animal Science Journal, 89, 735–742. doi: 10.1111/asj.12983

264. Glenn, T. C., & Smith, D. R. (1993). Genetic variation and subspecific relationships of Michigan elk (Cervus elaphus). Journal of Mammalogy, 74, 782–792. doi: 10.2307/1382303

265. Gloag, R., Ding, G., Christie, J. R., Buchmann, G., Beekman, M., & Oldroyd, B. P. (2016). An invasive social insect overcomes genetic load at the sex locus. Nature Ecology & Evolution, 1, 1–6. doi: 10.1038/s41559-016-0011

266. Glover, K. A., Quintela, M., Wennevik, V., Besnier, F., Sørvik, A. G. E., & Skaala, Ø. (2012). Three decades of farmed escapees in the wild: A spatio-temporal analysis of atlantic salmon population genetic structure throughout norway. PLoS ONE, 7, e43129. doi: 10.1371/journal.pone.0043129

267. Gold, J. R., Pak, E., & DeVries, D. A. (2002). Population structure of king mackerel (Scomberomorus cavalla) around peninsular Florida, as revealed by microsatellite DNA. Fishery Bulletin, 100, 491–509.

268. Gold, J. R., Richardson, L. R., Furman, C., & King, T. L. (1993). Mitochondrial DNA differentiation and population structure in red drum (Sciaenops ocellatus) from the Gulf of Mexico and Atlantic Ocean. *Marine Biology*, *116*, 175–185. doi: doi.org/10.1007/BF00350007

269. Goldstien, S. J., Inglis, G. J., Schiel, D. R., & Gemmell, N. J. (2013). Using Temporal Sampling to Improve Attribution of Source Populations for Invasive Species. PLoS ONE, 8, e65656. doi: 10.1371/journal.pone.0065656

270. Golovanov, I. S., Marchenko, S. L., & Pustovoit, S. P. (2009). Genetic monitoring of northern Sea of Okhotsk populations of pink salmon (oncorhynchus gorbuscha). Cytology and Genetics, 43, 379–386. doi: 10.3103/S0095452709060036

271. Gomaa, N. H., Montesinos-Navarro, A., Alonso-Blanco, C., & Picó, F. X. (2011). Temporal variation in genetic diversity and effective population size of Mediterranean and subalpine Arabidopsis thaliana populations. Molecular Ecology, 20, 3540–3554. doi: 10.1111/j.1365-294X.2011.05193.x

272. Gómez, R., Méndez-Vigo, B., Marcer, A., Alonso-Blanco, C., & Picó, F. X. (2018). Quantifying temporal change in plant population attributes: Insights from a resurrection approach. AoB PLANTS, 10, 1–17. doi: 10.1093/aobpla/ply063

273. González-Ittig, R. E., Polop, F. J., Andreo, V. C., Chiappero, M. B., Levis, S., Calderón, G., … Gardenal, C. N. (2015). Temporal fine-scale genetic variation in the zoonosis-carrying long-tailed pygmy rice rat in Patagonia, Argentina. Journal of Zoology, 296, 216–224. doi: 10.1111/jzo.12238

274. Gonzalez, E. G., Beerli, P., & Zardoya, R. (2008). Genetic structuring and migration patterns of Atlantic bigeye tuna, Thunnus obesus (Lowe, 1839). BMC Evolutionary Biology, 8, 1–14. doi: 10.1186/1471-2148-8-252

275. Gordeeva, N. V, Salmenkova, E. A., Altukhov, Y. P., Makhrov, A. A., & Pustovoit, S. P. (2003). Genetic changes in pink salmon Oncorhynchus gorbuscha Walbaum during acclimatization in the White Sea basin. Russian Journal of Genetics, 39, 322–332. doi: 10.1023/A:1023283919776

276. Gottelli, D., Sillero-Zubiri, C., Marino, J., Funk, S. M., & Wang, J. (2013). Genetic structure and patterns of gene flow among populations of the endangered Ethiopian wolf. Animal Conservation, 16, 234–247. doi: 10.1111/j.1469-1795.2012.00591.x

277. Gow, J. L., Noble, L. R., Rollinson, D., Tchuenté, L. A. T., & Jones, C. S. (2007). Contrasting temporal dynamics and spatial patterns of population genetic structure correlate with differences in demography and habitat between two closely-related African freshwater snails. Biological Journal of the Linnean Society, 90, 747–760. doi: 10.1111/j.1095-8312.2007.00771.x

278. Gow, J. L., Tamkee, P., Heggenes, J., Wilson, G. A., & Taylor, E. B. (2011). Little impact of hatchery supplementation that uses native broodstock on the genetic structure and diversity of steelhead trout revealed by a large-scale spatio-temporal microsatellite survey. Evolutionary Applications, 4, 763–782. doi: 10.1111/j.1752-4571.2011.00198.x

279. Gowell, C. P., Quinn, T. P., & Taylor, E. B. (2012). Coexistence and origin of trophic ecotypes of pygmy whitefish, Prosopium coulterii, in a south-western Alaskan lake. Journal of Evolutionary Biology, 25, 2432–2448. doi: 10.1111/jeb.12011

280. Goyache, F., Álvarez, I., Fernández, I., Pérez-Pardal, L., Royo, L. J., & Lorenzo, L. (2011). Usefulness of molecular-based methods for estimating effective population size in livestock assessed using data from the endangered black-coated Asturcón pony. Journal of Animal Science, 89, 1251–1259. doi: 10.2527/jas.2010-3620

281. Gracianne, C., Jan, P. L., Fournet, S., Olivier, E., Arnaud, J. F., Porte, C., … Petit, E. J. (2016). Temporal sampling helps unravel the genetic structure of naturally occurring populations of a phytoparasitic nematode. 2. Separating the relative effects of gene flow and genetic drift. Evolutionary Applications, 9, 1005–1016. doi: 10.1111/eva.12401

282. Grant, W. S., & Cheng, W. (2012). Incorporating deep and shallow components of genetic structure into the management of Alaskan red king crab. Evolutionary Applications, 5, 820–837. doi: 10.1111/j.1752-4571.2012.00260.x

283. Gravot, E., Huet, M., & Veuille, M. (2004). Effect of Breeding Structure on Population Genetic Parameters in Drosophila. Genetics, 166, 779–788. doi: 10.1534/genetics.166.2.779

284. Gredler, J. N., Hish, A. J., & Noor, M. A. F. (2015). Temporal stability of molecular diversity measures in natural populations of drosophila pseudoobscura and drosophila persimilis. Heredity, 106, 407–411. doi: 10.1093/jhered/esv027

285. Greene, D. U., Gore, J. A., & Austin, J. D. (2017). Reintroduction of captive-born beach mice: The importance of demographic and genetic monitoring. Journal of Mammalogy, 98, 513–522. doi: 10.1093/jmammal/gyw229

286. Groombridge, J. J., Dawson, D. A., Burke, T., Prys-Jones, R., Brooke, M. de L., & Shah, N. (2009). Evaluating the demographic history of the Seychelles kestrel (Falco araea): Genetic evidence for recovery from a population bottleneck following minimal conservation management. *Biological Conservation*, *142*, 2250–2257. doi: 10.1016/j.biocon.2009.04.026

287. Grueber, C. E., Wallis, G. P., & Jamieson, I. G. (2013). Genetic drift outweighs natural selection at toll-like receptor (TLR) immunity loci in a re-introduced population of a threatened species. Molecular Ecology, 22, 4470–4482. doi: 10.1111/mec.12404

288. Guardiola, M., Frotscher, J., & Uriz, M. J. (2012). Genetic structure and differentiation at a short-time scale of the introduced calcarean sponge Paraleucilla magna to the western Mediterranean. Hydrobiologia, 687, 71–84. doi: 10.1007/s10750-011-0948-1

289. Gum, B., Geist, J., Eckenfels, S., & Brinker, A. (2014). Genetic diversity of upper Lake Constance whitefish Coregonus spp. under the influence of fisheries: A DNA study based on archived scale samples from 1932, 1975 and 2006. Journal of Fish Biology, 84, 1721–1739. doi: 10.1111/jfb.12393

290. Gurung, S., Short, D. P. G., Atallah, Z. K., & Subbarao, K. V. (2014). Clonal expansion of verticillium dahliae in lettuce. Phytopathology, 104, 641–649. doi: 10.1094/PHYTO-10-13-0282-R

291. Habel, J. C., Finger, A., Schmitt, T., & Nève, G. (2011). Survival of the endangered butterfly Lycaena helle in a fragmented environment: Genetic analyses over 15 years. Journal of Zoological Systematics and Evolutionary Research, 49, 25–31. doi: 10.1111/j.1439-0469.2010.00575.x

292. Hadly, E. A., Ramakrishnan, U., Chan, Y. L., Van Tuinen, M., O'Keefe, K., Spaeth, P. A., & Conroy, C. J. (2004). Genetic response to climatic change: Insights from ancient DNA and phylochronology. PLoS Biology, 2, 1600–1609. doi: 10.1371/journal.pbio.0020290

293. Hagenblad, J., Zie, J., & Leino, M. W. (2012). Exploring the population genetics of genebank and historical landrace varieties. Genetic Resources and Crop Evolution, 59, 1185–1199. doi: 10.1007/s10722-011-9754-x

294. Hájková, P., Pertoldi, C., Zemanová, B., Roche, K., Hájek, B., Bryja, J., & Zima, J. (2007). Genetic structure and evidence for recent population decline in Eurasian otter populations in the Czech and Slovak Republics: Implications for conservation. Journal of Zoology, 272, 1–9. doi: 10.1111/j.1469-7998.2006.00259.x

295. Hamilton, P. B., Nicol, E., De-Bastos, E. S. R., Williams, R. J., Sumpter, J. P., Jobling, S., … Tyler, C. R. (2014). Populations of a cyprinid fish are self-sustaining despite widespread feminization of males. *BMC Biology*, *12*, 1–13. doi: 10.1186/1741-7007-12-1

296. Han, Q., & Caprio, M. A. (2002). Temporal and spatial patterns of allelic frequencies in cotton bollworm (Lepidoptera: Noctuidae). Environmental Entomology, 31, 462–468. doi: 10.1603/0046-225X-31.3.462

297. Han, Q., & Caprio, M. A. (2004). Evidence from genetic markers suggests seasonal variation in dispersal in Heliothis virescens (Lepidoptera: Noctuidae). Environmental Entomology, 33, 1223–1231. doi: 10.1603/0046-225X-33.5.1223

298. Han, Y. S., Sun, Y. L., Liao, Y. F., Liao, I. C., Shen, K. N., & Tzeng, W. N. (2008). Temporal analysis of population genetic composition in the overexploited Japanese eel Anguilla japonica. Marine Biology, 155, 613–621. doi: 10.1007/s00227-008-1057-1

299. Hansen, M. M. (2002). Estimating the long-term effects of stocking domesticated trout into wild brown trout (Salmo trutta) populations: An approach using microsatellite DNA analysis of historical and contemporary samples. Molecular Ecology, 11, 1003–1015. doi: 10.1046/j.1365-294X.2002.01495.x

300. Hansen, M. M., & Mensberg, K. L. D. (2009). Admixture analysis of stocked brown trout populations using mapped microsatellite DNA markers: Indigenous trout persist in introgressed populations. Biology Letters, 5, 656–659. doi: 10.1098/rsbl.2009.0214

301. Hansen, M. M., Limborg, M. T., Ferchaud, A. L., & Pujolar, J. M. (2014). The effects of medieval dams on genetic divergence and demographic history in brown trout populations. BMC Evolutionary Biology, 14, 1–14. doi: 10.1186/1471-2148-14-122

302. Hansen, M. M., Meier, K., & Mensberg, K. L. D. (2010). Identifying footprints of selection in stocked brown trout populations: A spatio-temporal approach. Molecular Ecology, 19, 1787–1800. doi: 10.1111/j.1365-294X.2010.04615.x

303. Hansen, M. M., Nielsen, E. E., & Mensberg, K. L. D. (2006). Underwater but not out of sight: Genetic monitoring of effective population size in the endangered North Sea houting (Coregonus oxyrhynchus). Canadian Journal of Fisheries and Aquatic Sciences, 63, 780–787. doi: 10.1139/f05-260

304. Hansen, M. M., Ruzzante, D. E., Nielsen, E. E., Bekkevold, D., & Mensberg, K. L. D. (2002). Long-term effective population sizes, temporal stability of genetic composition and potential for local adaptation in anadromous brown trout (Salmo trutta) populations. Molecular Ecology, 11, 2523–2535. doi: 10.1046/j.1365-294X.2002.01634.x

305. Hansen, M. M., Skaala, Ø., Jensen, L. F., Bekkevold, D., & Mensberg, K. L. D. (2007). Gene flow, effective population size and selection at major histocompatibility complex genes: Brown trout in the Hardanger Fjord, Norway. Molecular Ecology, 16, 1413–1425. doi: 10.1111/j.1365-294X.2007.03255.x

306. Haponski, A. E., & Stepien, C. A. (2016). Two decades of genetic consistency in a reproductive population in the face of exploitation: patterns of adult and larval walleye (Sander vitreus) from Lake Erie's Maumee River. Conservation Genetics, 17, 1345–1362. doi: 10.1007/s10592-016-0866-x

307. Harkins, G. W., D'Amato, M. E., & Gibbons, M. J. (2013). Self-maintaining or continuously refreshed? The genetic structure of Euphausia lucens populations in the Benguela upwelling ecosystem. Journal of Plankton Research, 35, 982–992. doi: 10.1093/plankt/fbt046

308. Harper, G. L., Maclean, N., & Goulson, D. (2006). Analysis of museum specimens suggests extreme genetic drift in the adonis blue butterfly (Polyommatus bellargus). Biological Journal of the Linnean Society, 88, 447–452. doi: 10.1111/j.1095-8312.2006.00632.x

309. Harrisson, K. A., Pavlova, A., Gonçąlves Da Silva, A., Rose, R., Bull, J. K., Lancaster, M. L., … Sunnucks, P. (2016). Scope for genetic rescue of an endangered subspecies though re-establishing natural gene flow with another subspecies. Molecular Ecology, 25, 1242–1258. doi: 10.1111/mec.13547

310. Hartmann, S. A., Schaefer, H. M., & Segelbacher, G. (2014). Genetic depletion at adaptive but not neutral loci in an endangered bird species. Molecular Ecology, 23, 5712–5725. doi: 10.1111/mec.12975

311. Hasselgren, M., Angerbjörn, A., Eide, N. E., Erlandsson, R., Flagstad, Ø., Landa, A., … Norén, K. (2018). Genetic rescue in an inbred Arctic fox (Vulpes lagopus) population. Proceedings of the Royal Society B, 285, 1–9. doi: 10.1098/rspb.2017.2814

312. Hauser, L., Adcock, G. J., Smith, P. J., Ramírez, J. H. B., & Carvalho, G. R. (2002). Loss of microsatellite diversity and low effective population size in an overexploited population of New Zealand snapper (Pagrus auratus). Proceedings of the National Academy of Sciences of the United States of America, 99, 11742–11747. doi: 10.1073/pnas.172242899

313. Heber, S., Varsani, A., Kuhn, S., Girg, A., Kempenaers, B., & Briskie, J. (2013). The genetic rescue of two bottlenecked south island robin populations using translocations of inbred donors. Proceedings of the Royal Society B, 280, 1–8. doi: 10.1098/rspb.2012.2228

314. Hedgecock, D., Hutchinson, E. S., Li, G., Sly, F. L., & Nelson, K. (1994). The central stock of northern anchovy (Engraulis mordax) is not a randomly mating population. *California Cooperative Oceanic Fisheries Investigations Report*, *35*, 121–136. Retrieved from http://calcofi.ucsd.edu/newhome/publications/CalCOFI_Reports/v35/pdfs/Vol_35_Hedgecock_etal.pdf

315. Hedrick, P. W., & Wehausen, J. D. (2014). Desert bighorn sheep: Changes in genetic variation over time and the impact of merging populations. Journal of Fish and Wildlife Management, 5, 3–13. doi: 10.3996/082013-JFWM-055

316. Hedrick, P. W., Peterson, R. O., Vucetich, L. M., Adams, J. R., & Vucetich, J. A. (2014). Genetic rescue in Isle Royale wolves: genetic analysis and the collapse of the population. Conservation Genetics, 15, 1111–1121. doi: 10.1007/s10592-014-0604-1

317. Hemmer-Hansen, J., Nielsen, E. E., Grønkjær, P., & Loeschcke, V. (2007). Evolutionary mechanisms shaping the genetic population structure of marine fishes; lessons from the European flounder (Platichthys flesus L.). Molecular Ecology, 16, 3104–3118. doi: 10.1111/j.1365-294X.2007.03367.x

318. Henriques, R., Nielsen, E. S., Durholtz, D., Japp, D., & von der Heyden, S. (2017). Genetic population sub-structuring of kingklip (Genypterus capensis – Ophidiidiae), a commercially exploited demersal fish off South Africa. *Fisheries Research*, *187*, 86–95. doi: 10.1016/j.fishres.2016.11.007

319. Henriques, R., von der Heyden, S., Lipinski, M. R., du Toit, N., Kainge, P., Bloomer, P., & Matthee, C. A. (2016). Spatio-temporal genetic structure and the effects of long-term fishing in two partially sympatric offshore demersal fishes. Molecular Ecology, 25, 5843–5861. doi: 10.1111/mec.13890

320. Hepburn, R. I., Sale, P. F., Dixon, B., & Heath, D. D. (2009). Genetic structure of juvenile cohorts of bicolor damselfish (Stegastes partitus) along the Mesoamerican barrier reef: Chaos through time. Coral Reefs, 28, 277–288. doi: 10.1007/s00338-008-0423-2

321. Herbinger, C. M., Vercaemer, B. M., Gjetvaj, B., & O'Dor, R. K. (1998). Absence of genetic differentiation among geographically close sea scallop (Placopecten magellanicus G.) beds with cDNA and microsatellite markers. Journal of Shellfish Research, 17, 117–122. doi: 10.2983/0730-8000(2007)26[675:P]2.0.CO;2

322. Herborg, L. M., Weetman, D., Van Oosterhout, C., & Hänfling, B. (2007). Genetic population structure and contemporary dispersal patterns of a recent European invader, the Chinese mitten crab, Eriocheir sinensis. Molecular Ecology, 16, 231–242. doi: 10.1111/j.1365-294X.2006.03133.x

323. Herrera-Arroyo, M. L., Sork, V. L., González-Rodríguez, A., Rocha-Ramírez, V., Vega, E., & Oyama, K. (2013). Seed-mediated connectivity among fragmented populations of Quercus castanea (Fagaceae) in a Mexican landscape. American Journal of Botany, 100, 1663–1671. doi: 10.3732/ajb.1200396

324. Herzog, R., & Hadrys, H. (2017). Long-Term genetic monitoring of a riverine dragonfly, Orthetrum coerulescens (Odonata: Libellulidae]: Direct anthropogenic impact versus climate change effects. PLoS ONE, 12, e0178014. doi: 10.1371/journal.pone.0178014

325. Hess, J. E., & Matala, A. P. (2014). Archival genetic analysis suggests recent immigration has altered a population of Chinook salmon in an unsupplemented wilderness area. Conservation Genetics, 15, 387–403. doi: 10.1007/s10592-013-0546-z

326. Hillman, T. L., Keenlance, P., Moore, J. A., Swanson, B. J., Jacquot, J. J., Witt, J. C., & Cornman, A. (2017). Genetic diversity of reintroduced American martens in Michigan’s Lower Peninsula. Journal of Mammalogy, 98, 1489–1496. doi: 10.1093/jmammal/gyx075

327. Hinkson, K. M., & Richter, S. C. (2016). Temporal trends in genetic data and effective population size support efficacy of management practices in critically endangered dusky gopher frogs (Lithobates sevosus). Ecology and Evolution, 6, 2667–2678. doi: 10.1002/ece3.2084

328. Hirase, S., Ikeda, M., Hayasaka, S., Iwasaki, W., & Kijima, A. (2016). Stability of genetic diversity in an intertidal goby population after exposure to tsunami disturbance. Marine Ecology, 37, 1161–1167. doi: 10.1111/maec.12377

329. Hoareau, T. B., Bosc, P., Valade, P., & Berrebi, P. (2007). Gene flow and genetic structure of Sicyopterus lagocephalus in the south-western Indian Ocean, assessed by intron-length polymorphism. Journal of Experimental Marine Biology and Ecology, 349, 223–234. doi: 10.1016/j.jembe.2007.05.015

330. Hodges, T. K., Athrey, G., Deitz, K. C., Overgaard, H. J., Matias, A., Caccone, A., & Slotman, M. A. (2013). Large fluctuations in the effective population size of the malaria mosquito Anopheles gambiae s.s. during vector control cycle. Evolutionary Applications, 6, 1171–1183. doi: 10.1111/eva.12094

331. Hoehn, M., Gruber, B., Sarre, S. D., Lange, R., & Henle, K. (2012). Can genetic estimators provide robust estimates of the effective number of breeders in small populations? PloS ONE, 7, e48464. doi: 10.1371/journal.pone.0048464

332. Hoelzel, A. R., Fleischer, R. C., Campagna, C., Le Boeuf, B. J., & Alvord, G. (2002). Impact of a population bottleneck on symmetry and genetic diversity in the northern elephant seal. Journal of Evolutionary Biology, 15, 567–575. doi: 10.1046/j.1420-9101.2002.00419.x

333. Hoffman, E. A., Schueler, F. W., & Blouin, M. S. (2004). Effective population sizes and temporal stability of genetic structure in Rana pipiens, the northern leopard frog. Evolution, 58, 2536–2545. doi: 10.1111/j.0014-3820.2004.tb00882.x

334. Hoffman, J. I., Grant, S. M., Forcada, J., & Phillips, C. D. (2011). Bayesian inference of a historical bottleneck in a heavily exploited marine mammal. Molecular Ecology, 20, 3989–4008. doi: 10.1111/j.1365-294X.2011.05248.x

335. Hoffmann, G. S., Johannesen, J., & Griebeler, E. M. (2016). Population dynamics of a natural red deer population over 200 years detected via substantial changes of genetic variation. Ecology and Evolution, 6, 3146–3153. doi: 10.1002/ece3.2063

336. Hogg, I. D., De Lafontaine, Y., & Eadie, J. M. (2000). Genotypic variation among Gammarus fasciatus (Crustacea: Amphipoda) from the Great Lakes - St. Lawrence River: Implications for the conservation of widespread freshwater invertebrates. Canadian Journal of Fisheries and Aquatic Sciences, 57, 1843–1852. doi: 10.1139/f00-136

337. Holbrook, J. D., Deyoung, R. W., Tewes, M. E., & Young, J. H. (2012). Demographic history of an elusive carnivore: Using museums to inform management. Evolutionary Applications, 5, 619–628. doi: 10.1111/j.1752-4571.2012.00241.x

338. Holmes, I., & Crawford, A. (2015). Temporal population genetic instability in range-edge western toads, anaxyrus boreas. Heredity, 106, 45–56. doi: 10.1093/jhered/esu068

339. Honka, J., Heino, M. T., Kvist, L., Askeyev, I. V, Shaymuratova, D. N., Askeyev, O. V, … Aspi, J. (2018). Over a thousand years of evolutionary history of domestic geese from Russian archaeological sites, analysed using ancient DNA. Genes, 9, 1–18. doi: 10.3390/genes9070367

340. Hoos, P. M., Whitman Miller, A., Ruiz, G. M., Vrijenhoek, R. C., & Geller, J. B. (2010). Genetic and historical evidence disagree on likely sources of the Atlantic amethyst gem clam Gemma gemma (Totten, 1834) in California. Diversity and Distributions, 16, 582–592. doi: 10.1111/j.1472-4642.2010.00672.x

341. Horne, J. B., Momigliano, P., Van Herwerden, L., & Newman, S. J. (2013). Murky waters: Searching for structure in genetically depauperate blue threadfin populations of Western Australia. *Fisheries Research*, *146*, 1–6. doi: 10.1016/j.fishres.2013.03.013

342. Horreo, J. L., Machado-Schiaffino, G., Griffiths, A. M., Bright, D., Stevens, J. R., & Garcia-Vazquez, E. (2011). Atlantic salmon at risk: Apparent rapid declines in effective population size in Southern European Populations. Transactions of the American Fisheries Society, 140, 605–610. doi: 10.1080/00028487.2011.585574

343. Hossaert-Mckey, M., Valero, M., Magda, D., Jarry, M., Cuguen, J., & Vernet, P. (1996). The evolving genetic history of a population of Lathyrus sylvestris: evidence from temporal and spatial genetic structure. Evolution, 50, 1808–1821. doi: 10.1111/j.1558-5646.1996.tb03567.x

344. Hsu, J. L., Crawford, J. C., Tammone, M. N., Ramakrishnan, U., Lacey, E. A., & Hadly, E. A. (2017). Genomic data reveal a loss of diversity in two species of tuco-tucos (genus Ctenomys) following a volcanic eruption. Scientific Reports, 7, 1–14. doi: 10.1038/s41598-017-16430-1

345. Huang, S., Molaei, G., & Andreadis, T. G. (2008). Genetic insights into the population structure of Culex pipiens (Diptera: Culicidae) in the northeastern United States by using microsatellite analysis. American Journal of Tropical Medicine and Hygiene, 79, 518–527. doi: 10.4269/ajtmh.2008.79.518

346. Huber, K., Luu, L. L., Tran, H. H., Tran, K. T., Rodhain, F., & Failloux, A. B. (2002). Temporal genetic variation in Aedes aegypti populations in Ho Chi Minh City (Vietnam). Heredity, 89, 7–14. doi: 10.1038/sj.hdy.6800086

347. Hudson, M. A., Young, R. P., D'Urban Jackson, J., Orozco-Terwengel, P., Martin, L., James, A., … Cunningham, A. A. (2016). Dynamics and genetics of a disease-driven species decline to near extinction: Lessons for conservation. *Scientific Reports, 6*, 1–13. doi: 10.1038/srep30772

348. Hughes, S., Fernández, H., Cucchi, T., Duffraisse, M., Casabianca, F., Istria, D., … Taberlet, P. (2012). A dig into the past mitochondrial diversity of corsican goats reveals the influence of secular herding practices. PLoS ONE, 7, e30272. doi: 10.1371/journal.pone.0030272

349. Husemann, M., Nguyen, R., Ding, B., & Danley, P. D. (2015). A genetic demographic analysis of Lake Malawi rock-dwelling cichlids using spatio-temporal sampling. Molecular Ecology, 24, 2686–2701. doi: 10.1111/mec.13205

350. Inoue, M. N., Ito, F., & Goka, K. (2015). Queen execution increases relatedness among workers of the invasive Argentine ant, Linepithema humile. Ecology and Evolution, 5, 4098–4107. doi: 10.1002/ece3.1681

351. Ishibashi, Y., Oi, T., Arimoto, I., Fujii, T., Mamiya, K., Nishi, N., … Yamada, T. (2017). Loss of allelic diversity in the MHC class II DQB gene in western populations of the Japanese black bear Ursus thibetanus japonicus. Conservation Genetics, 18, 247–260. doi: 10.1007/s10592-016-0897-3

352. Israel, J. A., & May, B. (2010). Indirect genetic estimates of breeding population size in the polyploid green sturgeon (Acipenser medirostris). Molecular Ecology, 19, 1058–1070. doi: 10.1111/j.1365-294X.2010.04533.x

353. Jacobsen, F., Nesje, M., Bachmann, L., & Lifjeld, J. T. (2008). Significant genetic admixture after reintroduction of peregrine falcon (Falco peregrinus) in Southern Scandinavia. Conservation Genetics, 9, 581–591. doi: 10.1007/s10592-007-9373-4

354. Jacquet, S., Huber, K., Guis, H., Setier-Rio, M. L., Goffredo, M., Allène, X., … Garros, C. (2016). Spatio-temporal genetic variation of the biting midge vector species Culicoides imicola (Ceratopogonidae) Kieffer in France. Parasites and Vectors, 9, 1–12. doi: 10.1186/s13071-016-1426-4

355. Jaffé, R., Dietemann, V., Crewe, R. M., & Moritz, R. F. A. (2009). Temporal variation in the genetic structure of a drone congregation area: An insight into the population dynamics of wild African honeybees (Apis mellifera scutellata). Molecular Ecology, 18, 1511–1522. doi: 10.1111/j.1365-294X.2009.04143.x

356. Jahnke, M., Serra, I. A., Bernard, G., & Procaccini, G. (2015). The importance of genetic make-up in seagrass restoration: A case study of the seagrass Zostera noltei. *Marine Ecology Progress Series*, *532*, 111–122. doi: 10.3354/meps11355

357. Jaimes-Dueñez, J., Arboleda, S., Triana-Chávez, O., & Gómez-Palacio, A. (2015). Spatio-Temporal Distribution of Aedes aegypti (Diptera: Culicidae) Mitochondrial Lineages in Cities with Distinct Dengue Incidence Rates Suggests Complex Population Dynamics of the Dengue Vector in Colombia. PLoS Neglected Tropical Diseases, 9, e0003553. doi: 10.1371/journal.pntd.0003553

358. Janečka, J. E., Tewes, M. E., Laack, L. L., Grassman, L. I., Haines, A. M., & Honeycutt, R. L. (2008). Small effective population sizes of two remnant ocelot populations (Leopardus pardalis albescens) in the United States. Conservation Genetics, 9, 869–878. doi: 10.1007/s10592-007-9412-1

359. Janecka, J. E., Tewes, M. E., Laack, L., Caso, A., Grassman, L. I., & Honeycutt, R. L. (2014). Loss of genetic diversity among ocelots in the United States during the 20th century linked to human induced population reductions. PLoS ONE, 9, e89384. doi: 10.1371/journal.pone.0089384

360. Jangjoo, M., Matter, S. F., Roland, J., & Keyghobadi, N. (2016). Connectivity rescues genetic diversity after a demographic bottleneck in a butterfly population network. Proceedings of the National Academy of Sciences of the United States of America, 113, 10914–10919. doi: 10.1073/pnas.1600865113

361. Jansson, E., Ruokonen, M., Kojola, I., & Aspi, J. (2012). Rise and fall of a wolf population: Genetic diversity and structure during recovery, rapid expansion and drastic decline. Molecular Ecology, 21, 5178–5193. doi: 10.1111/mec.12010

362. Jansson, M., & Laikre, L. (2014). Recent breeding history of dog breeds in Sweden: Modest rates of inbreeding, extensive loss of genetic diversity and lack of correlation between inbreeding and health. Journal of Animal Breeding and Genetics, 131, 153–162. doi: 10.1111/jbg.12060

363. Jara, L., Muñoz, I., Cepero, A., Martín-Hernández, R., Serrano, J., Higes, M., & De la Rúa, P. (2015). Stable genetic diversity despite parasite and pathogen spread in honey bee colonies. Science of Nature, 102, 1–8. doi: 10.1007/s00114-015-1298-z

364. Jason Kennington, W., Hevroy, T. H., & Johnson, M. S. (2012). Long-term genetic monitoring reveals contrasting changes in the genetic composition of newly established populations of the intertidal snail Bembicium vittatum. Molecular Ecology, 21, 3489–3500. doi: 10.1111/j.1365-294X.2012.05636.x

365. Jay, K., Crossman, J. A., & Scribner, K. T. (2014). Estimates of Effective Number of Breeding Adults and Reproductive Success for White Sturgeon. Transactions of the American Fisheries Society, 143, 1204–1216. doi: 10.1080/00028487.2014.931301

366. Jensen, A. B., Hansen, L. M., & Eilenberg, J. (2008). Grain aphid population structure: No effect of fungal infections in a 2-year field study in Denmark. Agricultural and Forest Entomology, 10, 279–290. doi: 10.1111/j.1461-9563.2008.00383.x

367. Jensen, E. L., Miller, J. M., Edwards, D. L., Garrick, R. C., Tapia, W., Caccone, A., & Russello, M. A. (2018). Temporal mitogenomics of the Galapagos giant tortoise from Pinzón reveals potential biases in population genetic inference. Heredity, 109, 631–640. doi: 10.1093/jhered/esy016

368. Jensen, L. F., Hansen, M. M., Carlsson, J., Loeschcke, V., & Mensberg, K. L. D. (2005). Spatial and temporal genetic differentiation and effective population size of brown trout (Salmo trutta, L.) in small Danish rivers. Conservation Genetics, 6, 615–621. doi: 10.1007/s10592-005-9014-8

369. Jensen, L. F., Hansen, M. M., Mensberg, K. L. D., & Loeschcke, V. (2008). Spatially and temporally fluctuating selection at non-MHC immune genes: Evidence from TAP polymorphism in populations of brown trout (Salmo trutta, L.). Heredity, 100, 79–91. doi: 10.1038/sj.hdy.6801067

370. Jensen, M. P., Bell, I., Limpus, C. J., Hamann, M., Ambar, S., Whap, T., … FitzSimmons, N. N. (2016). Spatial and temporal genetic variation among size classes of green turtles (Chelonia mydas) provides information on oceanic dispersal and population dynamics. *Marine Ecology Progress Series*, *543*, 241–256. doi: 10.3354/meps11521

371. Johnsen, A., Brabrand, Å., Anmarkrud, J. A., Bjørnstad, G., Pavels, H., & Saltveit, S. J. (2014). Impact of human-induced environmental changes on genetic structure and variability in Atlantic salmon, Salmo salar. Fisheries Management and Ecology, 21, 32–41. doi: 10.1111/fme.12049

372. Johnson, B. M., Kemp, B. M., & Thorgaard, G. H. (2018). Increased mitochondrial DNA diversity in ancient Columbia River basin Chinook salmon Oncorhynchus tshawytscha. PLoS ONE, 13, e0190059. doi: 10.1371/journal.pone.0190059

373. Johnson, J. A., Bellinger, M. R., Toepfer, J. E., & Dunn, P. (2004). Temporal changes in allele frequencies and low effective population size in greater prairie-chickens. Molecular Ecology, 13, 2617–2630. doi: 10.1111/j.1365-294X.2004.02264.x

374. Johnson, J. A., Dunn, P. O., & Bouzat, J. L. (2007). Effects of recent population bottlenecks on reconstructing the demographic history of prairie-chickens. Molecular Ecology, 16, 2203–2222. doi: 10.1111/j.1365-294X.2007.03285.x

375. Johnson, J. A., Gilbert, M., Virani, M. Z., Asim, M., & Mindell, D. P. (2008). Temporal genetic analysis of the critically endangered oriental white-backed vulture in Pakistan. Biological Conservation, 141, 2403–2409. doi: 10.1016/j.biocon.2008.07.001

376. Johnson, J. A., Talbot, S. L., Sage, G. K., Burnham, K. K., Brown, J. W., Maechtle, T. L., … Mindell, D. P. (2010). The use of genetics for the management of a recovering population: Temporal assessment of migratory peregrine falcons in North America. PLoS ONE, 5, e14042. doi: 10.1371/journal.pone.0014042

377. Johnson, W. E., Onorato, D. P., Roelke, M. E., Land, E. D., Cunningham, M., Belden, R. C., … O'Brien, S. J. (2010). Genetic restoration of the Florida panther. Science, 329, 1641–1645. doi: 10.1126/science.1192891

378. Jolly, M. T., Maitland, P. S., & Genner, M. J. (2011). Genetic monitoring of two decades of hybridization between allis shad (Alosa alosa) and twaite shad (Alosa fallax). Conservation Genetics, 12, 1087–1100. doi: 10.1007/s10592-011-0211-3

379. Jones, P. H., Biggins, D. E., Eads, D. A., Eads, S. L., & Britten, H. B. (2012). Deltamethrin flea-control preserves genetic variability of black-tailed prairie dogs during a plague outbreak. Conservation Genetics, 13, 183–195. doi: 10.1007/s10592-011-0275-0

380. Jónsdóttir, Ó. D. B., Daníelsdóttir, A. K., & Nævdal, G. (2001). Genetic differentiation among Atlantic cod (Gadus morhua L.) in Icelandic waters: Temporal stability. ICES Journal of Marine Science, 58, 114–122. doi: 10.1006/jmsc.2000.0995

381. Jourdan-Pineau, H., David, P., & Crochet, P. A. (2012). Phenotypic plasticity allows the Mediterranean parsley frog Pelodytes punctatus to exploit two temporal niches under continuous gene flow. Molecular Ecology, 21, 876–886. doi: 10.1111/j.1365-294X.2011.05420.x

382. Juárez, O. E., Enríquez, L., Camarena-Rosales, F., Arena, L., Galindo-Sánchez, C. E., Lafarga-De la Cruz, F., … Rosas, C. (2018). Genetic monitoring of the Mexican four-eyed octopus Octopus maya population: New insights and perspectives for the fishery management. Fisheries Research, 206, 109–114. doi: 10.1016/j.fishres.2018.05.002

383. Juarez, R. L., Schwartz, M. K., Pilgrim, K. L., Thompson, D. J., Tucker, S. A., Smith, J. B., & Jenks, J. A. (2016). Assessing temporal genetic variation in a cougar population: influence of harvest and neighboring populations. Conservation Genetics, 17, 379–388. doi: 10.1007/s10592-015-0790-5

384. Jueterbock, A., Coyer, J. A., Olsen, J. L., & Hoarau, G. (2018). Decadal stability in genetic variation and structure in the intertidal seaweed Fucus serratus (Heterokontophyta: Fucaceae). BMC Evolutionary Biology, 18, 1–12. doi: 10.1186/s12862-018-1213-2

385. Junge, C., Vøllestad, L. A., Barson, N. J., Haugen, T. O., Otero, J., Sætre, G. P., … Primmer, C. R. (2011). Strong gene flow and lack of stable population structure in the face of rapid adaptation to local temperature in a spring-spawning salmonid, the European grayling (Thymallus thymallus). Heredity, 106, 460–471. doi: 10.1038/hdy.2010.160

386. Kaeuffer, R., Coltman, D. W., Chapuis, J. L., Pontier, D., & Réale, D. (2007). Unexpected heterozygosity in an island mouflon population founded by a single pair of individuals. Proceedings of the Royal Society B, 274, 527–533. doi: 10.1098/rspb.2006.3743

387. Kalkvik, H. M., Stout, I. J., & Parkinson, C. L. (2012). Unraveling natural versus anthropogenic effects on genetic diversity within the southeastern beach mouse (Peromyscus polionotus niveiventris). Conservation Genetics, 13, 1653–1664. doi: 10.1007/s10592-012-0417-z

388. Kamath, P. L., Haroldson, M. A., Luikart, G., Paetkau, D., Whitman, C., & Van Manen, F. T. (2015). Multiple estimates of effective population size for monitoring a long-lived vertebrate: An application to Yellowstone grizzly bears. Molecular Ecology, 24, 5507–5521. doi: 10.1111/mec.13398

389. Kanapeckas, K. L., Vigueira, C. C., Ortiz, A., Gettler, K. A., Burgos, N. R., Fischer, A. J., & Lawton-Rauh, A. L. (2016). Escape to ferality: The endoferal origin of weedy rice from crop rice through de-domestication. PLoS ONE, 11, e0162676. doi: 10.1371/journal.pone.0162676

390. Kanthaswamy, S., & Smith, D. G. (2002). Assessment of genetic management at three specific-pathogen-free rhesus macaque (Macaca mulatta) colonies. Comparative Medicine, 52, 414–423.

391. Karaiskou, N., Lappa, M., Kalomoiris, S., Oikonomidis, G., Psaltopoulou, C., Abatzopoulos, T. J., … Triantafyllidis, A. (2011). Genetic monitoring and effects of stocking practices on small Cyprinus carpio populations. Conservation Genetics, 12, 1299–1311. doi: 10.1007/s10592-011-0231-z

392. Katalinas, C. J., Brenkert, K., Darden, T., & Denson, M. R. (2018). A Genetic Assessment of a Red Drum, Sciaenops ocellatus, Stock Enhancement Program. Journal of the World Aquaculture Society, 49, 523–539. doi: 10.1111/jwas.12442

393. Katz, E. M., Tolley, K. A., & Bishop, J. M. (2014). Temporal changes in allelic variation among Cape Dwarf Chameleons, Bradypodion pumilum, inhabiting a transformed, semi-urban wetland. African Journal of Herpetology, 63, 1–12. doi: 10.1080/21564574.2013.834852

394. Keiper, F. J., Haque, M. S., Hayden, M. J., & Park, R. F. (2006). Genetic diversity in Australian populations of Puccinia graminis f. sp. avenae. Phytopathology, 96, 96–104. doi: 10.1094/PHYTO-96-0096

395. Kekkonen, J., Hanski, I. K., Jensen, H., Väisänen, R. A., & Brommer, J. E. (2011). Increased genetic differentiation in house sparrows after a strong population decline: From panmixia towards structure in a common bird. Biological Conservation, 144, 2931–2940. doi: 10.1016/j.biocon.2011.08.012

396. Keller, L. F., Jeffery, K. J., Arcese, P., Beaumont, M. A., Hochachka, W. M., Smith, J. N. M., & Bruford, M. W. (2001). Immigration and the ephemerality of a natural population bottleneck: Evidence from molecular markers. Proceedings of the Royal Society B, 268, 1387–1394. doi: 10.1098/rspb.2001.1607

397. Kennington, W. J., Cadee, S. A., Berry, O., Groth, D. M., Johnson, M. S., & Melville-Smith, R. (2013). Maintenance of genetic variation and panmixia in the commercially exploited western rock lobster (Panulirus cygnus). Conservation Genetics, 14, 115–124. doi: 10.1007/s10592-012-0433-z

398. Kennington, W. J., Keron, P. W., Harvey, E. S., Wakefield, C. B., Williams, A. J., Halafihi, T., & Newman, S. J. (2017). High intra-ocean, but limited inter-ocean genetic connectivity in populations of the deep-water oblique-banded snapper Pristipomoides zonatus (Pisces: Lutjanidae). Fisheries Research, 193, 242–249. doi: 10.1016/j.fishres.2017.04.015

399. Khrustaleva, A. M., Klovach, N. V, Gritsenko, O. F., & Seeb, J. E. (2014). Intra- and interpopulation variability of southwestern Kamchatka sockeye salmon Oncorhynchus nerka inferred from the data on single nucleotide polymorphism. Russian Journal of Genetics, 50, 736–748. doi: 10.1134/S1022795414070096

400. Khrustaleva, A. M., Klovach, N. V, Vedischeva, E. V, & Seeb, J. E. (2015). Genetic differentiation of sockeye salmon Oncorhynchus nerka from Kamchatka River basin and the lake–river systems of the west coast of the bering sea as inferred from data on single nucleotide polymorphism. Russian Journal of Genetics, 51, 980–991. doi: 10.1134/S1022795415090057

401. Kiatsopit, N., Sithithaworn, P., Saijuntha, W., Pitaksakulrat, O., Petney, T. N., Webster, J. P., & Andrews, R. H. (2014). Analysis of the population genetics of Opisthorchis viverrini sensu lato in the Nam Ngum River wetland, Lao PDR, by multilocus enzyme electrophoresis. Parasitology Research, 113, 2973–2981. doi: 10.1007/s00436-014-3959-9

402. Kierepka, E. M., Kilgo, J. C., & Rhodes, O. E. (2017). Effect of compensatory immigration on the genetic structure of coyotes. Journal of Wildlife Management, 81, 1394–1407. doi: 10.1002/jwmg.21320

403. Kikko, T., Kai, Y., Kuwahara, M., & Nakayama, K. (2008). Genetic diversity and population structure of white-spotted charr, Salvelinus leucomaenis, in the Lake Biwa water system inferred from AFLP analysis. Ichthyological Research, 55, 141–147. doi: 10.1007/s10228-007-0017-y

404. Kim, E. S., & Kirkpatrick, B. W. (2009). Linkage disequilibrium in the north American holstein population. Animal Genetics, 40, 279–288. doi: 10.1111/j.1365-2052.2008.01831.x

405. Kim, H., Hoelmer, K. A., & Lee, S. (2017). Population genetics of the soybean aphid in North America and East Asia: test for introduction between native and introduced populations. Biological Invasions, 19, 597–614. doi: 10.1007/s10530-016-1299-7

406. Kim, J. H., Kang, J. H., Jang, J. E., Choi, S. K., Kim, M. J., Park, S. R., & Lee, H. J. (2017). Population genetic structure of eelgrass (Zostera marina) on the Korean coast: Current status and conservation implications for future management. PLoS ONE, 12, e0174105. doi: 10.1371/journal.pone.0174105

407. Kinnison, M. T., Bentzen, P., Unwin, M. J., & Quinn, T. P. (2002). Reconstructing recent divergence: Evaluating nonequilibrium population structure in New Zealand chinook salmon. Molecular Ecology, 11, 739–754. doi: 10.1046/j.1365-294X.2002.01477.x

408. Kitanishi, S., Ikeda, T., & Yamamoto, T. (2017). Short-term temporal instability in fine-scale genetic structure of masu salmon. Freshwater Biology, 62, 1655–1664. doi: 10.1111/fwb.12978

409. Kittelson, P. M., Pinahs, C., Dwyer, J., Ingersoll, A., Mans, E., Rieke, J., … Volenec, M. (2009). Age structure and genetic diversity of four Quercus macrocarpa (michx.) populations in fragmented oak savanna along the central Minnesota River Valley. The American Midland Naturalist, 161, 301–312. doi: 10.1674/0003-0031-161.2.301

410. Klaedtke, S. M., Caproni, L., Klauck, J., de La Grandville, P., Dutartre, M., Stassart, P. M., … Raggi, L. (2017). Short-term local adaptation of historical common bean (Phaseolus vulgaris L.) varieties and implications for in situ management of bean diversity. International Journal of Molecular Sciences, 18, 1–19. doi: 10.3390/ijms18030493

411. Klauke, N., Schaefer, H. M., Bauer, M., & Segelbacher, G. (2016). Limited dispersal and significant fine - Scale genetic structure in a tropical montane parrot species. PLoS ONE, 11, e0169165. doi: 10.1371/journal.pone.0169165

412. Klein, M., Teixeira, S., Assis, J., Serrão, E. A., Gonçalves, E. J., & Borges, R. (2016). High interannual variability in connectivity and genetic pool of a temperate clingfish matches oceanographic transport predictions. PLoS ONE, 11, e0165881. doi: 10.1371/journal.pone.0165881

413. Knafler, G. J., Ortiz-Catedral, L., Jackson, B., Varsani, A., Grueber, C. E., Robertson, B. C., & Jamieson, I. G. (2016). Comparison of beak and feather disease virus prevalence and immunity-associated genetic diversity over time in an island population of red-crowned parakeets. Archives of Virology, 161, 811–820. doi: 10.1007/s00705-015-2717-3

414. Knight, N. L., Vaghefi, N., Hansen, Z. R., Kikkert, J. R., & Pethybridge, S. J. (2018). Temporal genetic differentiation of Cercospora beticola populations in New York table beet fields. Plant Disease, 102, 2074–2082. doi: 10.1094/PDIS-01-18-0175-RE

415. Kogura, Y., Seeb, J. E., Azuma, N., Kudo, H., Abe, S., & Kaeriyama, M. (2011). The genetic population structure of lacustrine sockeye salmon, Oncorhynchus nerka, in Japan as the endangered species. Environmental Biology of Fishes, 92, 539–550. doi: 10.1007/s10641-011-9876-1

416. Kohyama, T. I., Omote, K., Nishida, C., Takenaka, T., Saito, K., Fujimoto, S., & Masuda, R. (2015). Spatial and temporal variation at major histocompatibility complex class IIB genes in the endangered Blakiston’s fish owl. Zoological Letters, 1, 1–12. doi: 10.1186/s40851-015-0013-4

417. Kollars, P. G., Beck, M. L., Mech, S. G., Kennedy, P. K., & Kennedy, M. L. (2004). Temporal and spatial genetic variability in white-tailed deer (Odocoileus virginianus). Genetica, 121, 269–276. doi: 10.1023/B:GENE.0000039851.27973.68

418. Kopatz, A., Eiken, H. G., Schregel, J., Aspi, J., Kojola, I., & Hagen, S. B. (2017). Genetic substructure and admixture as important factors in linkage disequilibrium-based estimation of effective number of breeders in recovering wildlife populations. Ecology and Evolution, 7, 10721–10732. doi: 10.1002/ece3.3577

419. Korstian, J. M., Hale, A. M., & Williams, D. A. (2015). Genetic Diversity, Historic Population Size, and Population Structure in 2 North American Tree Bats. Journal of Mammalogy, 96, 972–980. doi: 10.1093/jmammal/gyv101

420. Koskinen, M. T., Sundell, P., Piironen, J., & Primmer, C. R. (2002). Genetic assessment of spatiotemporal evolutionary relationships and stocking effects in grayling (Thymallus thymallus, Salmonidae). Ecology Letters, 5, 193–205. doi: 10.1046/j.1461-0248.2002.00302.x

421. Kostow, K. E., Marshall, A. R., & Phelps, S. R. (2003). Naturally Spawning Hatchery Steelhead Contribute to Smolt Production but Experience Low Reproductive Success. Transactions of the American Fisheries Society, 132, 780–790. doi: 10.1577/t02-051

422. Kotoulas, G., Bonhomme, F., & Borsa, P. (1995). Genetic structure of the common sole Solea vulgaris at different geographic scales. Marine Biology, 122, 361–375. doi: 10.1007/BF00350869

423. Kovach, R. P., Gharrett, A. J., & Tallmon, D. A. (2012). Genetic change for earlier migration timing in a pink salmon population. Proceedings of the Royal Society B, 279, 3870–3878. doi: 10.1098/rspb.2012.1158

424. Krehenwinkel, H., & Pekar, S. (2015). An analysis of factors affecting genotyping success from museum specimens reveals an increase of genetic and morphological variation during a historical range expansion of a European spider. PLoS ONE, 10, e0136337. doi: 10.1371/journal.pone.0136337

425. Kruckenhauser, L., Rauer, G., Däubl, B., & Haring, E. (2009). Genetic monitoring of a founder population of brown bears (Ursus arctos) in central Austria. Conservation Genetics, 10, 1223–1233. doi: 10.1007/s10592-008-9654-6

426. Kubota, H., & Watanabe, K. (2003). Genetic diversity in wild and reared populations of the Japanese bitterling Tanakia tanago (Cyprinidae). Ichthyological Research, 50, 123–128. doi: 10.1007/s10228-002-0146-2

427. Kubota, H., Watanabe, K., Kakehi, Y., & Watanabe, S. (2008). An assessment of genetic diversity in wild and captive populations of endangered Japanese bitterling Tanakia tanago (Cyprinidae) using amplified fragment length polymorphism (AFLP) markers. Fisheries Science, 74, 494–502. doi: 10.1111/j.1444-2906.2008.01551.x

428. Kubota, H., Watanabe, K., Suguro, N., Tabe, M., Umezawa, K., & Watanabe, S. (2010). Genetic population structure and management units of the endangered Tokyo bitterling, Tanakia tanago (Cyprinidae). Conservation Genetics, 11, 2343–2355. doi: 10.1007/s10592-010-0120-x

429. Kume, M., Mori, S., Kitano, J., Sumi, T., & Nishida, S. (2018). Impact of the huge 2011 Tohoku-oki tsunami on the phenotypes and genotypes of Japanese coastal threespine stickleback populations. Scientific Reports, 8, 1–11. doi: 10.1038/s41598-018-20075-z

430. Kusakabe, S., Yamaguchi, Y., Baba, H., & Mukai, T. (2000). The genetic structure of the Raleigh natural population of Drosophila melanogaster revisited. Genetics, 154, 679–685. doi: 10.1093/genetics/154.2.679

431. Kuster, C. J., Schwarzenberger, A., & von Elert, E. (2013). Seasonal dynamics of sestonic protease inhibition: Impact on Daphnia populations. Hydrobiologia, 715, 37–50. doi: 10.1007/s10750-012-1303-x

432. Kvistad, L., Ingwersen, D., Pavlova, A., Bull, J. K., & Sunnucks, P. (2015). Very low population structure in a highly mobile and wide-ranging endangered bird species. PLoS ONE, 10, e0143746. doi: 10.1371/journal.pone.0143746

433. Lachish, S., Miller, K. J., Storfer, A., Goldizen, A. W., & Jones, M. E. (2011). Evidence that disease-induced population decline changes genetic structure and alters dispersal patterns in the Tasmanian devil. Heredity, 106, 172–182. doi: 10.1038/hdy.2010.17

434. Lacson, J. M., & Morizot, D. C. (1991). Temporal genetic variation in subpopulations of bicolor damselfish (Stegastes partitus) inhabiting coral reefs in the Florida Keys. *Marine Biology*, *110*, 353–357. doi: 10.1007/BF01344354

435. Lado-Insua, T., Pérez, M., Diz, A. P., & Presa, P. (2011). Temporal estimates of genetic diversity in some Mytilus galloprovincialis populations impacted by the Prestige oil-spill. Continental Shelf Research, 31, 466–475. doi: 10.1016/j.csr.2010.06.008

436. Lage, C., & Kornfield, I. (2006). Reduced genetic diversity and effective population size in an endangered Atlantic salmon (Salmo Salar) population from Maine, USA. Conservation Genetics, 7, 91–104. doi: 10.1007/s10592-005-8669-5

437. Lainhart, W., Bickersmith, S. A., Moreno, M., Rios, C. T., Vinetz, J. M., & Conn, J. E. (2015). Changes in genetic diversity from field to laboratory during colonization of anopheles darlingi root (Diptera: Culicidae). American Journal of Tropical Medicine and Hygiene, 93, 998–1001. doi: 10.4269/ajtmh.15-0336

438. Lampert, K. P., Regmi, B. P., Wathne, I., & Larsson, P. (2014). Clonal diversity and turnover in an overwintering Daphnia pulex population, and the effect of fish predation. Freshwater Biology, 59, 1735–1743. doi: 10.1111/fwb.12378

439. Larsson, J. K., Jansman, H. A. H., Segelbacher, G., Höglund, J., & Koelewijn, H. P. (2008). Genetic impoverishment of the last black grouse (Tetrao tetrix) population in the Netherlands: Detectable only with a reference from the past. Molecular Ecology, 17, 1897–1904. doi: 10.1111/j.1365-294X.2008.03717.x

440. Larsson, L. C., Laikre, L., André, C., Dahlgren, T. G., & Ryman, N. (2010). Temporally stable genetic structure of heavily exploited Atlantic herring (Clupea harengus) in Swedish waters. Heredity, 104, 40–51. doi: 10.1038/hdy.2009.98

441. Laurent, V., & Planes, S. (2007). Effective population size estimation on Sardina pilchardus in the Bay of Biscay using a temporal genetic approach. Biological Journal of the Linnean Society, 90, 591–602. doi: 10.1111/j.1095-8312.2007.00747.x

442. Lawrence, H. A., Scofield, R. P., Crockett, D. E., Millar, C. D., & Lambert, D. M. (2008). Ancient genetic variation in one of the world's rarest seabirds. Heredity, 101, 543–547. doi: 10.1038/hdy.2008.85

443. Lawson, L. P., Fessl, B., Hernán Vargas, F., Farrington, H. L., Francesca Cunninghame, H., Mueller, J. C., … Petren, K. (2017). Slow motion extinction: inbreeding, introgression, and loss in the critically endangered mangrove finch (Camarhynchus heliobates). Conservation Genetics, 18, 159–170. doi: 10.1007/s10592-016-0890-x

444. Le Clerc, V., Bazante, F., Baril, C., Guiard, J., & Zhang, D. (2005). Assessing temporal changes in genetic diversity of maize varieties using microsatellite markers. Theoretical and Applied Genetics, 110, 294–302. doi: 10.1007/s00122-004-1834-2

445. Le Clerc, V., Cadot, V., Canadas, M., Lallemand, J., Guèrin, D., & Boulineau, F. (2006). Indicators to assess temporal genetic diversity in the French Catalogue: No losses for maize and peas. Theoretical and Applied Genetics, 113, 1197–1209. doi: 10.1007/s00122-006-0368-1

446. le Gouar, P. J., Vallet, D., David, L., Bermejo, M., Gatti, S., Levréro, F., … Ménard, N. (2009). How Ebola impacts genetics of western lowland gorilla populations. PLoS ONE, 4, e8375. doi: 10.1371/journal.pone.0008375

447. Leblois, R., Gautier, M., Rohfritsch, A., Foucaud, J., Burban, C., Galan, M., … Kerdelhué, C. (2018). Deciphering the demographic history of allochronic differentiation in the pine processionary moth Thaumetopoea pityocampa. Molecular Ecology, 27, 264–278. doi: 10.1111/mec.14411

448. Lee, H. J., & Boulding, E. G. (2007). Mitochondrial DNA variation in space and time in the northeastern Pacific gastropod, Littorina keenae. Molecular Ecology, 16, 3084–3103. doi: 10.1111/j.1365-294X.2007.03364.x

449. Lee, S. H., Cho, Y. M., Lim, D., Kim, H. C., Choi, B. H., Park, H. S., … Hong, S. K. (2011). Linkage disequilibrium and effective population size in Hanwoo Korean cattle. Asian-Australasian Journal of Animal Sciences, 24, 1660–1665. doi: 10.5713/ajas.2011.11165

450. Lefèvre, F., Fady, B., Fallour-Rubio, D., Ghosn, D., & Bariteau, M. (2004). Impact of founder population, drift and selection on the genetic diversity of a recently translocated tree population. Heredity, 93, 542–550. doi: 10.1038/sj.hdy.6800549

451. Lehmann, T., Hawley, W. A., Grebert, H., & Collins, F. H. (1998). The effective population size of Anopheles gambiae in Kenya: implications for population structure. Molecular Biology and Evolution, 15, 264–276. doi: 10.1093/oxfordjournals.molbev.a025923

452. Lemic, D., Mikac, K. M., Ivkosic, S. A., & Bažok, R. (2015). The temporal and spatial invasion genetics of the western corn rootworm (Coleoptera: Chrysomelidae) in southern Europe. PLoS ONE, 10, e0138796. doi: 10.1371/journal.pone.0138796

453. Levy, F., & Neal, C. L. (1999). Spatial and temporal genetic structure in chloroplast and allozyme markers in Phacelia dubia implicate genetic drift. Heredity, 82, 422–431. doi: 10.1038/sj.hdy.6885000

454. Li, Y., & Kim, J. J. (2015). Effective population size and signatures of selection using bovine 50K SNP chips in Korean native cattle (Hanwoo). *Evolutionary Bioinformatics*, *11*, 143–153. doi: 10.4137/EBO.S24359

455. Liang, J. M., Xayamongkhon, H., Broz, K., Dong, Y., McCormick, S. P., Abramova, S., … Kistler, H. C. (2014). Temporal dynamics and population genetic structure of Fusarium graminearum in the upper Midwestern United States. *Fungal Genetics and Biology*, *73*, 83–92. doi: 10.1016/j.fgb.2014.10.002

456. Limburg, P. A., & Weider, L. J. (2002). "Ancient" DNA in the resting egg bank of a microcrustacean can serve as a palaeolimnological database. Proceedings of the Royal Society B, 269, 281–287. doi: 10.1098/rspb.2001.1868

457. Lin, H., Fan, X., Zhou, X., & Gao, J. (2012). Self-interference is reduced in a secondary pollen presentation species, Duperrea pavettifolia (Rubiaceae). Flora, 207, 895–902. doi: 10.1016/j.flora.2012.10.001

458. Lin, W., Karczmarski, L., Xia, J., Zhang, X., Yu, X., & Wu, Y. (2016). Increased human occupation and agricultural development accelerates the population contraction of an estuarine delphinid. Scientific Reports, 6, 1–12. doi: 10.1038/srep35713

459. Lion, M. B., Eizirik, E., Garda, A. A., da Fontoura-Rodrigues, M. L., Rodrigues, F. H. G., & Marinho-Filho, J. S. (2011). Conservation genetics of maned wolves in a highly impacted area of the Brazilian Cerrado biome. Genetica, 139, 369–381. doi: 10.1007/s10709-011-9555-3

460. Liu, I. A., López-Ortiz, R., Ramos-Álvarez, K., & Medina-Miranda, R. (2018). Using population genetics and demographic reconstruction to predict outcomes of genetic rescue for an endangered songbird. Conservation Genetics, 19, 729–736. doi: 10.1007/s10592-018-1050-2

461. Liu, J. X., & Ely, B. (2009). Sibship reconstruction demonstrates the extremely low effective population size of striped bass Morone saxatilis in the Santee-Cooper system, South Carolina, USA. Molecular Ecology, 18, 4112–4120. doi: 10.1111/j.1365-294X.2009.04343.x

462. Liu, Y., Keller, I., & Heckel, G. (2013). Temporal genetic structure and relatedness in the Tufted Duck Aythya fuligula suggests limited kin association in winter. Ibis, 155, 499–507. doi: 10.1111/ibi.12059

463. Lobo, J. A., Jimenez, D., Solis-Hernandez, W., & Fuchs, E. J. (2015). Lack of early inbreeding depression and distribution of selfing rates in the neotropical emergent tree Ceiba pentandra: Assessment from several reproductive events. American Journal of Botany, 102, 983–991. doi: 10.3732/ajb.1400520

464. Lombal, A. J., Wenner, T. J., Carlile, N., Austin, J. J., Woehler, E., Priddel, D., & Burridge, C. P. (2017). Population genetic and behavioural variation of the two remaining colonies of Providence petrel (Pterodroma solandri). Conservation Genetics, 18, 117–129. doi: 10.1007/s10592-016-0887-5

465. Lonsinger, R. C., Adams, J. R., & Waits, L. P. (2018). Evaluating effective population size and genetic diversity of a declining kit fox population using contemporary and historical specimens. Ecology and Evolution, 8, 12011–12021. doi: 10.1002/ece3.4660

466. López, A., Vera, M., Planas, M., & Bouza, C. (2015). Conservation genetics of threatened hippocampus guttulatus in vulnerable habitats in NW Spain: Temporal and spatial stability of wild populations with flexible polygamous mating system in captivity. PLoS ONE, 10, e0117538. doi: 10.1371/journal.pone.0117538

467. Louette, G., Vanoverbeke, J., Ortells, R., & De Meester, L. (2007). The founding mothers: the genetic structure of newly established Daphnia populations. Oikos, 116, 728–741. doi: 10.1111/j.2007.0030-1299.15664.x

468. Lounsberry, Z. T., Almeida, J. B., Lanctot, R. B., Liebezeit, J. R., Sandercock, B. K., Strum, K. M., … Wisely, S. M. (2014). Museum collections reveal that Buff-breasted Sandpipers (Calidris subruficollis) maintained mtDNA variability despite large population declines during the past 135 years. Conservation Genetics, 15, 1197–1208. doi: 10.1007/s10592-014-0611-2

469. Low, G. W., Chattopadhyay, B., Garg, K. M., Irestedt, M., Ericson, P. G. P., Yap, G., … Rheindt, F. E. (2018). Urban landscape genomics identifies fine-scale gene flow patterns in an avian invasive. Heredity, 120, 138–153. doi: 10.1038/s41437-017-0026-1

470. Loxdale, H. D., Tarr, I. J., Weber, C. P., Brookes, C. P., Digby, P. G. N., & Castañera, P. (1985). Electrophoretic study of enzymes from cereal aphid populations. III. Spatial and temporal genetic variation of populations of Sitobion avenae (F.) (Hemiptera: Aphididae). Bulletin of Entomological Research, 75, 121–141. doi: 10.1017/S0007485300014218

471. Lozier, J. D., & Cameron, S. A. (2009). Comparative genetic analyses of historical and contemporary collections highlight contrasting demographic histories for the bumble bees Bombus pensylvanicus and B. impatiens in Illinois. Molecular Ecology, 18, 1875–1886. doi: 10.1111/j.1365-294X.2009.04160.x

472. Lucentini, L., Palomba, A., Gigliarelli, L., Sgaravizzi, G., Lancioni, H., Lanfaloni, L., … Panara, F. (2009). Temporal changes and effective population size of an Italian isolated and supportive-breeding managed northern pike (Esox lucius) population. Fisheries Research, 96, 139–147. doi: 10.1016/j.fishres.2008.10.007

473. Lukindu, M., Bergey, C. M., Wiltshire, R. M., Small, S. T., Bourke, B. P., Kayondo, J. K., & Besansky, N. J. (2018). Spatio-temporal genetic structure of Anopheles gambiae in the Northwestern Lake Victoria Basin, Uganda: Implications for genetic control trials in malaria endemic regions. Parasites and Vectors, 11, 1–12. doi: 10.1186/s13071-018-2826-4

474. Lundy, C. J., Rico, C., & Hewitt, G. M. (2000). Temporal and spatial genetic variation in spawning grounds of European hake (Merluccius merluccius) in the Bay of Biscay. Molecular Ecology, 9, 2067–2079. doi: 10.1046/j.1365-294X.2000.01120.x

475. Lyalina, E. V, Boldyrev, S. V, & Pomortsev, A. A. (2016). Current state of the genetic polymorphism in spring barley (Hordeum vulgare L.) from Russia assessed by the alleles of hordein-coding loci. Russian Journal of Genetics, 52, 565–577. doi: 10.1134/S1022795416060077

476. MacLeod, I. M., Larkin, D. M., Lewin, H. A., Hayes, B. J., & Goddard, M. E. (2013). Inferring demography from runs of homozygosity in whole-genome sequence, with correction for sequence errors. Molecular Biology and Evolution, 30, 2209–2223. doi: 10.1093/molbev/mst125

477. Madsen, C. L., Vilstrup, J. T., Fernández, R., Marchi, N., Hakansson, B., Krog, M., … Orlando, L. (2015). Mitochondrial genetic diversity of eurasian red squirrels (Sciurus vulgaris) from Denmark. Heredity, 106, 719–727. doi: 10.1093/jhered/esv074

478. Maebe, K., Golsteyn, L., Nunes-Silva, P., Blochtein, B., & Smagghe, G. (2018). Temporal changes in genetic variability in three bumblebee species from Rio Grande do Sul, South Brazil. Apidologie, 49, 415–429. doi: 10.1007/s13592-018-0567-1

479. Maebe, K., Meeus, I., Maharramov, J., Grootaert, P., Michez, D., Rasmont, P., & Smagghe, G. (2013). Microsatellite analysis in museum samples reveals inbreeding before the regression of Bombus veteranus. Apidologie, 44, 188–197. doi: 10.1007/s13592-012-0170-9

480. Maes, G. E., Pujolar, J. M., Hellemans, B., & Volckaert, F. A. M. (2006). Evidence for isolation by time in the European eel (Anguilla anguilla L.). Molecular Ecology, 15, 2095–2107. doi: 10.1111/j.1365-294X.2006.02925.x

481. Magsino, R. M., & Juinio-Meñez, M. A. (2008). The influence of contrasting life history traits and oceanic processes on genetic structuring of rabbitfish populations Siganus argenteus and Siganus fuscescens along the eastern Philippine coasts. Marine Biology, 154, 519–532. doi: 10.1007/s00227-008-0946-7

482. Maliti, D., Ranson, H., Magesa, S., Kisinza, W., Mcha, J., Haji, K., … Weetman, D. (2014). Islands and stepping-stones: Comparative population structure of Anopheles gambiae sensu stricto and Anopheles arabiensis in Tanzania and implications for the spread of insecticide resistance. PLoS ONE, 9, e110910. doi: 10.1371/journal.pone.0110910

483. Maltagliati, F., & Camilli, L. (2000). Temporal genetic variation in a population of Aphanius fasciatus (Cyprinodontidae) from a brackish-water habitat at Elba Island (Italy). Environmental Biology of Fishes, 57, 107–112. doi: 10.1023/A:1007604831242

484. Malysheva-Otto, L., Ganal, M. W., Law, J. R., Reeves, J. C., & Röder, M. S. (2007). Temporal trends of genetic diversity in European barley cultivars (Hordeum vulgare L.). Molecular Breeding, 20, 309–322. doi: 10.1007/s11032-007-9093-y

485. Mamidi, S., Rossi, M., Annam, D., Moghaddam, S., Lee, R., Papa, R., & McClean, P. (2011). Investigation of the domestication of common bean (Phaseolus vulgaris) using multilocus sequence data. Functional Plant Biology, 38, 953–967. doi: 10.1071/FP11124

486. Mandák, B., & Plačková, I. (2009). How does population genetic diversity change over time? An experimental seed bank study of Atriplex tatarica (Chenopodiaceae). Flora, 204, 423–433. doi: 10.1016/j.flora.2008.05.005

487. Manifesto, M. M., Schlatter, A. R., Hopp, H. E., Suárez, E. Y., & Dubcovsky, J. (2001). Quantitative evaluation of genetic diversity in wheat germplasm using molecular markers. Crop Science, 41, 682–690. doi: doi.org/10.2135/cropsci2001.413682x

488. Mank, J. E., Carlson, J. E., & Brittingham, M. C. (2004). A century of hybridization: Decreasing genetic distance between American black ducks and mallards. Conservation Genetics, 5, 395–403. doi: 10.1023/B:COGE.0000031139.55389.b1

489. Maretto, F., & Cassandro, M. (2014). Temporal variation in genetic diversity and population structure of Burlina cattle breed. Italian Journal of Animal Science, 13, 322–329. doi: 10.4081/ijas.2014.3091

490. Mariette, N., Mabon, R., Corbière, R., Boulard, F., Glais, I., Marquer, B., … Andrivon, D. (2016). Phenotypic and genotypic changes in French populations of Phytophthora infestans: Are invasive clones the most aggressive? Plant Pathology, 65, 577–586. doi: 10.1111/ppa.12441

491. Marquez, J. G., Moon, R. D., & Krafsur, E. S. (2001). Genetic differentiation among populations of house flies (Diptera: Muscidae) breeding at a multiple-barn, egg-laying facility in Central Minnesota. Journal of Medical Entomology, 38, 218–222. doi: 10.1603/0022-2585-38.2.218

492. Marranca, J. M., Welsh, A. B., & Roseman, E. (2015). Genetic effects of habitat restoration in the Laurentian Great Lakes: An assessment of lake sturgeon origin and genetic diversity. Restoration Ecology, 23, 455–464. doi: 10.1111/rec.12200

493. Marsden, C. D., Wayne, R. K., & Mable, B. K. (2012). Inferring the ancestry of African wild dogs that returned to the Serengeti-Mara. Conservation Genetics, 13, 525–533. doi: 10.1007/s10592-011-0304-z

494. Marsden, C. D., Woodroffe, R., Mills, M. G. L., McNutt, J. W., Creel, S., Groom, R., … Mable, B. K. (2012). Spatial and temporal patterns of neutral and adaptive genetic variation in the endangered African wild dog (Lycaon pictus). Molecular Ecology, 21, 1379–1393. doi: 10.1111/j.1365-294X.2012.05477.x

495. Martin, A. P. (2010). The conservation genetics of Ash Meadows pupfish populations. I. The Warm Springs pupfish Cyprinodon nevadensis pectoralis. Conservation Genetics, 11, 1847–1857. doi: 10.1007/s10592-010-0077-9

496. Martin, M. D., Zimmer, E. A., Olsen, M. T., Foote, A. D., Gilbert, M. T. P., & Brush, G. S. (2014). Herbarium specimens reveal a historical shift in phylogeographic structure of common ragweed during native range disturbance. Molecular Ecology, 23, 1701–1716. doi: 10.1111/mec.12675

497. Martínez-Cruz, B., Godoy, J. A., & Negro, J. J. (2007). Population fragmentation leads to spatial and temporal genetic structure in the endangered Spanish imperial eagle. Molecular Ecology, 16, 477–486. doi: 10.1111/j.1365-294X.2007.03147.x

498. Martínez, J. G., Mira, Ó., Sánchez-Prieto, C. B., Barea-Azcón, J. M., & Tinaut, A. (2018). Population size and genetic variability of a relict population of an endangered butterfly, Parnassius apollo filabricus. Insect Conservation and Diversity, 11, 294–304. doi: 10.1111/icad.12276

499. Martínez, P., González, E. G., Castilho, R., & Zardoya, R. (2006). Genetic diversity and historical demography of Atlantic bigeye tuna (Thunnus obesus). Molecular Phylogenetics and Evolution, 39, 404–416. doi: 10.1016/j.ympev.2005.07.022

500. Massonnet, B., Simon, J. C., & Weisser, W. W. (2002). Metapopulation structure of the specialized herbivore Macrosiphoniella tanacetaria (Homoptera, Aphididae). Molecular Ecology, 11, 2511–2521. doi: 10.1046/j.1365-294X.2002.01633.x

501. Mátics, R., Hoffmann, G., Farkas, S., Dawson, D., Frantz, A., Varga, D., … Klein, Á. (2017). Demographic decline and detection of genetic bottleneck in a population of Barn Owl Tyto alba in Hungary. Journal of Ornithology, 158, 811–821. doi: 10.1007/s10336-017-1433-z

502. Matocq, M. D., & Villablanca, F. X. (2001). Low genetic diversity in an endangered species: Recent or historic pattern? Biological Conservation, 98, 61–68. doi: 10.1016/S0006-3207(00)00142-7

503. Mattiucci, S., Cipriani, P., Paoletti, M., Nardi, V., Santoro, M., Bellisario, B., & Nascetti, G. (2015). Temporal stability of parasite distribution and genetic variability values of Contracaecum osculatum sp. D and C. osculatum sp. E (Nematoda: Anisakidae) from fish of the Ross Sea (Antarctica). International Journal for Parasitology: Parasites and Wildlife, 4, 356–367. doi: 10.1016/j.ijppaw.2015.10.004

504. Maynard, A. J., Ambrose, L., Cooper, R. D., Chow, W. K., Davis, J. B., Muzari, M. O., … Beebe, N. W. (2017). Tiger on the prowl: Invasion history and spatio-temporal genetic structure of the Asian tiger mosquito Aedes albopictus (Skuse 1894) in the Indo-Pacific. PLoS Neglected Tropical Diseases, 11, e0005546. doi: 10.1371/journal.pntd.0005546

505. McBride, M. C., Hasselman, D. J., Willis, T. V, Palkovacs, E. P., & Bentzen, P. (2015). Influence of stocking history on the population genetic structure of anadromous alewife (Alosa pseudoharengus) in Maine rivers. Conservation Genetics, 16, 1209–1223. doi: 10.1007/s10592-015-0733-1

506. McCairns, R. J. S., Kuparinen, A., Panda, B., Jokikokko, E., & Merilä, J. (2012). Effective size and genetic composition of two exploited, migratory whitefish (Coregonus lavaretus lavaretus) populations. Conservation Genetics, 13, 1509–1520. doi: 10.1007/s10592-012-0394-2

507. McCusker, M. R., & Bentzen, P. (2010). Historical influences dominate the population genetic structure of a sedentary marine fish, Atlantic wolffish (Anarhichas lupus), across the North Atlantic Ocean. Molecular Ecology, 19, 4228–4241. doi: 10.1111/j.1365-294X.2010.04806.x

508. McCusker, M. R., Mandrak, N. E., Egeh, B., & Lovejoy, N. R. (2014). Population structure and conservation genetic assessment of the endangered Pugnose Shiner, Notropis anogenus. Conservation Genetics, 15, 343–353. doi: 10.1007/s10592-013-0542-3

509. McDowell, J. R., & Graves, J. E. (2008). Population structure of striped marlin (Kajikia audax) in the Pacific Ocean based on analysis of microsatellite and mitochondrial DNA. Canadian Journal of Fisheries and Aquatic Sciences, 65, 1307–1320. doi: 10.1139/F08-054

510. McEachern, M. B., Eadie, J. M., Van Vuren, D. H., & Ecology Graduate Group, . (2007). Local genetic structure and relatedness in a solitary mammal, Neotoma fuscipes. Behavioral Ecology and Sociobiology, 61, 1459–1469. doi: 10.1007/s00265-007-0378-2

511. McFarlane, S., Manseau, M., Flasko, A., Horn, R. L., Arnason, N., Neufeld, L., … Wilson, P. (2018). Genetic influences on male and female variance in reproductive success and implications for the recovery of severely endangered mountain caribou. *Global Ecology and Conservation*, *16*, e00451. doi: 10.1016/j.gecco.2018.e00451

512. McLean, J. E., Hay, D. E., & Taylor, E. B. (1999). Marine population structure in an anadromous fish: Life-history influences patterns of mitochondrial DNA variation in the eulachon, Thaleichthys pacificus. Molecular Ecology, 8, S143–S158. doi: 10.1046/j.1365-294x.1999.00818.x

513. McLean, J. E., Seamons, T. R., Dauer, M. B., Bentzen, P., & Quinn, T. P. (2008). Variation in reproductive success and effective number of breeders in a hatchery population of steelhead trout (Oncorhynchus mykiss): Examination by microsatellite-based parentage analysis. Conservation Genetics, 9, 295–304. doi: 10.1007/s10592-007-9340-0

514. Meglécz, E., Pecsenye, K., Varga, Z., & Solignac, M. (1998). Comparison of differentiation pattern at allozyme and microsatellite loci in Parnassius mnemosyne (Lepidoptera) populations. Hereditas, 128, 95–103. doi: 10.1111/j.1601-5223.1998.00095.x

515. Mehmood, Y., Sambasivam, P., Kaur, S., Davidson, J., Leo, A. E., Hobson, K., … Ford, R. (2017). Evidence and consequence of a highly adapted clonal haplotype within the australian ascochyta rabiei population. Frontiers in Plant Science, 8, 1–11. doi: 10.3389/fpls.2017.01029

516. Meldgaard, T., Nielsen, E. E., & Loeschcke, V. (2003). Fragmentation by weirs in a riverine system: A study of genetic variation in time and space among populations of European grayling (Thymallus thymallus) in a Danish river system. Conservation Genetics, 4, 735–747. doi: 10.1023/B:COGE.0000006115.14106.de

517. Mendonça, B. A. A., de Sousa, A. C. B., de Souza, A. P., & Scarpassa, V. M. (2014). Temporal genetic structure of major dengue vector Aedes aegypti from Manaus, Amazonas, Brazil. Acta Tropica, 134, 80–88. doi: 10.1016/j.actatropica.2014.02.014

518. Menéndez, J., Goyache, F., Beja-Pereira, A., Fernández, I., Menéndez-Arias, N. A., Godinho, R., & Álvarez, I. (2016). Genetic characterisation of the endangered Gochu Asturcelta pig breed using microsatellite and mitochondrial markers: Insights for the composition of the Iberian native pig stock. *Livestock Science, 187,* 162–167. doi: 10.1016/j.livsci.2016.03.013

519. Meunier, C., Hurtrez-Bousses, S., Durand, P., Rondelaud, D., & Renaud, F. (2004). Small effective population sizes in a widespread selfing species, Lymnaea truncatula (Gastropoda: Pulmonata). Molecular Ecology, 13, 2535–2543. doi: 10.1111/j.1365-294X.2004.02242.x

520. Mhemmed, G., Kamel, H., & Chedly, A. (2008). Does habitat fragmentation reduce genetic diversity and subpopulation connectivity? Ecography, 31, 751–756. doi: 10.1111/j.1600-0587.2008.05622.x

521. Michel, A. P., Grushko, O., Guelbeogo, W. M., Sagnon, N., Costantini, C., & Besansky, N. J. (2006). Effective population size of Anopheles funestus chromosomal forms in Burkina Faso. *Malaria Journal, 5,* 1–6. doi: 10.1186/1475-2875-5-115

522. Milla-Lewis, S. R., Zuleta, M. C., & Isleib, T. G. (2010). Assessment of genetic diversity among U.S. runner-type peanut cultivars using simple sequence repeat markers. Crop Science, 50, 2396–2405. doi: 10.2135/cropsci2010.04.0223

523. Miller, C. R., & Waits, L. P. (2003). The history of effective population size and genetic diversity in the Yellowstone grizzly (Ursus arctos): Implications for conservation. Proceedings of the National Academy of Sciences of the United States of America, 100, 4334–4339. doi: 10.1073/pnas.0735531100

524. Miller, L. M., & Kapuscinski, A. R. (1997). Historical analysis of genetic variation reveals low effective population size in a Northern pike (Esox lucius) population. Genetics, 147, 1249–1258. doi: 10.1093/genetics/147.3.1249

525. Milton, K., Lozier, J. D., & Lacey, E. A. (2009). Genetic structure of an isolated population of mantled howler monkeys (Alouatta palliata) on Barro Colorado Island, Panama. Conservation Genetics, 10, 347–358. doi: 10.1007/s10592-008-9584-3

526. Minhós, T., Chikhi, L., Sousa, C., Vicente, L. M., Ferreira da Silva, M., Heller, R., … Bruford, M. W. (2016). Genetic consequences of human forest exploitation in two colobus monkeys in Guinea Bissau. *Biological Conservation*, *194*, 194–208. doi: 10.1016/j.biocon.2015.12.019

527. Miño, C. I., & Del Lama, S. N. (2014). Genetic differentiation among populations of the Roseate spoonbill (Platalea ajaja; Aves: Pelecaniformes) in three Brazilian wetlands. Biochemical Genetics, 52, 321–337. doi: 10.1007/s10528-014-9650-0

528. Mishra, P. K., Tewari, J. P., Turkington, T. K., & Clear, R. M. (2009). Genetic evidence for a recent geographic expansion of 15- acetyldeoxynivalenol chemotypes of Fusarium graminearum in Canada. Canadian Journal of Plant Pathology, 31, 468–474. doi: 10.1080/07060660909507621

529. Mitrovski, P., Hoffmann, A. A., Heinze, D. A., & Weeks, A. R. (2008). Rapid loss of genetic variation in an endangered possum. Biology Letters, 4, 134–138. doi: 10.1098/rsbl.2007.0454

530. Miura, O., Kanaya, G., Nakai, S., Itoh, H., Chiba, S., Makino, W., … Urabe, J. (2017). Ecological and genetic impact of the 2011 Tohoku Earthquake Tsunami on intertidal mud snails. Scientific Reports, 7, 1–10. doi: 10.1038/srep44375

531. Miyahara, H., Yamada, H., Sato, T., Harada, Y., & Kawamura, K. (2014). Genetic evaluation of reintroduced amago salmon Oncorhynchus masou ishikawae. Nippon Suisan Gakkaishi (Japanese Edition), 80, 233–240. doi: 10.2331/suisan.80.233

532. Mladineo, I., Tomas, M., & Stanic, R. (2015). Cross-fertilization as a reproductive strategy in a tissue flukes Didymosulcus katsuwonicola (Platyhelmintes: Didymozoidae) inferred by genetic analysis. Parasitology, 142, 1422–1429. doi: 10.1017/S0031182015000839

533. Modorov, M. V, & Pozolotina, V. N. (2011). Allozyme variation of the pygmy wood mouse Apodemus uralensis (Rodenita, Muridae) in the ural region. Russian Journal of Genetics, 47, 332–339. doi: 10.1134/S1022795411030094

534. Molfetti, É., Torres Vilaça, S., Georges, J. Y., Plot, V., Delcroix, E., Le Scao, R., … de Thoisy, B. (2013). Recent Demographic History and Present Fine-Scale Structure in the Northwest Atlantic Leatherback (Dermochelys coriacea) Turtle Population. PLoS ONE, 8, e58061. doi: 10.1371/journal.pone.0058061

535. Monroe, E. M., & Britten, H. B. (2015). Single-sample estimation of effective population size in several populations of the endangered Hine’s emerald dragonfly. Freshwater Science, 34, 1058–1064. doi: 10.1086/682073

536. Monroe, E. M., Alexander, K. D., & Britten, H. B. (2016). Still here after all these years: the persistence of the Uncompahgre fritillary butterfly. Journal of Insect Conservation, 20, 305–313. doi: 10.1007/s10841-016-9867-9

537. Montes, I., Iriondo, M., Manzano, C., Santos, M., Conklin, D., Carvalho, G. R., … Estonba, A. (2016). No loss of genetic diversity in the exploited and recently collapsed population of Bay of Biscay anchovy (Engraulis encrasicolus, L.). Marine Biology, 163, 1–10. doi: 10.1007/s00227-016-2866-2

538. Moodley, Y., Russo, I.-R. M., Robovsky, J., Dalton, D. L., Kotzé, A., Smith, S., … Bruford, M. W. (2018). Contrasting evolutionary history, anthropogenic declines and genetic contact in the northern and southern white rhinoceros (Ceratotherium simum). Proceedings of the Royal Society B, 285, 20181567. doi: 10.1098/rspb.2018.1567

539. Moody, K. N., Hunter, S. N., Childress, M. J., Blob, R. W., Schoenfuss, H. L., Blum, M. J., & Ptacek, M. B. (2015). Local adaptation despite high gene flow in the waterfall-climbing Hawaiian goby, Sicyopterus stimpsoni. Molecular Ecology, 24, 545–563. doi: 10.1111/mec.13016

540. Moon, H. S., Nicholson, J. S., Heineman, A., Lion, K., Van Der Hoeven, R., Hayes, A. J., & Lewis, R. S. (2009). Changes in genetic diversity of U.S. flue-cured tobacco germplasm over seven decades of cultivar development. Crop Science, 49, 498–508. doi: 10.2135/cropsci2008.05.0253

541. Moraes, A. M., Ruiz-Miranda, C. R., Ribeiro, M. C., Grativol, A. D., da S. Carvalho, C., Dietz, J. M., … Galetti, P. M. (2017). Temporal genetic dynamics of reintroduced and translocated populations of the endangered golden lion tamarin (Leontopithecus rosalia). Conservation Genetics, 18, 995–1009. doi: 10.1007/s10592-017-0948-4

542. Morvezen, R., Boudry, P., Laroche, J., & Charrier, G. (2016). Stock enhancement or sea ranching? Insights from monitoring the genetic diversity, relatedness and effective population size in a seeded great scallop population (Pecten maximus). Heredity, 117, 142–148. doi: 10.1038/hdy.2016.42

543. Möst, M., Oexle, S., Marková, S., Aidukaite, D., Baumgartner, L., Stich, H. B., … Spaak, P. (2015). Population genetic dynamics of an invasion reconstructed from the sediment egg bank. Molecular Ecology, 24, 4074–4093. doi: 10.1111/mec.13298

544. Mourier, J., Buray, N., Schultz, J. K., Clua, E., & Planes, S. (2013). Genetic Network and Breeding Patterns of a Sicklefin Lemon Shark (Negaprion acutidens) Population in the Society Islands, French Polynesia. PLoS ONE, 8, e73899. doi: 10.1371/journal.pone.0073899

545. Mousset, M., David, P., Petit, C., Pouzadoux, J., Hatt, C., Flaven, É., … Mignot, A. (2016). Lower selfing rates in metallicolous populations than in non-metallicolous populations of the pseudometallophyte Noccaea caerulescens (Brassicaceae) in Southern France. Annals of Botany, 117, 507–519. doi: 10.1093/aob/mcv191

546. Moyer, G. R., Sweka, J. A., & Peterson, D. L. (2012). Past and present processes influencing genetic diversity and effective population size in a natural population of Atlantic sturgeon. Transactions of the American Fisheries Society, 141, 56–67. doi: 10.1080/00028487.2011.651073

547. Mudrik, E. A., Kashentseva, T. A., Postelnykh, K. A., Nosachenko, G. V, & Politov, D. V. (2014). Genetic diversity and relatedness in different generations of the Siberian crane (Grus leucogeranus Pallas) captive population. Russian Journal of Genetics, 50, 1192–1199. doi: 10.1134/S1022795414100093

548. Mueller, A. K., Chakarov, N., Krüger, O., & Hoffman, J. I. (2016). Long-term effective population size dynamics of an intensively monitored vertebrate population. Heredity, 117, 290–299. doi: 10.1038/hdy.2016.67

549. Müller, J., Wöll, S., Fuchs, U., & Seitz, A. (2001). Genetic interchange of Dreissena polymorpha populations across a canal. Heredity, 86, 103–109. doi: 10.1046/j.1365-2540.2001.00816.x

550. Mundy, N. I., Winchell, C. S., Burr, T., & Woodruff, D. S. (1997). Microsatellite variation and microevolution in the critically endangered San Clemente Island loggerhead shrike (Lanius ludovicianus mearnsi). Proceedings of the Royal Society B, 264, 869–875. doi: 10.1098/rspb.1997.0121

551. Muñoz-Colmenero, M., Jeunen, G. J., Borrell, Y. J., Martinez, J. L., Turrero, P., & Garcia-Vazquez, E. (2015). Response of top shell assemblages to cyclogenesis disturbances. A case study in the Bay of Biscay. Marine Environmental Research, 112, 2–10. doi: 10.1016/j.marenvres.2015.06.012

552. Muñoz-Fuentes, V., Green, A. J., Negro, J. J., & Sorenson, M. D. (2005). Population structure and loss of genetic diversity in the endangered white-headed duck, Oxyura leucocephala. Conservation Genetics, 6, 999–1015. doi: 10.1007/s10592-005-9093-6

553. Muñoz, I., Pinto, M. A., & De La Rúa, P. (2014). Effects of queen importation on the genetic diversity of Macaronesian island honey bee populations (Apis mellifera Linneaus 1758). Journal of Apicultural Research, 53, 296–302. doi: 10.3896/IBRA.1.53.2.11

554. Murphy, S. M., Augustine, B. C., Ulrey, W. A., Guthrie, J. M., Scheick, B. K., McCown, J. W., & Cox, J. J. (2017). Consequences of severe habitat fragmentation on density, genetics, and spatial capture-recapture analysis of a small bear population. PLoS ONE, 12, e0181849. doi: 10.1371/journal.pone.0181849

555. Murphy, S. M., Cox, J. J., Clark, J. D., Augustine, B. C., Hast, J. T., Gibbs, D., … Dobey, S. (2015). Rapid growth and genetic diversity retention in an isolated reintroduced black bear population in the central Appalachians. Journal of Wildlife Management, 79, 807–818. doi: 10.1002/jwmg.886

556. Mussmann, S. M., Douglas, M. R., Anthonysamy, W. J. B., Davis, M. A., Simpson, S. A., Louis, W., & Douglas, M. E. (2017). Genetic rescue, the greater prairie chicken and the problem of conservation reliance in the Anthropocene. Royal Society Open Science, 4, 1–14. doi: 10.1098/rsos.160736

557. Nabholz, B., Sarah, G., Sabot, F., Ruiz, M., Adam, H., Nidelet, S., … Glémin, S. (2014). Transcriptome population genomics reveals severe bottleneck and domestication cost in the African rice (Oryza glaberrima). Molecular Ecology, 23, 2210–2227. doi: 10.1111/mec.12738

558. Nair, A., Fountain, T., Ikonen, S., Ojanen, S. P., & Van Nouhuys, S. (2016). Spatial and temporal genetic structure at the fourth trophic level in a fragmented landscape. Proceedings of the Royal Society B, 283, 1–8. doi: 10.1098/rspb.2016.0668

559. Nakahama, N., & Isagi, Y. (2018). Recent transitions in genetic diversity and structure in the endangered semi-natural grassland butterfly, Melitaea protomedia, in Japan. Insect Conservation and Diversity, 11, 330–340. doi: 10.1111/icad.12280

560. Namroud, M. C., Tremblay, F., & Bergeron, Y. (2005). Temporal variation in quaking aspen (Populus tremuloides) genetic and clonal structures in the mixedwood boreal forest of eastern Canada. Ecoscience, 12, 82–91. doi: 10.2980/i1195-6860-12-1-82.1

561. Narum, S. R., Boe, S., Moran, P., & Powell, M. (2006). Small-Scale Genetic Structure and Variation in Steelhead of the Grande Ronde River, Oregon, USA. Transactions of the American Fisheries Society, 135, 979–986. doi: 10.1577/t05-070.1

562. Nathalie, T., & Bernatchez, L. (1999). Stability of population structure and genetic diversity across generations assessed by microsatellites among sympatric populations of landlocked Atlantic salmon (Salmo salar L .). *Molecular Ecology*, *8*, 169–179. doi: 10.1046/j.1365-294X.1999.00547.x

563. Navajas, M., Perrot-Minnot, M. J., Lagnel, J., Migeon, A., Bourse, T., & Cornuet, J. M. (2002). Genetic structure of a greenhouse population of the spider mite Tetranychus urticae: Spatio-temporal analysis with microsatellite markers. Insect Molecular Biology, 11, 157–165. doi: 10.1046/j.1365-2583.2002.00320.x

564. Negro, S., Solé, M., Pelayo, R., Gómez, M. D., Azor, P. J., & Valera, M. (2016). Molecular diversity between two cohorts of six Spanish riding-horse breeds: Impact of selection in Crossbred vs Purebred populations. Livestock Science, 193, 88–91. doi: 10.1016/j.livsci.2016.09.013

565. Neophytou, C., Gärtner, S. M., Vargas-Gaete, R., & Michiels, H. G. (2015). Genetic variation of Central European oaks: shaped by evolutionary factors and human intervention? Tree Genetics and Genomes, 11, 1–15. doi: 10.1007/s11295-015-0905-7

566. Neuwald, J. L., & Templeton, A. R. (2013). Genetic restoration in the eastern collared lizard under prescribed woodland burning. Molecular Ecology, 22, 3666–3679. doi: 10.1111/mec.12306

567. Ng, W. C., Leung, F. C. C., Chak, S. T. C., Slingsby, G., & Williams, G. A. (2010). Temporal genetic variation in populations of the limpet Cellana grata from Hong Kong shores. Marine Biology, 157, 325–337. doi: 10.1007/s00227-009-1320-0

568. Nielsen, E. E., Hansen, M. M., & Loeschcke, V. (1999). Genetic variation in time and space: microsatellite analysis of extinct and extant populations of Atlantic salmon. Evolution, 53, 261–268. doi: 10.1111/j.1558-5646.1999.tb05351.x

569. Nielsen, E. E., Hemmer-Hansen, J., Poulsen, N. A., Loeschcke, V., Moen, T., Johansen, T., … Carvalho, G. R. (2009). Genomic signatures of local directional selection in a high gene flow marine organism; The Atlantic cod (Gadus morhua). BMC Evolutionary Biology, 9, 1–11. doi: 10.1186/1471-2148-9-276

570. Nielsen, R. K., Pertoldi, C., & Loeschcke, V. (2007). Genetic evaluation of the captive breeding program of the Persian wild ass. Journal of Zoology, 272, 349–357. doi: 10.1111/j.1469-7998.2007.00294.x

571. Nikolic, N., Butler, J. R. A., Baglènire, J. L., Laughton, R., McMyn, I. A. G., & Chevalet, C. (2009). An examination of genetic diversity and effective population size in Atlantic salmon populations. Genetics Research, 91, 395–412. doi: 10.1017/S0016672309990346

572. Nishimura, S., Hinomoto, N., & Takafuji, A. (2005). Gene flow and spatio-temporal genetic variation among sympatric populations of Tetranychus kanzawai (Acari: Tetranychidae) occurring on different host plants, as estimated by microsatellite gene diversity. Experimental and Applied Acarology, 35, 59–71. doi: 10.1007/s10493-004-2005-5

573. Nittinger, F., Gamauf, A., Pinsker, W., Wink, M., & Haring, E. (2007). Phylogeography and population structure of the saker falcon (Falco cherrug) and the influence of hybridization: mitochondrial and microsatellite data. *Molecular Ecology*, *16*, 1497–1517. doi: 10.1111/j.1365-294X.2007.03245.x

574. Norton, A. J., Gower, C. M., Lamberton, P. H. L., Webster, B. L., Lwambo, N. J. S., Blair, L., … Webster, J. P. (2010). Genetic consequences of mass human chemotherapy for Schistosoma mansoni: Population structure pre- and post-praziquantel treatment in Tanzania. American Journal of Tropical Medicine and Hygiene, 83, 951–957. doi: 10.4269/ajtmh.2010.10-0283

575. Nunziata, S. O., Lance, S. L., Scott, D. E., Lemmon, E. M., & Weisrock, D. W. (2017). Genomic data detect corresponding signatures of population size change on an ecological time scale in two salamander species. Molecular Ecology, 26, 1060–1074. doi: 10.1111/mec.13988

576. Nunziata, S. O., Scott, D. E., & Lance, S. L. (2015). Temporal genetic and demographic monitoring of pond-breeding amphibians in three contrasting population systems. Conservation Genetics, 16, 1335–1344. doi: 10.1007/s10592-015-0743-z

577. O'Donnell, T. P., Arnott, S. A., Denson, M. R., & Darden, T. L. (2016). Effects of Cold Winters on the Genetic Diversity of an Estuarine Fish, the Spotted Seatrout. *Marine and Coastal Fisheries*, *8*, 263–276. doi: 10.1080/19425120.2016.1152333

578. O'Leary, S. J., Martinez, C., Baumann, H., Abercrombie, D. L., Conover, D. O., Poulakis, G. R., … Chapman, D. D. (2016). Population genetics and geometric morphometrics of the key silverside, Menidia conchorum, a marine fish in a highly-fragmented, inland habitat. Bulletin of Marine Science, 92, 33–50. doi: 10.5343/bms.2015.1035

579. O'Reilly, P. T., Canino, M. F., Bailey, K. M., & Bentzen, P. (2004). Inverse relationship between FST and microsatellite polymorphism in the marine fish, walleye pollock (Theragra chalcogramma): Implications for resolving weak population structure. Molecular Ecology, 13, 1799–1814. doi: 10.1111/j.1365-294X.2004.02214.x

580. Ochoa, A., Gasca, J., Ceballos, G. J., & Eguiarte, L. E. (2012). Spatiotemporal population genetics of the endangered Perote ground squirrel (Xerospermophilus perotensis) in a fragmented landscape. Journal of Mammalogy, 93, 1061–1074. doi: 10.1644/11-MAMM-A-371.1

581. Oddou-Muratorio, S., Klein, E. K., Vendramin, G. G., & Fady, B. (2011). Spatial vs. temporal effects on demographic and genetic structures: The roles of dispersal, masting and differential mortality on patterns of recruitment in Fagus sylvatica. Molecular Ecology, 20, 1997–2010. doi: 10.1111/j.1365-294X.2011.05039.x

582. Oh, G. S., Kang, S. S., & Chung, M. G. (1996). Temporal genetic structure in Camellia japonica (Theaceae). *Genes & Genetic Systems*, *71*, 9–13. doi: 10.1266/GGS.71.9

583. Ohbayashi, K., Hodoki, Y., Kondo, N. I., Kunii, H., & Shimada, M. (2017). A massive tsunami promoted gene flow and increased genetic diversity in a near threatened plant species. Scientific Reports, 7, 1–9. doi: 10.1038/s41598-017-11270-5

584. Oka, T., Tadano, R., Goto, T., Shiraishi, J. I., Osman, S. A. M., Nagasaka, N., … Tsudzuki, M. (2014). Time-dependent changes in the genetic diversity and structure of the onagadori breed of chickens based on microsatellite DNA polymorphisms. Journal of Poultry Science, 51, 262–269. doi: 10.2141/jpsa.0130199

585. Okello, J. B. A., Wittemyer, G., Rasmussen, H. B., Arctander, P., Nyakaana, S., Douglas-Hamilton, I., & Siegismund, H. R. (2008). Effective population size dynamics reveal impacts of historic climatic events and recent anthropogenic pressure in African elephants. Molecular Ecology, 17, 3788–3799. doi: 10.1111/j.1365-294X.2008.03871.x

586. Olafsdottir, G. A., Westfall, K. M., Edvardsson, R., & Palsson, S. (2014). Historical DNA reveals the demographic history of Atlantic cod (Gadus morhua) in medieval and early modern Iceland. Proceedings of the Royal Society B, 281, 1–8. doi: 10.1098/rspb.2013.2976

587. Oliver, M. K., & Piertney, S. B. (2012). Selection maintains MHC diversity through a natural population bottleneck. Molecular Biology and Evolution, 29, 1713–1720. doi: 10.1093/molbev/mss063

588. Oliver, M. K., Lambin, X., Cornulier, T., & Piertney, S. B. (2009). Spatio-temporal variation in the strength and mode of selection acting on major histocompatibility complex diversity in water vole (Arvicola terrestris) metapopulations. Molecular Ecology, 18, 80–92. doi: 10.1111/j.1365-294X.2008.04015.x

589. Olmos-Pérez, L., Roura, Á., Pierce, G. J., & González, Á. F. (2018). Sepiolid paralarval diversity in a regional upwelling area of the NE Atlantic. Hydrobiologia, 808, 57–70. doi: 10.1007/s10750-017-3186-3

590. Onyabe, D. Y., & Conn, J. E. (2001). Population genetic structure of the malaria mosquito Anopheles arabiensis across Nigeria suggests range expansion. Molecular Ecology, 10, 2577–2591. doi: 10.1046/j.0962-1083.2001.01387.x

591. Orsini, L., Marshall, H., Cuenca Cambronero, M., Chaturvedi, A., Thomas, K. W., Pfrender, M. E., … De Meester, L. (2016). Temporal genetic stability in natural populations of the waterflea Daphnia magna in response to strong selection pressure. Molecular Ecology, 25, 6024–6038. doi: 10.1111/mec.13907

592. Ort, B. S., & Thornton, W. J. (2016). Changes in the population genetics of an invasive Spartina after 10 years of management. Biological Invasions, 18, 2267–2281. doi: 10.1007/s10530-016-1177-3

593. Ortego, J., Aparicio, J. M., Calabuig, G., & Cordero, P. J. (2007). Increase of heterozygosity in a growing population of lesser kestrels. Biology Letters, 3, 585–588. doi: 10.1098/rsbl.2007.0268

594. Ortego, J., Yannic, G., Shafer, A. B. A., Mainguy, J., Festa-Bianchet, M., Coltman, D. W., & Côté, S. D. (2011). Temporal dynamics of genetic variability in a mountain goat (Oreamnos americanus) population. Molecular Ecology, 20, 1601–1611. doi: 10.1111/j.1365-294X.2011.05022.x

595. Ortells, R., Vanoverbeke, J., Louette, G., & De Meester, L. (2014). Colonization of Daphnia magna in a newly created pond: founder effects and secondary immigrants. Hydrobiologia, 723, 167–179. doi: 10.1007/s10750-013-1593-7

596. Ottewell, K. M., Bickerton, D., & Lowe, A. J. (2011). Can a seed bank provide demographic and genetic rescue in a declining population of the endangered shrub Acacia pinguifolia? Conservation Genetics, 12, 669–678. doi: 10.1007/s10592-010-0173-x

597. Ottewell, K., Dunlop, J., Thomas, N., Morris, K., Coates, D., & Byrne, M. (2014). Evaluating success of translocations in maintaining genetic diversity in a threatened mammal. *Biological Conservation*, *171*, 209–219. doi: 10.1016/j.biocon.2014.01.012

598. Ou, W., Takekawa, S., Yamada, T., Terada, C., Uno, H., Nagata, J., … Saitoh, T. (2014). Temporal change in the spatial genetic structure of a sika deer population with an expanding distribution range over a 15-year period. *Population Ecology*, *56*, 311–325. doi: 10.1007/s10144-013-0425-y

599. Ovčarenko, I., Kapantaidaki, D. E., Lindström, L., Gauthier, N., Tsagkarakou, A., Knott, K. E., & Vänninen, I. (2014). Agroecosystems shape population genetic structure of the greenhouse whitefly in Northern and Southern Europe. BMC Evolutionary Biology, 14, 1–17. doi: 10.1186/s12862-014-0165-4

600. Ovenden, J. R., Peel, D., Street, R., Courtney, A. J., Hoyle, S. D., Peel, S. L., & Podlich, H. (2007). The genetic effective and adult census size of an Australian population of tiger prawns (Penaeus esculentus). Molecular Ecology, 16, 127–138. doi: 10.1111/j.1365-294X.2006.03132.x

601. Ozer, F., Gellerman, H., & Ashley, M. V. (2011). Genetic impacts of Anacapa deer mice reintroductions following rat eradication. Molecular Ecology, 20, 3525–3539. doi: 10.1111/j.1365-294X.2011.05165.x

602. Ozerov, M. Y., Gross, R., Bruneaux, M., Vähä, J. P., Burimski, O., Pukk, L., & Vasemägi, A. (2016). Genomewide introgressive hybridization patterns in wild Atlantic salmon influenced by inadvertent gene flow from hatchery releases. Molecular Ecology, 25, 1275–1293. doi: 10.1111/mec.13570

603. Pacioni, C., Hunt, H., Allentoft, M. E., Vaughan, T. G., Wayne, A. F., Baynes, A., … Bunce, M. (2015). Genetic diversity loss in a biodiversity hotspot: Ancient DNA quantifies genetic decline and former connectivity in a critically endangered marsupial. Molecular Ecology, 24, 5813–5828. doi: 10.1111/mec.13430

604. Palmer, G., Hogan, J. D., Sterba-Boatwright, B. D., & Overath, R. D. (2016). Invasive lionfish Pterois volitans reduce the density but not the genetic diversity of a native reef fish. Marine Ecology Progress Series, 558, 223–234. doi: 10.3354/meps11924

605. Palomares, F., Godoy, J. A., López-Bao, J. V, Rodríguez, A., Roques, S., Casas-Marce, M., … Delibes, M. (2012). Possible Extinction Vortex for a Population of Iberian Lynx on the Verge of Extirpation. Conservation Biology, 26, 689–697. doi: 10.1111/j.1523-1739.2012.01870.x

606. Pante, M. J. R., Gjerde, B., & McMillan, I. (2001). Inbreeding levels in selected populations of rainbow trout, Oncorhynchus mykiss. Aquaculture, 192, 213–224. doi: 10.1016/S0044-8486(00)00466-X

607. Papa, R., Israel, J. A., Marzano, F. N., & May, B. (2007). Assessment of genetic variation between reproductive ecotypes of Klamath River steelhead reveals differentiation associated with different run-timings. Journal of Applied Ichthyology, 23, 142–146. doi: 10.1111/j.1439-0426.2006.00825.x

608. Papetti, C., Pujolar, J. M., Mezzavilla, M., La Mesa, M., Rock, J., Zane, L., & Patarnello, T. (2012). Population genetic structure and gene flow patterns between populations of the Antarctic icefish Chionodraco rastrospinosus. Journal of Biogeography, 39, 1361–1372. doi: 10.1111/j.1365-2699.2011.02682.x

609. Papetti, C., Susana, E., Patarnello, T., & Zane, L. (2009). Spatial and temporal boundaries to gene flow between Chaenocephalus aceratus populations at South Orkney and South Shetlands. *Marine Ecology Progress Series, 376*, 269–281. doi: 10.3354/meps07831

610. Papetti, C., Zane, L., Bortolotto, E., Bucklin, A., & Patarnello, T. (2005). Genetic differentiation and local temporal stability of population structure in the euphausiid Meganyctiphanes norvegica. *Marine Ecology Progress Series*, *289*, 225–235. doi: 10.3354/meps289225

611. Paris, J. R., King, R. A., & Stevens, J. R. (2015). Human mining activity across the ages determines the genetic structure of modern brown trout (Salmo trutta L.) populations. Evolutionary Applications, 8, 573–585. doi: 10.1111/eva.12266

612. Patrício, A. R., Vélez-Zuazo, X., Van Dam, R. P., & Diez, C. E. (2017). Genetic composition and origin of juvenile green turtles foraging at Culebra, Puerto Rico, as revealed by mtDNA. Latin American Journal of Aquatic Research, 45, 506–520. doi: 10.3856/vol45-issue3-fulltext-2

613. Pavey, S. A., Hamon, T. R., & Nielsen, J. L. (2007). Revisiting evolutionary dead ends in sockeye salmon (Oncorhynchus nerka) life history. Canadian Journal of Fisheries and Aquatic Sciences, 64, 1199–1208. doi: 10.1139/F07-091

614. Paz-Vinas, I., Comte, L., Chevalier, M., Dubut, V., Veyssiere, C., Grenouillet, G., … Blanchet, S. (2013). Combining genetic and demographic data for prioritizing conservation actions: Insights from a threatened fish species. Ecology and Evolution, 3, 2696–2710. doi: 10.1002/ece3.645

615. Paz, G., Douek, J., Mo, C., Goren, M., & Rinkevich, B. (2003). Genetic structure of Botryllus schlosseri (Tunicata) populations from the Mediterranean coast of Israel. *Marine Ecology Progress Series, 250*, 153–162. doi: 10.3354/meps250153

616. Pecsenye, K., Rácz, R., Bereczki, J., Bátori, E., & Varga, Z. (2014). Loss of genetic variation in declining populations of Aricia artaxerxes in Northern Hungary. Journal of Insect Conservation, 18, 233–243. doi: 10.1007/s10841-014-9634-8

617. Pecsenye, K., Tóth, A., Bereczki, J., & Varga, Z. (2017). A possible genetic basis for vulnerability in Euphydryas maturna (Lepidoptera: Nymphalidae). Genetica, 145, 151–161. doi: 10.1007/s10709-017-9953-2

618. Peng, Y., Lachmuth, S., Gallegos, S. C., Kessler, M., Ramsay, P. M., Renison, D., … Hensen, I. (2015). Pleistocene climatic oscillations rather than recent human disturbance influence genetic diversity in one of the world's highest treeline species. American Journal of Botany, 102, 1676–1684. doi: 10.3732/ajb.1500131

619. Pereoglou, F., Lindenmayer, D. B., MacGregor, C., Ford, F., Wood, J., & Banks, S. C. (2013). Landscape genetics of an early successional specialist in a disturbance-prone environment. Molecular Ecology, 22, 1267–1281. doi: 10.1111/mec.12172

620. Pérez De Rosas, A. R., Segura, E. L., Fichera, L., & García, B. A. (2008). Macrogeographic and microgeographic genetic structure of the Chagas' disease vector Triatoma infestans (Hemiptera: Reduviidae) from Catamarca, Argentina. Genetica, 133, 247–260. doi: 10.1007/s10709-007-9208-8

621. Pérez-Figueroa, A., Fernández, C., Amaro, R., Hermida, M., & San Miguel, E. (2015). Population structure and effective/census population size ratio in threatened three-spined stickleback populations from an isolated river basin in northwest Spain. Genetica, 143, 403–411. doi: 10.1007/s10709-015-9839-0

622. Pérez-Portela, R., Turon, X., & Bishop, J. D. D. (2012). Bottlenecks and loss of genetic diversity: patio-temporal patterns of genetic structure in an ascidian recently introduced in Europe. *Marine Ecology Progress Series*, *451*, 93–105. doi: 10.3354/meps09560

623. Persoons, A., Hayden, K. J., Fabre, B., Frey, P., De Mita, S., Tellier, A., & Halkett, F. (2017). The escalatory Red Queen: Population extinction and replacement following arms race dynamics in poplar rust. Molecular Ecology, 26, 1902–1918. doi: 10.1111/mec.13980

624. Pertoldi, C., Barker, S. F., Madsen, A. B., Jørgensen, H., Randi, E., Muñoz, J., … Loeschcke, V. (2008). Spatio-temporal population genetics of the Danish pine marten (Martes martes). Biological Journal of the Linnean Society, 93, 457–464. doi: 10.1111/j.1095-8312.2007.00892.x

625. Pertoldi, C., Loeschcke, V., Randi, E., Madsen, A. B., Hansen, M. M., Bijlsma, R., & Van De Zande, L. (2005). Present and past microsatellite variation and assessment of genetic structure in Eurasian badger (Meles meles) in Denmark. Journal of Zoology, 265, 387–394. doi: 10.1017/S0952836905006485

626. Pertoldi, C., Pellegrino, I., Cucco, M., Mucci, N., Randi, E., Laursen, J. T., … Kristensen, T. N. (2012). Genetic consequences of population decline in the Danish population of the little owl (Athene noctua). Evolutionary Ecology Research, 14, 921–932.

627. Petersen, C., Saebelfeld, M., Barbosa, C., Pees, B., Hermann, R. J., Schalkowski, R., … Schulenburg, H. (2015). Ten years of life in compost: Temporal and spatial variation of North German Caenorhabditis elegans populations. Ecology and Evolution, 5, 3250–3263. doi: 10.1002/ece3.1605

628. Pichler, F. B., & Baker, C. S. (2000). Loss of genetic diversity in the endemic Hector's dolphin due to fisheries-related mortality. Proceedings of the Royal Society B, 267, 97–102. doi: 10.1098/rspb.2000.0972

629. Piggott, M. P., Banks, S. C., MacGregor, C., & Lindenmayer, D. B. (2018). Population genetic patterns in an irruptive species, the long-nosed bandicoot (Perameles nasuta). Conservation Genetics, 19, 655–663. doi: 10.1007/s10592-017-1044-5

630. Pilger, T. J., Gido, K. B., Propst, D. L., Whitney, J. E., & Turner, T. F. (2015). Comparative conservation genetics of protected endemic fishes in an arid-land riverscape. Conservation Genetics, 16, 875–888. doi: 10.1007/s10592-015-0707-3

631. Pineda, M. C., Lorente, B., López-Legentil, S., Palacín, C., & Turon, X. (2016). Stochasticity in space, persistence in time: Genetic heterogeneity in harbour populations of the introduced ascidian Styela plicata. PeerJ, 4, 1–21. doi: 10.7717/peerj.2158

632. Pineda, M. C., Turon, X., Pérez-Portela, R., & López-Legentil, S. (2016). Stable populations in unstable habitats: temporal genetic structure of the introduced ascidian Styela plicata in North Carolina. Marine Biology, 163, 1–14. doi: 10.1007/s00227-016-2829-7

633. Pinsky, M. L., Newsome, S. D., Dickerson, B. R., Fang, Y., Van Tuinen, M., Kennett, D. J., … Hadly, E. A. (2010). Dispersal provided resilience to range collapse in a marine mammal: Insights from the past to inform conservation biology. Molecular Ecology, 19, 2418–2429. doi: 10.1111/j.1365-294X.2010.04671.x

634. Pinto, M. A., Rubink, W. L., Patton, J. C., Coulson, R. N., & Johnston, J. S. (2005). Africanization in the United States: Replacement of feral European honeybees (Apis mellifera L.) by an African hybrid swarm. Genetics, 170, 1653–1665. doi: 10.1534/genetics.104.035030

635. Pita, A., Leal, A., Santafé-Muñoz, A., Piñeiro, C., & Presa, P. (2016). Genetic inference of demographic connectivity in the Atlantic European hake metapopulation (Merluccius merluccius) over a spatio-temporal framework. *Fisheries Research*, *179*, 291–301. doi: 10.1016/j.fishres.2016.03.017

636. Pita, A., Pérez, M., Cerviño, S., & Presa, P. (2011). What can gene flow and recruitment dynamics tell us about connectivity between European hake stocks in the Eastern North Atlantic? Continental Shelf Research, 31, 376–387. doi: 10.1016/j.csr.2010.09.010

637. Pitt, D., Bruford, M. W., Barbato, M., Orozco-terWengel, P., Martínez, R., & Sevane, N. (2019). Demography and rapid local adaptation shape Creole cattle genome diversity in the tropics. Evolutionary Applications, 12, 105–122. doi: 10.1111/eva.12641

638. Planes, S., & Lenfant, P. (2002). Temporal change in the genetic structure between and within cohorts of a marine fish, Diplodus sargus, induced by a large variance in individual reproductive success. Molecular Ecology, 11, 1515–1524. doi: 10.1046/j.1365-294X.2002.01521.x

639. Ploshnitsa, A. I., Goltsman, M. E., Happ, G. M., Macdonald, D. W., & Kennedy, L. J. (2013). Historical and modern neutral genetic variability in Mednyi Arctic foxes passed through a severe bottleneck. Journal of Zoology, 289, 68–76. doi: 10.1111/j.1469-7998.2012.00964.x

640. Ploshnitsa, A. I., Goltsman, M. E., Macdonald, D. W., Kennedy, L. J., & Sommer, S. (2011). Impact of historical founder effects and a recent bottleneck on MHC variability in Commander Arctic foxes (Vulpes lagopus). Ecology and Evolution, 2, 165–180. doi: 10.1002/ece3.42

641. Plot, V., de Thoisy, B., Blanc, S., Kelle, L., Lavergne, A., Roger-Bérubet, H., … Georges, J. Y. (2012). Reproductive synchrony in a recovering bottlenecked sea turtle population. Journal of Animal Ecology, 81, 341–351. doi: 10.1111/j.1365-2656.2011.01915.x

642. Pollegioni, P., Woeste, K., Chiocchini, F., Del Lungo, S., Ciolfi, M., Olimpieri, I., … Malvolti, M. E. (2017). Rethinking the history of common walnut (Juglans regia L.) in Europe: Its origins and human interactions. PLoS ONE, 12, e0172541. doi: 10.1371/journal.pone.0172541

643. Ponnikas, S., Ollila, T., & Kvist, L. (2017). Turnover and post-bottleneck genetic structure in a recovering population of Peregrine Falcons Falco peregrinus. Ibis, 159, 311–323. doi: 10.1111/ibi.12460

644. Popovic, D., Panagiotopoulou, H., Kleszcz, M., Baca, M., Rutkowski, R., Heese, T., … Stankovic, A. (2013). Restitution of vimba (Vimba vimba, Cyprinidae) in Poland: Genetic variability of existing and restored populations. Ichthyological Research, 60, 149–158. doi: 10.1007/s10228-012-0326-7

645. Portis, E., Acquadro, A., Comino, C., & Lanteri, S. (2004). Effect of farmers' seed selection on genetic variation of a landrace population of pepper (Capsicum annuum L.), grown in North-West Italy. Genetic Resources and Crop Evolution, 51, 581–590. doi: 10.1023/B:GRES.0000024648.48164.c3

646. Poteaux, C., Baubet, E., Kaminski, G., Brandt, S., Dobson, F. S., & Baudoin, C. (2009). Socio-genetic structure and mating system of a wild boar population. Journal of Zoology, 278, 116–125. doi: 10.1111/j.1469-7998.2009.00553.x

647. Poteaux, C., Beaudou, D., & Berrebi, P. (1998). Temporal variations of genetic introgression in stocked brown trout populations. Journal of Fish Biology, 53, 701–713. doi: 10.1006/jfbi.1998.0736

648. Poulsen, N. A., Nielsen, E. E., Schierup, M. H., Loeschcke, V., & Grønkjær, P. (2006). Long-term stability and effective population size in North Sea and Baltic Sea cod (Gadus morhua). Molecular Ecology, 15, 321–331. doi: 10.1111/j.1365-294X.2005.02777.x

649. Price, M. R., & Hadfield, M. G. (2014). Population genetics and the effects of a severe bottleneck in an ex situ population of critically endangered hawaiian tree snails. PLoS ONE, 9, e114377. doi: 10.1371/journal.pone.0114377

650. Pruett, C. L., Li, T., & Winker, K. (2018). Population genetics of Alaska Common Raven show dispersal and isolation in the world's largest songbird. Auk, 135, 868–880. doi: 10.1642/AUK-17-144.1

651. Pruett, C. L., Tanksley, S. M., Small, M. F., Taylor, J. F., & Forstner, M. R. J. (2011). The effects of range expansion on the population genetics of white-winged doves in texas. The American Midland Naturalist, 166, 415–425. doi: 10.1674/0003-0031-166.2.415

652. Pujolar, J. M., Bevacqua, D., Capoccioni, F., Ciccotti, E., de Leo, G. A., & Zane, L. (2011). No apparent genetic bottleneck in the demographically declining European eel using molecular genetics and forward-time simulations. Conservation Genetics, 12, 813–825. doi: 10.1007/s10592-011-0188-y

653. Pujolar, J. M., Roldán, M. I., & Pla, C. (2002). A genetic assessment of the population structure of swordfish (Xiphias gladius) in the Mediterranean Sea. Journal of Experimental Marine Biology and Ecology, 276, 19–29. doi: 10.1016/S0022-0981(02)00245-9

654. Pujolar, J. M., Roldán, M. I., & Pla, C. (2003). Genetic analysis of tuna populations, Thunnus thynnus thynnus and T. alalunga. Marine Biology, 143, 613–621. doi: 10.1007/s00227-003-1080-1

655. Qi, J., Liu, X., Shen, D., Miao, H., Xie, B., Li, X., … Huang, S. (2013). A genomic variation map provides insights into the genetic basis of cucumber domestication and diversity. Nature Genetics, 45, 1510–1515. doi: 10.1038/ng.2801

656. Qiu, J., Xu, J., & Shi, J. (2014). Molecular characterization of the Fusarium graminearum species complex in Eastern China. European Journal of Plant Pathology, 139, 811–823. doi: 10.1007/s10658-014-0435-4

657. Queney, G., Ferrand, N., Marchandeau, S., Azevedo, M., Mougel, F., Branco, M., & Monnerot, M. (2000). Absence of a genetic bottleneck in a wild rabbit (Oryctolagus cuniculus) population exposed to a severe viral epizootic. Molecular Ecology, 9, 1253–1264. doi: 10.1046/j.1365-294X.2000.01003.x

658. Quintela, M., Skaug, H. J., Øien, N., Haug, T., Seliussen, B. B., Solvang, H. K., … Glover, K. A. (2014). Investigating population genetic structure in a highly mobile marine organism: The minke whale balaenoptera acutorostrata acutorostrata in the north east atlantic. PLoS ONE, 9, e108640. doi: 10.1371/journal.pone.0108640

659. Rajora, O. P., Rahman, M. H., Buchert, G. P., & Dancik, B. P. (2000). Microsatellite DNA analysis of genetic effects of harvesting in old-growth eastern white pine (Pinus strobus) in Ontario, Canada. Molecular Ecology, 9, 339–348. doi: 10.1046/j.1365-294X.2000.00886.x

660. Ramp, J. M., Collinge, S. K., & Ranker, T. A. (2006). Restoration genetics of the vernal pool endemic Lasthenia conjugens (Asteraceae). Conservation Genetics, 7, 631–649. doi: 10.1007/s10592-005-9052-2

661. Rannamäe, E., Lõugas, L., Speller, C. F., Valk, H., Maldre, L., Wilczyński, J., … Saarma, U. (2016). Three thousand years of continuity in the maternal lineages of ancient sheep (Ovis aries) in Estonia. PLoS ONE, 11, e0163676. doi: 10.1371/journal.pone.0163676

662. Raquin, A. L., Depaulis, F., Lambert, A., Galic, N., Brabant, P., & Goldringer, I. (2008). Experimental estimation of mutation rates in a wheat population with a gene genealogy approach. Genetics, 179, 2195–2211. doi: 10.1534/genetics.107.071332

663. Ravago-Gotanco, R. G., & Juinio-Meñez, M. A. (2004). Population genetic structure of the milkfish, Chanos chanos, based on PCR-RFLP analysis of the mitochondrial control region. Marine Biology, 145, 789–801. doi: 10.1007/s00227-004-1372-0

664. Reding, D. M., Freed, L. A., Cann, R. L., & Fleischer, R. C. (2010). Spatial and temporal patterns of genetic diversity in an endangered Hawaiian honeycreeper, the Hawaii Akepa (Loxops coccineus coccineus). Conservation Genetics, 11, 225–240. doi: 10.1007/s10592-009-0025-8

665. Rekdal, S. L., Hansen, R. G., Borchers, D., Bachmann, L., Laidre, K. L., Wiig, Ø., … Heide-Jørgensen, M. P. (2015). Trends in bowhead whales in West Greenland: Aerial surveys vs. genetic capture-recapture analyses. Marine Mammal Science, 31, 133–154. doi: 10.1111/mms.12150

666. Rey, O., Fourtune, L., Paz-Vinas, I., Loot, G., Veyssière, C., Roche, B., & Blanchet, S. (2015). Elucidating the spatio-temporal dynamics of an emerging wildlife pathogen using approximate Bayesian computation. Molecular Ecology, 24, 5348–5363. doi: 10.1111/mec.13401

667. Reynolds, L. K., Stachowicz, J. J., Hughes, A. R., Kamel, S. J., Ort, B. S., & Grosberg, R. K. (2017). Temporal stability in patterns of genetic diversity and structure of a marine foundation species (Zostera marina). Heredity, 118, 404–412. doi: 10.1038/hdy.2016.114

668. Rhode, C., Bester-Van Der Merwe, A. E., & Roodt-Wilding, R. (2017). An assessment of spatio-temporal genetic variation in the South African abalone (Haliotis midae), using SNPs: implications for conservation management. Conservation Genetics, 18, 17–31. doi: 10.1007/s10592-016-0879-5

669. Rhodes, K. L., Lewis, R. I., Chapman, R. W., & Sadovy, Y. (2003). Genetic structure of camouflage grouper, Epinephelus polyphekadion (Pisces: Serranidae), in the western central Pacific. Marine Biology, 142, 771–776. doi: 10.1007/s00227-002-1002-7

670. Ribeiro, Â., Morán, P., & Caballero, A. (2008). Genetic diversity and effective size of the Atlantic salmon Salmo salar L. inhabiting the River Eo (Spain) following a stock collapse. Journal of Fish Biology, 72, 1933–1944. doi: 10.1111/j.1095-8649.2007.01769.x

671. Ribolli, J., Hoeinghaus, D. J., Johnson, J. A., Zaniboni-Filho, E., de Freitas, P. D., & Galetti, P. M. (2017). Isolation-by-time population structure in potamodromous Dourado Salminus brasiliensis in southern Brazil. Conservation Genetics, 18, 67–76. doi: 10.1007/s10592-016-0882-x

672. Rick, J. A., Moen, R. A., Erb, J. D., & Strasburg, J. L. (2017). Population structure and gene flow in a newly harvested gray wolf (Canis lupus) population. Conservation Genetics, 18, 1091–1104. doi: 10.1007/s10592-017-0961-7

673. Rikalainen, K., Aspi, J., Galarza, J. A., Koskela, E., & Mappes, T. (2012). Maintenance of genetic diversity in cyclic populations-a longitudinal analysis in Myodes glareolus. Ecology and Evolution, 2, 1491–1502. doi: 10.1002/ece3.277

674. Rivadeneira, C., Allen, J. M., & Reed, D. L. (2011). Microsatellite loci for testing temporal changes in the population genetics of the Florida mouse (Podomys floridanus). Conservation Genetics Resources, 3, 135–139. doi: 10.1007/s12686-010-9308-0

675. Rizzi, R., Tullo, E., Cito, A. M., Caroli, A., & Pieragostini, E. (2011). Monitoring of genetic diversity in the endangered Martina Franca donkey population. Journal of Animal Science, 89, 1304–1311. doi: 10.2527/jas.2010-3379

676. Robainas-Barcia, A., Blanco, G., Sánchez, J. A., Monnerot, M., Solignac, M., & García-Machado, E. (2008). Spatiotemporal genetic differentiation of Cuban natural populations of the pink shrimp Farfantepenaeus notialis. Genetica, 133, 283–294. doi: 10.1007/s10709-007-9212-z

677. Roberts, D. G., Gray, C. A., West, R. J., & Ayre, D. J. (2011). Temporal stability of a hybrid swarm between the migratory marine and estuarine fishes Acanthopagrus australis and A. butcheri. *Marine Ecology Progress Series, 421*, 199–204. doi: 10.3354/meps08901

678. Roberts, D. G., Ottewell, K. M., Whelan, R. J., & Ayre, D. J. (2014). Is the post-disturbance composition of a plant population determined by selection for outcrossed seedlings or by the composition of the seedbank? Heredity, 112, 409–414. doi: 10.1038/hdy.2013.119

679. Rodríguez-Muñoz, R., Rodríguez del Valle, C., Bañuelos, M. J., & Mirol, P. (2015). Revealing the consequences of male-biased trophy hunting on the maintenance of genetic variation. Conservation Genetics, 16, 1375–1394. doi: 10.1007/s10592-015-0747-8

680. Røed, K. H., Flagstad, Ø., Bjørnstad, G., & Hufthammer, A. K. (2011). Elucidating the ancestry of domestic reindeer from ancient DNA approaches. Quaternary International, 238, 83–88. doi: 10.1016/j.quaint.2010.07.031

681. Roos, L., Hinrichs, D., Nissen, T., & Krieter, J. (2015). Investigations into genetic variability in Holstein horse breed using pedigree data. *Livestock Science*, *177*, 25–32. doi: 10.1016/j.livsci.2015.04.013

682. Rosewich, U. L., Pettway, R. E., McDonald, B. A., Duncan, R. R., & Frederiksen, R. A. (1998). Genetic structure and temporal dynamics of a Colletotrichum graminicola population in a sorghum disease nursery. Phytopathology, 88, 1087–1093. doi: 10.1094/PHYTO.1998.88.10.1087

683. Rosvold, J., Røed, K. H., Hufthammer, A. K., Andersen, R., & Stenøien, H. K. (2012). Reconstructing the history of a fragmented and heavily exploited red deer population using ancient and contemporary DNA. BMC Evolutionary Biology, 12, 1471–2148. doi: 10.1186/1471-2148-12-191

684. Rotheray, E. L., Lepais, O., Nater, A., Krützen, M., Greminger, M., Goulson, D., & Bussière, L. F. (2012). Genetic variation and population decline of an endangered hoverfly Blera fallax (Diptera: Syrphidae). Conservation Genetics, 13, 1283–1291. doi: 10.1007/s10592-012-0371-9

685. Roy, M. S., Geffen, E., Smith, D., & Wayne, R. K. (1996). Molecular genetics of pre-1940 red wolves. Conservation Biology, 10, 1413–1424. doi: 10.1046/j.1523-1739.1996.10051413.x

686. Rubidge, E. M., Patton, J. L., Lim, M., Burton, A. C., Brashares, J. S., & Moritz, C. (2012). Climate-induced range contraction drives genetic erosion in an alpine mammal. Nature Climate Change, 2, 285–288. doi: 10.1038/nclimate1415

687. Ruggeri, P., Splendiani, A., Bonanomi, S., Arneri, E., Cingolani, N., Santojanni, A., … Caputo, V. (2012). Temporal genetic variation as revealed by a microsatellite analysis of European sardine (Sardina pilchardus) archived samples. Canadian Journal of Fisheries and Aquatic Sciences, 69, 1698–1709. doi: 10.1139/F2012-092

688. Ruggeri, P., Splendiani, A., Di Muri, C., Fioravanti, T., Santojanni, A., Leonori, I., … Caputo Barucchi, V. (2016). Coupling demographic and genetic variability from archived collections of European anchovy (Engraulis encrasicolus). PLoS ONE, 11, e0151507. doi: 10.1371/journal.pone.0151507

689. Ruokonen, M., Aarvak, T., Chesser, R. K., Lundqvist, A. C., & Merilä, J. (2010). Temporal increase in mtDNA diversity in a declining population. Molecular Ecology, 19, 2408–2417. doi: 10.1111/j.1365-294X.2010.04653.x

690. Ruscoe, W. A., Mather, P. B., & Wilson, J. (1998). Genetic structure in populations of Rattus sordidus in sugarcane-growing districts of Queensland, Australia. Journal of Mammalogy, 79, 612–623. doi: 10.2307/1382991

691. Ryabova, G. D., Klimonov, V. O., Afanas'ev, K. I., Rubtsova, G. A., Dovgopol, G. F., & Khodorevskaya, R. P. (2006). A comparison of the spawning migration, genetic and biological parameters of stellate sturgeon from the Volga population in 1985 and 1996. Russian Journal of Genetics, 42, 1180–1188. doi: 10.1134/S1022795406100103

692. Rychlik, T., Pakulski, T., & Krawczyk, A. (2011). Monitoring genetic variation in a conservation flock of Coloured Merino sheep based on blood group and protein polymorphism. Annals of Animal Science, 11, 41–52.

693. Saarinen, E. V, Austin, J. D., & Daniels, J. C. (2010). Genetic estimates of contemporary effective population size in an endangered butterfly indicate a possible role for genetic compensation. Evolutionary Applications, 3, 28–39. doi: 10.1111/j.1752-4571.2009.00096.x

694. Sackett, L. C., Collinge, S. K., & Martin, A. P. (2013). Do pathogens reduce genetic diversity of their hosts? Variable effects of sylvatic plague in black-tailed prairie dogs. Molecular Ecology, 22, 2441–2455. doi: 10.1111/mec.12270

695. Sacks, B. N., Statham, M. J., Perrine, J. D., Wisely, S. M., & Aubry, K. B. (2010). North American montane red foxes: Expansion, fragmentation, and the origin of the Sacramento Valley red fox. Conservation Genetics, 11, 1523–1539. doi: 10.1007/s10592-010-0053-4

696. Saijuntha, W., Sithithaworn, P., Chilton, N. B., Petney, T. N., Klinbunga, S., Satrawaha, R., … Andrews, R. H. (2009). Impact of temporal changes and host factors on the genetic structure of a population of Opisthorchis viverrini sensu lato in Khon Kaen Province (Thailand). Parasitology, 136, 1057–1063. doi: 10.1017/S0031182009006441

697. Salmona, J., Heller, R., Quéméré, E., & Chikhi, L. (2017). Climate change and human colonization triggered habitat loss and fragmentation in Madagascar. Molecular Ecology, 26, 5203–5222. doi: 10.1111/mec.14173

698. Sánchez-Montes, G., Wang, J., Ariño, A. H., Vizmanos, J. L., & Martínez-Solano, I. (2017). Reliable effective number of breeders/adult census size ratios in seasonal-breeding species: Opportunity for integrative demographic inferences based on capture–mark–recapture data and multilocus genotypes. Ecology and Evolution, 7, 10301–10314. doi: 10.1002/ece3.3387

699. Sanchez, J. A., La-Spina, M., Guirao, P., & Cánovas, F. (2013). Inferring the population structure of Myzus persicae in diverse agroecosystems using microsatellite markers. Bulletin of Entomological Research, 103, 473–484. doi: 10.1017/S0007485313000059

700. Sattler, R. L., Willoughby, J. R., & Swanson, B. J. (5AD). Decline of heterozygosity in a large but isolated population: A 45-year examination of moose genetic diversity on Isle Royale. PeerJ, 2017, e3584. doi: 10.7717/peerj.3584

701. Savage, W. K., Fremier, A. K., & Shaffer, H. B. (2010). Landscape genetics of alpine Sierra Nevada salamanders reveal extreme population subdivision in space and time. Molecular Ecology, 19, 3301–3314. doi: 10.1111/j.1365-294X.2010.04718.x

702. Savary, R., Dufresnes, C., Champigneulle, A., Caudron, A., Dubey, S., Perrin, N., & Fumagalli, L. (2017). Stocking activities for the Arctic charr in Lake Geneva: Genetic effects in space and time. Ecology and Evolution, 7, 5201–5211. doi: 10.1002/ece3.3073

703. Scheu, A., Powell, A., Bollongino, R., Vigne, J. D., Tresset, A., Çakirlar, C., … Burger, J. (2015). The genetic prehistory of domesticated cattle from their origin to the spread across Europe. BMC Genetics, 16, 1–11. doi: 10.1186/s12863-015-0203-2

704. Schmid, S., Neuenschwander, S., Pitteloud, C., Heckel, G., Pajkovic, M., Arlettaz, R., & Alvarez, N. (2018). Spatial and temporal genetic dynamics of the grasshopper Oedaleus decorus revealed by museum genomics. Ecology and Evolution, 8, 1480–1495. doi: 10.1002/ece3.3699

705. Schönhuth, S., Luikart, G., & Doadrio, I. (2003). Effects of a founder event and supplementary introductions on genetic variation in a captive breeding population of the endangered Spanish killifish. Journal of Fish Biology, 63, 1538–1551. doi: 10.1111/j.1095-8649.2003.00265.x

706. Schoville, S. D., Lam, A. W., & Roderick, G. K. (2012). A range-wide genetic bottleneck overwhelms contemporary landscape factors and local abundance in shaping genetic patterns of an alpine butterfly (Lepidoptera: Pieridae: Colias behrii). Molecular Ecology, 21, 4242–4256. doi: 10.1111/j.1365-294X.2012.05696.x

707. Scraggs, E., Zanella, R., Wojtowicz, A., Taylor, J. F., Gaskins, C. T., Reeves, J. J., … Neibergs, H. L. (2014). Estimation of inbreeding and effective population size of full-blood wagyu cattle registered with the American Wagyu Cattle Association. Journal of Animal Breeding and Genetics, 131, 3–10. doi: 10.1111/jbg.12066

708. Scribner, K. T., Smith, M. H., Garrott, R. A., & Carpenter, L. H. (1991). Temporal, spatial and age-specific changes in genotypic composition of mule deer. Journal of Mammalogy, 72, 126–137. doi: doi.org/10.2307/1381987

709. Segelbacher, G., Manel, S., & Tomiuk, J. (2008). Temporal and spatial analyses disclose consequences of habitat fragmentation on the genetic diversity in capercaillie (Tetrao urogallus). Molecular Ecology, 17, 2356–2367. doi: 10.1111/j.1365-294X.2008.03767.x

710. Segelbacher, G., Strand, T. M., Quintela, M., Axelsson, T., Jansman, H. A. H., Koelewijn, H. P., & Höglund, J. (2014). Analyses of historical and current populations of black grouse in Central Europe reveal strong effects of genetic drift and loss of genetic diversity. Conservation Genetics, 15, 1183–1195. doi: 10.1007/s10592-014-0610-3

711. Sekino, M., Saitoh, K., Shimizu, D., Wada, T., Kamiyama, K., Gambe, S., … Aritaki, M. (2011). Genetic structure in species with shallow evolutionary lineages: A case study of the rare flatfish Verasper variegatus. Conservation Genetics, 12, 139–159. doi: 10.1007/s10592-010-0128-2

712. Semenova, A. V, Karpov, A. K., Andreeva, A. P., Rubtsova, G. A., & Afanas'ev, K. I. (2017). Temporal stability of the population genetic structure of the White Sea herring Clupea pallasii marisalbi. Russian Journal of Genetics, 53, 100–107. doi: 10.1134/S1022795416120127

713. Sepúlveda, F. A., & González, M. T. (2015). Patterns of genetic variation and life history traits of Zeuxapta seriolae infesting Seriola lalandi across the coastal and oceanic areas in the southeastern Pacific Ocean: Potential implications for aquaculture. Parasites and Vectors, 8, 1–10. doi: 10.1186/s13071-015-0892-4

714. Sepúlveda, F. A., & González, M. T. (2017). Spatio-temporal patterns of genetic variations in populations of yellowtail kingfish Seriola lalandi from the south-eastern Pacific Ocean and potential implications for its fishery management. Journal of Fish Biology, 90, 249–264. doi: 10.1111/jfb.13179

715. Serbezov, D., Jorde, P. E., Bernatchez, L., Olsen, E. M., & Asbjørn Vøllestad, L. (2012). Short-term genetic changes: Evaluating effective population size estimates in a comprehensively described brown trout (Salmo Trutta) population. Genetics, 191, 579–592. doi: 10.1534/genetics.111.136580

716. Serieys, L. E. K., Lea, A., Pollinger, J. P., Riley, S. P. D., & Wayne, R. K. (2015). Disease and freeways drive genetic change in urban bobcat populations. Evolutionary Applications, 8, 75–92. doi: 10.1111/eva.12226

717. Sharma, R., Arora, N., Goossens, B., Nater, A., Morf, N., Salmona, J., … Chikhi, L. (2012). Effective Population Size Dynamics and the Demographic Collapse of Bornean Orang-Utans. PLoS ONE, 7, e49429. doi: 10.1371/journal.pone.0049429

718. Shen, K. N., & Tzeng, W. N. (2007). Population genetic structure of the year-round spawning tropical eel, Anguilla reinhardtii, in Australia. Zoological Studies, 46, 441–453.

719. Shen, K. N., Jamandre, B. W., Hsu, C. C., Tzeng, W. N., & Durand, J. D. (2011). Plio-Pleistocene sea level and temperature fluctuations in the northwestern Pacific promoted speciation in the globally-distributed flathead mullet Mugil cephalus. BMC Evolutionary Biology, 11, 1–17. doi: 10.1186/1471-2148-11-83

720. Shephard, J. M., Ogden, R., Tryjanowski, P., Olsson, O., & Galbusera, P. (2013). Is population structure in the European white stork determined by flyway permeability rather than translocation history? Ecology and Evolution, 3, 4881–4895. doi: 10.1002/ece3.845

721. Shitova, M. V, Kochnev, A. A., Dolnikova, O. G., Kryukova, N. V, Malinina, T. V, & Pereverzev, A. A. (2017). Genetic diversity of the Pacific walrus (Odobenus rosmarus divergens) in the western part of the Chukchi Sea. Russian Journal of Genetics, 53, 242–251. doi: 10.1134/S1022795417020107

722. Shrimpton, J. M., & Heath, D. D. (2003). Census vs. effective population size in chinook salmon: Large- and small-scale environmental perturbation effects. Molecular Ecology, 12, 2571–2583. doi: 10.1046/j.1365-294X.2003.01932.x

723. Signorile, A. L., Lurz, P. W. W., Wang, J., Reuman, D. C., & Carbone, C. (2016). Mixture or mosaic? Genetic patterns in UK grey squirrels support a human-mediated “long-jump” invasion mechanism. Diversity and Distributions, 22, 566–577. doi: 10.1111/ddi.12424

724. Simard, F., Lehmann, T., Lemasson, J. J., Diatta, M., & Fontenille, D. (2000). Persistence of Anopheles arabiensis during the severe dry season conditions in Senegal: An indirect approach using microsatellite loci. Insect Molecular Biology, 9, 467–479. doi: 10.1046/j.1365-2583.2000.00210.x

725. Sindičić, M., Polanc, P., Gomerčić, T., Jelenčič, M., Huber, D., Trontelj, P., & Skrbinšek, T. (2013). Genetic data confirm critical status of the reintroduced Dinaric population of Eurasian lynx. Conservation Genetics, 14, 1009–1018. doi: 10.1007/s10592-013-0491-x

726. Siol, M., Bonnin, I., Olivieri, I., Prosperi, J. M., & Ronfort, J. (2007). Effective population size associated with self-fertilization: Lessons from temporal changes in allele frequencies in the selfing annual Medicago truncatula. Journal of Evolutionary Biology, 20, 2349–2360. doi: 10.1111/j.1420-9101.2007.01409.x

727. Sire, C., Durand, P., Pointier, J. P., & Théron, A. (2001). Genetic diversity of Schistosoma mansoni within and among individual hosts (Rattus rattus): Infrapopulation differentiation at microspatial scale. International Journal for Parasitology, 31, 1609–1616. doi: 10.1016/S0020-7519(01)00294-6

728. Sischo, D. R., Price, M. R., Pascua, M. A., & Hadfield, M. G. (2016). Genetic and Demographic Insights into the Decline of a Captive Population of the Endangered Hawaiian Tree Snail Achatinella fuscobasis (Achatinellinae). Pacific Science, 70, 133–141. doi: 10.2984/70.2.1

729. SkrbinŠek, T., JelenČiČ, M., Waits, L., Kos, I., Jerina, K., & Trontelj, P. (2012). Monitoring the effective population size of a brown bear (Ursus arctos) population using new single-sample approaches. Molecular Ecology, 21, 862–875. doi: 10.1111/j.1365-294X.2011.05423.x

730. Sloop, C. M., Ayres, D. R., & Strong, D. R. (2011). Spatial and temporal genetic structure in a hybrid cordgrass invasion. Heredity, 106, 547–556. doi: 10.1038/hdy.2010.63

731. Smilansky, V., & Lasker, H. R. (2014). Fine-scale genetic structure in the surface brooding Caribbean octocoral, Antillogorgia elisabethae. Marine Biology, 161, 853–861. doi: 10.1007/s00227-013-2385-3

732. Snegin, E. A. (2015). Temporal dynamics of the genetic structure and effective size of Bradybaena fruticum Müll. (Mollusca, Gastropoda, Pulmonata) populations in the south of the Central Russian Upland. Russian Journal of Ecology, 46, 260–266. doi: 10.1134/S1067413615020113

733. Snyder, M. R., & Stepien, C. A. (2017). Genetic patterns across an invasion’s history: a test of change versus stasis for the Eurasian round goby in North America. Molecular Ecology, 26, 1075–1090. doi: 10.1111/mec.13997

734. Solorzano, C. D., Szalanski, A. L., Owens, C. B., & Dayton Steelman, C. (2010). Genetic diversity of Aedes vexans (Diptera, Culicidae) from New Orleans: Pre-and post-katrina. Biochemical Genetics, 48, 711–726. doi: 10.1007/s10528-010-9354-z

735. Song, N., Jia, N., Yanagimoto, T., Lin, L., & Gao, T. (2013). Genetic differentiation of Trachurus japonicus from the Northwestern Pacific based on the mitochondrial DNA control region. Mitochondrial DNA, 24, 705–712. doi: 10.3109/19401736.2013.773982

736. Sønstebø, J. H., Borgstrøm, R., & Heun, M. (2008). High genetic introgression in alpine brown trout (Salmo trutta L.) populations from Hardangervidda, Norway. Ecology of Freshwater Fish, 17, 174–183. doi: 10.1111/j.1600-0633.2007.00271.x

737. Sønstebø, J. H., Tollefsrud, M. M., Myking, T., Steffenrem, A., Nilsen, A. E., Edvardsen, M., … El-Kassaby, Y. A. (2018). Genetic diversity of Norway spruce (Picea abies (L.) Karst.) seed orchard crops: Effects of number of parents, seed year, and pollen contamination. Forest Ecology and Management, 411, 132–141. doi: 10.1016/j.foreco.2018.01.009

738. Sonsthagen, S. A., Wilson, R. E., & Underwood, J. G. (2017). Genetic implications of bottleneck effects of differing severities on genetic diversity in naturally recovering populations: An example from Hawaiian coot and Hawaiian gallinule. Ecology and Evolution, 7, 9925–9934. doi: 10.1002/ece3.3530

739. Souter, P., Willis, B. L., Bay, L. K., Caley, M. J., Muirhead, A., & Van Oppen, M. J. H. (2010). Location and disturbance affect population genetic structure in four coral species of the genus Acropora on the great Barrier Reef. *Marine Ecology Progress Series*, *416*, 35–45. doi: 10.3354/meps08740

740. Souza, K. R., Ribeiro, G., Silva dos Santos, C. G., de Lima, E. C., Melo, P. R. S., Reis, M. G., … Silva, L. K. (2013). Vector control measures failed to affect genetic structure of Aedes aegypti in a sentinel metropolitan area of Brazil. Acta Tropica, 128, 598–605. doi: 10.1016/j.actatropica.2013.09.002

741. Speller, C. F., Kooyman, B., Rodrigues, A. T., Langemann, E. G., Jobin, R. M., & Yang, D. Y. (2014). Assessing prehistoric genetic structure and diversity of North American elk (Cervus elaphus) populations in Alberta, Canada. Canadian Journal of Zoology, 92, 285–298. doi: 10.1139/cjz-2013-0253

742. Spurgin, L. G., Wright, D. J., van der Velde, M., Collar, N. J., Komdeur, J., Burke, T., & Richardson, D. S. (2014). Museum DNA reveals the demographic history of the endangered Seychelles warbler. Evolutionary Applications, 7, 1134–1143. doi: 10.1111/eva.12191

743. Stabile, J., Lipus, D., Maceda, L., Maltz, M., Roy, N., & Wirgin, I. (2016). Microsatellite DNA analysis of spatial and temporal population structuring of Phragmites australis along the Hudson River Estuary. Biological Invasions, 18, 2517–2529. doi: 10.1007/s10530-016-1157-7

744. Stadler, K., Koch, M., Bernhardt, K. G., & Greimler, J. (2010). Spatial arrangement and genetic structure in Gentianella aspera in a regional, local, and temporal context. Plant Systematics and Evolution, 286, 7–19. doi: 10.1007/s00606-010-0274-5

745. Steinfartz, S., Glaberman, S., Lanterbecq, D., Marquez, C., Rassmann, K., & Caccone, A. (2007). Genetic impact of a severe El Niño event on Galápagos marine iguanas (Amblyrhynchus cristatus). PLoS ONE, 2, e1285. doi: 10.1371/journal.pone.0001285

746. Stepien, C. A., Eddins, D. J., Snyder, M. R., & Marshall, N. T. (2018). Genetic change versus stasis over the time course of invasions: Trajectories of two concurrent, allopatric introductions of the eurasian ruffe. Aquatic Invasions, 13, 537–552. doi: 10.3391/ai.2018.13.4.11

747. Stepien, C. A., Snyder, M. R., & Knight, C. T. (2018). Genetic Divergence of Nearby Walleye Spawning Groups in Central Lake Erie: Implications for Management. North American Journal of Fisheries Management, 38, 783–793. doi: 10.1002/nafm.10176

748. Suárez, N. M., Betancor, E., Fregel, R., Rodríguez, F., & Pestano, J. (2012). Genetic signature of a severe forest fire on the endangered Gran Canaria blue chaffinch (Fringilla teydea polatzeki). Conservation Genetics, 13, 499–507. doi: 10.1007/s10592-011-0302-1

749. Sugimoto, T., Aramilev, V. V, Kerley, L. L., Nagata, J., Miquelle, D. G., & McCullough, D. R. (2014). Noninvasive genetic analyses for estimating population size and genetic diversity of the remaining Far Eastern leopard (Panthera pardus orientalis) population. Conservation Genetics, 15, 521–532. doi: 10.1007/s10592-013-0558-8

750. Sullivan, T. J., & Stepien, C. A. (2015). Temporal population genetic structure of yellow perch spawning groups in the lower Great Lakes. Transactions of the American Fisheries Society, 144, 211–226. doi: 10.1080/00028487.2014.982260

751. Sun, J. C., Cao, G. L., Ma, J., Chen, Y. F., & Han, L. Z. (2012). Comparative genetic structure within single-origin pairs of rice (Oryza sativa L.) landraces from in situ and ex situ conservation programs in Yunnan of China using microsatellite markers. Genetic Resources and Crop Evolution, 59, 1611–1623. doi: 10.1007/s10722-011-9786-2

752. Sun, X., & Hedgecock, D. (2017). Temporal genetic change in North American Pacific oyster populations suggests caution in seascape genetics analyses of high gene-flow species. *Marine Ecology Progress Series*, *565*, 79–93. doi: 10.3354/meps12009

753. Sun, Z., Pan, T., Wang, H., Pang, M., & Zhang, B. (2016). Yangtze River, an insignificant genetic boundary in tufted deer (Elaphodus cephalophus): The evidence from a first population genetics study. PeerJ, 4, e2654. doi: 10.7717/peerj.2654

754. Suni, S. S., Bronstein, J. L., & Brosi, B. J. (2014). Spatio-temporal genetic structure of a tropical bee species suggests high dispersal over a fragmented landscape. Biotropica, 46, 202–209. doi: 10.1111/btp.12084

755. Sutton, J. T., Robertson, B. C., & Jamieson, I. G. (2015). MHC variation reflects the bottleneck histories of New Zealand passerines. Molecular Ecology, 24, 362–373. doi: 10.1111/mec.13039

756. Swaegers, J., Mergeay, J., St-Martin, A., De Knijf, G., Larmuseau, M. H. D., & Stoks, R. (2015). Genetic signature of the colonisation dynamics along a coastal expansion front in the damselfly Coenagrion scitulum. Ecological Entomology, 40, 353–361. doi: 10.1111/een.12189

757. Swei, A., Brylski, P. V, Spencer, W. D., Dodd, S. C., & Patton, J. L. (2003). Hierarchical genetic structure in fragmented populations of the Little Pocket Mouse (Perognathus longimembris) in Southern California. Conservation Genetics, 4, 501–514. doi: 10.1023/A:1024768831808

758. Szczys, P., Nisbet, I. C. T., & Wingate, D. B. (2012). Conservation genetics of the Common Tern (Sterna hirundo) in the North Atlantic region; implications for the critically endangered population at Bermuda. Conservation Genetics, 13, 1039–1043. doi: 10.1007/s10592-012-0351-0

759. Takami, Y., Koshio, C., Ishii, M., Fujii, H., Hidaka, T., & Shimizu, I. (2004). Genetic diversity and structure of urban populations of Pieris butterflies assessed using amplified fragment length polymorphism. Molecular Ecology, 13, 245–258. doi: 10.1046/j.1365-294X.2003.02040.x

760. Tamaki, I., Kuze, T., Hirota, K., & Mizuno, M. (2016). Genetic variation and population demography of the landrace population of Camellia sinensis in Kasuga, Gifu Prefecture, Japan. Genetic Resources and Crop Evolution, 63, 823–831. doi: 10.1007/s10722-015-0286-7

761. Techer, M. A., Clémencet, J., Turpin, P., Volbert, N., Reynaud, B., & Delatte, H. (2015). Genetic characterization of the honeybee (Apis mellifera) population of Rodrigues Island, based on microsatellite and mitochondrial DNA. Apidologie, 46, 445–454. doi: 10.1007/s13592-014-0335-9

762. Terrell, K. A., Crosier, A. E., Wildt, D. E., O'Brien, S. J., Anthony, N. M., Marker, L., & Johnson, W. E. (2016). Continued decline in genetic diversity among wild cheetahs (Acinonyx jubatus) without further loss of semen quality. *Biological Conservation*, *200*, 192–199. doi: 10.1016/j.biocon.2016.05.034

763. Thalmann, O., Wegmann, D., Spitzner, M., Arandjelovic, M., Guschanski, K., Leuenberger, C., … Vigilant, L. (2011). Historical sampling reveals dramatic demographic changes in western gorilla populations. BMC Evolutionary Biology, 11, 1–10. doi: 10.1186/1471-2148-11-85

764. Thaulow, J., Borgstrøm, R., & Heun, M. (2013). Brown trout population structure highly affected by multiple stocking and river diversion in a high mountain national park. Conservation Genetics, 14, 145–158. doi: 10.1007/s10592-012-0438-7

765. Thériault, V., Bernatchez, L., & Dodson, J. J. (2007). Mating system and individual reproductive success of sympatric anadromous and resident brook charr, Salvelinus fontinalis, under natural conditions. Behavioral Ecology and Sociobiology, 62, 51–65. doi: 10.1007/s00265-007-0437-8

766. Therkildsen, N. O., Nielsen, E. E., Swain, D. P., & Pedersen, J. S. (2010). Large effective population size and temporal genetic stability in Atlantic cod (Gadus morhua) in the southern Gulf of St.Lawrence. Canadian Journal of Fisheries and Aquatic Sciences, 67, 1585–1595. doi: 10.1139/F10-084

767. Thiele, E. A., Corrêa-Oliveira, G., Gazzinelli, A., & Minchella, D. J. (2013). Elucidating the temporal and spatial dynamics of Biomphalaria glabrata genetic diversity in three Brazilian villages. Tropical Medicine and International Health, 18, 1164–1173. doi: 10.1111/tmi.12164

768. Thomann, M., Imbert, E., Engstrand, R. C., & Cheptou, P. O. (2015). Contemporary evolution of plant reproductive strategies under global change is revealed by stored seeds. Journal of Evolutionary Biology, 28, 766–778. doi: 10.1111/jeb.12603

769. Thormann, I., Reeves, P., Thumm, S., Reilley, A., Engels, J. M. M., Biradar, C. M., … Richards, C. M. (2017). Genotypic and phenotypic changes in wild barley (Hordeum vulgare subsp. spontaneum) during a period of climate change in Jordan. Genetic Resources and Crop Evolution, 64, 1295–1312. doi: 10.1007/s10722-016-0437-5

770. Tison, J. L., Blennow, V., Palkopoulou, E., Gustafsson, P., Roos, A., & Dalén, L. (2015). Population structure and recent temporal changes in genetic variation in Eurasian otters from Sweden. Conservation Genetics, 16, 371–384. doi: 10.1007/s10592-014-0664-2

771. Tokarska, M., KawaŁko, A., WÓjcik, J. M., & Pertoldi, C. (2009). Genetic variability in the European bison (Bison bonasus) population from Białowieża forest over 50 years. Biological Journal of the Linnean Society, 97, 801–809. doi: 10.1111/j.1095-8312.2009.01203.x

772. Tranchida-Lombardo, V., Roy, M., Bugot, E., Santoro, G., Püttsepp, Ü., Selosse, M., & Cozzolino, S. (2010). Spatial repartition and genetic relationship of green and albino individuals in mixed populations of Cephalanthera orchids. Plant Biology, 12, 659–667. doi: 10.1111/j.1438-8677.2009.00252.x

773. Triest, L., & Fénart, S. (2014). Clonal diversity and spatial genetic structure of Potamogeton pectinatus in managed pond and river populations. Hydrobiologia, 737, 145–161. doi: 10.1007/s10750-013-1583-9

774. Tripp-Valdez, M. A., García de León, F. J., Ortega-García, S., Lluch-Cota, D., López-Martínez, J., & Cruz, P. (2010). Population genetic structure of dolphinfish (Coryphaena hippurus) in the Gulf of California, using microsatellite loci. Fisheries Research, 105, 172–177. doi: 10.1016/j.fishres.2010.03.023

775. Troupin, D., Nathan, R., & Vendramin, G. G. (2006). Analysis of spatial genetic structure in an expanding Pinus halepensis population reveals development of fine-scale genetic clustering over time. Molecular Ecology, 15, 3617–3630. doi: 10.1111/j.1365-294X.2006.03047.x

776. Tseng, M. C., Kao, H. W., Hung, Y. H., & Lee, T. L. (2012). A study of genetic variations, population size, and population dynamics of the catadromous Japanese eel Anguilla japonica (Pisces) in northern Taiwan. Hydrobiologia, 683, 203–216. doi: 10.1007/s10750-011-0958-z

777. Tseng, M. C., Tzeng, W. N., & Lee, S. C. (2006). Population genetic structure of the Japanese eel Anguilla japonica in the northwest Pacific Ocean: Evidence of non-panmictic populations. *Marine Ecology Progress Series*, *308*, 221–230. doi: 10.3354/meps308221

778. Tseng, M., Tzeng, W., & Lee, S. (2003). Historical Decline in the Japanese Eel Anguilla japonica in Northern Taiwan Inferred from Temporal Genetic Variations. Zoological Studies, 42, 556–563.

779. Tzika, A. C., Remy, C., Gibson, R., & Milinkovitch, M. C. (2009). Molecular genetic analysis of a captive-breeding program: The vulnerable endemic Jamaican yellow boa. Conservation Genetics, 10, 69–77. doi: 10.1007/s10592-008-9519-z

780. Ueno, S., Tomaru, N., Yoshimaru, H., Manabe, T., & Yamamoto, S. (2002). Size-class differences in genetic structure and individual distribution of Camellia japonica L. in a Japanese old-growth evergreen forest. Heredity, 89, 120–126. doi: 10.1038/sj.hdy.6800111

781. Underwood, J. N., Richards, Z. T., Miller, K. J., Puotinen, M. L., & Gilmour, J. P. (2018). Genetic signatures through space, time and multiple disturbances in a ubiquitous brooding coral. Molecular Ecology, 27, 1586–1602. doi: 10.1111/mec.14559

782. Uzans, A. J., Lucas, Z., McLeod, B. A., & Frasier, T. R. (2015). Small Ne of the Isolated and Unmanaged Horse Population on Sable Island. Heredity, 106, 660–665. doi: 10.1093/jhered/esv051

783. Valentin, A. E., Power, D., & Sévigny, J. M. (2015). Understanding recruitment patterns of historically strong juvenile year classes in redfish (Sebastes spp.): The importance of species identity, population structure, and juvenile migration. Canadian Journal of Fisheries and Aquatic Sciences, 72, 774–784. doi: 10.1139/cjfas-2014-0149

784. Valenzuela, L. O., Sironi, M., Rowntree, V. J., & Seger, J. (2009). Isotopic and genetic evidence for culturally inherited site fidelity to feeding grounds in southern right whales (Eubalaena australis). Molecular Ecology, 18, 782–791. doi: 10.1111/j.1365-294X.2008.04069.x

785. Valiente, A. G., Beall, E., & Garcia-Vazquez, E. (2010). Population genetics of south European Atlantic salmon under global change. Global Change Biology, 16, 36–47. doi: 10.1111/j.1365-2486.2009.01922.x

786. Vallianatos, M., Lougheed, S. C., & Boag, P. T. (2002). Conservation genetics of the loggerhead shrike (Lanius ludovicianus) in central and eastern North America. Conservation Genetics, 3, 1–13. doi: 10.1023/A:1014232326576

787. Valtonen, M., Palo, J. U., Aspi, J., Ruokonen, M., Kunnasranta, M., & Nyman, T. (2014). Causes and consequences of fine-scale population structure in a critically endangered freshwater seal. *BMC Ecology*, *14*, 1–14. doi: 10.1186/1472-6785-14-22

788. Valtonen, M., Palo, J. U., Ruokonen, M., Kunnasranta, M., & Nyman, T. (2012). Spatial and temporal variation in genetic diversity of an endangered freshwater seal. Conservation Genetics, 13, 1231–1245. doi: 10.1007/s10592-012-0367-5

789. van den Broeck, F., Maes, G. E., Larmuseau, M. H. D., Rollinson, D., Sy, I., Faye, D., … Huyse, T. (2015). Reconstructing colonization dynamics of the human parasite schistosoma mansoni following anthropogenic environmental changes in Northwest Senegal. PLoS Neglected Tropical Diseases, 9, e0003998. doi: 10.1371/journal.pntd.0003998

790. Van Der Valk, T., Sandoval-Castellanos, E., Caillaud, D., Ngobobo, U., Binyinyi, E., Nishuli, R., … Guschanski, K. (2018). Significant loss of mitochondrial diversity within the last century due to extinction of peripheral populations in eastern gorillas. Scientific Reports, 8, 1–10. doi: 10.1038/s41598-018-24497-7

791. van Doornik, D. M., Berejikian, B. A., Campbell, L. A., & Volk, E. C. (2010). The effect of a supplementation program on the genetic and life history characteristics of an oncorhynchus mykiss population. Canadian Journal of Fisheries and Aquatic Sciences, 67, 1449–1458. doi: 10.1139/F10-073

792. van Doornik, D. M., Eddy, D. L., Waples, R. S., Boe, S. J., Hoffnagle, T. L., Berntson, E. A., & Moran, P. (2013). Genetic Monitoring of Threatened Chinook Salmon Populations: Estimating Introgression of Nonnative Hatchery Stocks and Temporal Genetic Changes. North American Journal of Fisheries Management, 33, 693–706. doi: 10.1080/02755947.2013.790861

793. van Doornik, D. M., Waples, R., Baird, M. C., Moran, P., & Berntson, E. A. (2011). Genetic monitoring reveals genetic stability within and among threatened Chinook salmon populations in the Salmon River, Idaho. North American Journal of Fisheries Management, 31, 96–105. doi: 10.1080/02755947.2011.562443

794. Vandergast, A. G., Wood, D. A., Thompson, A. R., Fisher, M., Barrows, C. W., & Grant, T. J. (2016). Drifting to oblivion? Rapid genetic differentiation in an endangered lizard following habitat fragmentation and drought. Diversity and Distributions, 22, 344–357. doi: 10.1111/ddi.12398

795. Varela, M. A., Martínez-Lage, A., & González-Tizón, A. M. (2009). Temporal genetic variation of microsatellite markers in the razor clam ensis arcuatus (bivalvia: Pharidae). Journal of the Marine Biological Association of the United Kingdom, 89, 1703–1707. doi: 10.1017/S0025315409000812

796. Varela, M. A., Martínez-Lage, A., & González-Tizón, A. M. (2012). Genetic heterogeneity in natural beds of the razor clam Ensis siliqua revealed by microsatellites. Journal of the Marine Biological Association of the United Kingdom, 92, 171–177. doi: 10.1017/S0025315411000646

797. Vasemägi, A., Gross, R., Paaver, T., Koljonen, M. L., & Nilsson, J. (2005). Extensive immigration from compensatory hatchery releases into wild Atlantic salmon population in the Baltic sea: Spatio-temporal analysis over 18 years. Heredity, 95, 76–83. doi: 10.1038/sj.hdy.6800693

798. Vasileiadou, K., Pavloudi, C., Camisa, F., Tsikopoulou, I., Fragopoulou, N., Kotoulas, G., & Arvanitidis, C. (2018). Genetic diversity of Nephtys hombergii (Phyllodocida, Polychaeta) associated with environmental factors in a highly fluctuating ecosystem. Journal of the Marine Biological Association of the United Kingdom, 98, 777–789. doi: 10.1017/S0025315416001910

799. Vega, R., Vázquez-Domínguez, E., White, T. A., Valenzuela-Galván, D., & Searle, J. B. (2017). Population genomics applications for conservation: the case of the tropical dry forest dweller Peromyscus melanophrys. Conservation Genetics, 18, 313–326. doi: 10.1007/s10592-016-0907-5

800. Velando, A., Barros, A., & Moran, P. (2015). Heterozygosity-fitness correlations in a declining seabird population. Molecular Ecology, 24, 1007–1018. doi: 10.1111/mec.13092

801. Verbylaite, R., Pliura, A., Lygis, V., Suchockas, V., Jankauskiene, J., & Labokas, J. (2017). Genetic diversity and its spatial distribution in self-regenerating norway spruce and scots pine stands. Forests, 8, 1–17. doi: 10.3390/f8120470

802. Verheyen, G. R., Kempenaers, B., Adriaensen, F., Van Den Broeck, M., Matthysen, E., Van Broeckhoven, C., & Dhondt, A. A. (1995). The genetic structure of Parus caeruleus (Blue tit) populations as revealed by minisatellite single locus probes. Heredity, 75, 571–577. doi: 10.1038/hdy.1995.176

803. Vialatte, A., Plantegenest, M., Simon, J. C., & Dedryver, C. A. (2007). Farm-scale assessment of movement patterns and colonization dynamics of the grain aphid in arable crops and hedgerows. Agricultural and Forest Entomology, 9, 337–346. doi: 10.1111/j.1461-9563.2007.00347.x

804. Vidya, T. N. C., Balmforth, Z., Le Roux, A., & Cherry, M. I. (2009). Genetic structure, relatedness and helping behaviour in the yellow mongoose in a farmland and a natural habitat. Journal of Zoology, 278, 57–64. doi: 10.1111/j.1469-7998.2009.00551.x

805. Vignieri, S. N. (2010). The genetic effects of ecological disturbance: Flooding in jumping mice. The American Naturalist, 175, 126–135. doi: 10.1086/648606

806. Vigouroux, Y., Mariac, C., de Mita, S., Pham, J. L., Gérard, B., Kapran, I., … Bezançon, G. (2011). Selection for earlier flowering crop associated with climatic variations in the Sahel. PLoS ONE, 6, e19563. doi: 10.1371/journal.pone.0019563

807. Vilas, R., Paniagua, E., & Sanmartín, M. L. (2003). Genetic variation within and among infrapopulations of the marine digenetic trematode Lecithochirium fusiforme. Parasitology, 126, 465–472. doi: 10.1017/S0031182003003081

808. Villanova, G. V, Vera, M., Brancolini, F., Díaz, J., Martinez, P., & Arranz, S. E. (2018). Species assignment and population genetic studies of Gran Paraná pejerrey (Odontesthes sp., Atheriniformes, Atherinopsidae) from La Plata Basin in South America. Hydrobiologia, 819, 243–257. doi: 10.1007/s10750-018-3643-7

809. Virgilio, M., & Abbiati, M. (2006). Temporal changes in the genetic structure of intertidal populations of Hediste diversicolor (Polychaeta: Nereididae). Journal of Sea Research, 56, 53–58. doi: 10.1016/j.seares.2006.03.008

810. Vostrá-Vydrová, H., Vostrý, L., Hofmanová, B., Krupa, E., Veselá, Z., & Schmidová, J. (2016). Genetic diversity within and gene flow between three draught horse breeds using genealogical information. Czech Journal of Animal Science, 61, 462–472. doi: 10.17221/91/2015-CJAS

811. Wachirachaikarn, A., Prakoon, W., Nguyen, T. T. T., Prompakdee, W., & Na-Nakorn, U. (2011). Loss of genetic variation of Phalacronotus bleekeri (Günther, 1864) in the hatchery stocks revealed by newly developed microsatellites. Aquaculture, 321, 298–302. doi: 10.1016/j.aquaculture.2011.08.019

812. Walker, C. W., Vilá, C., Landa, A., Lindén, M., & Ellegren, H. (2001). Genetic variation and population structure in Scandinavian wolverine (Gulo gulo) populations. *Molecular Ecology*, *10*, 53–63. doi: 10.1046/j.1365-294x.2001.01184.x

813. Wang, I. J., & Shaffer, H. B. (2017). Population genetic and field-ecological analyses return similar estimates of dispersal over space and time in an endangered amphibian. Evolutionary Applications, 10, 630–639. doi: 10.1111/eva.12479

814. Wang, K.-S. (2003). Genetic diversity and temporal genetic structure in European beech (Fagus sylvatica L.). Silvae Genetica, 52, 100–106.

815. Wang, M., Lang, G., & Schreiber, A. (2002). Temporal shifts of DNA-microsatellite allele profiles in roe deer (Capreolus capreolus L.) within three decades. Journal of Zoological Systematics and Evolutionary Research, 40, 232–236. doi: 10.1046/j.1439-0469.2002.00198.x

816. Wang, N., Li, F., Chen, B., Xu, K., Yan, G., Qiao, J., … Wu, X. (2014). Genome-wide investigation of genetic changes during modern breeding of Brassica napus. Theoretical and Applied Genetics, 127, 1817–1829. doi: 10.1007/s00122-014-2343-6

817. Wang, S., Leroy, G., Malm, S., Lewis, T., Strandberg, E., & Fikse, W. F. (2017). Merging pedigree databases to describe and compare mating practices and gene flow between pedigree dogs in France, Sweden and the UK. Journal of Animal Breeding and Genetics, 134, 152–161. doi: 10.1111/jbg.12242

818. Wang, Z. S., Liu, H., Wei, N., Xu, W. X., & An, S. Q. (2010). Contribution of progeny cohorts to the restoration of genetic diversity in the post-harvest dragon spruce (Picea asperata) stands. Forestry, 83, 307–314. doi: 10.1093/forestry/cpq013

819. Waples, R. S., Elz, A., Arnsberg, B. D., Faulkner, J. R., Hard, J. J., Timmins-Schiffman, E., & Park, L. K. (2017). Human-mediated evolution in a threatened species? Juvenile life-history changes in Snake River salmon. Evolutionary Applications, 10, 667–681. doi: 10.1111/eva.12468

820. Waples, R. S., Scribner, K. T., Moore, J. A., Draheim, H. M., Etter, D., & Boersen, M. (2018). Accounting for age structure and spatial structure in eco-evolutionary analyses of a large, mobile vertebrate. Heredity, 109, 709–723. doi: 10.1093/jhered/esy018

821. Ward, R., Bowers, K., Hensley, R., Mobley, B., & Belouski, E. (2007). Genetic variability in spotted seatrout (Cynoscion nebulosus), determined with microsatellite DNA markers. Fishery Bulletin, 105, 197–206.

822. Was, A., Gosling, E., & Hoarau, G. (2010). Microsatellite analysis of plaice (Pleuronectes platessa L.) in the NE Atlantic: Weak genetic structuring in a milieu of high gene flow. Marine Biology, 157, 447–462. doi: 10.1007/s00227-009-1331-x

823. Waters, C. D., Hard, J. J., Brieuc, M. S. O., Fast, D. E., Warheit, K. I., Waples, R. S., … Naish, K. A. (2015). Effectiveness of managed gene flow in reducing genetic divergence associated with captive breeding. Evolutionary Applications, 8, 956–971. doi: 10.1111/eva.12331

824. Watts, P. C., Hamilton, J. G. C., Ward, R. D., Noyes, H. A., Souza, N. A., Kemp, S. J., … Maingon, R. D. C. (2005). Male sex pheromones and the phylogeographic structure of the Lutzomyia longipalpis species complex (Diptera: Psychodidae) from Brazil and Venezuela. American Journal of Tropical Medicine and Hygiene, 73, 734–743. doi: 10.4269/ajtmh.2005.73.734

825. Watts, P. C., Kay, S. M., Wolfenden, D., Fox, C. J., Geffen, A. J., Kemp, S. J., & Nash, R. D. M. (2010). Temporal patterns of spatial genetic structure and effective population size in European plaice (Pleuronectes platessa) along the west coast of Scotland and in the Irish Sea. ICES Journal of Marine Science, 67, 607–616. doi: 10.1093/icesjms/fsp274

826. Watts, P. C., Saccheri, I. J., Kemp, S. J., & Thompson, D. J. (2007). Effective population sizes and migration rates in fragmented populations of an endangered insect (Coenagrion mercuriale: Odonata). Journal of Animal Ecology, 76, 790–800. doi: 10.1111/j.1365-2656.2007.01249.x

827. Weber, D. S., Stewart, B. S., & Lehman, N. (2004). Genetic Consequences of a Severe Population Bottleneck in the Guadalupe fur Seal (Arctocephalus townsendi). Heredity, 95, 144–153. doi: 10.1093/jhered/esh018

828. Weber, D. S., Stewart, B. S., Garza, J. C., & Lehman, N. (2000). An empirical genetic assessment of the severity of the northern elephant seal population bottleneck. Current Biology, 10, 1287–1290. doi: 10.1016/S0960-9822(00)00759-4

829. Webster, J. P., Davies, C. M., Ndamba, J., Noble, L. R., Jones, C. S., & Woolhouse, M. E. J. (2001). Spatio-temporal genetic variability in the schistosome intermediate host Biomphalaria pfeifferi. Annals of Tropical Medicine and Parasitology, 95, 515–527. doi: 10.1080/00034980120072239

830. Wei, X., & Jiang, M. (2012). Limited genetic impacts of habitat fragmentation in an “old rare” relict tree, Euptelea pleiospermum (Eupteleaceae). Plant Ecology, 213, 909–917. doi: 10.1007/s11258-012-0052-2

831. Weider, L. J., Lampert, W., Wessels, M., Colbourne, J. K., & Limburg, P. (1997). Long-term genetic shifts in microcrustacean egg bank associated with anthropogenic changes in the Lake Constance ecosystem. Proceedings of the Royal Society B, 264, 1613–1618. doi: 10.1098/rspb.1997.0225

832. Welch, A. J., Wiley, A. E., James, H. F., Ostrom, P. H., Stafford, T. W., & Fleischer, R. C. (2012). Ancient DNA reveals genetic stability despite demographic decline: 3,000 years of population history in the endemic hawaiian petrel. Molecular Biology and Evolution, 29, 3729–3740. doi: 10.1093/molbev/mss185

833. Wellmann, R., Hartwig, S., & Bennewitz, J. (2012). Optimum contribution selection for conserved populations with historic migration. Genetics Selection Evolution, 44, 1–11. doi: 10.1186/1297-9686-44-34

834. Wennerström, L., Ryman, N., Tison, J. L., Hasslow, A., Dalén, L., & Laikre, L. (2016). Genetic landscape with sharp discontinuities shaped by complex demographic history in moose (Alces alces). Journal of Mammalogy, 97, 1–13. doi: 10.1093/jmammal/gyv146

835. White, D. J., Watts, C., Allwood, J., Prada, D., Stringer, I., Thornburrow, D., & Buckley, T. R. (2017). Population history and genetic bottlenecks in translocated Cook Strait giant weta, Deinacrida rugosa: recommendations for future conservation management. Conservation Genetics, 18, 411–422. doi: 10.1007/s10592-016-0916-4

836. White, N. E., Bunce, M., Mawson, P. R., Dawson, R., Saunders, D. A., & Allentoft, M. E. (2014). Identifying conservation units after large-scale land clearing: A spatio-temporal molecular survey of endangered white-tailed black cockatoos (Calyptorhynchus spp.). Diversity and Distributions, 20, 1208–1220. doi: 10.1111/ddi.12202

837. White, S. E., Kennedy, P. K., & Kennedy, M. L. (1998). Temporal genetic variation in the raccoon, Procyon lotor. Journal of Mammalogy, 79, 747–754. doi: 10.2307/1383085

838. Whiteley, A. R., Coombs, J. A., O’Donnell, M. J., Nislow, K. H., & Letcher, B. H. (2017). Keeping things local: Subpopulation Nb and Ne in a stream network with partial barriers to fish migration. Evolutionary Applications, 10, 348–365. doi: 10.1111/eva.12454

839. Wickliffe, J. K., Dunina-Barkovskaya, Y. V, Gaschak, S. P., Rodgers, B. E., Chesser, R. K., Bondarkov, M., & Baker, R. J. (2006). Variation in mitochondrial DNA control region haplotypes in populations of the bank vole, Clethrionomys glareolus, living in the chernobyl environment, Ukraine. Environmental Toxicology and Chemistry, 25, 503–508. doi: 10.1897/05-327R.1

840. Wildes, S. L., Vollenweider, J. J., Nguyen, H. T., & Guyon, J. R. (2011). Genetic variation between outer-coastal and fjord populations of pacific herring (Clupea Pallasii) in the eastern Gulf of Alaska. Fishery Bulletin, 109, 382–393.

841. Willems, H., Welte, J., Hecht, W., & Reiner, G. (2016). Temporal variation of the genetic diversity of a German red deer population between 1960 and 2012. European Journal of Wildlife Research, 62, 277–284. doi: 10.1007/s10344-016-0999-8

842. Williams, C. L., Blejwas, K., Johnston, J. J., & Jaeger, M. M. (2003). Temporal genetic variation in a coyote (Canis latrans) population experiencing high turnover. Journal of Mammalogy, 84, 177–184. doi: 10.1644/1545-1542(2003)084<0177:TGVIAC>2.0.CO;2

843. Williams, D. E., Miller, M. W., & Baums, I. B. (2014). Cryptic changes in the genetic structure of a highly clonal coral population and the relationship with ecological performance. Coral Reefs, 33, 595–606. doi: 10.1007/s00338-014-1157-y

844. Williamson, K. S., & May, B. (2005). Homogenization of Fall-Run Chinook Salmon Gene Pools in the Central Valley of California, USA. North American Journal of Fisheries Management, 25, 993–1009. doi: 10.1577/m04-136.1

845. Wilson, A. S., Marra, P. P., & Fleischer, R. C. (2012). Temporal patterns of genetic diversity in Kirtland's warblers (Dendroica kirtlandii), the rarest songbird in North America. BMC Ecology, 12, 1–11. doi: 10.1186/1472-6785-12-8

846. Wingen, L. U., Orford, S., Goram, R., Leverington-Waite, M., Bilham, L., Patsiou, T. S., … Griffiths, S. (2014). Establishing the A. E. Watkins landrace cultivar collection as a resource for systematic gene discovery in bread wheat. Theoretical and Applied Genetics, 127, 1831–1842. doi: 10.1007/s00122-014-2344-5

847. Wojnicka-Półtorak, A., Celiński, K., & Chudzińska, E. (2016). Temporal dynamics in the genetic structure of a natural population of Picea abies. Biologia (Poland), 71, 875–884. doi: 10.1515/biolog-2016-0109

848. Wojnicka-Półtorak, A., Prus-Głowacki, W., Celiński, K., & Korczyk, A. (2013). Genetic aspects of age dynamics of a natural Picea abies (L.) Karst. population in the Białowieża Primeval Forest, Poland. New Forests, 44, 811–825. doi: 10.1007/s11056-013-9367-7

849. Wojnicka-Półtorak, A., Wachowiak, W., Prus-Głowacki, W., Celiński, K., & Korczyk, A. (2014). Genetic heterogeneity in age classes of naturally regenerated old growth forest of Picea abies (L.) Karst. Silvae Genetica, 63, 185–190. doi: 10.1515/sg-2014-0024

850. Wommack, E. A., Dawson, R. D., Shrimpton, J. M., & Bowie, R. C. K. (2015). Changes in population size and genetic diversity of a raptor species occurring in the boreal forest of Saskatchewan. Conservation Genetics, 16, 535–547. doi: 10.1007/s10592-014-0677-x

851. Wondji, C., Simard, F., Lehmann, T., Fondjo, E., Samè-Ekobo, A., & Fontenille, D. (2005). Impact of insecticide-treated bed nets implementation on the genetic structure of Anopheles arabiensis in an area of irrigated rice fields in the Sahelian region of Cameroon. Molecular Ecology, 14, 3683–3693. doi: 10.1111/j.1365-294X.2005.02699.x

852. Wood, J. L. A., Belmar-Lucero, S., Hutchings, J. A., & Fraser, D. J. (2014). Relationship of habitat variability to population size in a stream fish. Ecological Applications, 24, 1085–1100. doi: 10.1890/13-1647.1

853. Woxvold, I. A., Adcock, G. J., & Mulder, R. A. (2006). Fine-scale genetic structure and dispersal in cooperatively breeding apostlebirds. Molecular Ecology, 15, 3139–3146. doi: 10.1111/j.1365-294X.2006.03009.x

854. Wu, M., Li, B., Liu, P., Weng, Q., Zhan, J., & Chen, Q. (2017). Genetic analysis of Phytophthora sojae populations in Fujian, China. Plant Pathology, 66, 1182–1190. doi: 10.1111/ppa.12666

855. Wu, Z., Xu, Q., Zhu, J., Dai, X., & Xu, L. (2014). Genetic population structure of the bigeye tuna Thunnus obesus in the central Pacific Ocean based on mtDNA Cytb sequences. Fisheries Science, 80, 415–426. doi: 10.1007/s12562-014-0712-3

856. Wynne, I. R., Loxdale, H. D., Brookes, C. P., & Woiwod, I. P. (2003). Genetic structure of fragmented November moth (Lepidoptera: Geometridae) populations in farmland. Biological Journal of the Linnean Society, 78, 467–477. doi: 10.1046/j.0024-4066.2002.00155.x

857. Xenikoudakis, G., Ersmark, E., Tison, J. L., Waits, L., Kindberg, J., Swenson, J. E., & Dalén, L. (2015). Consequences of a demographic bottleneck on genetic structure and variation in the Scandinavian brown bear. Molecular Ecology, 24, 3441–3454. doi: 10.1111/mec.13239

858. Yamamoto, S., Uchida, K., Sato, T., Katsura, K., & Takasawa, T. (2007). Population genetic structure and effective population size of ayu (Plecoglossus altivelis), an amphidromous fish. Journal of Fish Biology, 70, 191–201. doi: 10.1111/j.1095-8649.2007.01396.x

859. Yamazaki, Y., Nakamura, T., Sasaki, M., Nakano, S., & Nishio, M. (2014). Decreasing genetic diversity in wild and captive populations of endangered Itasenpara bittering (Acheilognathus longipinnis) in the Himi region,

central Japan, and recommendations for conservation. Conservation Genetics, 15, 921–932. doi: 10.1007/s10592-014-0589-9

860. Yang, J., & Jiang, Z. (2011). Genetic diversity, population genetic structure and demographic history of Przewalski's gazelle (Procapra przewalskii): Implications for conservation. Conservation Genetics, 12, 1457–1468. doi: 10.1007/s10592-011-0244-7

861. Yang, S. Y., Han, M. J., Kang, L. F., Li, Z. W., Shen, Y. H., & Zhang, Z. (2014). Demographic history and gene flow during silkworm domestication. BMC Evolutionary Biology, 14, 1–8. doi: 10.1186/s12862-014-0185-0

862. Yannic, G., Basset, P., & Hausser, J. (2009). Chromosomal rearrangements and gene flow over time in an inter-specific hybrid zone of the Sorex araneus group. Heredity, 102, 616–625. doi: 10.1038/hdy.2009.19

863. Yu, J. N., Azuma, N., & Abe, S. (2012). Genetic differentiation between collections of hatchery and wild masu salmon (Oncorhynchus masou) inferred from mitochondrial and microsatellite DNA analyses. Environmental Biology of Fishes, 94, 259–271. doi: 10.1007/s10641-011-9869-0

864. Zalewski, A., Zalewska, H., Lunneryd, S. G., André, C., & Mikusiñski, G. (2016). Reduced genetic diversity and increased structure in American mink on the Swedish coast following invasive species control. PLoS ONE, 11, e0157972. doi: 10.1371/journal.pone.0157972

865. Zane, L., Marcato, S., Bargelloni, L., Bortolotto, E., Papetti, C., Simonato, M., … Patarnello, T. (2006). Demographic history and population structure of the Antarctic silverfish Pleuragramma antarcticum. Molecular Ecology, 15, 4499–4511. doi: 10.1111/j.1365-294X.2006.03105.x

866. Zane, L., Ostellari, L., Maccatrozzo, L., Bargelloni, L., Cuzin-Roudy, J., Buchholz, F., & Patarnello, T. (2000). Genetic differentiation in a pelagic crustacean (Meganyctiphanes norvegica: Euphausiacea) from the North East Atlantic and the Mediterranean Sea. Marine Biology, 136, 191–199. doi: 10.1007/s002270050676

867. Zanetti, E., de Marchi, M., Abbadi, M., & Cassandro, M. (2011). Variation of genetic diversity over time in local Italian chicken breeds undergoing in situ conservation. Poultry Science, 90, 2195–2201. doi: 10.3382/ps.2011-01527

868. Zaslavskaya, N. I., Vashchenko, M. A., & Zhadan, P. M. (2012). The genetic structure of populations of the sea urchin Strongylocentrotus intermedius from the northwestern Sea of Japan in connection with a shift in spawning time. Russian Journal of Marine Biology, 38, 325–338. doi: 10.1134/S1063074012040104

869. Zavodna, M., Abdelkrim, J., Pellissier, V., & Machon, N. (2015). A long-term genetic study reveals complex population dynamics of multiple-source plant reintroductions. *Biological Conservation*, *192*, 1–9. doi: 10.1016/j.biocon.2015.08.025

870. Zelenina, D. A., Shepetov, D. M., Volkov, A. A., Barmintseva, A. E., Melnikov, S. P., & Myuge, N. S. (2011). Population structure of beaked redfish (Sebastes mentella Travin, 1951) in the Irminger Sea and adjacent waters inferred from microsatellite data. Russian Journal of Genetics, 47, 1333–1344. doi: 10.1134/S1022795411110202

871. Zenboudji, S., Cheylan, M., Arnal, V., Bertolero, A., Leblois, R., Astruc, G., … Montgelard, C. (2016). Conservation of the endangered Mediterranean tortoise Testudo hermanni hermanni: The contribution of population genetics and historical demography. *Biological Conservation*, *195*, 279–291. doi: 10.1016/j.biocon.2016.01.007

872. Zhan, J., Mundt, C. C., & McDonald, B. A. (2001). Using restriction fragment length polymorphisms to assess temporal variation and estimate the number of ascospores that initiate epidemics in field populations of Mycosphaerella graminicola. Phytopathology, 91, 1011–1017. doi: 10.1094/PHYTO.2001.91.10.1011

873. Zhang, B., Li, M., Zhang, Z., Goossens, B., Zhu, L., Zhang, S., … Wei, F. (2007). Genetic viability and population history of the giant panda, putting an end to the "evolutionary dead end"? Molecular Biology and Evolution, 24, 1801–1810. doi: 10.1093/molbev/msm099

874. Zhang, J., Chen, T., Wang, Y., Chen, Q., Sun, B., Luo, Y., … Wang, X. (2018). Genetic diversity and domestication footprints of Chinese cherry [cerasus pseudocerasus (Lindl.) g.don] as revealed by nuclear microsatellites. Frontiers in Plant Science, 9, 1–13. doi: 10.3389/fpls.2018.00238

875. Zhang, K., Xiao, G., Wang, W., Jin, X., Song, W., & Gao, Y. (2015). Genetic variation analysis across six life periods in a natural population of the Chinese shrimp "Fenneropenaeus chinensis" in Bohai bay using SSR markers. Russian Journal of Marine Biology, 41, 10–16. doi: 10.1134/S1063074015010113

876. Zhang, L., Li, Y., Li, Y., Yang, Z., Li, Y., Wang, Y., … Bao, Z. (2018). A Population Genetic Analysis of Continuously Selected Chlamys farreri Populations. Journal of Ocean University of China, 17, 913–919. doi: 10.1007/s11802-018-3539-1

877. Zhang, S. M., Wang, D. Q., & Zhang, Y. P. (2003). Mitochondrial DNA variation, effective female population size and population history of the endangered Chinese sturgeon, Acipenser sinensis. Conservation Genetics, 4, 673–683. doi: 10.1023/B:COGE.0000006107.46111.bc

878. Zhang, Y., Zhou, A., Xi, Y. L., Sun, Q., Ning, L. F., Xie, P., … Xiang, X. L. (2018). Temporal patterns and processes of genetic differentiation of the Brachionus calyciflorus (Rotifera) complex in a subtropical shallow lake. Hydrobiologia, 807, 313–331. doi: 10.1007/s10750-017-3407-9

879. Zheng, W., Zou, L., & Han, Z. (2015). Genetic analysis of the populations of Japanese anchovy Engraulis japonicus from the Yellow Sea and East China Sea based on mitochondrial cytochrome b sequence. *Biochemical Systematics and Ecology*, *58*, 169–177. doi: 10.1016/j.bse.2014.12.007

880. Zhu, L., Hu, Y., Qi, D., Wu, H., Zhan, X., Zhang, Z., … Wei, F. (2013). Genetic consequences of historical anthropogenic and ecological events on giant pandas. Ecology, 94, 2346–2357. doi: 10.1890/12-1451.1

881. Zimmerman, M., Oddy, D., Stolen, E., Breininger, D., & Pruett, C. L. (2015). Microspatial sampling reveals cryptic influences on gene flow in a threatened mammal. Conservation Genetics, 16, 1403–1414. doi: 10.1007/s10592-015-0749-6

882. Zou, Z., Zhang, X., & Fernando, W. G. D. (2018). Distribution of mating-type alleles and genetic variability in field populations of Leptosphaeria maculans in western Canada. Journal of Phytopathology, 166, 438–447. doi: 10.1111/jph.12706
