# Supplementary material for: Global meta-analysis shows action is needed to halt genetic diversity loss
Source: Nature. 2025 Jan 29;638(8051):704–10. doi: 10.1038/s41586-024-08458-x (PMC11839457; doi:10.1038/s41586-024-08458-x)
Supplement: Supplementary file 7 — Web of Science advanced search string. [file 41586_2024_8458_MOESM7_ESM.pdf]

TS=(genetic* OR "effective population size" OR genom*) AND TS=(population*) AND TS=(NE OR temporal OR monitor* OR historic* OR museum OR archeolog* OR archaeolog* OR change* OR declin* OR grow* OR increas* OR decreas* OR bottleneck* OR expan* OR erosion) NOT TS=(patient OR cancer OR clinic* OR hospital OR hypertension OR microbiom*) NOT TS=(physics) NOT DT=(Meeting Abstract OR Correction OR Review OR Database Review OR News Item OR Retracted Publication OR Biographical Item OR Editorial Material OR Book Review OR Software Review OR Book Chapter) NOT WC=(Medical Laboratory Technology OR Endocrinology Metabolism OR Psychiatry OR Clinical Neurology OR Neurosciences OR Hematology OR Obstetrics Gynecology OR Cardiac Cardiovascular Systems OR Nutrition Dietetics OR Substance Abuse OR Geriatrics Gerontology OR Urology Nephrology OR Pediatrics OR Oncology OR Respiratory System OR Critical Care Medicine OR Ophthalmology OR Allergy OR Surgery OR Rheumatology OR Medicine General Internal OR Social Sciences Biomedical OR Chemistry Medicinal OR Gastroenterology Hepatology OR Optics OR Radiology Nuclear Medicine Medical Imaging OR Dermatology OR Cell Tissue Engineering OR Engineering Biomedical OR Microbiology OR Engineering Civil OR Engineering Industrial OR Engineering Manufacturing OR Engineering Electrical Electronic OR Engineering Chemical OR Instruments Instrumentation OR Physics Mathematical OR Engineering Mechanical OR Thermodynamics OR Physics Applied OR Computer Science Hardware Architecture OR Astronomy Astrophysics OR Telecommunications OR Nanoscience Nanotechnology OR Sport Sciences OR Automation Control Systems OR Otorhinolaryngology OR Health Care Sciences Services OR Medical Informatics OR Audiology Speech OR Medical Informatics OR Audiology Speech Language Pathology OR Education Scientific Disciplines OR Psychology Developmental OR Orthopedics) NOT SO=(“American Journal of Human Genetics” OR “Human Biology” OR “European Journal of Human Genetics” OR “Human Molecular Genetics” OR “Human Genetics” OR “Journal of Human Genetics”) NOT CF=(“Annual meeting of the society for integrative and comparative biology” OR “18th symposium of the society of population ecology” OR “9th meeting of the international organization of plant biosystematists IOPB” OR “International summit on evolutionary change in human altered environments” OR “28th annual meeting of the human biology association” OR “Annual meeting of the society for conservation biology” OR “ESF congen meeting on integrating population genetics and conservation biology” OR “3rd international dna fingerprinting conference” OR “Annual meeting of the society for experimental biology” OR “Annual symposium of the fisheries society of the british isles” OR “3rd international symposium on stock enhancement and sea ranching” OR “Annual meeting of the society for integrative and comparative biology SICB symposium on mangrove killifish an exemplar of integrative biology” OR “International conference on dynamics and conservation of genetic diversity in forest ecosystems” OR “50th anniversary annual symposium of the fisheries society of the british isles fsbi understanding fish populations” OR “Colloquium on in the light of evolution i” OR “20th lowell wakefield fisheries symposium” OR “5th international mycological congress” OR “Conference on genetics and population health” OR “9th international symposium on genetics in aquaculture” OR “6th international symposium on genetics in aquaculture” OR “Conference on pacific temperate conifers as introduced and native” OR “Ices nasco symposium on interactions between salmon culture and wild stocks of atlantic salmon the scientific and management issues” OR “Aga symposium on conservation and genetics of marine organisms” OR “Dna in forensics 2006” OR “Meeting on current knowledge and management of genetic resources” OR “Aga symposium on genetics of fragmented populations” OR “Evoworkshops 2006” OR

“Meeting on phylogeography hybridization and speciation” OR “Conference on extinction thresholds” OR “Ices annual science conference” OR “Natural environment research council royal society of edinburgh symposium on molecular genetics in animal ecology” OR “Conference on invasive plants in natural and managed systems” OR “International brassica conference phenomics to genomics and everything in between” OR “14th genetic analysis workshop” OR “Discussion meeting on species and speciation in micro organisms” OR “International congress on biodiversity conservation and management” OR “3rd international meeting on ecological genetics in mammals” OR “Fisheries society of the british isles annual symposium on molecular biology in fish fisheries and aquaculture” OR “International society of molecular evolution symposium on junk dna the role and the evolution of non coding sequences” OR “7th international symposium on genetics in aquaculture” OR “Genetic analysis workshop 12 gaw12” OR “International workshop on clone 2000” OR “5th international symposium on genetics in aquaculture” OR “International conference on integrated appraches to sustain and improve plant production under drought stress” OR “International workshop on marine genetics” OR “18th congress of the scientific association of animal production aspa” OR “International symposium in fisheries ecology” OR “Meeting of the royal society of london” OR “2nd international meeting on ecological genetics in mammals” OR “International workshop on integrating across scales predicting patterns of change in atlantic salmon” OR “National academy of sciences colloquium on the future of evolution” OR “8th international symposium on genetics in aquaculture” OR “Napa conference on genetic and molecular ecotoxicology” OR “Royal society discussion meeting on insecticide resistance from mechanisms to management” OR “Annual symposium on fish genes and genomes contributions to ecology evolution and management” OR “Symposium on building on bevertons legacy life history variation and fisheries management” OR “Symp on the evolution of sex” OR “Colloquium on genetics and the origin of species” OR “Symposium on transgenic pest resistant crops held at the annual asa cssa ssa meeting” OR “Symposium on gene conservation held in conjunction with the 89th annual meeting of the national shellfisheries association” OR “Ices nasco symposium on interactions between aquaculture and wild stocks of atlantic salmon and other diadromous fish species” OR “13th international plant virus epidemiology symposium” OR “Symposium on intraclonal genetic variation ecological and evolutionary aspects” OR “International conference on global landscapes in cereal rust control” OR “16th conference of the association for the advancement of animal breeding and genetics aaabg” OR “Symposium on species delimitation new approaches for discovering diversity” OR “Meeting on ecological genetics in mammals current research and future perspectives” OR “1994 international conference on restoration of lake trout in the laurentian great lakes restore” OR “Symposium on the genus mus as a model for evolutionary studies held at the 4th european congress of mammalogy” OR “10th biennal congress of the european society for evolutionary biology eseb” OR “1st international conference on natural computation icnc 2005” OR “Workshop on evolution and role of transposable elements” OR “10th world buffalo congress 7th asian buffalo congress” OR “2nd asia pacific conference on simulated evolution and learning seal 98” OR “Xiv eucarpia congress on adaptation in plant breeding” OR “11th lowell wakefield fisheries international symposium on genetics of subarctic fish and shellfish” OR “3rd conference on microbiological surveillance and emerging infections” OR “10th international conference of the associacao brasileira de bioinformatica e biologia computacional” OR “20th annual meeting of the society of environmental toxicology and chemistry” OR “3rd

international conference of quantitative genetics” OR “10th international rotifer symposium” OR “5th annual genetic and evolutionary computation conference gecco 2003” OR “3rd international conference on dna fingerprinting” OR “10th symposium on insect plant relationships sip 10” OR “6th annual genetic and evolutionary computation conference gecco 2004” OR “3rd society for molecular biology and evolution young investigators workshop smbeyi 3” OR “10th tenth international symposium on genetics in aquaculture” OR “Aga presidential symposium on local adaptation from phenotype to genotype to fitness” OR “4th international otolith symposium” OR “12th eucarpia biometrics in plant breeding conference” OR “Symposium on advances in plant conservation biology implications for management and restoration” OR “4th international symposium on strugeon” OR “12th international symposium on digestive physiology of pigs” OR “Workshop on drosophila melanogaster” OR “5th international dna fingerprinting conference” OR “12th international symposium on the biology and management of coregonid fishes isbmcf” OR “Workshop on genetic protein variation in the atlantic salmon” OR “7th international symposium on sturgeons sturgeons science and society at the cross roads meeting the challenges of the 21st century” OR “16th biennial conference of the australasian pig science association apsa” OR “Workshop on molecular tools for monitoring marine invasive species” OR “17th annual meeting of the international genetic epidemiology society” OR “International conference workshop on new horizons in evolutionary biology” OR “14th deep sea biology symposium dsbs” OR “17th congress of the scientific association of animal production” OR “International cow fertility conference on new science new practices” OR “14th international hypoxia symposium” OR “1st eucarpia meeting of the section organic plant breeding and low input agriculture” OR “International polyploidy conference” OR “15th internation congress on animal reporduction” OR “22nd congress of the international primatological society” OR “International symposium on dispersal polymorphism of insects its adaptation and evolution” OR “15th international bat research conference” OR “23rd international conference of the world association for the advancement of veterinary parasitology” OR “International symposium on environmental factors cellular stress and evolution” OR “15th international congress on sexual plant reproduction” OR “23rd world poultry congress” OR “International symposium on linking herring” OR “17th international conference on industrial and engineering applications of artificial intelligence and expert systems” OR “2nd international downy mildews symposium” OR “Meeting on putting plant physiology on the map” OR “17th international meeting for specialists in air pollution effects on forest ecosystems stress factors and air pollution” OR “3rd biennial meeting of the international biogeography society” OR “Royal society discussion meeting on infection polymorphism and evolution” OR “17th world congress of the international union of prehistoric and protohistoric sciences uispp” OR “3rd international symposium on fish otolith research and application” OR “Satellite symposium on implementation challenges of smart semen and embryo technologies in cattle” OR “18th australian joint conference on artificial intelligence” OR “3rd international symposium on sturgeons iss” OR “Southern regional information exchange group meeting on genetic diversity in commercial forest tree plantations” OR “18th general congress of the european association for research on plant breeding” OR “3rd symposium on new concepts in stream ecology an integrated approach” OR “Symposium of the cephalopod international advisory council” OR “18th international congress of genetics” OR “3rd thematic meeting of craynet” OR “Symposium on eco evolutionary dynamics in cold blood at the joint

meeting of ichthyologists and herpetologists” OR “1992 annual conf of deutschen gesellschaft fur zuchtungskunde” OR “3rd workshop on clonal growth in plants” OR “Symposium on evolutionary physiology at the annual meeting of the society for integrative and comparative biology” OR “19th biennial meeting of the philosophy of science association” OR “4th international symposium on cladocera” OR “Symposium on gadoid mariculture” OR “19th conference of the world association for the advancement of veterinary parasitology” OR “4th international workshop on environmental applications of machine learning eaml” OR “Symposium on genetics of the fauna of the california current calcofi conference” OR “1st aquaculture symp the rainbow trout” OR “5th benelux congress of zoology” OR “Symposium on plant phenotyping” OR “1st international meeting of the international society of molecular evolution” OR “5th central european congress of life sciences eurobiotech” OR “Symposium on selection experiments as a tool in evolutionary and comparative physiology” OR “1st international meeting on sustaining the future of acacia plantation forestry” OR “5th international wheat conference” OR “Symposium on the variety types and significance of planted forests technical and social considerations for planted forests” OR “1st international symposium on chloroplast genomics engineering” OR “64th annual meeting of the american association of physical anthropologists” OR “Workshop on genetic simulation tools for post genome wide association studies of complex diseases” OR “1st international workshop on summer dormancy in grasses coping with increasing aridity and heat under climate change” OR “6th international symposium on cladocera” OR “Workshop on simian virus 40 sv40 a possible human polyomavirus” OR “1st meeting of french organic geochemists” OR “7th international symposium on abalone biology fisheries and culture” OR “10th conference on bayesian nonparametrics” OR “1st synthetic wheat symposium” OR “9th international conference on parallel problem solving from nature ppsn ix” OR “10th international conference on goats technological development and associate attempts to a sustainable small livestock activity” OR “20th anniversary meeting of the iga annual conference of the eaap” OR “9th international veterinary immunology symposium ivis summary of the comparative mhc workshops veterinary immunology committee toolkit workshop” OR “10th international symposium on genetics in aquaculture” OR “20th biennial meeting of the philosophy of science association” OR “Anton dohrn workshop on neutralism and selectionism the end of a debate” OR “10th international symposium on tardigrada” OR “21st conference on maize and sorghum breeding in the genomics era” OR “Bard workshop on aquaculture genetics status and prospects” OR “114th aoac international annual meeting” OR “21st international conference on dna computing and molecular programming dna” OR “Breeding and genetics symposium” OR “11th international conference and workshop on lobster biology and management icwl” OR “21st international congress of entomology” OR “British ornithologists union annual conference” OR “11th international conference on goats” OR “21st international ornithological congress” OR “Colloquium on tempo and mode in evolution” OR “11th international symposium on pollutant responses in marine organisms primo 11” OR “21st meeting of the international popular commission” OR “Conf on breeding for disease resistance” OR “11th international workshop on virus evolution and molecular epidemiology” OR “22nd conference of the international working group on ostrinia and other maize pests” OR “Conference on evolutionary genomics of sex evsex” OR “12th international goat conference icg” OR “22nd german conference on weed biology and weed control” OR “Conference on optimality in bird migration” OR “13th australasian plant breeding conference” OR “22nd meeting of the

environmental mutagen soc” OR “Conference on wood breeding biotechnology and industrial expectations” OR “13th international congress of parasitology” OR “22nd symposium of the society of population ecology” OR “Havemeyer foundation symposium to celebrate the life and work of rodger v short” OR “14th annual meeting of the gesellschafr fur okologie” OR “23rd congress of the international primatological society” OR “Hla and anthropology workshop conjoint meeting of the human biology association american association of physical anthropologists” OR “24th german conference on weed biology and weed control” OR “4th international charr symposium” OR “7th clonal plant workshop” OR “24th international conference on genome informatics giw” OR “4th international conference on advances in canine and feline genomics and inherited diseases” OR “7th conference on small genomes” OR “24th international ornithological congress” OR “4th international conference on bluetongue and related orbiviruses” OR “7th european conference on artifical life” OR “26th international ornithological congress ioc” OR “4th international ratite science symposium” OR “7th european conference on genetic programming” OR “28th congress of the international society of sugar cane technologists” OR “4th international symp on genetics in aquaculture” OR “7th international plant virus epidemiology symposium” OR “29th international symposium and national science meeting of the royal entomological society” OR “4th international symposium on ecological genetics in mammals” OR “7th international symposium on avian influenza” OR “2nd annual translational bioinformatics conference tbc” OR “4th international triticeae symposium” OR “7th international y chromosome user workshop 4th international empop meeting” OR “2nd global herbicide resistance challenge ghrc conference” OR “4th international zinc zn symposium” OR “8th alps adria scientific workshop” OR “2nd international conference on advances in information systems” OR “4th symp on chilean marine algae” OR “8th european conference on genetic programming” OR “2nd international conference on evolutionary multi criterion optimization emo 2001” OR “4th symposium for european freshwater science sefs 4” OR “8th international conference on parallel problem solving from nature ppsn viii” OR “2nd international conference on the advances in canine and feline genomics” OR “50th annual meeting of the society of nematologists symposium on legacy of entomopathogenic nematodes contributions to science and environmental sustainability” OR “8th international congress of plant molecular biology” OR “2nd international crocodilian dna workshop” OR “50th annual systematics symposium of the missouri botanical garden” OR “8th international congress of toxicology” OR “2nd international symposium on krill” OR “5th advances against aspergillosis conference” OR “8th international congress on molecular epidemiology and evolutionary genetics of infectious diseases” OR “2nd isirv international symposium on neglected influenza viruses” OR “5th greenhouse gases and animal agriculture ggaa conference” OR “8th international coral reef symposium” OR “2nd plant genome size workshop and discussion meeting” OR “5th ices international science otolith symposium ios” OR “8th international meeting of the spanish association for animal reproduction” OR “2nd symp on life history traits in tropical invertebrates at the 5th international congress of ecology” OR “5th international conference on boar semen preservation” OR “8th international symposium on cladocera” OR “30th annual conference of the international embryo transfer society” OR “5th international conference on parallel problem solving from nature ppsn v” OR “8th international symposium on the biology of management of coregonid fishes” OR “30th annual meeting of the brazilian embryo technology society sbte” OR “5th international conference on serpentine ecology” OR “8th

international symposium on the nutrition of herbivores isnh” OR “30th annual meeting of the plant population biology working group of the ecological society of germany austria switzerland” OR “5th international conference on stickleback behavior and evolution” OR “94th annual meeting of the german zoological society” OR “38th european congress of toxicology” OR “5th international symposium on abalone biology fisheries and culture” OR “96th annual meeting of the german zoological society” OR “39th european marine biology symposium” OR “5th international symposium on deep sea corals isdsc5” OR “9th european conference on genetic programming eurogp 2006” OR “3rd conference of the european ornithologists union” OR “5th international symposium on fig biology” OR “9th international abalone symposium” OR “3rd european conference on artificial life” OR “5th international symposium on reproduction in domestic ruminants” OR “9th international kimberlite conference” OR “3rd international conference on advances in pattern recognition” OR “5th international symposium on sturgeons” OR “9th international symposium of integrative zoology” OR “3rd international conference on evolutionary multi criterion optimization emo 2005” OR “5th symposium on speciation in ancient lakes sial” OR “9th international symposium on ecology of aphidophaga” OR “3rd international conference on loaches of the genus cobitis and related genera” OR “6th conference on the biology of plethodontid salamanders” OR “9th international symposium on insect plant relationships” OR “3rd international symposium of carabidology” OR “6th international conference on dormice” OR “9th international symposium on iron nutrition and interactions in plants” OR “3rd international symposium on cladocera as model organisms in biology” OR “6th international conference on mammoths and their relatives icmr” OR “9th pan african ornithological congress” OR “3rd international wildlife management congress” OR “6th international conference on simulated evolution and learning” OR “9th symposium of the international society for root research” OR “49th annual meeting of the american society of human genetics” OR “6th international rotifer symp” OR “Aaronsohn lectures on wild emmer wheat symposium” OR “4th annual scientific meeting of ocean production enhancement network open” OR “6th international symposium on sturgeons” OR “Aga presidential symposium on chromosome evolution molecular mechanisms and evolutionary consequences” OR “4th european conference on genetic programming eurogp 2001” OR “6th national congress of limnology” OR “Annual general meeting of the canadian phytopathological society symposium on contributions of genomics to plant pathology” OR “4th fish and shellfish larviculture symposium” OR “7th asian fisheries forum on restocking and stock enhancement of coastal fisheries” OR “Annual meeting of the american botanical society” OR “4th international billfish symposium” OR “Annual meeting of the american malacological society” OR “International conference on advances in genomics biodiversity and rapid systems for detection of toxigenic fungi and mycotoxins” OR “Meeting on marine biodiversity causes and consequences” OR “Annual meeting of the deutsche gesellschaft fur zuchtungskunde” OR “International conference on assessing the ecological integrity of running waters” OR “Meeting on nuclear techniques in food and agriculture” OR “Annual meeting of the deutsche gesellschaft fur zuchtungskunde ev” OR “International conference on biomarkers for toxicology and molecular epidemiology” OR “Mini workshop of the southeast asia germany alumni network” OR “Annual meeting of the poultry science association” OR “International conference on computational and mathematical population dynamics” OR “Nagoya international quaternary science meeting” OR “Arthur m sackler colloquium of the national academy of sciences on regenerative medicine” OR

“International conference on phenology climate change impacts and adaptation” OR “Poultry science association ancillary scientists symposium” OR “Ascochyta 2006 workshop” OR “International conference on systems biology isb” OR “Royal society discussion meeting on brainstem neural networks vital for life” OR “Beef species symposium joint annual meeting” OR “International gmelina workshop” OR “Royal society discussion meeting on the evloutionary legacy of the ice ages” OR “Bertebos prize symposium on impact of reproductive technology on animal breeding and genetic conservation” OR “International meeting on cholinesterases” OR “Royal society discussion meeting on the evolutionary legacy of the ice ages” OR “Biology of lactation in farm animals symposium on biology of lactation from genes to cells to milk held at the joint annual meeting of the american society of animal science asas and canadian society of animal science csas” OR “International meeting on genomes and evolution 2004” OR “Sap symposium on fish stock assessments and predictions” OR “Cephalopod international advisory council symposium and workshops ciac 2000” OR “International meeting on malaria progress problems and plans in the genomic era” OR “Scar workshop on evolutionary biology of antarctic organisms” OR “Colloquium on biotic and abiotic interactions regulating life cycle of marine invertebrates” OR “International symposium on analysing pathogen and pest populations in poplar and willow” OR “Studies of the hudson river estuary session of the 12th biennial international estuarine research federation conference” OR “Colloquium on links between recombination and replication vital roles of recombination” OR “International symposium on boron in soils and plants boron97” OR “Symp on the ecological and genetic implications of fish introductions” OR “Colloquium on systematics and the origin of species” OR “International symposium on dynamical systems theory and its applications to biology and environmental sciences” OR “Symposium in honor of the centenary of the birth of lionel penrose on unconventional genetic mechanisms and an unconventional geneticist” OR “Colloquium on variation and evalution in plants and micro organisms toward a new synthesis 50 years after stebbins” OR “International symposium on frontiers in molecular endocrinology” OR “Symposium of the society for experimental biology held in honour of georges bernier” OR “Colloquium on variation and evolution in plants and micro organisms toward a new synthesis 50 years after stebbins” OR “International symposium on live food organisms and marine larviculture” OR “Symposium on avian dispersal and demography scaling up to the landscape and beyond held at the 121st annual meeting of the aou” OR “Conference on adaptation of plants to water limited mediterranean environments” OR “International symposium on new challenges for animal science in a new century” OR “Symposium on coral reefs and environmental changes adaptation acclimation” OR “Extinction at the annual meeting of the society for comparative and integrative biology” OR “Conference on aquatic resources in arid lands” OR “International symposium on sandy beaches 94” OR “Symposium on crop modeling and genomics” OR “Conference on decision making and science the balancing of risk based decisions that influence sustainability of agricultural production” OR “International symposium on taeniasis cysticercosis and echinococcosis with focus on asia and the pacific” OR “Symposium on dna based profiling of mating systems and reproductive behaviors in poikilothermic vertebrates” OR “Conference on genetic improvement of broadleaved trees” OR “International symposium on the physiology of abscisic acid” OR “Symposium on emergence and control of zoonotic viral encephalitis” OR “Conference on molecular anthropology toward a new evolutionary paradigm” OR “International workshop on canine genetics the map the genes the diseases” OR “Symposium on

environmental impact of transgenic crops on soil biological processes and functions” OR “Conference on optimal migration” OR “International workshop on current approaches in basic and applied phycology” OR “Symposium on evolution of arthropod body plans integrating phylogeny fossils and development at the annual meeting of the society for integrative and comparative biology” OR “Conference on potato tissue culture” OR “International workshop on increasing wheat yield potential” OR “Symposium on food chain held in conjunction with 12th world congress of food science and technology” OR “Conference on risk assessment issues for sensitive human populations” OR “International workshop on modelling quality traits and their genetic variability for wheat” OR “Symposium on genomic impact of eukaryotic transposable elements” OR “Discussion meeting on conditions for the emergence of life on the early earth” OR “Iufro international conference on eucalypts and diversity balancing productivity and sustainability” OR “Symposium on hiv variants and hepatitis b virus surface antigen hbsag mutants” OR “Eucarpia fodder crops section meeting” OR “Joint conference of the 12th conference of the european foundation for plant pathology efpp 10th conference of the french society for phytopathology sfp” OR “Symposium on hybridization and systematics in herpetology at the 2nd world congress of herpetology” OR “Eucarpia genetic manipulation in plant breeding section meeting” OR “Joint meeting of the american society of animal science american dairy science association” OR “Symposium on individual intra population and inter population variability in human dna at the human biology association annual meeting” OR “Eucarpia meeting on tropical crop breeding” OR “Joint meeting of the british ecological society society for experimental biology effects of rising temperature on the ecology and physiology of aquatic organisms” OR “Symposium on integrated crop protection towards sustainability” OR “Evoworkshops 2000” OR “Keystone conference on genetic manipulation of insects” OR “Symposium on integrative approaches to the study of human adaptation and population health” OR “Fall meeting of the ohio plant biotechnology consortium opbc” OR “Lennoxville conference on milk production science serving the industry” OR “Symposium on integrative life history of whole organism performance at the annual meeting of the society for integrative and comparative biology sicb” OR “Forages and pastures symposium on use of marginal lands and fibrous byproducts in efficient beef and dairy production systems held at the joint annual meeting of the amer dairy sci assoc the amer soc of animal sci and the canadian soc of animal sci” OR “Meeting on ageing science medicine and society” OR “Symposium on one health zoonoses by virchow to date” OR “Genetics society spring meeting on sex” OR “Meeting on european crayfish as heritage species” OR “Symposium on post green revolution trends in crop yield potential increasing stagnant” OR “Greater resistance to stress held at the annual asa cssa sssa meeting” OR “Iets post conference symposium on implementation of artificial insemination in candes satellite symposium of the 35th international embryo transfer society” OR “Meeting on genetic variation and human health” OR “Symposium on safety an dadequacy testing of foods feeds nutritionally enhanced through biotechnology” OR “International belyaev conference on genetics and evolution genetics” OR “Meeting on iufro ecology and silviculture group” OR “Symposium on speciation in molluscs 75th annual meeting of the american malacological society” OR “International conference aqua 2000” OR “Symposium on sponges new views of old animals held at the annual meeting of the society for integrative and comparative biology” OR “10th jubilee east west immunogenetics conference ewic” OR “13th australian nitrogen fixation conference” OR “Symposium on the biology ecology and

physiology of zebra mussels at the american society of zoologists annual meeting" OR "10th meeting of the european study group on the moloecular biology of picornaviruses europic 98" OR "13th biennial conference of the australian society of agronomy" OR "Symposium on the evolutionaly ecology of genetic quality" OR "10th pan african ornithological congress" OR "13th european workshop on astrobiology eana" OR "Symposium on the extinction of the european neandertals during isotope stage 3 held at the 16th inqua congress" OR "10th teriological conference" OR "13th genetic analysis workshop" OR "Symposium on the first decade of namibian fisheries science" OR "10th winternational symposium of the canadian society of biochemistry and molecular and cellular biology" OR "13th international colloquium on soil zoology icsz" OR "Symposium on tomorrows poultry genomics physiology and well being" OR "110th annual meeting of the american society for horticultural science" OR "13th international conference on accelerator and beam utilization" OR "Viiith international rotifer symposium" OR "11th annual conference of the european society for domestic animal reproduction" OR "13th international congress on animal reproduction" OR "Workshop on chemical communication in aquatic systems" OR "11th european congress of ichthyology" OR "13th international symposium on aquatic oligochaeta isao" OR "Workshop on connectivity and resilience sustaining coral reefs during the coming century" OR "11th genetic analysis workshop on analysis of genetic and environmental factors in common diseases" OR "13th international symposium on methodologies for intelligent systems ismis 2002" OR "Workshop on equine cyathostomins" OR "11th international conference on frankia and actinorhizal plants" OR "13th international work conference on artificial neural networks iwann" OR "Workshop on molecular evolution" OR "11th international conference on luminescence and electron spin resonance dating" OR "13th symposium on virtual and augmented reality svr" OR "Workshop on reproduction and genetics of freshwater fish in aquaculture" OR "11th international conference on trichinellosis" OR "13th triennial state of the heartworm symposium 2010" OR "Workshop on reproductive biotechnology in finfish aquaculture" OR "11th international congress of endocrinology" OR "13th world congress on pain" OR "Workshop on the metapopulation concept in coastal waters" OR "11th international rotifer symposium" OR "14th eurographics workshop on graphics and cultural heritage gch" OR "World conference on the scientific and technical bases for the sustainability of fisheries" OR "11th international symposium on aquatic weeds" OR "14th international aids conference" OR "World congress of the international association for landscape ecology iale" OR "11th international symposium on genetics in aquaculture" OR "14th international bioinformatics workshop on virus evolution and molecular epidemiology" OR "Xiiith max born symposium on statistical physics in biology perspectives in dna analysis population dynamics and ageing" OR "11th international symposium on iron nutrition and interactions in plants" OR "14th international colloquium on soil zoology soil animals and ecosystems services" OR "Xivth international plant protection congress ippc" OR "11th international symposium on pre harvest sprouting in cereals" OR "14th international conference on the synthesis and simulation of living systems alife" OR "101st annual meeting of the american society for clinical pharmacology and therapeutics" OR "11th scientific committee on antarctic research symposium" OR "14th international congress on animal reproduction research and practice ii" OR "10th annual international conference on research in computational molecular biology" OR "12th animal sciences congress asian australasian association of animal production

societies” OR “14th international seaweed symp” OR “10th annual meeting of the national birth defects prevention network” OR “12th colloque of the club des bacteries lactiques” OR “14th international symposium on pollutant responses in marine organisms primo 14” OR “10th conference of the spanish association for artificial intelligence caepia 2003 5th conference on technology transfer ttia 2003” OR “12th international conference of the pacific basin consortium for environment and health sciences” OR “14th international symposium on soil and plant analysis isspa” OR “10th institute for computational fluid dynamics icfd conference” OR “12th international conference on ephemeroptera 16th international symposium on plecoptera” OR “14th international workshop on instabilities and non equilibrium structures” OR “10th international cmv betaherpesvirus workshop” OR “12th international congress on invertebrate reproduction and development icird” OR “14th north american colloquium on animal cytogenetics and gene mapping” OR “10th international conference on frankia and actinorhizal plants” OR “12th international meeting on cholinesterases 6th international conference on paraoxonase” OR “14th turkish symposium on artificial intelligence and neural networks” OR “10th international conference on toxic cyanobacteria ictc” OR “12th international rotifer symposium rotifera” OR “14th workshop of the international association of phytoplankton taxonomy and ecology” OR “10th international congress on infectious diseases” OR “12th international symposium on environmental pollution and its impact on life in the mediterranean region” OR “14th world congress of the union internationale de phlebologie” OR “10th international plant virus epidemiology symposium” OR “12th international symposium on fish nutrition and feeding” OR “15th australian joint conference on artificial intelligence” OR “10th international symposium on cladocera” OR “12th international symposium on health related water microbiology” OR “15th conference of the cochin saint vincent de paul clinical pharmacology on child and adult vaccines” OR “10th international symposium on human identification” OR “12th international symposium on insect plant relationships sip 12” OR “15th conference on applied stochastic models and data analysis asmda” OR “10th international symposium on pre harvest sprouting in cereals” OR “12th international symposium on pollutant responses in marine organisms primo 12” OR “15th european congress of ichthyology” OR “10th international symposium on schistosomiasis” OR “12th meeting of the scientific group on methodologies for the safety evaluation of chemicals sgomsec 12” OR “15th international conference on accelerators and beam utilizations” OR “10th international symposium on soil and plant analysis” OR “13th annual australian weeds conference” OR “15th international conference on aquatic invasive species icais” OR “10th international symposium on structural and syntactic pattern recognition 5th international conference on statistical techniques in pattern recognition” OR “15th international conference on industrial and engineering applications of artificial intelligence and expert systems iea aie 2002” OR “18th international symposium on bioelectrochemistry and bioenergetics 3rd spring meeting of the international society of electrochemistry” OR “1st international conference on oxygen and environmental stress in plants” OR “15th international congress of comparative endocrinology” OR “18th iufro workshop on air pollution stress forest responses to the pollution climate of the 21st century” OR “1st international conference on phylogenomics” OR “15th international congress on animal reproduction” OR “18th world congress of soil science” OR “1st international fisheries symposium” OR “15th italian hungarian symposium on spectrochemistry ihss” OR “1990 annual meeting of the american soc of zoologists”

OR “1st international lake ladoga symposium on ecological problems of lake ladoga” OR “15th iufost world congress of food science and technology on food science solutions in evolving world” OR “1992 beilstein workshop on similarity in organic chemistry” OR “1st international meeting on cognitive systems with integrated sensor” OR “15th meeting of the plant population biology section of the ecological society of germany” OR “1993 conference of the australian society for parasitology” OR “1st international meeting on microbial phosphate solubilization” OR “15th triennial symposium of the american heartworm society” OR “1995 ssb symposium” OR “1st international palaeobiogeography symposium” OR “15th workshop of the international association of phytoplankton taxonomy and ecology” OR “1997 american association for artificial intelligence spring symposium on the adaptation co evolution and learning in multiagent systems” OR “1st international rice research congress” OR “15th world congress on bryology” OR “1997 annual meeting of the societe zoologique de france” OR “1st international symp on flatfish ecology” OR “16th annual meeting of the american society for reproductive immunology” OR “1998 symposium on the ecology and management of fragmented tropical landscapes” OR “1st international symp on the cereal stem borer chilo tropical stem borers of graminaceous crops a new synthesis” OR “16th annual meeting of the society of behavioral medicine” OR “19th acvim meeting” OR “1st international symposium on donkey science” OR “16th baltic marine biologists symposium” OR “19th congress of the international academy of legal medicine” OR “1st international symposium on fire blight of rosaceous plants isfb” OR “16th conference of the international work group for palaeoethnobotany iwgp” OR “19th genetic analysis workshop gaw” OR “1st international symposium on riverine landscapes” OR “16th discussion meeting on darwin and the evolution of flowers” OR “19th global biennial conference of the international society for ecological modelling isem” OR “1st international symposium on the biology of non weedy hemiparasitic ex scrophulariaceae” OR “16th international congress on animal reproduction” OR “19th international botanical congress ibc” OR “1st international symposium on wolverine research and management” OR “16th international symposium on insect plant relationships sip” OR “19th international conference on industrial engineering and other applications of applied intelligent systems” OR “1st international symposium oncomputational and information science” OR “16th international symposium on methodologies for intelligent systems” OR “19th international conference on pharmacoepidemiology annual meeting of the international society for pharmacoepidemiology” OR “1st international workshop on internet and network economics” OR “17th annual australian conference on artificial intelligence” OR “19th international congress of the transplantation society” OR “1st international workshop on the biology of fish sperm” OR “17th annual meeting of the associaton for the advancement of industrial crops” OR “19th north american testis workshop” OR “1st meeting of the international soc for biochemical systematics” OR “17th biennial meeting of the philosophy of science association” OR “1st benelux congress on zoology” OR “1st national meet on arid zones” OR “17th international botanical congress” OR “1st biosafety workshop of cost action fp0905” OR “1st national research conference on childrens environmental health research practice prevention and policy” OR “17th international symposium on animal science days” OR “1st central european meeting on mouse epigenetics” OR “1st q bio conference on cellular information processing” OR “17th mendeleev congress on general and applied chemistry” OR “1st cmu unsw science challenges symposium” OR “1st seminar on remote sensing applied to

fishing” OR “17th symposium of population ecology” OR “1st colombian autoimmune symposium” OR “1st toponorge workshop on geological survey of norway” OR “17th world congress of the international society for heart research” OR “1st conference of the european society for domestic animal reproduction” OR “1st workshop of the mediterranean academy of forensic sciences” OR “17th world congress of the uispp” OR “1st european influenza conference” OR “1st workshop on equitation science” OR “18th annual meeting of the american society for bone and mineral research” OR “1st global change and terrestrial ecosystems science conference” OR “1st world congress of agroforestry” OR “18th biennial meeting of the philosophy of science association” OR “1st international combinatorial catalysis symposium iccs” OR “2000 great unkowns symposium” OR “18th conference on process integration modelling and optimisation for energy saving and pollution reduction pres” OR “1st international conference on bioinformatics and computational biology” OR “20th conference of the world association for the advancement of veterinary parasitology” OR “18th congress of the italian society of sheep and goat pathology and production” OR “1st international conference on chemical biological radiological and nuclear research and innovation cbrn ri” OR “20th eucarpia general congress” OR “18th european colloquium of arachnology” OR “1st international conference on european shads” OR “21st annual meeting of the society of environmental toxicology and chemistry” OR “18th international biohydrometallurgy symposium” OR “1st international conference on evolutioary multi criterion optimization emo 2001” OR “21st congress of the international primatological society” OR “18th international conference of the world association for the advancement veterinary parasitology” OR “1st international conference on evolutionary multi criterion optimization emo 2001” OR “21st international biohydrometallurgy symposium ibs” OR “18th international congress of biochemistry and molecular biology” OR “21st international symposium for polycyclic aromatic compounds” OR “2nd embo workshop on pathogenesis and amoebiasis” OR “2nd international workshop on the assessment of animal welfare at farm and group level” OR “21st symposium of the society of population ecology” OR “2nd european conference on pesticides and related organic micropollutants in the environment” OR “2nd japanese israeli symposium on aquaculture” OR “22nd annual conference of the american college of toxicology” OR “2nd european workshop of invertebrate ecophysiology” OR “2nd mediterranean symposium on medicinal and aromatic plants mesmap” OR “22nd international congress of entomology” OR “2nd forensic y chromosome user workshop” OR “2nd norwegian environmental toxicology symposium” OR “22nd iufro world forestry congress” OR “2nd hellenic conference on artificial intelligence” OR “2nd russian chinese conference on biodiversity of the animal kingdom and on the functions of its ecosystem” OR “22nd world congress of the international union of forestry research organization” OR “2nd ieee international conference on intelligent information hiding and multimedia signal processing” OR “2nd sendai histamine symposium” OR “22nd world poultry congress” OR “2nd inoculant forum on recent advances in plant inoculants research” OR “2nd symposium on urbanization and stream ecology” OR “23rd international congress of cardiology” OR “2nd international 6th national conference on plant cell biology in vitro and biotechnology” OR “2nd thematic meeting of craynet” OR “24th annual meeting of the behavior genetics association” OR “2nd international conf on tropical entomology bio control in the tropics” OR “2nd wilhelm stahl symposium on animal husbandry 2000” OR “24th colloquium of the sfea” OR “2nd international conference on bio logging science” OR “2nd william r and lenore mote international symposium in

fisheries ecology” OR “24th congress of the polish physiological society” OR “2nd international conference on control decision and information technologies codit” OR “2nd world conference on ornamental fish aquaculture” OR “24th institute of foresters of australia ifa national conference on australian forestry a climate of change” OR “2nd international conference on deterministic and stochastic modeling of biological interaction destebio 2000” OR “2nd world congress of herpetology” OR “25th conference of the international association of agricultural economists” OR “2nd international conference on environmental and engineering geophysics” OR “2nd world congress of international plant proteomics organization inppo” OR “25th international conference on adaptive structures and technologies icast” OR “2nd international conference on environmental mutagens in human populations” OR “2nd world congress on allelopathy” OR “25th pattern recognition symposium of the german association for pattern recognition” OR “2nd international conference on mediterranean pines” OR “2nd world congress on vaccines and immunisation” OR “26th annual meeting of the american society of primatologists” OR “2nd international conference on natural computation icnc 2006” OR “31st annual meeting of the brazilian embryo technology society sbte” OR “26th biennial conference of the australian society of animal production” OR “2nd international conference on novel approaches to the control of helminth parasites of livestock” OR “31st european marine biology symposium on interactions and adaptation strategies of marine organisms” OR “26th european congress of clinical microbiology and infectious diseases eccmid” OR “2nd international conference on seed science and technology” OR “31st international geological congress” OR “26th european marine biology symp on biological effects disturbances on estuarine and coastal marine environments” OR “2nd international conference on water energy and environment icwee” OR “31th conference of the association mare amico” OR “26th international congress of entomology ice” OR “2nd international jellyfish blooms symposium” OR “32nd annual conference of the international embryo transfer society” OR “27th biannual meeting of the german society for parasitology dgp” OR “2nd international large branchiopod symposium” OR “34th annual meeting of the society for neuroscience” OR “27th biennial conference of the australian society of animal production 68th annual conference of the new zealand society of animal production” OR “2nd international meeting on a molecular and cellular view of protein kinase ck2” OR “34th congress of the federation of european biochemical societies” OR “27th congress of the international society of sugar cane technologists” OR “2nd international meeting on mammalian embryogenomics” OR “36th annual conference of the international embryo transfer society” OR “27th sgai international conference on innovative techniques and applications of artificial intelligence” OR “2nd international percid fish symposium percid ii” OR “36th symposium of the european marine biological association” OR “28th annual meeting of the brazilian embryo technology society sbte” OR “2nd international symp workshop on frugivores and seed dispersal” OR “37th annual meeting of the environmental mutagen society” OR “28th international conference on genome informatics medical genomics” OR “2nd international symposium of aquatic plant biology macrophyte” OR “38th spring meeting of the german society of experimental and clinical pharmacology and toxicology” OR “29th annual conference of the international embryo transfer society” OR “2nd international symposium on candidate genes for animal health” OR “39th annual meeting of the society for economic botany” OR “29th congress of the international society of sugar cane technologists issct” OR “2nd international symposium on fish behaviour in exploited ecosystems” OR “39th congress of the

european societies of toxicology” OR “29th congress on occupational and environmental health in the chemical industry” OR “2nd international symposium on helicobacter pylori infection and gastric pathology” OR “3rd adelf thematic conference on social epidemiology and health inequalities” OR “29th meeting of the association of marine labortories of the caribbean amlc” OR “2nd international symposium on nutritional strategies and management of aquaculture waste” OR “3rd annual conference on sturgeons of the north american chapter of the world sturgeon conservation society” OR “2nd alcala international conference on mathematical ecology aicme2” OR “2nd international symposium on stickleback behaviour” OR “3rd annual meeting of the european veterinary parasitology college” OR “2nd aquaculture sponsored symposium” OR “2nd international symposium on sweeteners” OR “3rd colloquium on particulate air pollution and human health” OR “2nd congress of italian evolutionary biologists 1st congress of the italian society for evolutionary biology” OR “2nd international workshop on machine learning and data mining in pattern recognition” OR “3rd conference of the hungarian genetical society” OR “2nd ecology of stream fish meeting” OR “3rd conference on physiology and biochemistry in animal nutrition” OR “3rd samuel a latt conference on genomics and proteomics in cancer” OR “4th international conference on computational science iccs 2004” OR “3rd intenational meeting on telomerase and cancer stem cells” OR “3rd scientific symposium on promoting marine science for management in eastern africa” OR “4th international conference on fertility control for wildlife management” OR “3rd international billfish symposium” OR “3rd symposium of the european association of acarology” OR “4th international conference on mycorrizae icom 2003” OR “3rd international conference for comparative physiology and biochemistry” OR “3rd symposium on industrial crops and products” OR “4th international conference on novel approaches to the control of helminth parasites of livestock” OR “3rd international conference of the brazilian association for bioinformatics and computational biology” OR “3rd symposium on the environmental monitoring and assessment program” OR “4th international conference on optimization simulation and control” OR “3rd international conference on advances in canine and feline genomics” OR “3rd workshop on the upper parana river floodplain conservation and biodiversity” OR “4th international conference on oxidative nitrosative stress and disease” OR “3rd international conference on molecular mechanisms of metal toxicity and carcinogenesis” OR “3th international meeting on esterases reacting with organophosphorus compounds” OR “4th international conference on rickettsiae and rickettsial diseases” OR “3rd international conference on neural tube defects” OR “40th annual meeting of the mississippi river research consortium” OR “4th international conference on stickleback behaviour and evolution” OR “3rd international conference on occupational and environmental toxicology icoetox 3rd ibero american meeting on toxicology and environmental health international ibamtox” OR “40th interscience conference on antimicrobial agents and chemotherapy” OR “4th international meeting on apicomplexan parasites in farm animals apicowplexa” OR “3rd international conference on water resource and environment wre” OR “40th national congress and 16th international conference of the spanish society for sheep and goat production seoc” OR “4th international seagrass biology workshop” OR “3rd international hydrogen forum” OR “41st annual meeting of the society for invertebrate pathology” OR “4th international study workshop on tsetse population and behaviour” OR “3rd international membrane research forum” OR “41st national congress of genetics” OR “4th international swan symposium 18th trumpeter swan society conference” OR “3rd

international mycovirus symposium ims” OR “44th european muscle conference emc” OR “4th international symp on genetic aspects of plant mineral nutrition” OR “3rd international symp on genetics in aquaculture” OR “44th international congress of meat science and technology” OR “4th international symposium on adances in legal medicine isalmiv” OR “3rd international symposium in fisheries ecology” OR “44th interscience conference on antimicrobial agents and chemotherapy” OR “4th international symposium on biological monitoring” OR “3rd international symposium on bioinformatics research and applications” OR “44th phylogenetisches symposium” OR “4th international symposium on flatfish ecology” OR “3rd international symposium on emys orbicularis” OR “45th annual meeting of the eaap” OR “4th international symposium on littorinid biology” OR “3rd international symposium on flatfish ecology” OR “45th annual systematics symposium of the missouri botanical garden” OR “4th international symposium on plant growth modelling and application pma12” OR “3rd international symposium on fusarium head blight” OR “46th annual meeting of the american society of human genetics” OR “4th international symposium on sturgeon” OR “3rd international symposium on inorganic carbon acquisition by aquatic photosynthetic organisms” OR “47th annual meeting of the international association of forensic toxicologists” OR “4th international veterinary immunology symposium” OR “3rd international symposium on monogenea” OR “48th annual meeting of the american academy of forensic sciences” OR “4th international weed science congress” OR “3rd international symposium on population dynamics of plant inhabiting mites” OR “48th phylogenetic symposium on historical biogeography” OR “4th international workshop on biology and culture of the tench tinca” OR “3rd international symposium on positive strand rna viruses” OR “49th annual systematics symposium of the missouri botanical garden” OR “4th international workshop on lobster biology and management” OR “3rd international symposium on trace elements in food tef 3” OR “4th annual meeting of the european veterinary parasitology college” OR “4th international workshop on signal transduction in the activation and development of mast cells and basophils” OR “3rd international workshop on crocodylian genetics and genomics in ecological genetics and physiology” OR “4th congress of the european association for clinical pharmacology and therapeutics” OR “4th international workshop on structure and function of large arteries” OR “3rd international workshop on genome privacy and security genopri” OR “4th congress of the european society for agronomy on perspectives for agronomy adopting ecological principles and managing resource use” OR “4th international zooplankton production symposium” OR “3rd international workshop on reproductive immunology immunological tolerance and immunology of preeclampsia” OR “4th crustacean larval conference” OR “4th iranian joint congress on fuzzy and intelligent systems cfis” OR “3rd international workshop on sea lice” OR “4th european conference on ecotoxicology and environmental safety” OR “4th iwa activated sludge population dynamics conference aspd4” OR “3rd international zooplankton production symposium” OR “4th european hemiptera congress” OR “4th meeting of the european academy of forensic science” OR “3rd iupac international conference on biodiversity icob 3” OR “4th european large lakes symposium ells” OR “4th ostertagia workshop on nematode parasites of importance to ruminant livestock” OR “3rd latin american congress of echinoderms” OR “4th hutton symoposium on the origin of granites and related rocks” OR “4th rcmi international aids symposium” OR “3rd national symposium on large scale structural analysis for high performance computers and workstations” OR “4th interdrought conference” OR “4th summer school of monolith technology for biochromatography bioconversion and

phase state synthesis” OR “3rd new england biolabs workshop on biological dna modification” OR “4th international black grouse conference” OR “4th symposium on speciation in ancient lakes” OR “3rd norwegian environmental toxicology symposium” OR “4th uk e science all hands meeting ahm 2005” OR “5th international conference on biological physics” OR “69th annual meeting of the american association of physical anthropologists” OR “4th world congress on alternatives and animal use in the life sciences” OR “5th international conference on dormouse myoxidae” OR “6th annual q bio conference” OR “50th international congress of the international society for applied ethology isae” OR “5th international conference on evolution artificielle” OR “6th australasian dairy science symposium” OR “51st annual meeting of the european association for animal production” OR “5th international conference on independent component analysis and blind signal separation ica” OR “6th conference of the asian society for mitochondrial research and medicine” OR “52nd annual systematics symposium of the missouri botanical garden” OR “5th international conference on information and management sciences” OR “6th european conference on ecological modelling challenges for ecological modelling in a changing world” OR “54th annual meeting of the european association for animal production” OR “5th international conference on marine pollution and ecotoxicology” OR “6th european conference on evolutionary computation in combinatorial optimization evocop 2006” OR “54th annual systematics symposium of the missouri bolanical garden on biodiversity and conservation in the andes” OR “5th international conference on the prevention of infection” OR “6th european conference on genetic programming eurogp 2003” OR “55th annual meeting of the european association of animal production” OR “5th international conference on toxic cyanoacteria” OR “6th iawq symposium on forest industry wasterwaters” OR “55th annual meeting of the italian society of veterinary sciences” OR “5th international poultry show and seminar” OR “6th iberian 3rd iberoamerican congress on environmental contamination and toxicology” OR “55th conference of estuarine coastal sciences association ecsa” OR “5th international psychiatry forum on mind matters” OR “6th ices pices zooplankton production symposium zps” OR “58th annual meeting of the italian society for veterinary sciences sisvet” OR “5th international symposium on aquatic oligochaeta” OR “6th international billfish symposium ibs” OR “59th annual meeting of the european association for animal production” OR “5th international symposium on emys orbicularis and the other european freshwater turtles” OR “6th international colloquium on the ecology and taxonomy of small african mammals” OR “59th international congress of meat science and technology icomst” OR “5th international symposium on fertility control in wildlife” OR “6th international conference and workshop on lobster biology and management” OR “5th acm conference on bioinformatics computational biology and health informatics acm bcb” OR “5th international symposium on flatfish ecology” OR “6th international conference on civil and structural engineering computing 4th international conference on the application of artificial intelligence to civil and structural engineering” OR “5th anniversary symposium of methods in ecology and evolution” OR “5th international symposium on inorganic carbon utilization by aquatic photosynthetic organisms” OR “6th international conference on computational science iccs 2006” OR “5th annual conference on vaccine research” OR “5th international symposium on littorinid biology” OR “6th international conference on copepoda” OR “5th annual meeting of the north american sturgeon and paddlefish society nasps” OR “5th international symposium on radioautography” OR “6th international conference on serpentine ecology” OR “5th anton dohrn workshop”

OR “5th international symposium on shallow lakes” OR “6th international conference on the simulation and synthesis of living systems” OR “5th ase international conference on big data 4th ase international conference on social informatics” OR “5th international veterinary immunology symposium” OR “6th international congress of toxicology toxicology from discovery and experimentation to the human perspective” OR “5th australasian soilborne diseases symposium” OR “5th international work conference on the interplay between natural and artificial computation iwinac” OR “6th international congress on twin studies” OR “5th australian tropical pastures conference” OR “5th international workshop on biology and culture of the tench” OR “6th international crustacean congress icc6” OR “5th biennial conference of the society for tropical veterinary medicine” OR “5th international workshop on functional structural plant models” OR “6th international littorinid symposium” OR “5th cec malaria contract holders meeting” OR “5th international workshop on mhc evolution” OR “6th international phytotechnologies conference” OR “5th clonal plant workshop on clonal plants and environmental heterogeneity space time and scale” OR “5th international workshop on project management and scheduling” OR “6th international symposium on abalone biology fisheries and culture” OR “5th conference on molecular approaches to malaria mam” OR “5th iwa international conference on biofilm systems” OR “6th international symposium on animal biology of reproduction isabr” OR “5th congress of sociedad espanola para los recursos geneticos animales serga” OR “5th meeting of the asian society for mitochondrial research and medicine asmrm” OR “6th international symposium on biochemical roles of eukaryotic cell surface macromolecules” OR “5th european conference on ecological modelling” OR “5th michael evenari memorial meeting” OR “6th international symposium on ecology and environmental problems” OR “5th european mammal congress on mammal conservation europe status and priorities” OR “5th symposium on enzymes in grain processing” OR “6th international symposium on fertility control in wildlife” OR “5th fish and shelfish larviculture symposium larvi 2009” OR “5th symposium on frontiers in protein chemistry and biotechnology 9th china korea regional symposium on biochemistry” OR “6th international symposium on fish endocrinology” OR “5th international billfish symposium” OR “5th symposium on human genetics” OR “6th international symposium on flatfish ecology” OR “5th international conf of the israel soc for ecology and environmental quality environmental quality and ecosystem stability” OR “63rd international congress of meat science and technology icomst” OR “6th international symposium on reproduction in domestic ruminants” OR “5th international conf on coelenterate biology” OR “66th annual meeting of the cooper ornithological society” OR “6th international symposium on the lacertids of the mediterranean basin” OR “5th international conference of anticancer research” OR “678th meeting of the biochemical society” OR “6th international workshop on advanced parallel processing technologies” OR “5th international conference on advances in pattern recognition” OR “6th international workshop on in vitro toxicology invitox” OR “7th nih symposium on therapeutic oligonucleotides” OR “8th international symposium on reproductive physiology of fish” OR “6th international workshop on reproductive immunology immunological tolerance and immunology of preeclampsia” OR “7th william r and lenore mote international symposium in fisheries ecology” OR “8th international symposium on the biology of turbellaria” OR “6th meeting of the international sorex araneus cytogenetics committee isacc” OR “7th world congress on genetics applied to livestock production” OR “8th international workshop on foundations of genetic

algorithms” OR “6th research conference of the european turfgrass society ets on different shades of green” OR “80th annual meeting of the american society of ichthyologists and herpetologists” OR “8th international workshop on salmonid smoltification” OR “6th united states national congress on computational mechanics” OR “80th annual meeting of the american society of ichthyologists and herpetologists society for the study of amphibians and reptiles and herpetologists league” OR “8th latin american congress and workshop on anaerobic digestion” OR “6th william r and lenore mote international symposium in fisheries ecology” OR “81st annual meeting of the society for american archaeology” OR “8th pacific rim international conference on artificial intelligence pricai 2004” OR “6th william r lenore mote international symposium in fisheries ecology” OR “87th annual meeting of the asas” OR “90th annual meeting of the ecological society of america 9th international congress of ecology” OR “7th annual meeting of the european veterinary parasitology college evpc” OR “88th annual meeting of the american association for cancer research” OR “91st annual congress of the quebec society for the protection of plants” OR “7th conference of the international society for plant anaerobiosis” OR “88th annual meeting of the ecological society of american” OR “92nd annual meeting of the american phytopathological society” OR “7th fuzzy international conference on computational intelligence” OR “8th biennial conference of the society for tropical veterinary medicine” OR “94th annual meeting of the american phytopatholgical society” OR “7th international chrysophyte symposium” OR “8th colloquium on epidemiological control of infectious diseases” OR “96th annual conference of the american society for horticultural science ashs” OR “7th international conference and workshop on lobster biology and management” OR “8th conference on models in population dynamics and ecology mpde” OR “96th annual meeting of the quebec society for the protection of plants” OR “7th international conference on artificial intelligence and soft computing” OR “8th congress of the european society of evolutionary biology” OR “97th annual meeting of american phytopathological society” OR “7th international conference on coelenterate biology” OR “8th international behavioral ecology congress” OR “97th annual meeting of the poultry science association” OR “7th international conference on evolution artificielle” OR “8th international botanical microscopy meeting” OR “9th annual conference of the european society for domestric animal reproduction esdar” OR “7th international conference on fata and effects of pulp and paper mill effluents returned to canada 9th international water assocation symposium on forest industry wastewaters” OR “8th international conf on frankia and actinorhizal plants” OR “9th annual international conference on computing and combinatorics” OR “7th international conference on fata and effects of pulp and paper mill effluents returned to canada 9th international water association symposium on forest industry wastewaters” OR “8th international conference and workshop on lobster biology and management” OR “9th conference on metal toxicity and carcinogenesis” OR “7th international conference on knowledge based intelligent information and engineering systems kes 2003” OR “8th international conference on artificial intelligence and soft computing icaisc 2006” OR “9th euring analytical meeting” OR “7th international congress on autoimmunity” OR “8th international conference on copepoda” OR “9th ifip wg 12 5 international conference on artificial intelligence applications and innovations aiai” OR “7th international deer biology congress idbc” OR “8th international conference on knowledge based intelligent information and engineering systems” OR “9th international conference on copepoda” OR “7th international ichthyology congress

the threatened world of fish” OR “8th international conference on sea lice” OR “9th international conference on frankia and actinorhizal plants” OR “7th international oat conference” OR “8th international conference on stickleback bahaviour and evolution” OR “9th international conference on goats” OR “7th international plant virus epidemilolgy symposium” OR “8th international congress on the zoogeography and ecology of greece and adjacent regions” OR “9th international conference on juvenile hormones” OR “7th international rotifer symposium” OR “8th international feline retrovirus research symposium” OR “9th international conference on magnetic fluids” OR “7th international sheep veterinary congress” OR “8th international grouse symposium” OR “9th international conference on neural tube defects ntds” OR “7th international symposium on cladocera” OR “8th international neural tube defects conference” OR “9th international conference on serpentine ecology icse” OR “7th international symposium on earthworm ecology isee7” OR “8th international sheep veterinary congress” OR “9th international congress on isozymes genes and gene families” OR “7th international symposium on extant and fossil charophytes” OR “8th international symposium on earthworm ecology isee8” OR “9th international congress on molecular epidemiology and evolutionary genetics of infectious diseases” OR “7th international symposium on reproductive physiology of fish” OR “8th international symposium on flatfish ecology” OR “9th international flatfish symposium ifs” OR “7th international symposium on use of algae for monitoring rivers” OR “8th international symposium on grapevine physiology and biotechnology” OR “9th international symposium of the icp br bee protection group” OR “7th international veterinary immunology symposium ivis 7” OR “8th international symposium on oceanography of the bay of biscay” OR “9th international symposium on avian influenza” OR “7th international workshop on salmonid smoltification” OR “8th international symposium on pollutant responses in marine organisms primo 9” OR “9th international symposium on responses of marine organisms to pollutants primo 9” OR “7th international workshop on the fragile x and x linked mental retardation” OR “8th international symposium on recent advances in drug delivery systems” OR “9th international veterinary hemoparasite disease conference” OR “7th neotropical ornithological congress” OR “9th pacific asia conference on knowledge discovery and data mining” OR “Annual meeting of the society for molecular biology and evolution” OR “Colloquium on protecting our food supply the value of plant genome initiatives” OR “9th symposium on the chemistry and fate of modern pesticides” OR “Annual meeting of the weed science society of america” OR “Colloquium on rupert c barneby and his legume legacy” OR “Acm genetic and evolutionary computation conference gecco” OR “Annual scientific conference of the internationals commission for exploration of seas ices” OR “Colloquium on staying healthy with chips the future of diagnosis” OR “Advances against aspergillosis conference” OR “Annual scientific meeting of the australian and south east asian tissue typing association aseatta” OR “Colloquium on update on statistical methods for interpreting horticulural data held at the ashs annual conference” OR “Aiaa asme asce ahs asc 45th structures structural dynamics and materials conference” OR “Annual scientific meeting of the human biology association” OR “Conf on marker aided selection a tool for the improvement of forest tree species” OR “Aiaa asme asce ahs asc 46th structures structural dynamics and materials conference 1st aiaa multidisciplinary design optimization specialist conference” OR “Annual scientific meeting on structural approaches to sequence evolution” OR “Conf on ungulate behavior and management” OR “Aiaa nasa usaf issmo 8th symposium on

multidisciplinary analysis and optimization” OR “Annual symposium of the biochemical society” OR “Conference of the society for veterinary epidemiology and preventive medicine” OR “Air pollution workshop 2006” OR “Annual symposium of the fisheries society of the british isles on tropical fish biology” OR “Conference on antimicrobial peptides mediators of innate immunity in the development of anti infective therapeutic and vaccination strategies” OR “American genetic association symposium on concepts in genetics” OR “Annual symposium on biology of polar fish of the fisheries society of the british isles” OR “Conference on application of technology to chemical mixture research” OR “Ancillary scientists symposium on avian immune system” OR “Annual symposium on molecular evolutionary genetics” OR “Conference on aristophanes upstairs and downstairs peace birds and frogs in ancient and modern performance” OR “Annual conference of the australasian association for philosophy” OR “Asas adsa midwestern sectional meeting at the environment and livestock production animal growth symposium stressors that alter growth” OR “Conference on behaviour and ecology of freshwater fish linking ecology and individual behaviour” OR “Annual conference of the australian society for parasitology” OR “Asian fish biodiversity conference” OR “Conference on beryllium related diseases” OR “Annual conference of the international embryo transfer society” OR “Asm beneficial microbes conference” OR “Conference on biochemical basic of respiratory disease” OR “Annual conference of the swiss zoological society a century of zoology history and perspective at zoologia 94” OR “Benelux meeting” OR “Conference on chromosome 2009” OR “Annual conference of the weed science society of america wssa” OR “Biennial symp of the ecological soc of australia on ecological interactions” OR “Conference on community trials for cardiopulmonary health directions for public health practice policy and research” OR “Annual meeting of the american college of medical genetics” OR “Bill e k interdisciplinary beef symposium beef cattle welfare and stress” OR “Conference on current issues of chemical mixtures” OR “Annual meeting of the american genetics association” OR “Biochemical society focused meeting on mechanics and control of cytokinesis” OR “Conference on demography and epidemiology frontiers in population health and aging” OR “Annual meeting of the american phytopathological society joint with the canadian phytopathological society mycological society of america” OR “Biochemical society focused meeting on pancreatic beta cell birth life and death” OR “Conference on ecological and evolutionary ethology of fishes” OR “Annual meeting of the american society of naturalists” OR “Biodiversity of the indian river lagoon conference” OR “Conference on ecological models as decision tools in the 21st century” OR “Annual meeting of the animal behavior society” OR “Bioscience 2005 conference” OR “Conference on end of kuru 50 years of research into an extraordinary disease” OR “Annual meeting of the association for the advancement of industrial crops” OR “Black forest symposium on environmental and ecological parasitology impact of global change” OR “Conference on environmentally induced alterations in development a focus on wildlife” OR “Annual meeting of the association for tropical biology and conservation” OR “Blue crab symposium” OR “Conference on epidemiology and public health” OR “Annual meeting of the canadian phytopathological society” OR “Bovine respiratory disease brd symposium on new approaches to bovine respiratory disease prevention management and diagnosis” OR “Conference on eugenic thought and practice a reappraisal towards the end of the 20th century” OR “Annual meeting of the canadian society of animal science” OR “Brazilian symposium on bioinformatics bsb 2005” OR “Conference on evolution ecology and management of scaphirhynchus” OR “Annual meeting of the crop science

society of america” OR “British society for parasitology bsp autumn symposium on the multidisciplinarity of parasitology host parasite evolution in an ever changing world” OR “Conference on food and forestry global change and global challenges” OR “Annual meeting of the deutschen gesellschaft fur zuchtungskunde e v” OR “British society for parasitology symposium on transmission cycles in protozoan parasites” OR “Conference on future directions in air quality research ecological atmospheric regulatory policy economic and educational issues” OR “Annual meeting of the national coastal oceanography programme national research project on coastal zones” OR “Cdc atsdr workshop on the use of race and ethnicity in public health surveillance” OR “Conference on genetically modified crops” OR “Annual meeting of the national council for radiation protection ncrp” OR “Cell biology symposium molecular basis for feed efficiency” OR “Conference on genomics genetics and society bridging the disciplinary divides” OR “Annual meeting of the quaternary research association” OR “Centre for veterinary medicine” OR “Conference on insects and environment” OR “Annual meeting of the regional network asian schistosomiasis and other zoonotic helminthiases rnas” OR “Centre for veterinary medicine meeting” OR “Conference on longevity regeneration and optimal health” OR “Annual meeting of the royal statistical society” OR “Cgiar science forum 2009” OR “Conference on making sense of the metabolome specia” OR “Annual meeting of the sociedad de genetica de chile” OR “Colloquium on biology and preservation of pollen fundamental and applied aspects” OR “Conference on managing for biodiversity emerging ideas for the electric utility industry” OR “Annual meeting of the society for comparative and integrative biology” OR “Colloquium on longevity and ageing” OR “Conference on marine and freshwater invasive species ecology impact and management mfis” OR “Annual meeting of the society for integrative and comparative biology sicb symposium on new frontiers from marine snakes to marine ecosystems” OR “Conference on mathematical methods for applications” OR “Discussion meeting on the orgins of hiv and the aids epidemic” OR “Ices symposium on population dynamics of calanus in the north atlantic” OR “Conference on molecular mechanisms of neurodegeneration” OR “Discussion meeting on the origin and control of pandemic influenza” OR “Ii north european symposium on the ecology of small and medium sized carniwores” OR “Conference on molecular physiology ii engineering crops for hostile environments” OR “Ecomorphology joint meeting of anatomical society and primate society of great britain psgb on primate ecomorphology” OR “Ilsi europe workshop on the significance of excursions of intake above the acceptable daily intake adi” OR “Conference on molecular strategies in biological evolution” OR “Ecsa 46 international conference on wadden sea changes and challenges in a world heritage site” OR “Inaugural wool industry science and technology conference” OR “Conference on molluscan conservation a strategy for the 21st century” OR “Encounters ichthyology in france conference” OR “Independent meeting on signalling the future” OR “Conference on morphogenesis in ontogeny and evolution heterochronies heterotopies and allometry” OR “Environment and pyrenees international conference epic” OR “Integration of ice core marine and terrestrial records intimate workshop on terrestrial records from central eastern europe for the last glacial interglacial transition” OR “Conference on mountain forest management in a changing world” OR “Esccap vector borne diseases vbd symposium” OR “International belyaev conference on genetics and evolution plant biology” OR “Conference on neural tube defects” OR “Eug 8 meeting” OR “International colloquium on epidemiology and control of bovine theileriosis” OR “Conference on new directions in pharmacogenetics and ecogenetics genetic defenses

against environmental impacts responses to infections foods and environmental toxicants” OR “Euring 94 conference” OR “International conf on climate of the northern latitudes past present and future” OR “Conference on particulate matter supersites program and related studies” OR “European conference on artificial evolution ae 95” OR “International conference of computational methods in sciences and engineering iccmse 2004” OR “Conference on physics of random networks econophysics and models of biophysics and sociophysics” OR “European society of nematologists organises biannual international symposia on soil microbial interactions and communities” OR “International conference of immunogenomics and immunomics” OR “Conference on proposals for the responsible use of racial and ethnic categories in biomedical research” OR “European vertebrate pest management conference” OR “International conference of the environment public authority on conservation biodiversity in the arid regions” OR “Conference on reproductive science in 2010” OR “Evoworkshops 2003 conference” OR “International conference of the insect toxicology 2000” OR “Conference on resistance 97 integrated approach to combating resistance” OR “Ewac conference on cereal aneuploids for genetical analysis and molecular techniques” OR “International conference on artificial neural networks icann 2001” OR “Conference on resource capture by crops integrated approaches” OR “Fenner conference on wildlife population dynamics and management” OR “International conference on biology and conservation of prosimians” OR “Conference on restore regenerate revegetate restoring ecological processes ecosystems and landscapes in a changing world” OR “Festschrift symposium 2007 held in honor of m michael cohen” OR “International conference on computational intelligence and security” OR “Conference on salinity solutions” OR “Fisheries society of the british isles annual symposium on ichthyoplankton ecology” OR “International conference on computational science and its applications iccsa 2006” OR “Conference on science into policy improving uptake and adoption of research” OR “Fisheries society of the british isles annual symposium on predator prey relations in fishes” OR “International conference on computational science iccs 2003” OR “Conference on the biogeography of southeast asia 2000 organisms and orogenesis” OR “Future forum conference” OR “International conference on conservation agriculture and sustainable land use caslu” OR “Conference on the ecology and management of large native pinewoods” OR “Genetic analysis workshop 10 gaw10” OR “International conference on conservation and management of arctic charr” OR “Conference on the effects of climate change on plants” OR “Genetic analysis workshop 9 analysis of complex oligogenic traits gaw9” OR “International conference on cyberworlds” OR “Conference on the evolution of infectious agents in relation to sex” OR “Genetic manipulation of photosynthesis session at the annual meeting of the society for experimental biology” OR “International conference on distribution taxonomy and genetic status of the european species of the genus gobio” OR “Conference on the optimisation of water use by plants in the mediterranean” OR “Geological association of canada nuna meeting nunavut environment assessment transect on looking to the future nunavut environments past and present” OR “International conference on emerging infectious diseases” OR “Conference on the reproductive tract and hiv 1 transmission” OR “Great lakes bioinformatics conference glbio” OR “International conference on environmental research and technology ecoimpuls” OR “Conference on the role of neural plasticity in chemical intolerance” OR “Greenhouse gas and animal agriculture conference ggaa2016” OR “International conference on forest vegetation management” OR “Conference on theoretical fundamentals of consistent applications in

environmental management” OR “Groningen conference on restoration ecology” OR “International conference on geobiology” OR “Conference on woodland birds their ecology and management” OR “Hawaii world conservation congress” OR “International conference on immunogenetic risk assessment in human disease” OR “Congress of the british cattle veterinary association bcva” OR “Health and environmental sciences institute workshop on evaluating biological variation in non transgenic crops” OR “International conference on industrial crops promoting sustainability 28th annual meeting of the association for the advancement of industrial crops aaic” OR “Congress of the croatian society of biochemistry and molecular biology” OR “Herbicide resistance workshop” OR “International conference on intelligent computing” OR “Consultation on community genetic services and a regional network of medical genetics in latin america” OR “Hugo mutations in the human genome meeting” OR “International conference on livestock and global climate change” OR “Cssa annual meeting 2003” OR “Ices conference on is there more to eels than slime” OR “International conference on loaches of the genus cobitis and related genera” OR “Dana conference on conservation and mobile peoples” OR “Ices science conference on monkfish angelfish across the world common problems and common solutions” OR “International conference on micro and macro systems in life sciences” OR “Discussion meeting of the royal soc of london the evolution and design of animal signalling systems” OR “Ices scor symposium on ecosystem effects of fishing” OR “International conference on molecular architecture of evolution primary and secondary determinants” OR “Discussion meeting of the royal society on epigenesis versus preformation during mammalian development” OR “International conference on northern greece and southeastern eurpe during the neolithic period an interaction zone” OR “International symposium on aquatic vascular plants” OR “International workshop on diversification in inland finfish aquaculture difa” OR “International conference on plant response and adaptation to anaerobiosis” OR “International symposium on autoimmunity induced by infection” OR “Immunization” OR “International workshop on drip loss and water holding capacity of porcine meat” OR “International conference on rabies in americas” OR “International symposium on biodiversity in agriculture for a sustainable future” OR “International workshop on ecophysiology and genetics of trees and forests in a changing environment” OR “International conference on radioecology and environmental radioactivity icrer” OR “International symposium on biology ecology and management of worlds worst plant invasive species” OR “International workshop on from particle size to sediment dynamics” OR “International conference on rickettsiae and rickettsial diseases” OR “International symposium on carcinogenic risks due to ionizing radiations” OR “International workshop on human caliciviruses” OR “International conference on scientific aspects of coral reef assessment monitoring and restoration” OR “International symposium on culture collections of algae” OR “International workshop on improving fish feed and feeding techniques fish processing labeling and marketing of fish products” OR “International conference on sturgeon biodiversity and conservation” OR “International symposium on declining fertility in dairy cows in the world” OR “International workshop on management of tropical coastal fisheries in asia” OR “International conference on systematics and evolution of the ranunculiflorae” OR “International symposium on diaupause in the crustacea” OR “International workshop on methods of research on soil structure soil biota interrelationships” OR “International conference on the ecology of estuaries and soft sediment habitats” OR “International symposium on ecology and management of northern goshawks held in

conjunction with annual meeting of the raptor research foundation” OR “International workshop on multilevel optimization algorithms and applications” OR “International conference on the nutrional enhancement of plant foods” OR “International symposium on eurasian ruffe gymnocephalus cernuus biology impacts and control” OR “International workshop on phytoremediation environmental and molecular biological aspects” OR “International conference on topics in biomathematics and related computational problems at the beginning of the third millennium” OR “International symposium on exposure and risk assessment with respect to contaminated soil” OR “International workshop on pond aquaculture in central and eastern europe in the 21st century” OR “International conference on topics in biomathematics and related computational problems biocmp 2002” OR “International symposium on fish and their habitat” OR “International workshop on potential impacts of climate change on tropical forest ecosystems” OR “International conference on vaccines for enteric diseases ved 2001” OR “International symposium on high mountain lake and streams indicators of a changing world” OR “International workshop on risk assessment of threatened species” OR “International conference on water saving agriculture and sustainable use of water and land resources” OR “International symposium on infectious agent transmission model building” OR “International workshop on the biological bases for aquaculture of siluriformes basil” OR “International conference on zoology of 50 years department of zoology” OR “International symposium on management of mycorrhizas in agriculture horticulture and forestry” OR “International workshop on the restoration of fish populations” OR “International congress for plant pathology 2008 icpp” OR “International symposium on mangroves” OR “International workshop on zoo animal welfare” OR “International congress in the wake of the double helix” OR “International symposium on methods for the assessment of sediment toxicity using zebrafish danio rerio” OR “Interspecies hybrids in mammals symposium australasian gene mapping workshop” OR “International congress of carnivorous plants” OR “International symposium on mosaicism in human skin” OR “Iufro xx world congress” OR “International congress on carnosine in exercise and disease” OR “International symposium on perspectives of biomedical research in the xxist century” OR “Iv european issx meeting on toxicological evaluation of chemical interactions relevance of social environmental and occupational factors” OR “International issues conference on biodiversity” OR “International symposium on recent advances in animal nutrition” OR “Ivth international scientific meeting of the cassava biotechnology network” OR “International larval fish conference” OR “International symposium on recent advances in benzene toxicity” OR “Ix international colloquium on apterygota” OR “International meeting on radiation processing imrp” OR “International symposium on renewable energy and sustainability isres” OR “Ixth international conference of plant embryologists” OR “International meeting on the state of fisheries” OR “International symposium on speciation from diversification to reproductive isolation” OR “Joint annual conference of the geological society of new zealand new zealand geophysical society” OR “International morphology symposium and workshop” OR “International symposium on species concepts and biodiversity” OR “Joint annual meeting of the american mathematical society mathematical association of america” OR “International nuclear atlantic conference inac 2005” OR “International symposium on the centennial of the discovery of chargas disease” OR “Joint bmb 15 ecsa 27 symposium” OR “International orchid workshop” OR “International symposium on the role of biological nitrogen fixation in sustainable agriculture for the tropics”

OR “Joint conference of the society of australian systemic biologists australasian evolution society” OR “International pike symposium held at the 136th annual meeting of the american fisheries society” OR “International symposium on theory to practice in honor of professor skjervold” OR “Joint meeting of the 6th international rice genetics symposium 7th international symposium of rice functional genomics” OR “International plankton symposium” OR “International workshop on approaches to improve the utilization of food feed crops” OR “Joint meeting of the american dairy science association amer society of animal science and the canadian society of animal science” OR “International plant disease epidemiology workshop” OR “International workshop on aquaculture application of controlled drug and vaccine delivery” OR “Joint meeting of the australian society for parasitology 8th international coccidiosis conference” OR “International society of root research 5th symposium on root demographics and their efficiencies in sustainable agriculture grasslands and forest ecosystems” OR “International workshop on artificial neural networks” OR “Joint meeting of the royal soc and the academie des sciences cellular recognition and interaction” OR “International symp on acremonium grass interactions” OR “International workshop on biodiversity and ecosystem function in marine ecosystems” OR “Joint meeting of the spe western regional aapg pacific section” OR “International symp on mussels biology and cultivation of mussels” OR “International workshop on capillary electrophoresis in the forensic sciences” OR “Joint symposium of the thai and japanese society for animal reproduction society for reproduction and development” OR “International symposium of chylomicrons in disease” OR “International workshop on differential equations and dynamical systems” OR “Joint symposium plants from high altitude phytochemistry and bioactivity” OR “International symposium on advanced biochemical and molecular approaches to sea bream aquaculture and its impact on the environment” OR “Joint united states australia workshop on recruitment and population dynamics of coral reef fishes reefish 95” OR “National meeting of the entomological society of america” OR “Symp on the development of herbicide resistant crop cultivars at the 1991 annual meeting of the weed science soc of america” OR “Larvi 95 symposium” OR “National seminar on designing crops for the changing climate” OR “Symp on the pathological conditions of wild salmonids” OR “Life sciences conference 1998 signalling concepts in life sciences” OR “Nbic convergence 2003 conference on converging technologies for improving human performance” OR “Symposium honoring the careers of ross and joyce bell and their contributions to scientific work” OR “Meb conference on new frontiers for monitoring european biodiversity the role and importance of amphipod crustaceans” OR “Nigel barlow symposium on the practical applications of ecological theory and modelling” OR “Symposium in honor of professor john croxall held at the british antarctic survey” OR “Meeting of the american society of agronomy” OR “Ontology and methodology conference” OR “Symposium of advances in genomics epidemiology and statistics sages” OR “Meeting of the british soc for development biology” OR “Pattern recognition in practice v conference” OR “Symposium of section xi of wssa on ecophysiological approaches in the development of weed management strategies at the wssa annual meeting” OR “Meeting of the cost action 8 22 work group on somaclonal variation and recalcitrance to regeneration” OR “Plant microbial interaction conference 2008” OR “Symposium of the american society of naturalists” OR “Meeting of the european science foundation canopy research programme” OR “Proceedings of the 7th african small mammal symposium” OR “Symposium of the society for conservation biology” OR “Meeting of the german veterinary societys

study group on small animal illnesses” OR “Proceedings of the fifth pan american symposium on animal plant and microbial toxins” OR “Symposium on aerial dispersal of pests and pathogens implications for developing and deploying integrated pest management strategies at a joint aps esa symposium” OR “Meeting of the ieg 40 group” OR “Research conference crc for sheep industry innovation” OR “Symposium on alpine and polar lichenology at the 5th international mycological congress” OR “Meeting of the panafrican archaeological association for prehistory and related studies paa meeting of the society of africanist archaeologists safa” OR “Resistance 2001 conference” OR “Symposium on apomixis and taxonomy” OR “Meeting of the philosophy of science association psa” OR “Royal society of tropical medicine and hygiene meeting jointly with the royal entomological society” OR “Symposium on behavioral and physiological adaptation to urban environments at the annual meeting of the society for integrative and comparative biology” OR “Meeting on conservation and monitoring of pond biodiversity” OR “Scientific conference dedicated to d k belyaev” OR “Symposium on behaviour and welfare of extensively farmed animals” OR “Meeting on evolution genomics and bioinformatics” OR “Scientific conference on the application of scientific knowledge to decisionmaking in managing forest ecosystems” OR “Symposium on beyond the mean biological impacts of changing patterns of temperature variation at the annual meeting of the society for integrative and comparative biology” OR “Meeting on mechanisms regulating gene flow in flowering plants” OR “Scientific meeting on geoecology in extreme environments mountains and semiarid areas” OR “Symposium on bias and completeness in the conodont fossil record held at the 8th international conodont symposium” OR “Meeting on orchid population biology conservation and challenges” OR “Scientific workshop on state of the research on red tide in the gulf of mexico” OR “Symposium on biobehavioral mechanisms of work related upper extremity disorders” OR “Meeting on plant genome horizons vistas and visions” OR “Secotox world congress 6th european conference on ecotoxicology and environmental safety” OR “Symposium on biodiversity systematics and conservation” OR “Meeting on radiation induced mutations and other advanced technologies for the production of crop mutants suitable for environmentally sustainable agriculture” OR “Seminar in proteomics uco 2003” OR “Symposium on biological nitrogen fixation for sustainable agriculture at the 15th congress of soil science” OR “Meeting on re introduction of endangered plant species” OR “Seminar of the french soc of theoretical biology” OR “Symposium on biology of plant reproduction” OR “Meeting on redox metabolism in malaria from genes to drugs” OR “Seminar on anchovy and its environment” OR “Symposium on biovigilance a framework for effective pest management held during the annual meeting of the canadian phytopathological society” OR “Meeting on reproduction ecology and evolution in marine systems held in honor of john s pearse” OR “Serono symposium on the disapperaring male” OR “Symposium on caviar production recent developments and future trends in breeding conservation and product processing of sturgeons” OR “Meeting on surface display and peptide libraries” OR “Session on ecology and siliviculture of the north american northern boreal forest understanding the past and charting the future at the cif saf joint annual meeting” OR “Symposium on census of antarctic marine life diversity and change in southern ocean ecosystems” OR “Meeting on the archaeology of the pleistocene holocene transition” OR “Sixth international workshop on the fragile x and x linked mental retardation” OR “Symposium on circulation at the 36th annual meeting of the canadian federation of biological societies” OR “Meeting on the future for the genus swietenia in its native forests is there a future for

mahogany” OR “Society for experimental biology annual meeting” OR “Symposium on climate variability and ecosystem impacts on the north pacific a basin scale synthesis” OR “Meeting on the implication of past and present landscape patterns for diversity research” OR “Spring conference of the british ornithologists union” OR “Symposium on conserved genes and developmental mechanisms in embryos of divergent species at the annual meeting of the american society of zoologists” OR “Meeting on the scientific basis for management of salmonid stocks in the british isles” OR “Spring meeting of the european journal of pharmacology” OR “Symposium on current knowledge of wood deterioration mechanisms and its impact on biotechnology and wood preservation” OR “Meeting on toxoplasma centennial congress from discovery to public health management” OR “Sustainable asia conference 2014” OR “Symposium on current status of sexing mammalian sperm” OR “Meeting on winter processes in arctic tundra ecosystems” OR “Symp at the midwestern meeting of the american soc of animal science national programs for genetic improvement of us herds and flocks” OR “Symposium on current themes in pharmaceuticals and agrochemicals principles and differences” OR “Meeting parasite variation immunological and ecological significance” OR “Symp on biochemical genetics and taxonomy of fish” OR “Symposium on diabetes update new developments in pathophysiology and treatment of niddm” OR “Mid term meeting of fish and land inland water ecotones on the importance of aquatic terrestrial ecotones for freshwater fish” OR “Symp on endangered bryophytes in europe causes and conservation” OR “Symposium on dynamic cropping systems for soil and water conservation” OR “Middle palaeolithic in the desert ii conference” OR “Symp on global environmental change and health” OR “Symposium on ecological implications of transgenic plant release” OR “Miguel r covian symposium” OR “Symp on management of postharvest ecosystems current and future trends at the 1990 annual meeting of the entomological soc of manitoba” OR “Symposium on ecology of long distance movements” OR “National academy of sciences colloquium on plants and population is there time” OR “Symp on population dynamics of plant inhabiting mites” OR “Symposium on emerging stresses on forests and agro ecosystems resulting from climate change held during the annual meeting of the canadian phytopathological society” OR “National beef science seminar” OR “Symposium on estrogens in the environment iii global health implications” OR “Symposium on physiology and disease” OR “Viith international symposium on the biology of the turbellaria” OR “Symposium on ethnobotany held at the 42nd annual meeting of the canadian botanical association” OR “Symposium on phytosanitary management of the major crops cereals pulses and sugar plants” OR “Vth international seminar on apterygota” OR “Symposium on evolution and phylogeography of arctic and alpine plants in europe” OR “Symposium on plant gene resources at the annual conference of the agricultural institute of canada” OR “Vth latin american workshop on non linear phenomena 11th medyfinol conference on statistical physics of dynamic and complex systems” OR “Symposium on evolution function development causation tinbergen s four questions and contemporary animal biology” OR “Symposium on prehistoric skeletal biology in island ecosystems at the 64th annual meeting of the american association of physical anthropologists” OR “W 168 symposium on seed biology and technology applications and advances” OR “Symposium on genetic diversity and evolution” OR “Symposium on rare plants of the southwest at the annual meeting of the southwestern association of naturalists” OR “William donald hamilton 1936 2000 memorial symposium” OR “Symposium on genome analysis and the molecular systemstics of retroelements” OR “Symposium on regional assessment of

freshwater ecosystems and climate change in north america” OR “Workshop at the 5th australian agronomy conf ideotypes and physiology tailoring plants for increased production” OR “Symposium on glyphosate held at the 252nd annual fall meeting of the american chemical society acs” OR “Symposium on responses of terrestrial arthropods to variation in the thermal and hydric environment at meeting of the society for integrative and comparative biology” OR “Workshop mechanized harvest of southern highbush blueberries for the fresh market” OR “Symposium on granitic systems” OR “Symposium on science as a way of knowing biodiversity at the annual meeting of the american society of zoologists” OR “Workshop of the global partnership for plant conservation gppc on plant conservation and the sustainable development goals sdgs” OR “Symposium on guayule in honor of francis nakayama held at the aaic annual meeting” OR “Symposium on scientific integration of western medicine and complementary alternative mind body medicine” OR “Workshop on agriculturally important toxigenic fungi” OR “Symposium on herbicide resistant crops from biotechnology held at the acs 227th national meeting” OR “Symposium on seed fates importance for structuring plant populations and communities at the botanical society of america meeting” OR “Workshop on aspergillus systematics in the genomic era” OR “Symposium on historical patterns of developmental integration at the annual meeting of the american society of zoologists” OR “Symposium on status of atlantic salmon” OR “Workshop on assessing health risks from environmental exposure to chemicals” OR “Symposium on implications of endocrine active substances for humans and wildlife” OR “Symposium on tapping the power of crustacean transcriptomics to address grand challenges in comparative biology at the annual meeting of the society for integrative and comparative biology” OR “Workshop on atmospheric change and biodiversity formulating a canadian science agenda” OR “Symposium on life history strategies at the meeting of the canadian society of zoologists” OR “Symposium on the athletes heart” OR “Workshop on bird migration in relation to climate change” OR “Symposium on linking genes and morphology in vertebrates” OR “Symposium on the biology and control of reproductive processes” OR “Workshop on cephalopod growth held in conjunction with 2003 cephalopod intenational advisory council” OR “Symposium on managing wetlands for waterbirds held at the 24th annual meeting of the waterbird society” OR “Symposium on the contribution of animal behavior studies to zoo propagation programs” OR “Workshop on context dependence in plant herbivore interactions” OR “Symposium on manipulation of the rumen fermentation for enhanced animal performance at the 75th asas southern section meeting” OR “Symposium on the contribution of long term studies to conservation” OR “Workshop on exotic invaders of the north sea shore” OR “Symposium on maternal effects on early life history their persistence and impact on organismal ecology at the annual meeting of the american society of zoologists” OR “Symposium on the ecology and conservation of spatially structured populations” OR “Workshop on experimental assessment of the toxicological effects of inhaled complex mixtures on the respiratory systems” OR “Symposium on methods for monitoring effects of pesticides at the 15th annual meeting of the society of environmental toxicology and chemistry” OR “Symposium on the importance of weed biology to weed management at the weed science society of america meeting” OR “Workshop on forage genetic resources meeting the requirements of industry” OR “Symposium on molecular biological methods in food microbiology” OR “Symposium on the mosaic of autoimmunity held in honor of yehuda shoenfelds 60th birthday” OR “Workshop on international research and development on stylosanthes” OR “Symposium on molecular techniques and molluscan phylogeny at the

11th international malacological congress” OR “Symposium on the use of molecular markers in plant population biology” OR “Workshop on long term ecological research current state and perspectives in the central and eastern europe” OR “Symposium on molluscan biogeography perspectives from the pacific ocean joint annual meeting of the western society of malacologists 76th annual meeting of the american malacological society” OR “Symposium on them s the brakes the past and future of north american bamboo” OR “Workshop on low p farming systems” OR “Symposium on molluscs as models in evolutionary biology held at the world congress of malacology” OR “Symposium on tobacco mosaic virus pioneering research for a century” OR “Workshop on metabolic disorders in the pathogenesis of nervous system damage in hiv infected drug abusers” OR “Symposium on morphological innovation” OR “Symposium on understanding microevolution and development in the arthropods” OR “Workshop on natureceuticals nutraceuticals herbal botanicals and psychoactives drug discovery and drug drug interactions” OR “Symposium on nanostructured biological materials held at the 5th meeting of the brazilian materials research society” OR “Symposium on understanding the evolution of endocrine system variation through large scale comparative analyses at the annual meeting of the society for integrative and comparative biology” OR “Workshop on ostertagia” OR “Symposium on national animal germplasm program at the asas 88th annual meeting” OR “Symposium on weed ecology in long term experiments held at the wssa 2002 annual meeting” OR “Workshop on outcomes of genome genome interactions” OR “Symposium on native plants as bioindicators of air pollutants held in conjunction withe the 34th air pollution workshop” OR “Symposium on wetland feeding site use by white ibises eudocimus albus breeding in coastal south carolina” OR “Workshop on phoma stem canker durable resistance” OR “Symposium on natural hazards and natural disturbances in mountain forest challenges and opportunities for silviculture” OR “Tcs conference” OR “Workshop on potential role of wild fruit tree and other food tree species for niutrition poverty alleviation and biodiversity conservation in sub saharan africa” OR “Symposium on new aspects in the biology and systematics of the mediterranean flora” OR “The crisis in invertebrate conservation symposium at the annual meeting of the american society of zoologists canadian society of zoologists” OR “Workshop on progress in molecular studies of lichens” OR “Symposium on new zealand seeds” OR “Thematic symposium of the southern african ornithological society migration dispersal and nomadism” OR “Workshop on rebuilding techniques for abalone in british columbia” OR “Symposium on northern dimension to biodiversity from arctic to boreal environments” OR “Unitas malacologica american malacological society symposium on interactions between man and molluscs” OR “Workshop on research in zoos from behaviour to sex ratio manipulation” OR “Symposium on pathogens and diseases of fish in aquatic ecosystems implications in fisheries management” OR “Us ireland functional food conference” OR “Workshop on southeast alaska marine biology and oceanography” OR “Symposium on patterns and process of morphological integration in primate and human evolution held at 77th annual meeting of the american association of physical anthropologists” OR “Vi international symposium on aquatic oligochaetes” OR “Workshop on spatial ecology of insect plant interactions” OR “Symposium on peopling of the americas genetic anthropological and archeological studies” OR “Viiith congress of the world association for animal production” OR “Workshop on the biology and conservation of caladenia” OR “Symposium on physical and genetic mechanisms for evolutionary novelty at the annual meeting of the society for integrative and comparative biology” OR “Workshop on the role of

intercellular communications in major tissue processes metabolic electrical and informational cooperation of excitable and non excitable cells” OR “World palms sympsoium” OR “Zinc crops 2007 conference” OR “World congress of the international association of landscape ecologists” OR “Xi world forestry congress” OR “Zooplankton ecology symp” OR “World congress on natural resource modeling”)
